# Synthesis of 1,3-Dienes from Alkenes via Alkenyl Thianthrenium Salts

**DOI:** 10.1021/jacs.5c22101

**Published:** 2026-03-25

**Authors:** Sven Müller, Nicolai Klask, Aboubacar Daff, Tobias Ritter

**Affiliations:** † 28314 Max-Planck-Institut für Kohlenforschung, Kaiser-Wilhelm-Platz 1, 45470 Mülheim an der Ruhr, Germany; ‡ Institute of Organic Chemistry, RWTH Aachen University, Landoltweg 1, 52074 Aachen, Germany

## Abstract

Herein, we show the conversion of alkenes into functionalized 1,3-dienes in two steps. First, C–H thianthrenation is used to access a wide pool of alkenyl electrophiles directly from alkenes. Second, the alkenyl thianthrenium salts undergo a Pd-catalyzed three-component coupling reaction with allene gas and nonorganometallic nucleophiles to afford 1,3-dienes. This method provides a robust and modular platform for accessing structurally complex 1,3-dienes that would be challenging to access otherwise.

## Graphical abstract

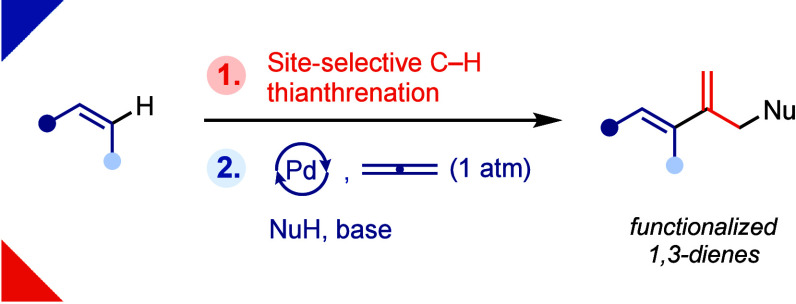

1,3-Dienes are synthetically useful motifs that provide entry points for cycloadditions like the Diels–Alder reaction, transition metal-catalyzed 1,2- and 1,4-difunctionalization reactions, and radical addition reactions.^1,2^ Commercially available 1,3-dienes are scarce and structurally simple. Catalytic methods for the synthesis of 1,3-dienes mainly rely on alkenyl electrophiles, which cannot be readily accessed from olefins, thereby limiting the chemical space available from them. Thus, a general and practical method for the transformation of alkenes to synthetically valuable 1,3-dienes remains elusive. Here, we address this challenge and show how alkenes can be used for the synthesis of 1,3-dienes via conversion into alkenyl thianthrenium salts (alkenyl-TT salts). We demonstrate for the first time how the various previously reported reaction pathways^3^ of alkenyl-TT salts with nitrogen-, carbon-, and sulfur-nucleophiles can be circumvented in favor of densely functionalized 1,3-diene formation via palladium catalysis, most likely through a Pd π-allyl alkylation pathway. The transformation is characterized by high modularity, robustness, and operational simplicity. A broad range of cyclic, acyclic, monosubstituted, and disubstituted alkenes are amenable to conversion into 1,3-dienes that are outside the reach of previous methods.

Our group^4^ and the Wickens^5^ group have previously reported the synthesis of alkenyl-TT salts from alkenes. C–H thianthrenation proceeds site-selectively at the most electron-rich position of the most electron-rich double bond and does not require the presence of directing groups. Therefore, alkenyl-TT salts unlock access to a large pool of alkenyl electrophiles, directly available from olefins in a single step. Like aryl-TT salts, alkenyl-TT salts are competent alkenyl electrophiles in transition metal-catalyzed reactions.^3,6^ Under palladium catalysis, the use of alkenyl-TT salts in Negishi-, Sonogashira-, Suzuki- and Heck-type cross-coupling reactions has been reported (Scheme 1A, top left).^4,7,8^ In the absence of transition metals, alkenyl-TT salts have been reported to undergo polar reactions with various non-organometallic nucleophiles, and these reactions rely on the alkenyl-TT to act either as an electrophile or acid in the first step of the sequence (Scheme 1A, bottom left).^3^ Alkenyl-TT salts are known to undergo aziridination^9,10^ and diamination^11,12^ reactions with amines, *cine*-substiution reactions with sulfinates,^13^ and cyclopropanation reactions with carbon nucleophiles.^10,14^ The synthesis of allylic amines^15–17^ from alkenyl-TT salts has been reported among other transformations.^18^ However, to date, the question of which of the many pathways mentioned above dominates in a transition metal-catalyzed reaction of alkenyl-TT salts in the presence of amines, sulfinates, and carbanions as nucleophiles has not yet been investigated in the literature (Scheme 1A, right). Based on this observation, we devised a reaction that combines these three components with the goal of achieving the synthesis of 1,3-dienes. Established methods for *de novo* syntheses of 1,3-dienes include alkyne dimerization reactions,^19,20^ enyne cross-metathesis,^21–23^ oxidative Heck reactions,^24^ and Kumada- ^25–28^ /Suzuki- ^29,30^ /Heck-type^31^ cross-coupling reactions, among others^32–35^ (Scheme 1B). At its core, the development of catalytic methods for the synthesis of 1,3-dienes is complicated by two main challenges: the control of stereochemistry and diene diversity. The first challenge is a result of the fact that 1,3-dienes contain two carbon–carbon double bonds, which can each be either *E*- or *Z*-configured and are linked together by a C–C single bond. In oxidative Heck reactions, for example, the configurations of both double bonds are set by the Pd-catalyzed formation of the central C–C single bond from two separate alkene units. However, stereocontrol can often not be achieved solely by the catalyst and directing groups are commonly used.^36^ The second challenge results from the fact that 1,3-dienes are composed of four carbon atoms with a total of eight potentially variable substituents on those carbon atoms. The diversity of 1,3-dienes generated by a given method, therefore, depends on the number of substitution patterns that can be accessed and the variety of substituents that can be incorporated at a given position of the diene. In the case of enyne metathesis, for instance, both the number of accessible substitution patterns and the variety of substitutents that can be introduced is low.^21,37^ Herein, we report a Pd-catalyzed three-component coupling of alkenyl-TT salts, allene gas, and N-, S-, and C-nucleophiles. We show that alkenyl-TT salts react preferentially with a palladium catalyst over other nucleophiles. We demonstrate how alkenyl-TT salts provide a robust and modular platform for accessing 1,3-dienes from alkenes, with both high stereocontrol and control over substituents on three of four carbon atoms of the 1,3-diene.

**Scheme 1:**
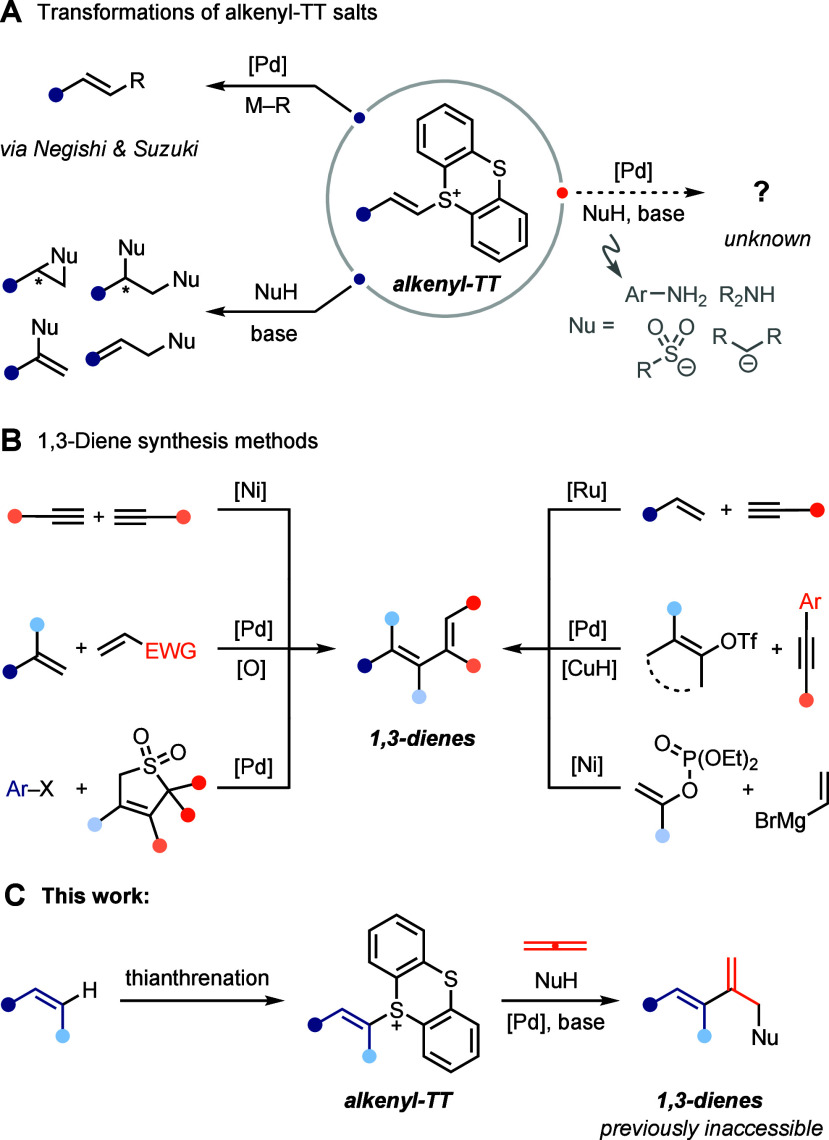
Known Transformations of Alkenyl-TT Salt and Approaches for the Synthesis of 1,3-Dienes

Based on our previous work on Pd-catalyzed reactions of alkenyl-TT salts,^4,7,8^ we designed a plausible catalytic cycle, shown in Scheme 2A. Upon oxidative addition of the alkenyl-TT to Pd^0^, cationic 14-electron alkenyl-Pd complex **I** could be generated. Coordination of allene to complex **I** could generate tetra-coordinate 16-electron complex **II**. Migratory insertion of the Pd–C σ-bond of complex **II** into one of allene’s C–C π-bonds forms cationic π-allyl Pd-complex **III**. Outer-sphere nucleophilic attack on electrophilic complex **III** leads to the formation of the desired 1,3-diene product, and regenerates Pd^0^. The opportunity to form both a C–C bond and a C–X bond in the same reaction distinguishes our design from those of other reaction chemistry. We anticipated three main challenges for the feasibility of our design (Scheme 2B). First, migratory insertion may not be fast enough, leading to premature reductive elimination from complex **I**, affording a functionalized alkene as the product (Scheme 2B top). Second, the nucleophile may not react with electrophilic complex **III**, but rather with the alkenyl-TT itself via Michael addition (Scheme 2B middle). The Wickens and Shu groups have demonstrated that alkenyl-TT salts can act as vicinal dication synthons at ambient temperature in aziridination and cyclopropanation reactions, and alkenyl cation synthons in *cine*-sulfonylation, -cyanation, -amination, and -amidation reactions.^9,10,13,14^ Third, the base may not deprotonate the nucleophile after its attack on complex **III** but rather deprotonate one of the acidic allylic C–H bonds of the alkenyl-TT salt (Scheme 2B bottom). The Wickens group has shown that allylic deprotonation of alkenyl-TT salts leads to double bond isomerization, leading to *Z*-allylic sulfonium ylides, which after protonation of the ylide can undergo nucleophilic substitution.^15^ The same group later reported that allylic deprotonation is irreversible and the only endergonic step in the sequence, with a calculated barrier of 20.9 kcal mol^–1^.^16^ Therefore, the rate of the turnover-limiting step of the catalytic 1,3-diene synthesis has to be faster than the rate of allylic deprotonation in order for the reaction to proceed efficiently.

**Scheme 2:**
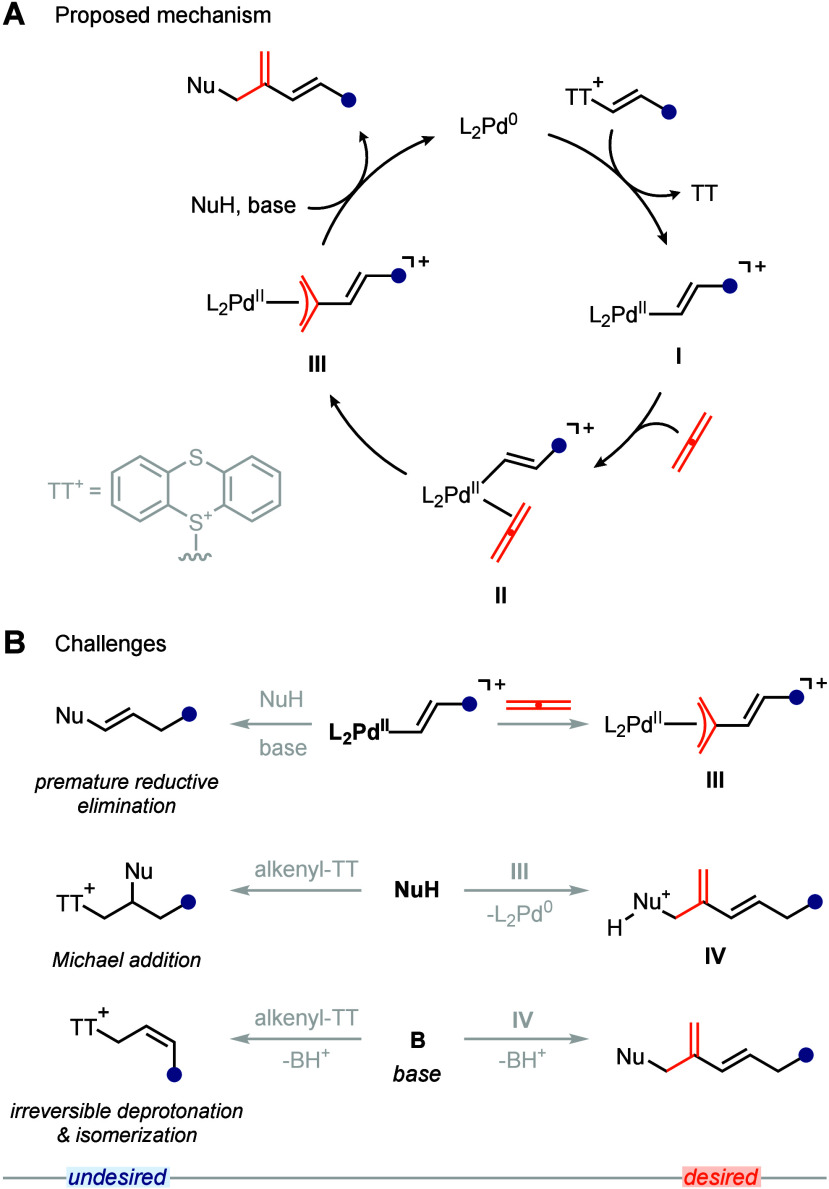
Proposed Mechanism and Anticipated Challenges^a^

a TT = thianthrene.

We started our investigation by examining the reaction of alkenyl-TT salt **TT-1** with aniline in the presence of K_2_CO_3_ and Pd-catalyst under an allene gas atmosphere (Figure 1). The reaction led to the formation of 1,3-diene **1a** in an 81% yield (Figure 1, entry 1). During reaction development, two side products were identified. Allylic amine **1b** arises from double bond isomerization, followed by nucleophilic displacement. Higher palladium loadings and higher loadings of aniline increase the formation of 2,3-dialkylbutadiene **1c**, which is the product of a known allene dimerization reaction (Figure 1, entries 2 and 3).^38^ DMF was chosen as solvent over dioxane to avoid possible solubility issues because, unlike dioxane, DMF can dissolve all alkenyl-TT salts used in this study at 23 °C and concentrations of 0.25 M. A lower reaction temperature would be desirable because propadiene is a flammable gas; however, a reaction conducted at 23 °C led to a yield of 22%, which is not synthetically useful (Figure 1, entry 4). Both Na_2_CO_3_ and K_2_CO_3_ can be used as base; however, Na_2_CO_3_ provided more consistent results over multiple replicates of the same reaction (Figure 1, entry 5, see Tables S6 and S7).

**Figure 1:**
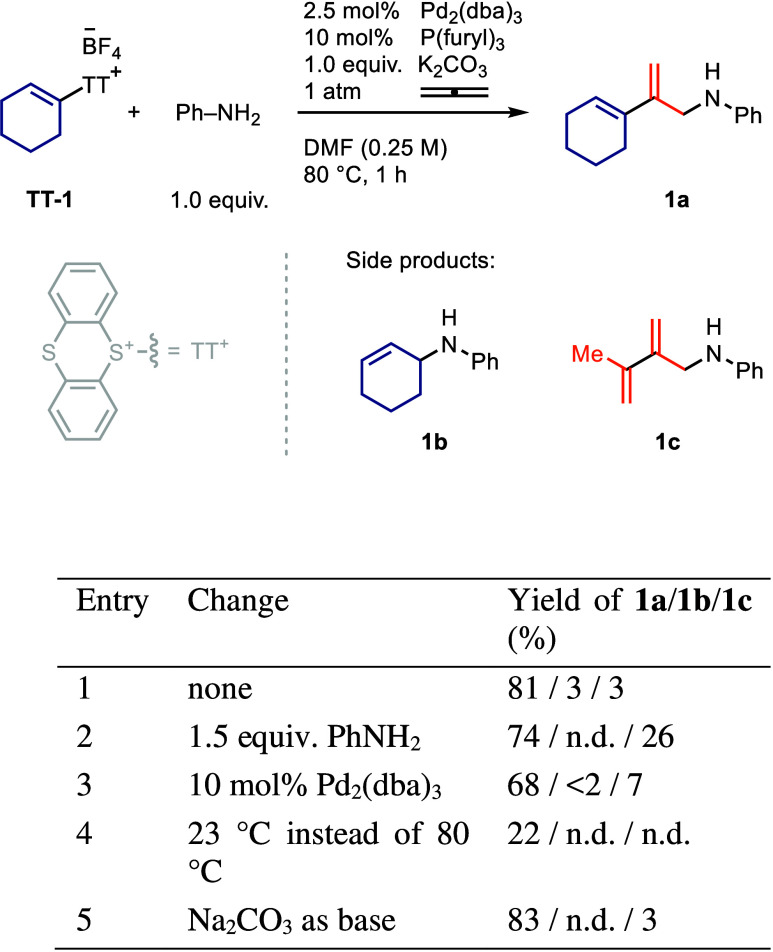
Reaction development. Reactions were conducted on a 0.100 mmol scale. n.d., not detected. Yield was determined by ^1^H NMR spectroscopy.

Among nucleophiles, secondary amines and anilines performed well in the reaction (Figure 2, see **2**–**11**). The reaction is robust and can be scaled up to at least 2 mmol without any decrease in yield (**11a**). Other N-nucleophiles such as uracil (**12**), imides (**13**), sulfimides (**14**) and azide (**15**) can also be used. Sulfinates (**16**, **17**) are competent nucleophiles in the reaction, and products arising from *cine*-substitution on alkenyl-TT salts were not observed. For **TT-16**, additional experiments conducted with **TT-16** and sodium *para*-toluenesulfinate are consistent with *Z-* **TT-16** being converted to *E* **-TT-16** in the reaction mixture, possibly via reversible Michael addition of the nucleophile to **TT-16** (see pages S24–S32). Carbanions generated from malonates (**18a**), malonitriles (**18b**), β-cyano esters ((±)-**18c**), and 1,3-diketones (**18d**) can be used as nucleophiles; however, deprotonation with sodium hydride has to be carried out first. Due to known hazards of mixtures of sodium hydride and DMF, care needs to be taken.^39^ Based on our results, we used Mayr’s nucleophilicity scale^40–42^ to gauge the degree of nucleophilicity of the nucleophile that is required for the reaction to proceed. Among the chemically competent nucleophiles, the lowest value of Mayr’s nucleophilicity parameter is *N* = 10.78 (saccharin anion in MeCN, see **14**),^43^ the highest value is *N* = 20.50 (azide ion in DMSO, see **15**).^44^ This range is agreement with predictions made based on the Mayr equation for Tsuji-Trost-type reactions.^41^ Weaker nucleophiles such as 1,2,5-trimethylpyrrole (*N* = 8.69 in MeCN) and 1-trimethylsiloxycyclohexene (*N* = 5.21 in DCM)^45^ did not afford any of desired product, respectively. 1,3-Substituted dienes (**1**–**9**, **15**, **16**) constitute an important class of 1,3-dienes for which several diversification reactions have been developed.^46–50^ O-nucleophiles like phenol and acetate were ineffective and did not afford 1,3-diene products (**19a**,**b**). The reaction with equimolar amounts of a substituted allene under an argon atmosphere did not provide any desired product (**20**). If migratory insertion were the turnover-limiting step of the catalytic cycle, the presence of an excess of allene might be required in order for the rate of the catalytic reaction to be fast enough to outcompete other pathways. The extent of allylic deprotonation is determined by two main factors. First, the basicity of the nucleophile needs to be considered. The Wickens group has previously demonstrated that, due its low basicity, aniline does not react with alkenyl-TT salts in the absence of external base, while *N*-methylbenzylamine can act as base and nucleophile in the allylic amination of alkenyl-TT salts.^16^ This finding is consistent with our observation that the reaction of the alkenyl-TT salt of *L*-carvone (**TT-21**) with aniline affords 40% of the 1,3-diene **21a** product along with 20% of the allylic amine **21b** (Figure 2, bottom). When a more basic secondary amine is used as the nucleophile, the allylic deprotonation pathway becomes dominant, and allylic amine **21c** was formed in 40% yield. Second, it is necessary to consider the number of allylic protons that can afford a sulfonium ylide after double bond isomerization. Alkenyl-TT salts of monosubstituted and 1,2-disubstituted alkenes can possess up to three allylic protons for which deprotonation, followed by double bond isomerization, leads to a sulfonium ylide. In these cases, allylic amination is suppressed by the faster formation of 1,3-diene (see Figure 2, **1**–**8**). *L*-Carvone-derived **TT-21** has four allylic protons, whose deprotonation can lead to the formation of a sulfonium ylide. In this case, the formation of the 1,3-diene can still be the dominant pathway depending on the choice of the nucleophile. Reactions of alkenyl–TTs that possess five or six allylic protons (see **TT-22**, **TT-23**, Figure 2, bottom), however, did not afford any 1,3-diene products, presumably due to allylic deprotonation becoming fully dominant.

**Figure 2:**
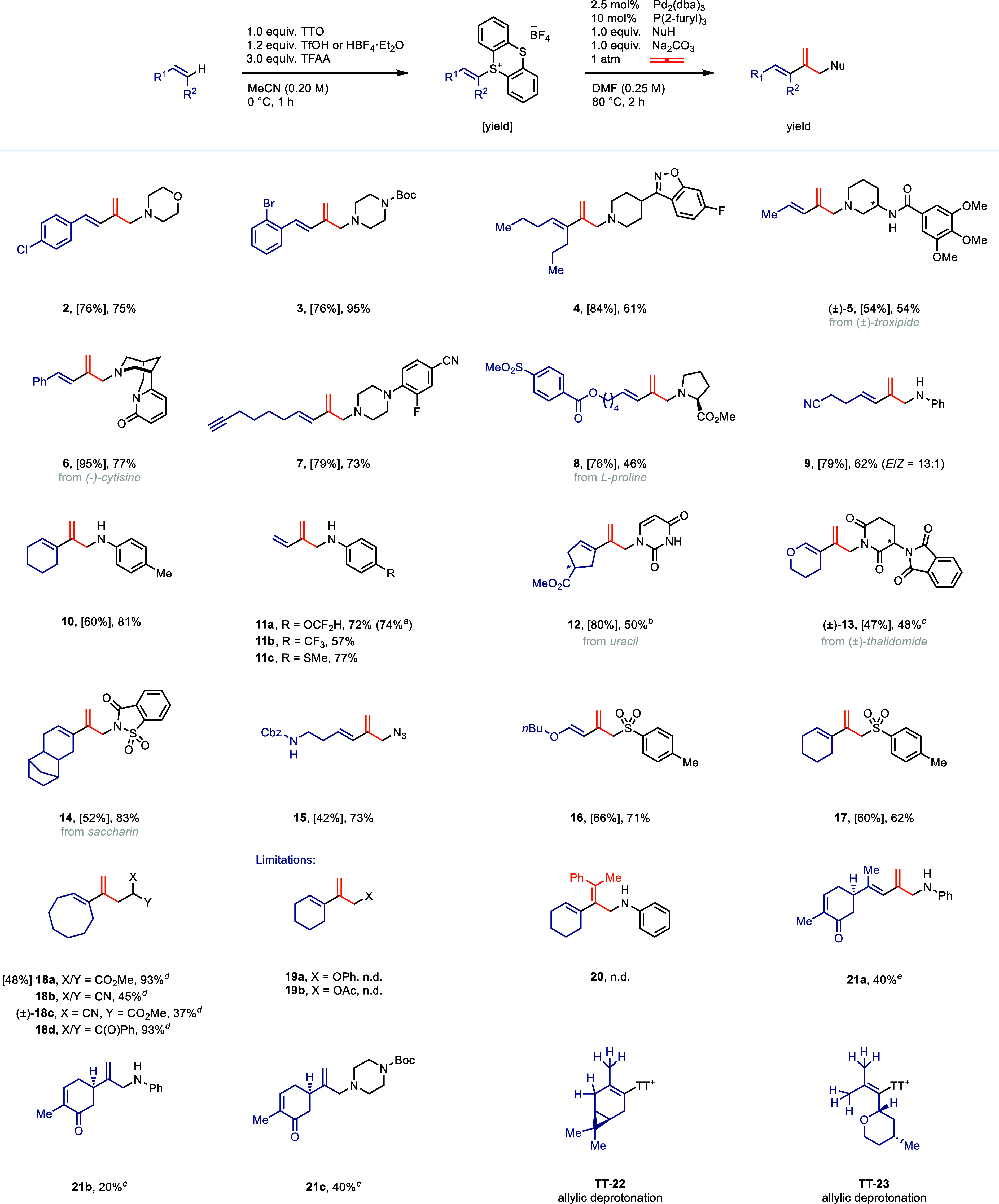
1,3-Diene synthesis scope. Reactions were conducted on a 0.300 mmol scale. TTO, thianthrene-*S*-oxide; TfOH, triflic acid; TFAA, trifluoroacetic anhydride; NuH, nucleophile; n.d., not detected. Legend: ^*a*^reaction was carried out on 2.00 mmol scale; ^*b*^5.0 mol % Pd_2_(dba)_3_, 20 mol % P­(2-furyl)_3_ and K_2_CO_3_ (5.0 equiv) were used; ^*c*^anhydrous dioxane (0.25 M) was used as solvent; ^*d*^1.5 equiv NuH and 1.5 equiv NaH were used; *^e^* yield was determined by ^1^H NMR spectroscopy.

In conclusion, we have developed a new method for the synthesis of functionalized 1,3-dienes directly from alkenes in two steps. This is the first time that N-, C- and S-nucleophiles have been used in a transition metal-catalyzed reaction involving alkenyl-TT salts. Key to this advancement was the fact that the rate of the Pd-catalyzed 1,3-diene synthesis is faster than the rate of irreversible allylic deprotonation and unaffected by reversible Michael addition of the nucleophile to the alkenyl-TT starting material. Thus, previously reported pathways for reactions of alkenyl-TT salts and the respective nucleophiles could be avoided.

## Supplementary Material



## Notes

Experimental procedures, analytical methods, and spectroscopic data (PDF)
