# Supplementary material for: Synthesis of 1,3-Dienes from Alkenes via Alkenyl Thianthrenium Salts
Source: J Am Chem Soc. 2026 Mar 25;148(13):13507–12. doi: 10.1021/jacs.5c22101 (PMC13067272; doi:10.1021/jacs.5c22101)
Supplement: Supplementary file 1 [file ja5c22101_si_001.pdf]

SUPPORTING INFORMATION

Synthesis of 1,3-Dienes From Alkenes via Alkenyl Thianthrenium Salts

Sven Müller[1,2], Nicolai Klask[1,2], Aboubacar Daff[1], and Tobias Ritter[1]*

[1] Max-Planck-Institut für Kohlenforschung, Kaiser-Wilhelm Platz 1, D-45470 Mülheim an der Ruhr, Germany.
[2] Institute of Organic Chemistry, RWTH Aachen University, Landoltweg 1, 52074 Aachen, Germany.

*E-mail: ritter@kofo.mpg.de

TABLE OF CONTENTS

TABLE OF CONTENTS 1

MATERIALS AND METHODS 6

EXPERIMENTAL DATA 8

General procedure for 1,3-diene syntheses from alkenes via alkenyl thianthrenium salts 8

General procedure for the thianthrenation of monosubstituted, 1,2-disubstituted and trisubstituted olefins 8

General procedure for 1,3-diene synthesis from alkenyl thianthrenium salts 8

Optimization of reaction conditions 10

General procedure 10

1,3-Diene product **1a** 10

Allylic amine side product (±)-**1b** 11

Solvent water content 11

Reaction setup 12

Temperature-dependence 12

Palladium loading 13

Base screening 13

Reproducibility screening 14

Solvent screening 16

Ligand screening 16

Pd source screening 18

Allene-dimerization side product **1c** 19

NMR spiking experiments 20

Influence of pressure 22

Mechanistic experiments 24

Michael addition reversibility 24

Michael addition of carbon nucleophiles 31

Preparation of alkenes 33

Synthesis of **Alk-2** 33

Synthesis of **Alk-8** 33

Synthesis of **Alk-12** 34

Synthesis of **Alk-15** 35

Preparation of alkenyl thianthrenium salts 36

Alkenyl thianthrenium salt summary 36

Preparation of thianthrene-S-oxide 37

Alkenyl thianthrenium salt **TT-1** 37

Alkenyl thianthrenium salt **TT-2** 38

Alkenyl thianthrenium salt **TT-3** 39

Alkenyl thianthrenium salt **TT-4** 40

Alkenyl thianthrenium salt **TT-5** 41

Alkenyl thianthrenium salt **TT-6** 41

Alkenyl thianthrenium salt **TT-7** 42

Alkenyl thianthrenium salt **TT-8** 43

Alkenyl thianthrenium salt **TT-9** 44

Alkenyl thianthrenium salt (±)**-TT-12** 45

Alkenyl thianthrenium salt **TT-13** 45

Alkenyl thianthrenium salt **TT-14** 46

Alkenyl thianthrenium salt **TT-15** 47

Alkenyl thianthrenium salt **TT-16** 48

Alkenyl thianthrenium salt **TT-18** 49

Alkenyl thianthrenium salt **TT-21** 50

Alkenyl thianthrenium salt **TT-22** 51

Alkenyl thianthrenium salt **TT-23** 52

Syntheses of 1,3-dienes 53

1,3-Diene **1a** 53

1,3-Diene **2** 54

1,3-Diene **3** 54

1,3-Diene **4** 55

1,3-Diene **5** 56

1,3-Diene **6** 57

1,3-Diene **7** 58

1,3-Diene **8** 59

1,3-Diene **9** 60

1,3-Diene **10** 61

1,3-Diene **11a** 62

Scale-up synthesis 1,3-diene **11a** 63

1,3-Diene **11b** 64

1,3-Diene **11c** 65

1,3-Diene (±)-**12** 65

1,3-Diene (±)-**13** 66

1,3-Diene **14** 67

1,3-Diene **15** 68

1,3-Diene **16** 69
1,3-Diene **17** 70
1,3-Diene **18a** 70
1,3-Diene **18b** 71
1,3-Diene (±)-**18c** 72
1,3-Diene **18d** 73
1,3-Diene 21a and allylic amine **21a** 74
Allylic amine **21c** 76

SPECTROSCOPIC DATA 77

^{1}H NMR of (±)-**CP-1** 77
^{13}C NMR of (±)-**CP-1** 78
^{1}H NMR of **16-Alk** 79
^{13}C NMR of **16-Alk** 80
^{1}H NMR of **Alk-8** 81
^{13}C NMR of **Alk-8** 82
^{1}H NMR of alkenyl thianthrenium salt **TT-2** 83
^{19}F NMR of alkenyl thianthrenium salt **TT-2** 84
^{13}C NMR of alkenyl thianthrenium salt **TT-2** 85
^{1}H NMR of alkenyl thianthrenium salt **TT-7** 86
^{19}F NMR of alkenyl thianthrenium salt **TT-7** 87
^{13}C NMR of alkenyl thianthrenium salt **TT-7** 88
^{1}H NMR of alkenyl thianthrenium salt **TT-8** 89
^{19}F NMR of alkenyl thianthrenium salt **TT-8** 90
^{13}C NMR of alkenyl thianthrenium salt **TT-8** 91
^{1}H NMR of alkenyl thianthrenium salt (±)-**TT-12** 92
^{19}F NMR of alkenyl thianthrenium salt (±)-**TT-12** 93
^{13}C NMR of alkenyl thianthrenium salt (±)-**TT-12** 94
^{1}H NMR of alkenyl thianthrenium salt TT-**15** 95
^{19}F NMR of alkenyl thianthrenium salt TT-**15** 96
^{13}C NMR of alkenyl thianthrenium salt TT-**15** 97
^{1}H NMR of alkenyl thianthrenium salt TT-**16** 98
^{19}F NMR of alkenyl thianthrenium salt TT-**16** 99
^{13}C NMR of alkenyl thianthrenium salt TT-**16** 100
^{1}H NMR of alkenyl thianthrenium salt TT-**21** 101
^{19}F NMR of alkenyl thianthrenium salt TT-**21** 102
^{13}C NMR of alkenyl thianthrenium salt TT-**21** 103
^{1}H NMR of alkenyl thianthrenium salt TT-**22** 104

^{19}F NMR of alkenyl thianthrenium salt **TT-22** 105
^{13}C NMR of alkenyl thianthrenium salt **TT-22** 106
^{1}H NMR of alkenyl thianthrenium salt **TT-23** 107
^{19}F NMR of alkenyl thianthrenium salt **TT-23** 108
^{13}C NMR of alkenyl thianthrenium salt **TT-23** 109
^{1}H NMR of 1,3-diene **1a** 110
^{13}C NMR of 1,3-diene **1a** 111
^{1}H NMR of 1,3-diene **1c** 112
^{13}C NMR of 1,3-diene **1c** 113
^{1}H NMR of 1,3-diene **2** 114
^{13}C NMR of 1,3-diene **2** 115
^{1}H NMR of 1,3-diene **3** 116
^{13}C NMR of 1,3-diene **3** 117
^{1}H NMR of 1,3-diene **4** 118
^{13}C NMR of 1,3-diene **4** 119
^{19}F NMR of 1,3-diene **4** 120
^{1}H NMR of 1,3-diene (±)-**5** 121
^{13}C NMR of 1,3-diene (±)-**5** 122
^{1}H NMR of 1,3-diene **6** 123
^{13}C NMR of 1,3-diene **6** 124
^{1}H NMR of 1,3-diene **7** 125
^{13}C NMR of 1,3-diene **7** 126
^{19}F NMR of 1,3-diene **7** 127
^{1}H NMR of 1,3-diene **8** 128
^{13}C NMR of 1,3-diene **8** 129
^{1}H NMR of 1,3-diene **9** 130
^{13}C NMR of 1,3-diene **9** 131
^{1}H NMR of 1,3-diene **10** 132
^{13}C NMR of 1,3-diene **10** 133
^{1}H NMR of 1,3-diene **11a** 134
^{13}C NMR of 1,3-diene **11a** 135
^{19}F NMR of 1,3-diene **11a** 136
^{1}H NMR of 1,3-diene **11b** 137
^{13}C NMR of 1,3-diene **11b** 138
^{19}F NMR of 1,3-diene **11b** 139
^{1}H NMR of 1,3-diene **11c** 140
^{13}C NMR of 1,3-diene **11c** 141
^{1}H NMR of 1,3-diene (±)-**12** 142

^{13}C NMR of 1,3-diene (±)-**12** 143
^{1}H NMR of 1,3-diene (±)-**13** 144
^{13}C NMR of 1,3-diene (±)-**13** 145
^{1}H NMR of 1,3-diene **14** 146
^{13}C NMR of 1,3-diene **14** 147
^{1}H NMR of 1,3-diene **15** 148
^{13}C NMR of 1,3-diene **15** 149
^{1}H NMR of 1,3-diene **16** 150
^{13}C NMR of 1,3-diene **16** 151
^{1}H NMR of 1,3-diene **17** 152
^{13}C NMR of 1,3-diene **17** 153
^{1}H NMR of 1,3-diene **18a** 154
^{13}C NMR of 1,3-diene **18a** 155
^{1}H NMR of 1,3-diene **18b** 156
^{13}C NMR of 1,3-diene **18b** 157
^{1}H NMR of 1,3-diene (±)-**18c** 158
^{13}C NMR of 1,3-diene (±)-**18c** 159
^{1}H NMR of 1,3-diene **18d** 160
^{13}C NMR of 1,3-diene **18d** 161
^{1}H NMR of 1,3-diene **21a** 162
^{13}C NMR of 1,3-diene **21a** 163
^{1}H NMR of allylic amine **21b** 164
^{13}C NMR of allylic amine **21b** 165
^{1}H NMR of allylic amine **21c** 166
^{13}C NMR of allylic amine **21c** 167

REFERENCES 168

MATERIALS AND METHODS

All reactions were carried out under a nitrogen atmosphere and monitored by thin-layer chromatography (TLC) or liquid chromatography-mass spectrometry (LC-MS) unless otherwise stated. High-resolution mass spectra were obtained using *Q Exactive Plus* from *Thermo*. Concentration under reduced pressure was performed by a Biotage V10 evaporator. Purified compounds were further dried under vacuum (10^{-6} – 10^{-3} bar). Yields refer to purified and spectroscopically pure compounds, unless otherwise stated.

Solvents

Anhydrous solvents were obtained from Phoenix Solvent Drying Systems. All deuterated solvents were purchased from Euriso-Top®.

Chromatography

Thin layer chromatography (TLC) was performed using EMD TLC plates pre-coated with 250 μm thickness silica gel 60 F_{254} plates and visualized by fluorescence quenching under UV light. Flash column chromatography was performed using silica gel (40 – 63 μm particle size) purchased from Geduran®.

Spectroscopy and Instruments

NMR spectra were recorded on a *Bruker AscendTM* 500 spectrometer, a *Bruker AVANCE Neo* 600 MHz equipped with a BBO cryoprobe, or a *Bruker AVANCE III* 600 MHz equipped with a TCI cryoprobe. Chemical shifts are reported in ppm with the solvent residual peak as the internal standard. For ^{1}H NMR: $CDCl_3$, δ 7.26, CD_3CN, δ 1.96. For ^{13}C NMR: $CDCl_3$, δ 77.16, CD_3CN, δ 1.79.[1] ^{19}F NMR spectra were referenced using a unified chemical shift scale based on the ^{1}H resonance of tetramethylsilane (1% (v/v) solution in the respective solvent). Data is reported as follows: s = singlet, d = doublet, t = triplet, q = quartet, m = multiplet, br = broad; coupling constants in Hz. Liquid-chromatography-mass spectrometry (LC-MS) was performed with an Agilent Technology 1260 Infinity HPLC system coupled to an Agilent Technologies 6120 Quadrupole mass analyzer.

Starting materials

All substrates were used as received from commercial suppliers, unless otherwise stated. Chemicals were purchased from *Sigma-Aldrich*, *TCI*, *Alfa Aesar, Abcr, or BLDpharm*.

Safety statement

The procedures provided here are meant to be carried out exclusively by trained individuals that are aware of the dangers associated with hazardous substances. General guidelines on the use and disposal of dangerous substances can be found in “Prudent Practices in the Laboratory” (Chapters 4, 6, 7, 8).[2] Regarding the proper handling of flammable gases like propadiene/allene, the reader is referred to Chapter 4.D.[2] Hazards associated with specific chemicals and reaction set-ups are provided as “Caution:” in the respective experimental procedure. There may be more hazards associated with the individual procedures

than those that are explicitly mentioned. The absence of a caution note does not mean that there is no danger associated with the chemicals used in that procedure.

EXPERIMENTAL DATA

General procedure for 1,3-diene syntheses from alkenes via alkenyl thianthrenium salts

General procedure for the thianthrenation of monosubstituted, 1,2-disubstituted and trisubstituted olefins

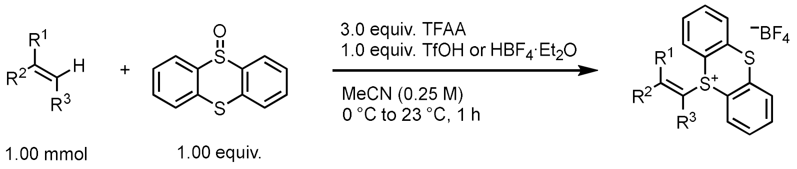

Under an ambient atmosphere, a 20 mL glass vial with a Teflon-coated magnetic stirring bar was charged with alkene (1.00 mmol, 1.0 equiv.), thianthrene-S-oxide (0.23 g, 1.00 mmol, 1.0 equiv.) and MeCN (4.0 mL, c = 0.25 M). The mixture was cooled to 0 °C using an ice bath. Under stirring, trifluoroacetic anhydride (0.42 mL, 0.63 g, 3.0 mmol, 3.0 equiv.) was added dropwise (2 drops per second) leading to a dark purple solution. Trifluoromethanesulfonic acid (0.11 mL, 0.18 g, 1.2 mmol, 1.2 eq.) was added dropwise (2 drops per second), and the mixture was stirred at 0 °C for 45 min followed by stirring at 25 °C for 15 min. The resulting mixture was diluted with DCM (5 mL) and was poured onto a saturated aqueous $NaHCO_3$ solution (10 mL). The layers were separated and the aqueous layer was further extracted with DCM (2 × 5 mL). The unified organic layer was washed with aqueous $NaBF_4$ solution (2 × 20 mL, 10% w/w). The organic layer was dried with Na_2SO_4, filtered, and the solvent was removed under reduced pressure. The residue was purified by chromatography on silica gel eluting with pentane/EtOAc (4:1, v/v) → DCM/MeOH (50:1, v/v) to afford the analytically pure alkenyl thianthrenium salt.

General procedure for 1,3-diene synthesis from alkenyl thianthrenium salts

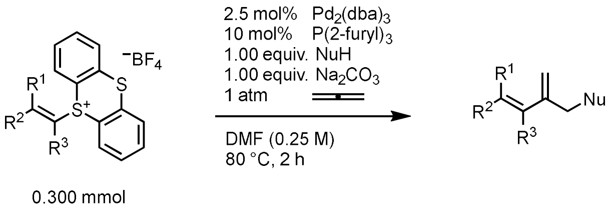

Caution: Propadiene/allene is a flammable gas and should be handled with appropriate care. Guidelines regarding the safe handling of flammable gases can be found in “Prudent Practices in the Laboratory” (Chapter 4.D).[2]

All manipulations were done under an ambient atmosphere in a fume hood (image **A**). A 4 mL borosilicate vial equipped with a Teflon-coated magnetic stirring bar was charged with $Pd_2(dba)_3$ (6.9 mg, 7.5 µmol, 2.5 mol%), tri(2-furyl)phosphine (7.0 mg, 30 µmol, 10 mol%), alkenyl thianthrenium salt (0.300 mmol, 1.0 equiv.), Na_2CO_3 (31.8 mg, 0.300 mmol, 1.0 equiv.) and nucleophile (0.300 mmol, 1.0 equiv.). Dry DMF (1.2 mL, c = 0.25 M) was added and the vial was sealed with a septum cap. The septum was pierced with an outlet needle

(Φ 0.80 × 40 mm) (image **B**) and another needle (Φ 0.80 × 120 mm), which was connected to an allene-containing balloon. A gentle stream of allene gas was passed through the mixture for 2 min (image **C**). Both needles were removed and the septum cap was quickly wrapped with parafilm (image **D**). The vial was transferred to a heating block, which had been preheated at 80 °C, and the reaction mixture was stirred at 1000 rpm for 2 h (image **E**). Then, the vial was removed from the heating block, the stirring bar was removed, and the solvent was evaporated using a Biotage V10. The obtained residues were purified by column chromatography on silica gel.

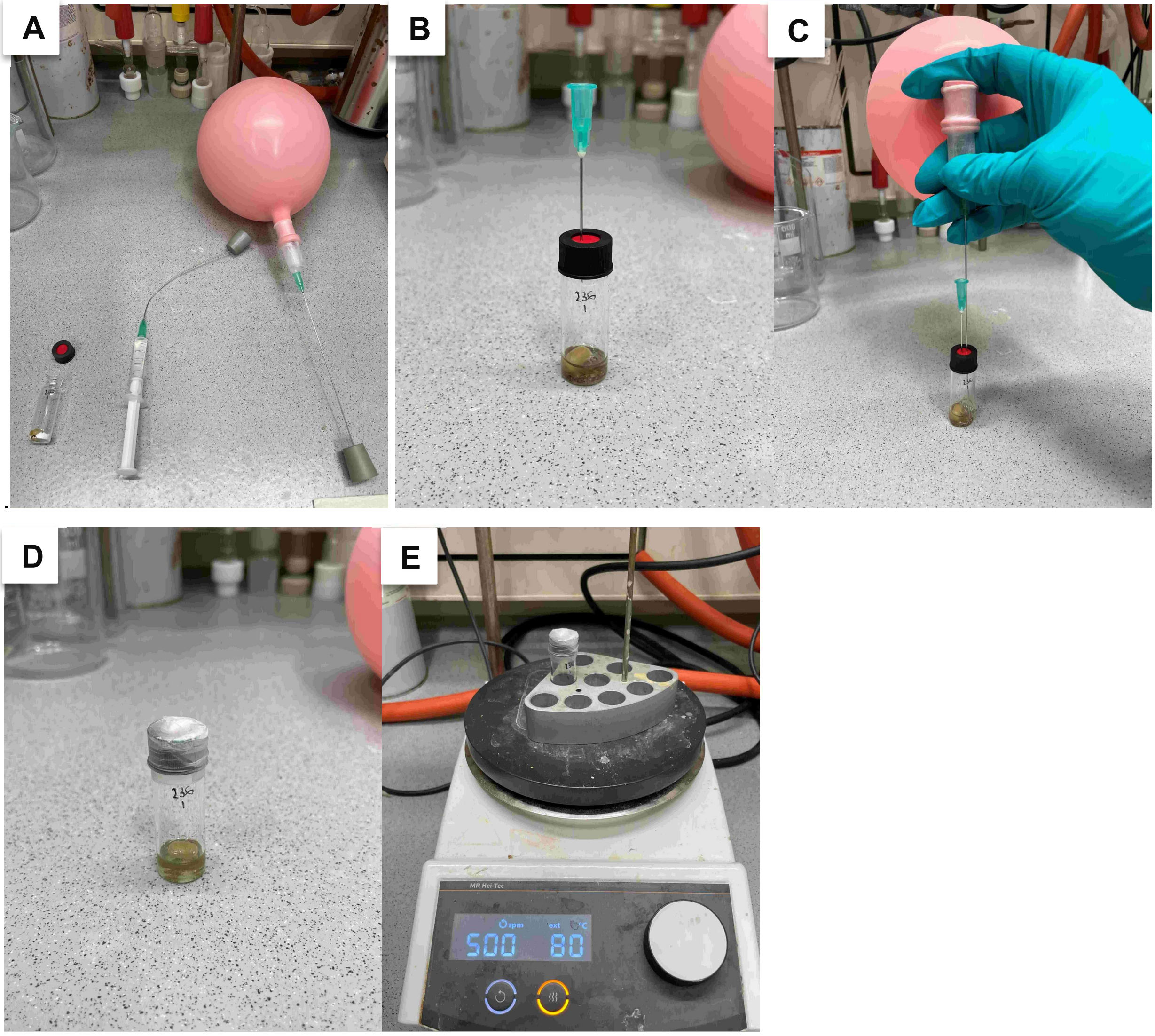

Optimization of reaction conditions

General procedure

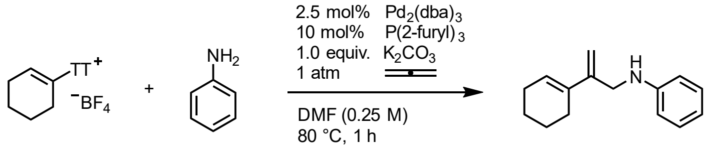

TT-1, 0.100 mmol 1.0 equiv. **1a**, 68%

Caution: Propadiene/allene is a flammable gas and should be handled with appropriate care. Guidelines regarding the safe handling of flammable gases can be found in "Prudent Practices in the Laboratory" (Chapter 4.D).[2]

A 4 mL borosilicate vial equipped with a Teflon-coated magnetic stirring bar was charged with $Pd_2(dba)_3$ (2.3 mg, 2.5 µmol, 2.5 mol%), tri(2-furyl)phosphine (2.3 mg, 10 µmol, 10 mol%), alkenyl thianthrenium salt **TT-1** (38.4 mg, 0.100 mmol, 1.0 equiv.), K_2CO_3 (13.8 mg, 0.100 mmol, 1.0 equiv.) and aniline (9.3 mg, 9.1 µL, 0.10 mmol, 1.0 equiv.). Dry DMF (0.4 mL, c = 0.25 M) was added and the vial was sealed with a septum cap. The septum was pierced with an outlet needle (Φ 0.80 × 40 mm) and another needle (Φ 0.80 × 120 mm), which was connected to an allene-containing balloon. A gentle stream of allene gas was passed through the mixture for 1 min. Both needles were removed and the septum cap was quickly wrapped with parafilm. The vial was transferred to a heating block, which had been preheated at 80 °C, and the reaction mixture was stirred at 500 rpm at 80 °C for 1 h. Then, the vial was removed from the heating block, the stirring bar was removed, and the solvent evaporated using a Biotage V10. Trichloroethylene (13.1 mg, 0.100 mmol, 1.00 equiv.) was added, and the exact weight added was noted. $CDCl_3$ (0.5 mL) was added, and the resulting mixture was vigorously shaken for 30 s. Then, an aliquot of the mixture was taken and passed into an NMR tube through a Pasteur pipette that had been fitted with a piece of cotton to remove insoluble solids. More $CDCl_3$ was passed through the same Pasteur pipette into the NMR tube until the NMR tube was filled with a volume of at least 0.5 mL. Then, a ^1H NMR spectrum (NS = 16, D1 = 1 s, SW = 19.9875 ppm, O1P = 6.175 ppm, TD = 65536, O2 = 3089.62 Hz, O2P = 6.175 ppm) was measured, and the yield of **1a** was determined by comparing the relative integrals of trichloroethylene's proton signal (δ = 6.46 ppm, s, 1H) and one of **1a**'s olefinic proton signals (δ = 5.97 ppm, t, J = 4.2 Hz, 1H).

All following experiments during reaction optimization were carried out analogously.

1,3-Diene product 1a

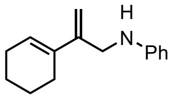

1a

R*f* = 0.55 (pentane/EtOAc = 19:1 (v/v), $KMnO_4$).

NMR Spectroscopy:

^{1}H NMR (500 MHz, $CDCl_3$) δ 7.23 – 7.13 (m, 2H), 6.71 (td, *J* = 7.3, 1.2 Hz, 1H), 6.65 – 6.56 (m, 2H), 5.97 (t, *J* = 4.2 Hz, 1H), 5.12 (d, *J* = 14.8 Hz, 2H), 3.94 (s, 2H), 3.80 (br s, 1H), 2.28 – 2.20 (m, 2H), 2.20 – 2.13 (m, 2H), 1.72 (qd, *J* = 7.7, 3.8 Hz, 2H), 1.68 – 1.56 (m, 2H).

^{13}C NMR (126 MHz, $CDCl_3$) δ 148.4, 144.8, 134.8, 129.3, 125.2, 117.3, 112.8, 110.6, 47.1, 26.1, 26.0, 22.9, 22.3.

HRMS-EI (m/z) calc'd for $C_{15}H_{19}N_1$ $[M]^+$, 213.1512; found, 213.1513; deviation: –0.3 ppm.

The obtained data are consistent with those previously reported in the literature.[3]

Allylic amine side product (±)-1b

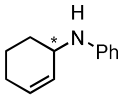

(±)-**1b**

R*f* = 0.29 (pentane/EtOAc = 9:1 (v/v), $KMnO_4$).

NMR Spectroscopy:

^{1}H NMR (500 MHz, $CDCl_3$) δ 7.22 – 7.14 (m, 2H), 6.69 (t, *J* = 7.3 Hz, 1H), 6.66 – 6.59 (m, 2H), 5.89 – 5.82 (m, 1H), 5.80 – 5.73 (m, 1H), 4.08 – 3.94 (m, 1H), 3.63 (s, 1H), 2.12 – 1.98 (m, 2H), 1.97 – 1.86 (m, 1H), 1.78 – 1.69 (m, 1H), 1.69 – 1.60 (m, 2H).

^{13}C NMR (126 MHz, $CDCl_3$) δ 147.3, 130.2, 129.4, 128.7, 117.2, 113.3, 48.0, 29.0, 25.3, 19.8.

HRMS-EI (m/z) calc'd for $C_{12}H_{15}N_1$ $[M]^+$, 173.1199; found, 173.1201; deviation: –1.4 ppm.

The obtained data are consistent with those previously reported in the literature.[4]

Solvent water content

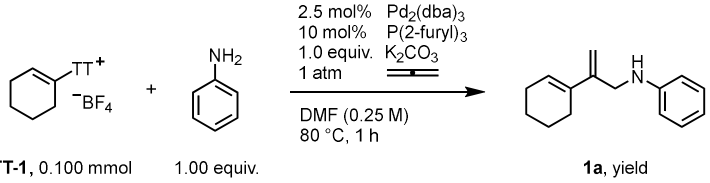

Table S1. Effect of water content of the solvent on the reaction.

Entry	Water content / ppm	Yield / %

**1** [a]	736 ± 169	27
**2** [a]	52 ± 6	73
**3** [b]	52 ± 4	71

[a] Reaction was set up in a fume hood using Schlenk techniques either using non-dried DMF or DMF that had been dried over molecular sieves. [b] Reaction was set up inside glovebox using DMF from an acroseal bottle. Water contents were determined via Karl-Fischer titration.

Reaction setup

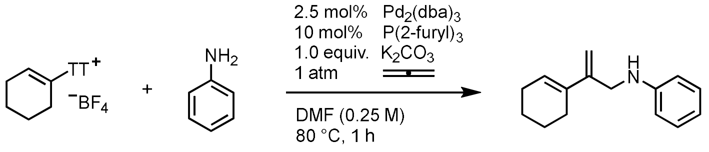

TT-1, 0.100 mmol   1.0 equiv.   **1a**, yield

Table S2. Comparison of reactions setup in the glovebox and in the fumehood.

Entry	Set-up	Yield / %
**1**	fumehood[a]	71
**2**	glovebox[b]	71

[a] All solids were weighed in, dry DMF from the glovebox was added under an ambient atmosphere, aniline was added, and the reaction was degassed using allene gas. [b] All compounds were weighed in, the vial was transferred inside the glovebox, and dry DMF and aniline were added. Then, the vial was transferred outside the glovebox and degassed using allene gas.

Temperature-dependence

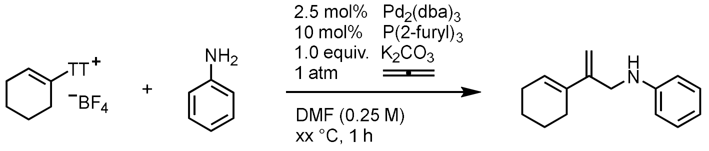

TT-1, 0.100 mmol   1.0 equiv.   **1a**, yield

Table S3. Reaction temperature dependence investigation.

Entry	Temperature / °C	Yield / %
**1**	23	22
**2**	40	35

**4**	60	65
**5**	70	83
**6**	80	81
**7**	90	57

Palladium loading

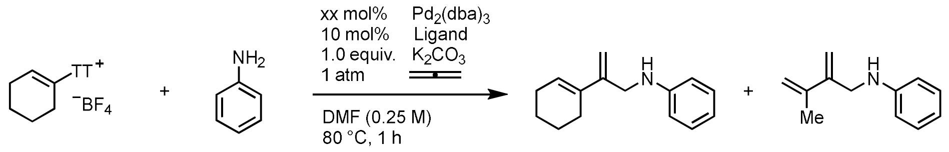

TT-1, 0.100 mmol 1.00 equiv. **1a**, yield **1c**, yield

Table S4. Screening of different palladium loadings.

**Entry**	**Palladium loading / mol%**	**Yield / %**	
		**1a**	**1c**
**1**	1.3	82	5
**2**	2.5	71	5
**3**	5.0	46	16
**4**	10	68	7

Base screening

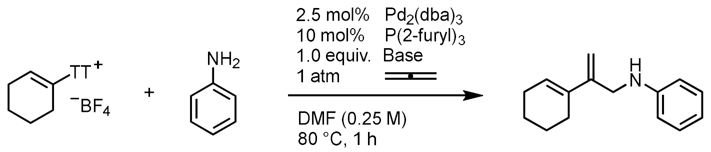

TT-1, 0.100 mmol 1.0 equiv. **1a**, yield

Table S5. Screening of different bases.

**Entry**	**Base**	**Yield / %**
**1**	K_2CO_3	71
**2**	$NaHCO_3$	78

**3**	Na_2SO_3	68
**4**	Na_2SO_4	n.d.
**5**	K_3PO_4	73
**6**	Na_2CO_3	83

Reproducibility screening

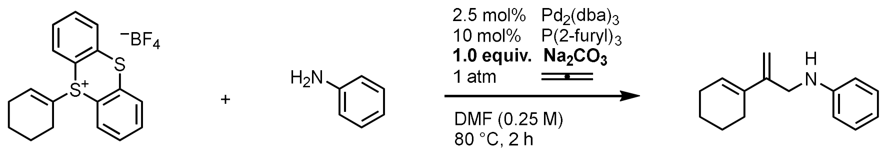

TT-1, 0.100 mmol 1.0 equiv. **1a**, yield

Average Yield: 73 ± 4%

Table S6. Reproducibility screening with Na_2CO_3.

**Entry**	**Yield / %**
**1**	74
**2**	77
**3**	71
**4**	75
**5**	68

Average yield: 73±4%

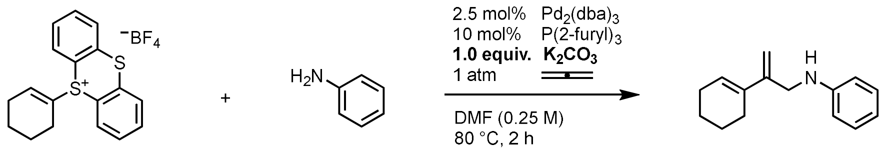

TT-1, 0.100 mmol 1.0 equiv. **1a**, yield

Average Yield: 74 ± 8%

Table S7. Reproducibility screening with K_2CO_3.

**Entry**	**Yield / %**
**1**	61

**2**	74
**3**	81
**4**	75
**5**	80

Average yield: 74±8%

We suspect that, when the reaction is done on 0.100 mmol scale, the inorganic base is the main reason that accounts for fluctuations in yield. During reaction set-up, particles of the inorganic base can inadvertently be spread to the walls of the reaction vessel when propadiene is bubbled through the solution (see image below). On 0.300 mmol scale, when 1.2 mL of DMF is used, most of these particles are eventually washed back into solution. On 0.100 mmol scale with only 0.4 mL of DMF present, the probability of base particles remaining outside the reaction solution is higher. This hypothesis is supported by experiments run at higher dilution (c = 0.1 M, see below).

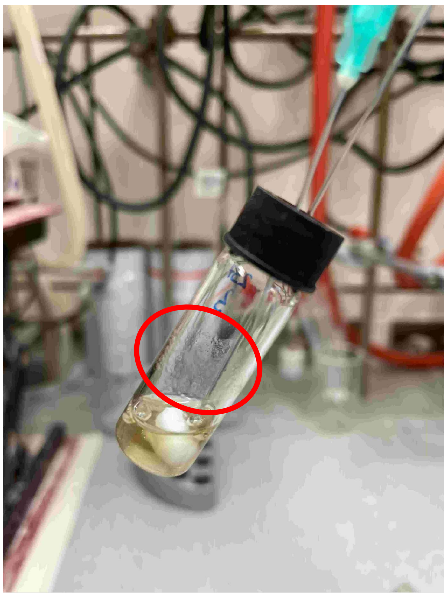

Image F: Representative example of base particles being spread to the vessel walls during introduction of propadiene.

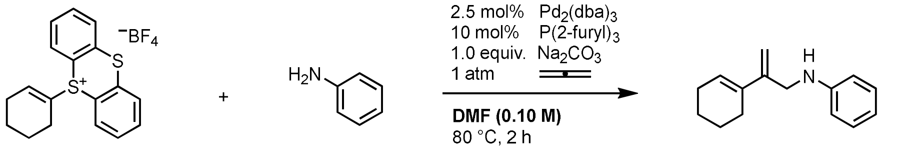

TT-1, 0.100 mmol 1.0 equiv. **1a**, yield

Average Yield: 83 ± 2%

Table S8. Reproducibility screening with Na_2CO_3 under diluted conditions.

Entry	Yield / %
**1**	84
**2**	81
**3**	84

Average yield: 83±2%

Solvent screening

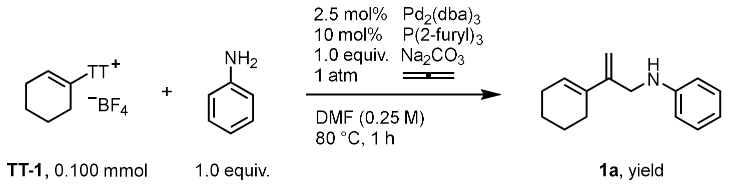

Table S9. Solvent screening.

Entry	Solvent	Yield / %
**1**	DMF	80
**2**	1,4-dioxane	81
**3**	MeCN	10
**4**	DMA	67
**5**	DMSO	51

Ligand screening

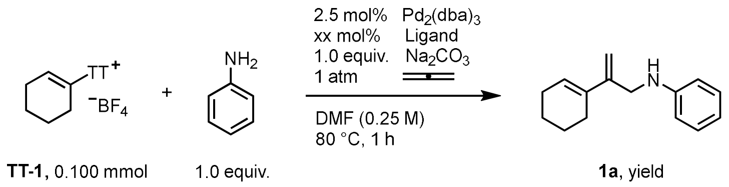

Table S10. Screening of mono- and bidentate phosphine ligands.

Entry	Ligand (mol%)	Yield / %

**1**	**L1** (10)	16
**2**	**L2** (10)	70
**3**	**L3** (10)	28
**4**	**L4** (10)	n.d
**5**	**L5** (10)	n.d.
**6**	**L6** (10)	60
**7**	**L7** (10)	2
**8**	**L8** (10)	n.d.
**11**	**L9** (10)	4
**12**	**L10** (10)	1
**13**	**L11** (10)	n.d.
**14**	**L12** (5)	2
**15**	**L13** (5)	6
**16**	**L14** (5)	n.d.

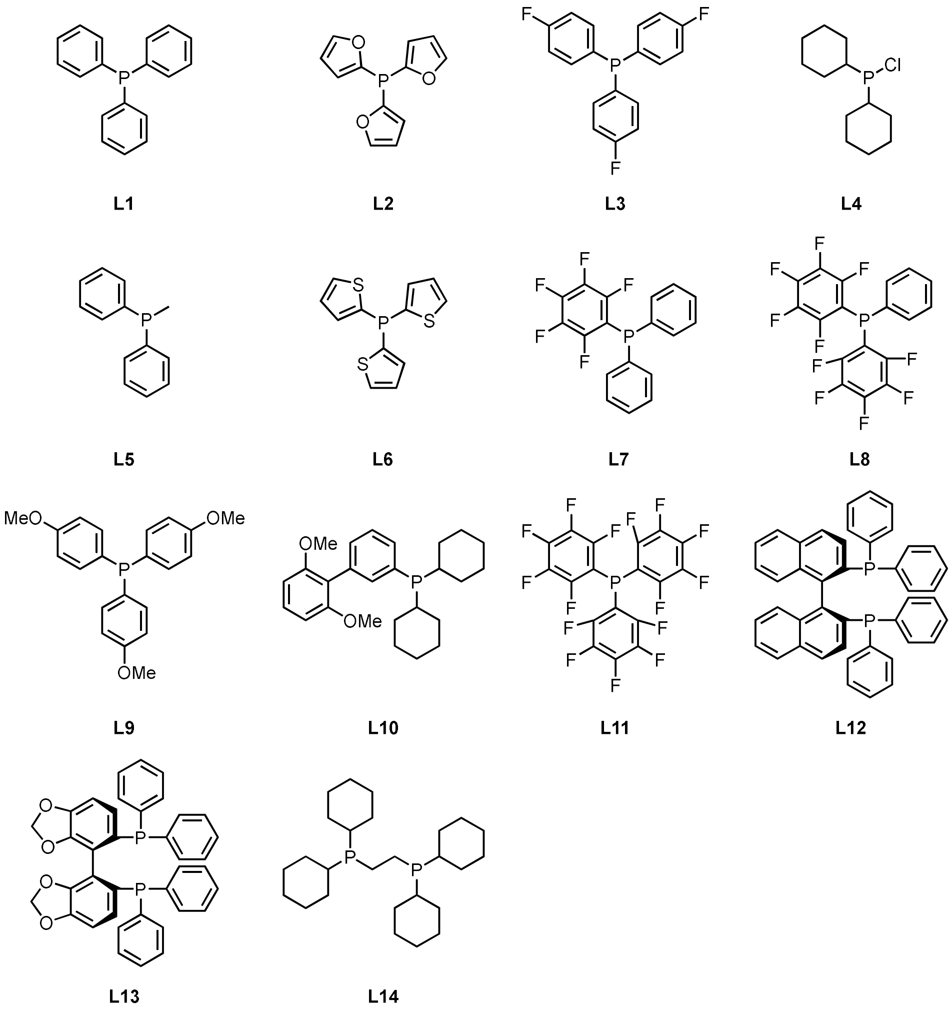

Figure S1. Mono- and bidentate phosphine ligands used in the optimization.

Pd source screening

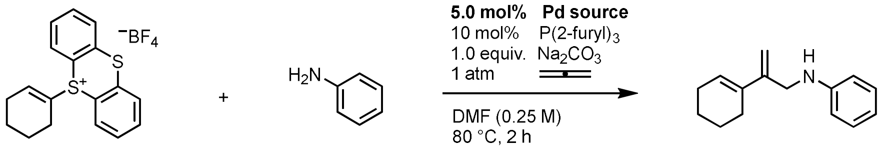

TT-1, 0.100 mmol   1.0 equiv.   **1a**, yield

Table S11. Reproducibility screening with Na_2CO_3.

Entry	Pd source	Yield / %
**1**	$Pd(OAc)_2$	38

**2**	$PdCl_2$	56
**3**	$Pd(acac)_2$	73
**4**	$Pd(PPh_3)_4$	75

Allene-dimerization side product 1c

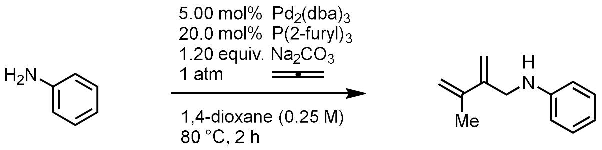

1.50 mmol

1c, 2%

Caution: Propadiene/allene is a flammable gas and should be handled with appropriate care. Guidelines regarding the safe handling of flammable gases can be found in “Prudent Practices in the Laboratory” (Chapter 4.D).[2]

A 4 mL borosilicate vial equipped with a Teflon-coated magnetic stirring bar was charged with $Pd_2(dba)_3$ (13.7 mg, 15.0 µmol, 5.00 mol%), tri(2-furyl)phosphine (13.9 mg, 60.0 µmol, 20.0 mol%), Na_2CO_3 (38.2 mg, 300 µmol, 1.20 equiv.) and aniline (27.3 µL, 27.9 mg, 0.300 mmol, 1.00 equiv.). Anhydrous 1,4-dioxane (1.2 mL, c = 0.25 M) was added, and the vial was sealed with a septum cap. The septum was pierced with an outlet needle (Φ 0.80 × 40 mm) and another needle (Φ 0.80 × 120 mm), which was connected to an allene-containing balloon. A gentle stream of allene gas was passed through the mixture for 2 min. Both needles were removed and the septum cap was quickly wrapped with parafilm. The vial was transferred to a heating block, which had been preheated at 80 °C, and the reaction mixture was stirred at 1000 rpm for 2 h. Then, the vial was removed from the heating block, the stirring bar was removed, and the solvent evaporated using a Biotage V10. Four more reactions were conducted in the same manner, and the respective residues were combined. The resulting residue was purified by column chromatography on silica gel eluting with hexanes/EtOAc (70:1 → 50:1, v/v) to afford 21.6 mg of a mixture of **1c** and tri(2-furyl)phosphine. This mixture was purified further via preparative thin-layer chromatography eluting with hexanes/acetone (15:1, v/v). The silica layer containing **1c** was scraped off and transferred to a fritted vacuum funnel. The silica was washed with EtOAc (10 mL) and filtered. This step was repeated two more times. The resulting filtrate was concentrated to afford **1c** (6.3 mg, 2%) as a yellow oil.

$\mathbf{R}_f$ = 0.36 (hexanes/EtOAc = 50:1 (v/v), $KMnO_4$).

$\mathbf{R}_f$ = 0.61 (hexanes/acetone = 15:1 (v/v), $KMnO_4$).

NMR Spectroscopy:

^{1}H NMR (500 MHz, $CDCl_3$) δ 7.18 (t, J = 7.8 Hz, 2H), 6.70 (t, J = 7.3 Hz, 1H), 6.60 (d, J = 7.9 Hz, 2H), 5.31 – 5.19 (m, 2H), 5.13 (s, 1H), 5.06 (s, 1H), 3.97 (s, 2H), 1.97 (s, 3H).

^{13}C NMR (126 MHz, $CDCl_3$) δ 148.3, 144.2, 141.6, 129.3, 117.5, 113.8, 113.1, 112.9, 46.9, 21.4.

HRMS-ESI (m/z) calc'd for $C_{12}H_{15}N_2$ $[M]^+$, 173.1198; found, 173.1200; deviation: –0.4 ppm.

NMR spiking experiments

The following experiment was carried out to confirm the presence of side product **1c**:

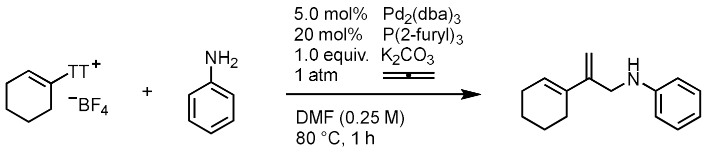

TT-1, 0.100 mmol 1.0 equiv. **1a**

Caution: Propadiene/allene is a flammable gas and should be handled with appropriate care. Guidelines regarding the safe handling of flammable gases can be found in "Prudent Practices in the Laboratory" (Chapter 4.D).[2]

A 4 mL borosilicate vial equipped with a Teflon-coated magnetic stirring bar was charged with $Pd_2(dba)_3$ (4.6 mg, 5.0 µmol, 5.0 mol%), tri(2-furyl)phosphine (4.6 mg, 20 µmol, 20 mol%) alkenyl thianthrenium salt **TT-1** (38.4 mg, 0.100 mmol, 1.0 equiv.), K_2CO_3 (10.6 mg, 0.100 mmol, 1.0 equiv.) and aniline (9.3 mg, 9.1 µL, 0.10 mmol, 1.0 equiv.). Dry DMF (0.4 mL, c = 0.25 M) was added and the vial was sealed with a septum cap. The septum was pierced with an outlet needle (Φ 0.80 × 40 mm) and another needle (Φ 0.80 × 120 mm), which was connected to an allene-containing balloon. A gentle stream of allene gas was passed through the mixture for 1 min. Both needles were removed and the septum cap was quickly wrapped with parafilm. The vial was transferred to a heating block, which had been preheated at 80 °C, and the reaction mixture was stirred at 500 rpm at 80 °C for 1 h. Then, the vial was removed from the heating block, the stirring bar was removed, and the solvent evaporated using a Biotage V10. $CDCl_3$ (0.5 mL) was added, and the resulting mixture was vigorously shaken for 30 s. Then, an aliquot of the mixture was taken and passed into an NMR tube through a Pasteur pipette that had been fitted with a piece of cotton to remove insoluble solids. More $CDCl_3$ was passed through the same Pasteur pipette into the NMR tube until the NMR tube was filled with a volume of at least 0.5 mL. Then, the ^{1}H NMR spectrum depicted below as measured (NS = 16, D1 = 1 s, SW = 19.9875 ppm, O1P = 6.175 ppm, TD = 65536, O2 = 3089.62 Hz, O2P = 6.175 ppm):

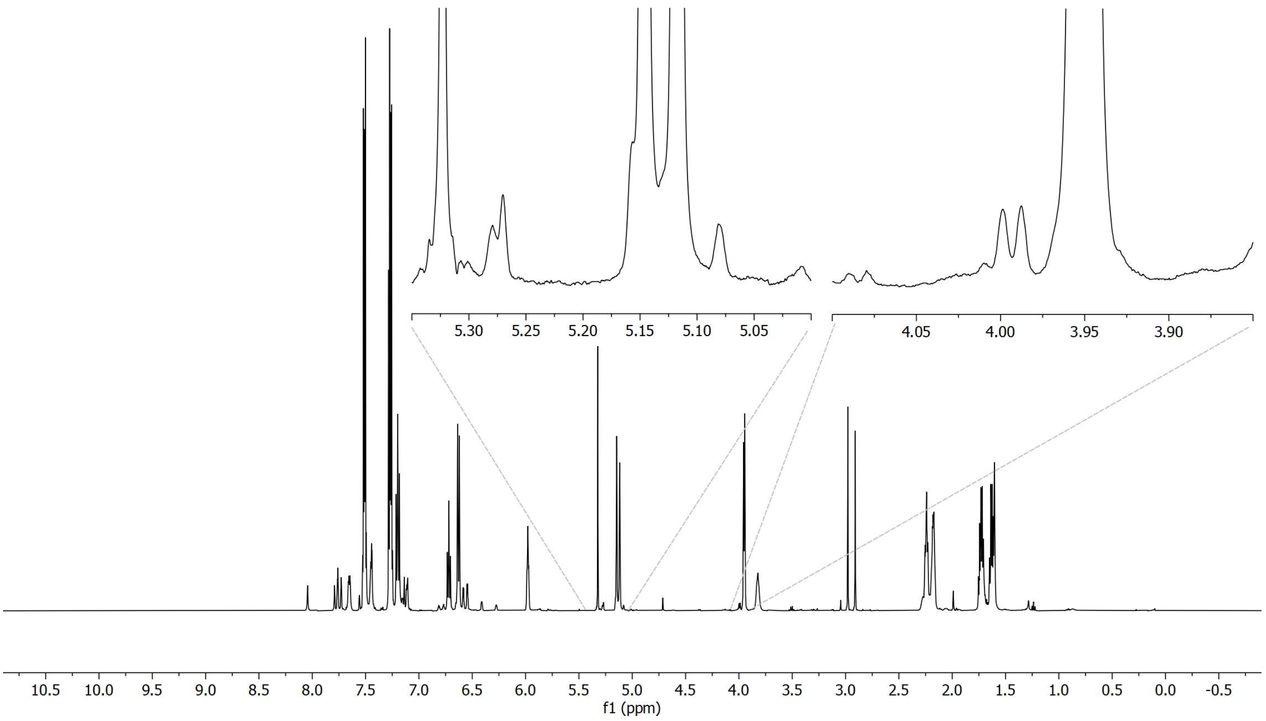

Figure S2: ^{1}H NMR spectrum of the crude reaction mixture before spiking with authentic **1c**.

Then, the authentic sample of **1c** was dissolved in $CDCl_3$ (0.5 mL), an aliquot was taken, and the aliquot was added to the NMR tube of the previously measured sample. Another ^{1}H NMR spectrum was measured with the same settings:

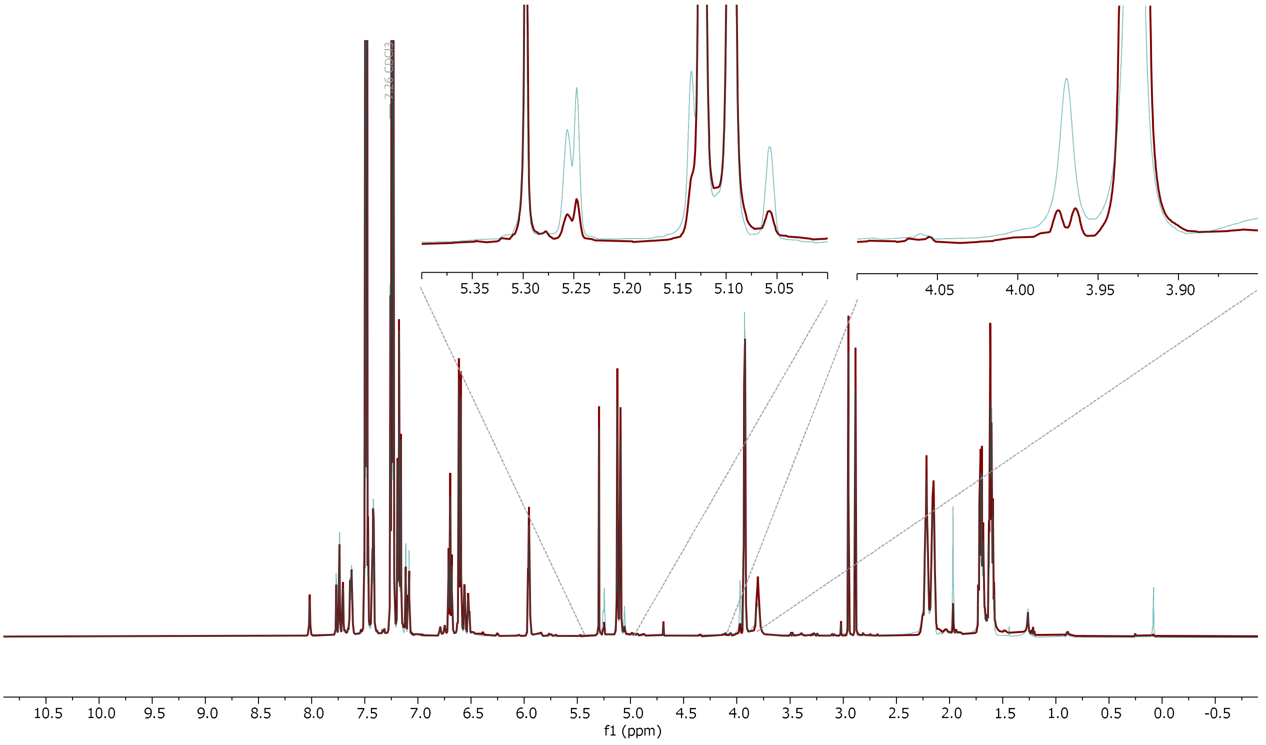

Figure S3: red: ^{1}H NMR spectrum of the crude reaction mixture before spiking with authentic **1c**, green: ^{1}H NMR spectrum of the crude reaction mixture after spiking with authentic **1c**.

The obtained spectra were interpreted to mean that **1c** was present in the crude reaction mixture before the spiking experiment.

Influence of pressure

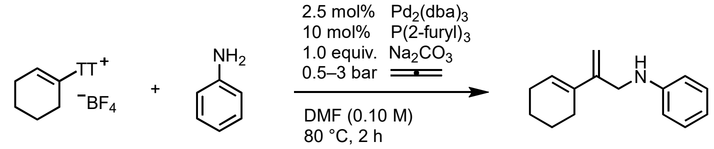

TT-1, 0.300 mmol   1.0 equiv.   **1a**, yield

Caution: Propadiene/allene is a flammable gas and should be handled with appropriate care. Guidelines regarding the safe handling of flammable gases can be found in "Prudent Practices in the Laboratory" (Chapter 4.D).[2]

A 40 mL Fisher-Porter tube equipped with a Teflon-coated magnetic stirring bar was charged with $Pd_2(dba)_3$ (6.9 mg, 7.5 µmol, 2.5 mol%), tri(2-furyl)phosphine (7.0 mg, 10 µmol, 10 mol%), alkenyl thianthrenium salt **TT-1** (115 mg, 0.300 mmol, 1.0 equiv.), Na_2CO_3 (31.8 mg, 0.300 mmol, 1.0 equiv.). The Fisher-Porter tube was introduced into a nitrogen-filled glove box. Aniline (28 mg, 27 µL, 0.10 mmol, 1.0 equiv.) and dry DMF (3.0 mL, c = 0.10 M) were added, and the tube was sealed with a manometer. The Fisher-Porter tube was removed from the glovebox and connected to a propadiene-containing gas cylinder. The tube was charged with the respective pressure of propadiene (0.5 bar, 1 bar, 3 bar) and the valve connecting the tube to the gas cylinder was closed. The solution was stirred for 1 min at 23 °C, leading to a drop in pressure due to propadiene going into solution. The same process was repeated until a constant pressure was reached (see image G, H, I below). Upon reaching constant pressure, the Fisher-Porter tube was placed in an oil bath, which had been preheated at 80 °C. A blast shield was placed in front of the reaction vessel and the reaction mixture was stirred at 500 rpm at 80 °C for 2 h. Then, the tube was removed from the oil bath and allowed to reach ambient temperature. The pressure valve was opened, the stirring bar was removed, and the mixture was transferred to a 20 mL glass vial. The solvent was evaporated using a Biotage V10. Trichloroethylene (39.3 mg, 0.300 mmol, 1.00 equiv.) was added, and the exact weight added was noted. $CDCl_3$ (1.0 mL) was added, and the resulting mixture was vigorously shaken for 30 s. Then, an aliquot of the mixture was taken and passed into an NMR tube through a Pasteur pipette that had been fitted with a piece of cotton to remove insoluble solids. More $CDCl_3$ was passed through the same Pasteur pipette into the NMR tube until the NMR tube was filled with a volume of at least 0.5 mL. Then, a ^1H NMR spectrum (NS = 16, D1 = 1 s, SW = 19.9875 ppm, O1P = 6.175 ppm, TD = 65536, O2 = 3089.62 Hz, O2P = 6.175 ppm) was measured, and the yield of **1a** was determined by comparing the relative integrals of trichloroethylene's proton signal (δ = 6.46 ppm, s, 1H) and one of **1a**'s olefinic proton signals (δ = 5.97 ppm, t, *J* = 4.2 Hz, 1H).

Table S12. Pressure screening.

Entry	Pressure / bar	Yield / %
**1**	0.5	53
**2**	1	89
**3**	3	60

Mechanistic experiments

Michael addition reversibility

This section describes experiments that were conducted based on observations made during the synthesis of 1,3-diene **16**. It was observed that **TT-16**, a mixture of the *E*-isomer ***E*-TT-16** and the *Z*-isomer ***Z*-TT-16**, was converted to the pure *E*-isomer of 1,3-diene **16**:

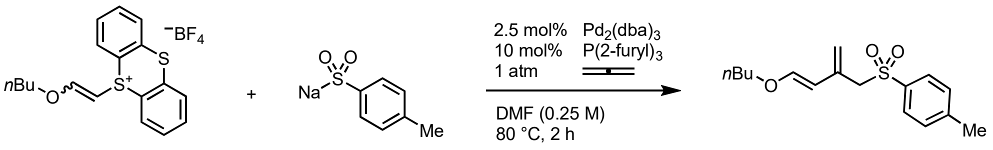

TT-16, 0.300 mmol (*E/Z* = 1:1.6) 1.0 equiv. **16**, 71%

To investigate whether a change of the ration between ***E*-TT-16** and ***Z*-TT-16** could be observed in the absence of $Pd_2(dba)_3$, the following experiment was conducted:

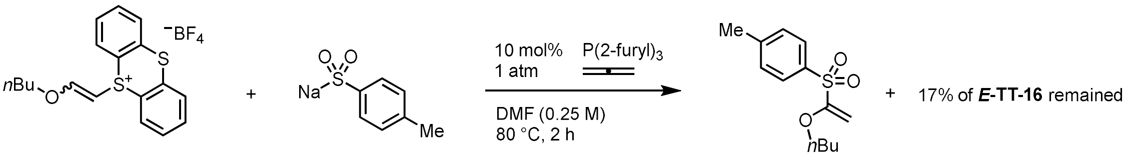

TT-16, 0.200 mmol (*E/Z* = 1:1) 1.0 equiv. **16-Alk**, 21%

Caution: Propadiene/allene is a flammable gas and should be handled with appropriate care. Guidelines regarding the safe handling of flammable gases can be found in "Prudent Practices in the Laboratory" (Chapter 4.D).[2]

A 4 mL borosilicate vial equipped with a Teflon-coated magnetic stirring bar was charged with tri(2-furyl)phosphine (4.6 mg, 20 µmol, 10 mol%), alkenyl thianthrenium salt **TT-16** (80.5 mg, 0.200 mmol, 1.0 equiv.) and sodium *p*-toluenesulfinate (35.6 mg, 0.200 mmol, 1.0 equiv.). Dry DMF (0.80 mL, c = 0.25 M) was added and the vial was sealed with a septum cap. The septum was pierced with an outlet needle (Φ 0.80 × 40 mm) and another needle (Φ 0.80 × 120 mm), which was connected to an allene-containing balloon. A gentle stream of allene gas was passed through the mixture for 2 min. Both needles were removed and the septum cap was quickly wrapped with parafilm. The vial was transferred to a heating block, which had been preheated at 80 °C, and the reaction mixture was stirred at 500 rpm for 2 h. Then, the vial was removed from the heating block, the stirring bar was removed, and the solvent evaporated using a Biotage V10. Trichloroethylene (27.8 mg, 19.0 µL, 1.06 equiv.) was added as internal standard, $CDCl_3$ (1.0 mL) was added, and the resulting mixture was vigorously shaken for 1 min. Then, an aliquot was taken and submitted to ^1H NMR spectroscopic analysis, resulting in the following spectrum:

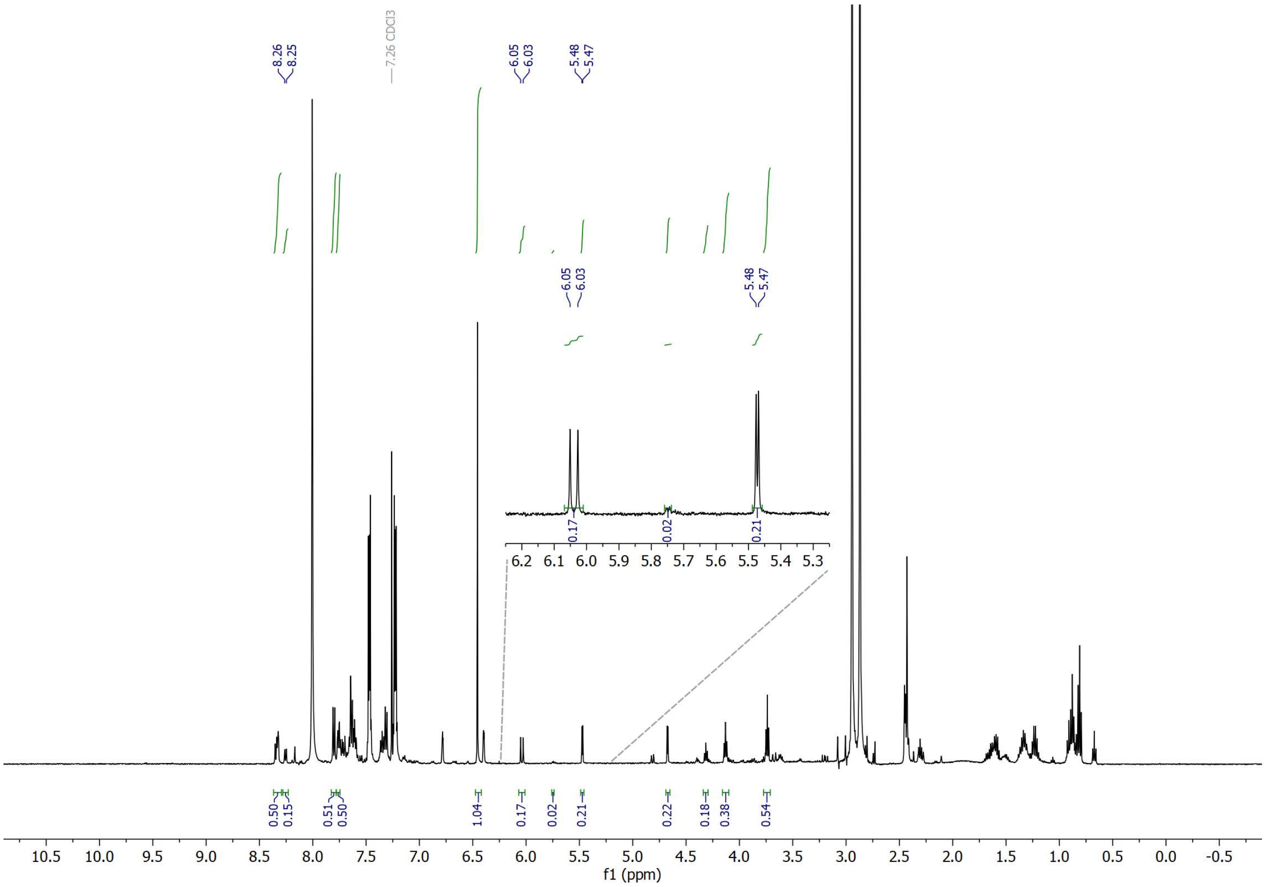

Figure S4: ^{1}H NMR spectrum of the crude reaction mixture before spiking with authentic **TT-16**.

The spectrum was interpreted to mean that all of ***Z*-TT-16** had been consumed, while 17% of ***E*-TT-16** remained. To confirm the presence of ***E*-TT-16**, a small aliquot of a solution of authentic **TT-16** dissolved in $CDCl_3$ was added to the NMR tube and the following ^1H NMR spectrum was obtained:

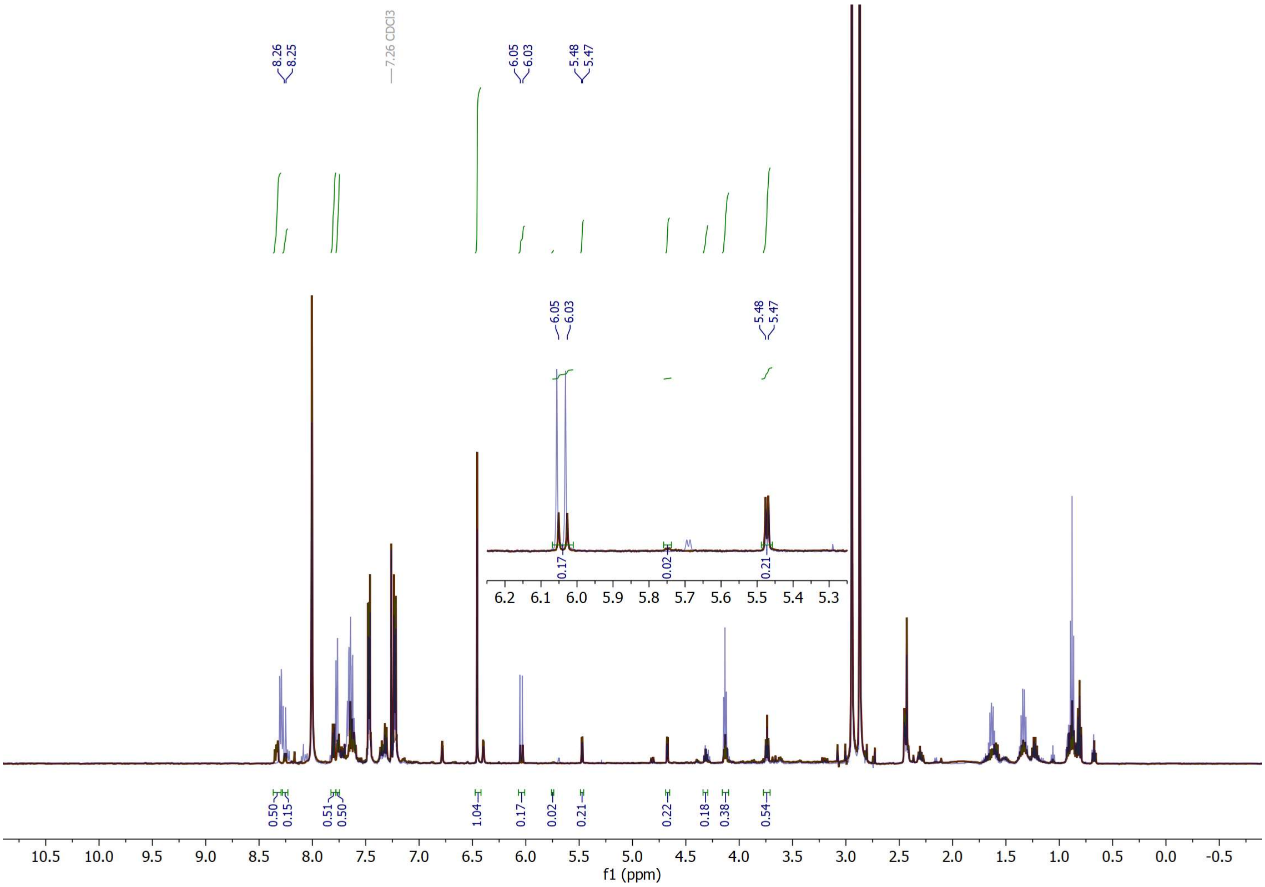

Figure S5: Red: ^{1}H NMR spectrum of the crude reaction mixture before spiking with authentic **TT-16**. Blue: ^{1}H NMR spectrum of the crude reaction mixture after spiking with authentic **TT-16**.

The spectrum was interpreted to mean that ***E*-TT-16** had indeed remained after the reaction. The remaining mixture that had resulted from dissolving the residue in $CDCl_3$ was purified by chromatography on silica gel eluting with hexanes/EtOAc (19:1 → 9:1 → 7:1 → 5:1, v/v) to afford **16-Alk** (10.6 mg, 21%) as a colorless oil.

$\mathbf{R_f}$ = 0.49 (Hex/EtOAc = 5:1 (v/v), $KMnO_4$).

NMR Spectroscopy:

^{1}H NMR (500 MHz, $CDCl_3$) δ 7.81 (d, *J* = 8.1 Hz, 2H), 7.32 (d, *J* = 8.0 Hz, 2H), 5.48 (d, *J* = 3.9 Hz, 1H), 4.68 (d, *J* = 3.9 Hz, 1H), 3.75 (t, *J* = 6.4 Hz, 2H), 2.44 (s, 3H), 1.67 – 1.50 (m, 2H), 1.33 – 1.17 (m, 3H), 0.82 (t, *J* = 7.4 Hz, 3H).

^{13}C NMR (151 MHz, $CDCl_3$) δ 160.4, 144.8, 135.4, 129.7, 128.9, 91.3, 70.7, 30.4, 21.8, 19.0, 13.7.

HRMS-ESI (m/z) calc'd for $C_{13}H_{18}S_1O_3$ $[M+Na]^+$, 277.0869; found, 277.0867; deviation: +0.6 ppm.

The experiment was interpreted to mean that ***Z*-TT-16** is consumed more readily than ***E*-TT-16** under the reaction conditions.

To determine how fast ***Z*-TT-16** is consumed, the same following experiment was executed with three different reaction times (10 min, 20 min, 30 min):

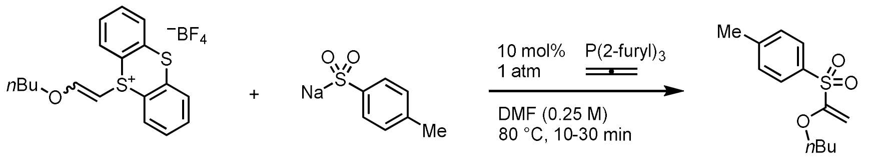

Entry	Time	**A** / %	***E*-TT-2** / %	***Z*-TT-2** / %
1	10 min	14	42	4
2	20 min	18	37	4
3	30 min	21	25	3

A 1 mL GC vial equipped with a Teflon-coated magnetic stirring bar was charged with tri(2-furyl)phosphine (1.2 mg, 5.0 µmol, 10 mol%), alkenyl thianthrenium salt **TT-16** (20.0 mg, 50.0 µmol, 1.0 equiv.) and sodium *p*-toluenesulfinate (8.9 mg, 50 µmol, 1.0 equiv.). Dry DMF (0.20 mL, c = 0.25 M) was added and the vial was sealed with a septum cap. The septum was pierced with an outlet needle (Φ 0.80 × 40 mm) and another needle (Φ 0.80 × 120 mm), which was connected to an allene-containing balloon. A gentle stream of allene gas was passed through the mixture for 1 min. Both needles were removed and the septum cap was quickly wrapped with parafilm. The vial was transferred to a heating block, which had been preheated at 80 °C, and the reaction mixture was stirred at 200 rpm for 10-30 min. Then, the vial was removed from the heating block, the stirring bar was removed, and the solvent evaporated using a Biotage V10. Trichloroethylene was added as internal standard, $CDCl_3$ (0.5 mL) was added, and the resulting mixture was vigorously shaken for 1 min. Then, an aliquot was taken and submitted to ^{1}H NMR spectroscopic analysis.

The data was interpreted to mean that ***Z*-TT-16** is readily converted within the first few minutes of the reaction, while ***E*-TT-16** reacts much slower.

In order to clarify what ***Z*-TT-16** is converted to, the following experiment was conducted:

A 1 mL GC vial equipped with a Teflon-coated magnetic stirring bar was charged with alkenyl thianthrenium salt **TT-16** (10.0 mg, 26.1 µmol, 1.0 equiv.) and trichloroethylene (6.7 mg, 51 µmol, 2.0 equiv.). d^7-DMF (0.5 mL) was added and the vial was sonicated until all components had fully dissolved. The resulting solution was transferred to a NMR tube and the following ^{1}H NMR spectrum was measured:

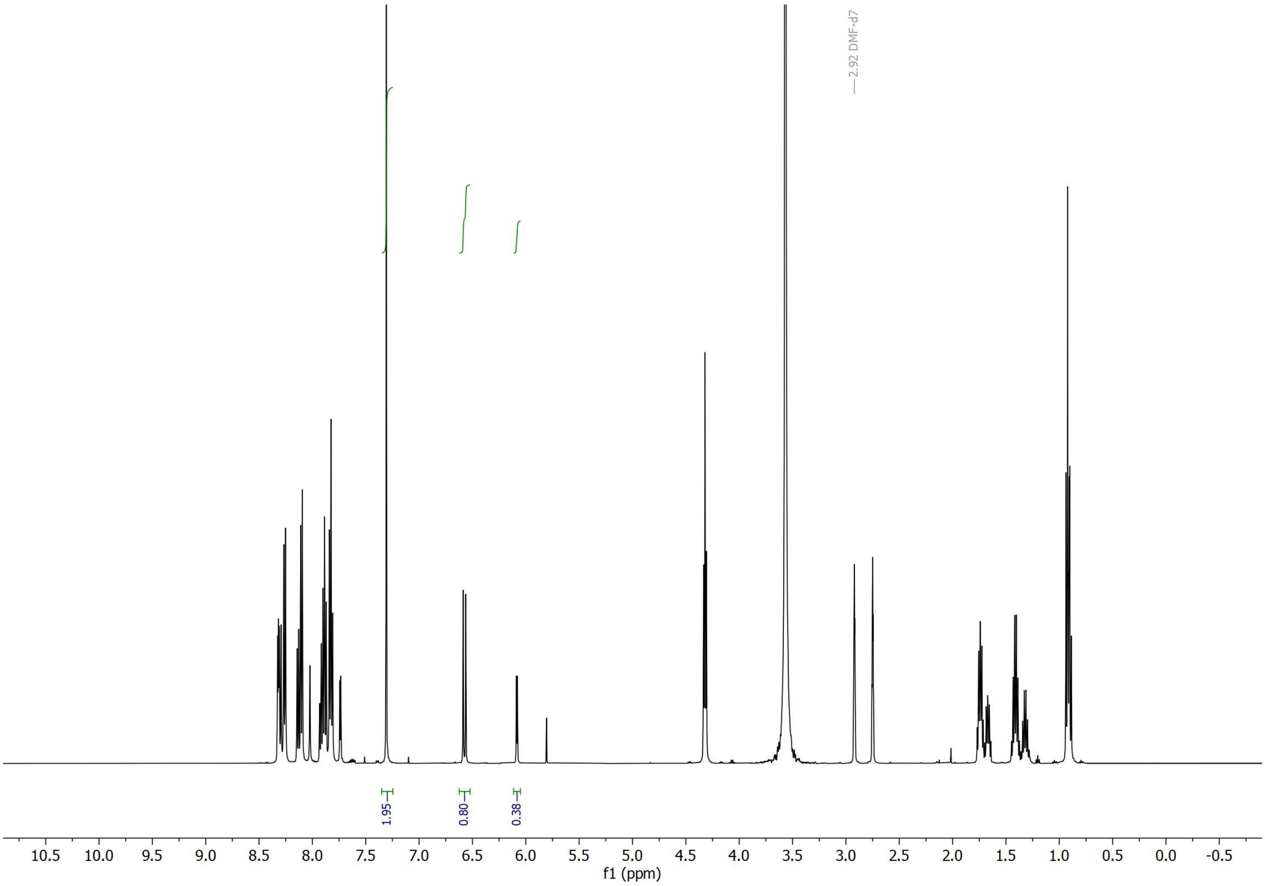

Figure S6: ^{1}H NMR spectrum of the crude reaction mixture before the addition of sodium *p*-toluenesulfinate.

Another 1 mL GC vial was charged with sodium *p*-toluenesulfinate (2.2 mg, 12 µmol, 0.5 equiv.). d^7-DMF (0.2 mL) was added and the vial was sonicated until a clear solution was obtained. The solution was then added to the NMR tube of the previous mixture, the NMR tube was shaken vertically by hand three times, and the following ^{1}H NMR spectrum was obtained:

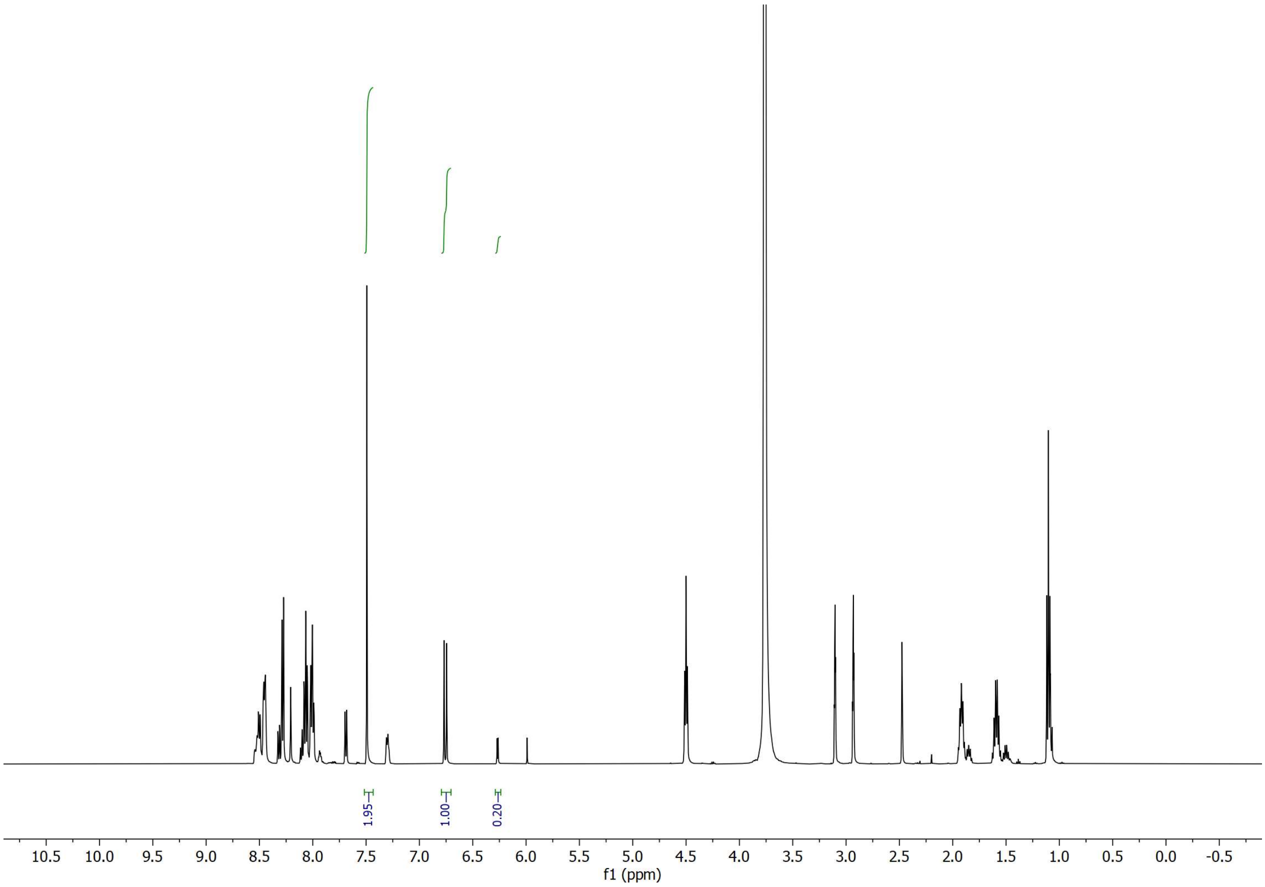

Figure S7: **Figure S4**: ^{1}H NMR spectrum of the crude reaction mixture after the addition of sodium *p*-toluenesulfinate.

By comparing the relative integrals of trichloroethylene (δ = 7.31 ppm, s, 1H), one olefinic signal of ***E*-TT-16** (δ = 6.57 ppm (d, *J* = 12.3 Hz, 1H), and one olefinic signal of ***Z*-TT-16** (δ = 6.08 ppm (d, *J* = 4.7 Hz, 1H), a ratio of 1.95 : 0.80 : 0.38 was determined for the first measurement, while a ratio of 1.95 : 1.00 : 0.20 was determined after the addition of sodium *p*-toluenesulfinate:

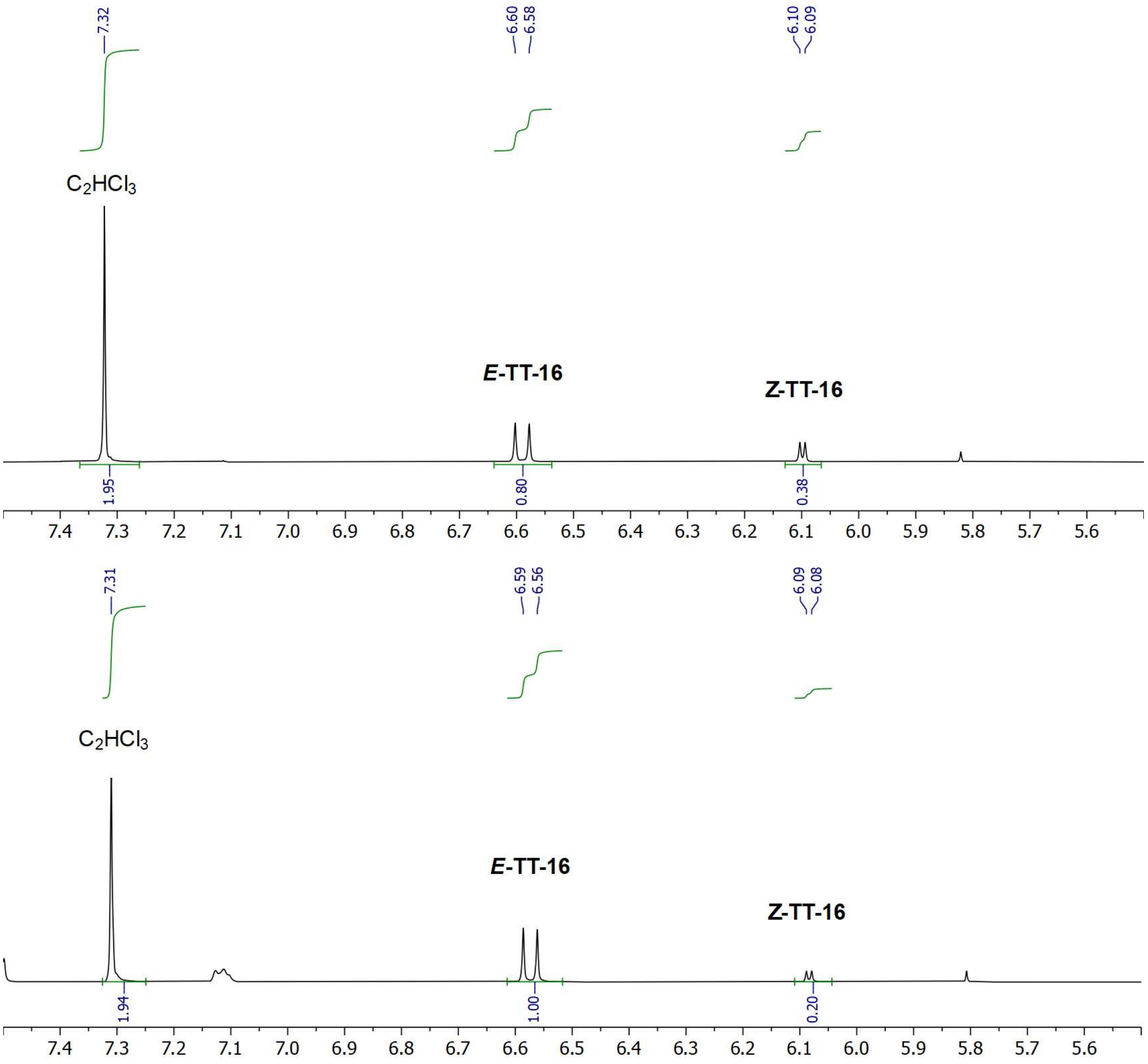

Figure S8: Comparison of the relative integrals of ***E*-TT-16** & ***Z*-TT-16** before (top) and after (bottom) addition of sodium *para*-toluenesulfinate.

The experiment was interpreted to mean that 20% of ***Z*-TT-16** converted to ***E*-TT-16** within minutes in the presence of *p*-toluenesulfinate.

All the obtained data is consistent with ***Z*-TT-16** being readily converted to ***E*-TT-16** in the presence of a nucleophile like sodium *p*-toluenesulfinate, which can be used to rationalize the stereochemical outcome of the synthesis of 1,3-diene **16**. We propose a reversible Michael addition step as a possible explanation for this:

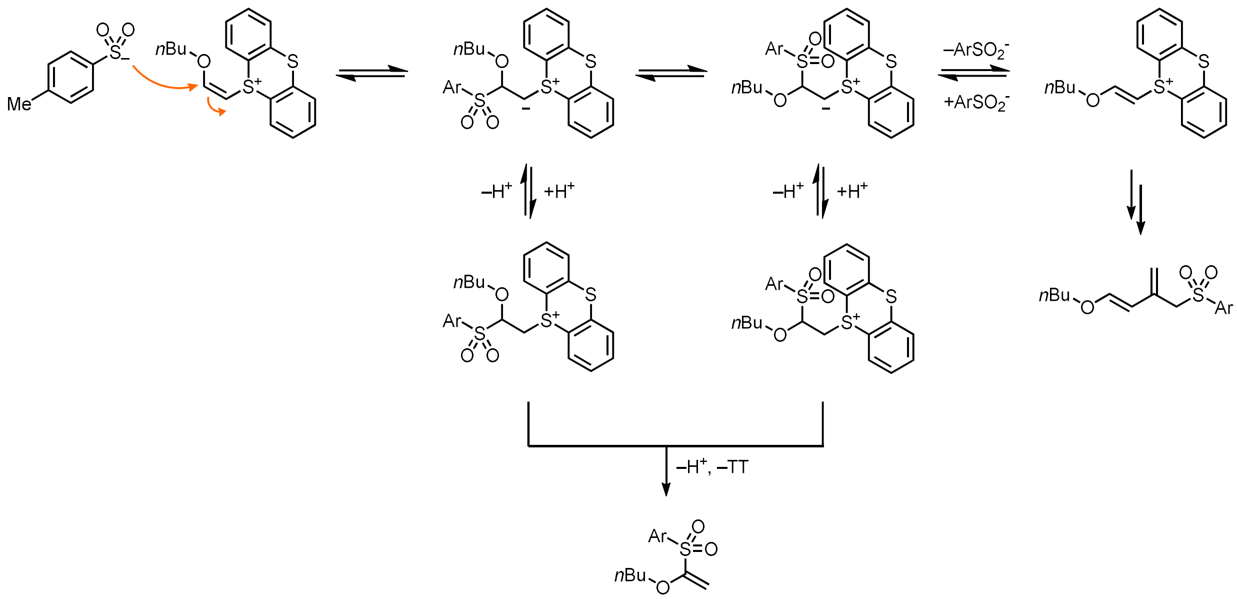

Figure S9: Proposed mechanism for the stereoconvergence of ***Z*-TT-16** to ***E*-TT-16** via reversible Michael addition.

Michael addition of carbon nucleophiles

While for sterically hindered alkenyl-TT salt **TT-18** the synthesis of the corresponding 1,3-dienes could be achieved, it was unclear whether a less hindered alkenyl-TT salt would give the same result considering reversible Michael addition of the carbon nucleophile could potentially be rendered irreversible via fast 3-exo-trig cyclization to afford cyclopropanes instead. To test this hypothesis, the following experiment was conducted:

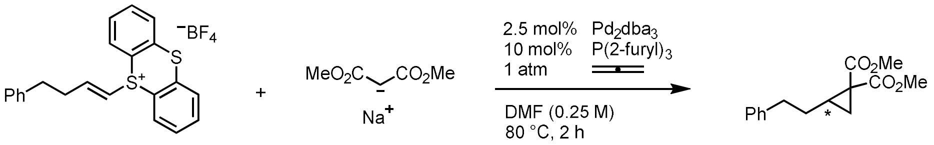

Caution: Propadiene/allene is a flammable gas and should be handled with appropriate care. Guidelines regarding the safe handling of flammable gases can be found in "Prudent Practices in the Laboratory" (Chapter 4.D).[2]

Caution: Heated mixtures of DMF and sodium hydride are known to be potentially explosive.[5] Careful risk assessment should be carried out before performing reactions involving DMF, NaH and heat. Substitution of DMF with 1,4-dioxane was not feasible due to the formation of a thick white goo that was ineffective in the reaction.

A 4 mL borosilicate vial equipped with a Teflon-coated magnetic stirring bar was charged with sodium hydride (18.0 mg, 0.450 mmol, 1.50 equiv., 60% dispersion in mineral oil) and dry DMF (1.2 mL). Under stirring, diethyl malonate (59.5 mg, 51.7 µL, 0.450 mmol, 1.50 equiv.) was added dropwise using a 100 µL Hamilton

syringe, and the resulting mixture was stirred for 15 min at 23 °C. Another 4 mL borosilicate vial equipped with a Teflon-coated magnetic stirring bar was charged with $Pd_2(dba)_3$ (6.9 mg, 7.5 µmol, 2.5 mol%), tri(2-furyl)phosphine (7.0 mg, 30 µmol, 10 mol%) and 4-phenylbutene-dervied alkenyl thianthrenium salt (130 mg, 0.300 mmol, 1.0 equiv.). To the same vial, the solution from the first vial was added and the vial was sealed with a septum cap. The septum was pierced with an outlet needle (Φ 0.80 × 40 mm) and another needle (Φ 0.80 × 120 mm), which was connected to an allene-containing balloon. A gentle stream of allene gas was passed through the mixture for 2 min. Both needles were removed and the septum cap was quickly wrapped with parafilm. The vial was transferred to a heating block, which had been preheated at 80 °C. A blast shield was placed in front of the stirring plate, and the reaction mixture was stirred at 1000 rpm for 2 h. Then, the vial was removed from the heating block, the stirring bar was removed, and the solvent evaporated using a Biotage V10. The residue was purified by column chromatography on silica gel eluting with pentane/EtOAc (30:1 → 20:1 → 9:1 → 8:2, v/v) to afford (±)-**CP-1** (66.7 mg, 85%) as a colorless oil.

R_f = 0.39 (hexanes/EtOAc = 9:1 (v/v), $KMnO_4$).

NMR Spectroscopy:

^{1}H NMR (500 MHz, $CDCl_3$) δ 7.28 (t, *J* = 7.6 Hz, 2H), 7.21 – 7.12 (m, 3H), 3.76 (s, 3H), 3.72 (s, 3H), 2.79 – 2.65 (m, 2H), 1.99 – 1.89 (m, 1H), 1.82 – 1.71 (m, 1H), 1.60 – 1.48 (m, 1H), 1.42 (dd, *J* = 9.0, 4.6 Hz, 1H), 1.38 (dd, *J* = 7.7, 4.6 Hz, 1H).

^{13}C NMR (126 MHz, $CDCl_3$) δ 170.9, 168.7, 141.4, 128.5, 126.1, 52.6, 35.2, 34.0, 30.9, 28.3, 21.3.

HRMS-ESI (m/z) calc'd for $C_{15}H_{18}O_4$ $[M+Na]^+$, 285.1097; found, 285.1095; deviation: +0.7 ppm.

The data obtained is consistent with reversible Michael addition followed by intramolecular cyclization being faster than Pd-catalyzed 1,3-diene formation.

Preparation of alkenes

Synthesis of Alk-2

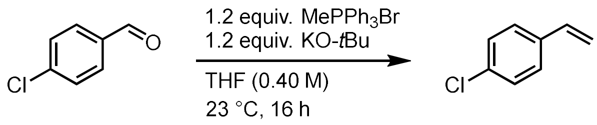

10 mmol **Alk-2**, 55%

The reaction was carried out according to a previously reported procedure.[6] Under an argon atmosphere, a flame-dried 100 mL Schlenk flask equipped with a Teflon-coated magnetic stirring bar was charged with methyltriphenylphosphonium bromide (4.3 g, 12 mmol, 1.2 equiv.) and anhydrous THF (25 mL, c = 0.40 M). Potassium *tert*-butoxide (1.3 g, 12 mL, 1.0 M solution in THF, 12 mmol, 1.2 equiv.) was added at once, leading to a yellow mixture. The resulting mixture was stirred at 23 °C for 90 min. Then, 4-chlorobenzaldehyde (1.4 g, 10 mmol, 1.0 equiv.) was dissolved in anhydrous THF (3.0 mL, c = 3.3 M), and the resulting solution was added at once to the yellow mixture. The resulting yellow mixture was stirred at 23 °C for 16 h. Water (40 mL) and DCM (50 mL) were added, and the phases were separated. The organic phase was dried over Na_2SO_4, filtered, and concentrated under vacuum. The resulting residue was purified by column chromatography on silica gel eluting with pentane (100%) to afford **Alk-2** (768 mg, 55%) as a colorless liquid.

R_f = 0.74 (pentane/DCM = 19:1 (v/v), $KMnO_4$).

NMR Spectroscopy:

^{1}H NMR (500 MHz, $CDCl_3$) δ 7.37 – 7.32 (m, 2H), 7.32 – 7.28 (m, 2H), 6.68 (dd, *J* = 17.6, 10.9 Hz, 1H), 5.79 – 5.68 (m, 1H), 5.28 (d, *J* = 10.9 Hz, 1H).

^{13}C NMR (126 MHz, $CDCl_3$) δ 136.2, 135.8, 133.6, 128.8, 127.6, 114.6.

HRMS-EI (m/z) calc'd for $C_8H_7Cl_1$ $[M]^+$, 138.0231; found, 138.0231; deviation: –0.6 ppm.

The obtained data are consistent with those previously reported in the literature.[7]

Synthesis of Alk-8

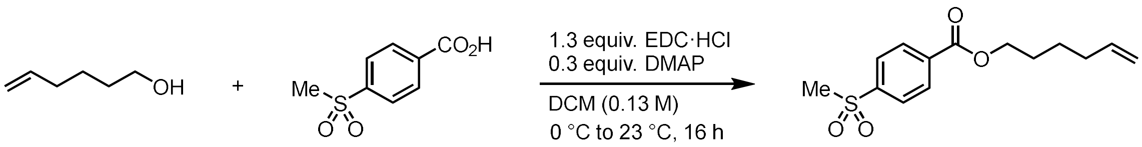

1.2 equiv. 5.0 mmol **Alk-8**, 79%

Under an ambient atmosphere, a 100 mL round-bottom flask equipped with a Teflon-coated magnetic stirring bar was charged with 4-(methylsulfonyl)benzoic acid (1.0 g, 5.0 mmol, 1.0 equiv.) and DCM (38 mL, c =

0.13 M). The resulting mixture was cooled to 0 °C using an ice bath. Under stirring, EDC·HCl (1.2 g, 6.3 mmol, 1.3 equiv.) was added. After 15 min, 5-hexen-1-ol (0.72 mL, 6.0 mmol, 1.2 equiv.) was added, followed by DMAP (0.15 g, 1.3 mmol, 0.30 equiv.). The resulting mixture was allowed to warm to 23 °C and was stirred for 16 h. Then, water (30 mL) was added, the layers were separated, the aqueous phase was extracted with DCM (2 × 20 mL). The unified organic layer was dried over Na_2SO_4, filtered, and concentrated under vacuum. The resulting residue was purified by column chromatography on silica gel eluting with a solvent mixture of pentane/EtOAc (3:1 (v/v)) to afford **Alk-8** (1.11 g, 79%) as a white solid.

R*f* = 0.15 (pentane/EtOAc = 5:1 (v/v), $KMnO_4$).

NMR Spectroscopy:

^{1}**H NMR** (500 MHz, $CDCl_3$) δ 8.23 – 8.21 (m, 2H), 8.03 – 8.01 (m, 2H), 5.85 – 5.77 (m, 1H), 5.05 – 5.01 (m, 1H), 4.99 – 4.97 (m, 1H), 4.37 (t, *J* = 6.6 Hz, 2H), 3.07 (s, 3H), 2.15 – 2.11 (m, 2H), 1.83 – 1.78 (m, 2H), 1.58 – 1.53 (m, 2H).

^{13}**C NMR** (126 MHz, $CDCl_3$) δ 165.1, 144.3, 138.2, 135.3, 130.6, 127.6, 115.1, 65.8, 44.4, 33.3, 28.1, 25.3.

HRMS-ESI (m/z) calc'd for $C_{14}H_{18}S_2O_4$ $[M+Na]^+$, 305.0818; found, 305.0818; deviation: –0.1 ppm.

Synthesis of Alk-12

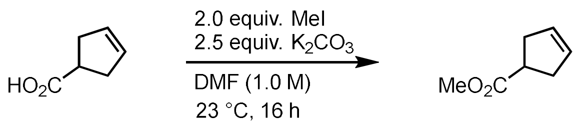

10 mmol **Alk-12**, 95%

Under an ambient atmosphere, a 25 mL round-bottom flask equipped with a Teflon-coated magnetic stirring bar was charged with 3-cyclopentenoic acid (1.1 g, 10 mmol, 1.0 equiv.), K_2CO_3 (3.4 g, 25 mmol, 2.5 equiv.) and DMF (10 mL, c = 1.0 M). After 15 min of stirring at 500 rpm, methyliodide (2.8 g, 1.2 mL, 20 mmol, 2.0 equiv.) was added, and the resulting mixture was stirred at 23 °C for 16 h. Then, water (30 mL) was added, the layers were separated, the aqueous phase was extracted with Et_2O (2 × 20 mL). The unified organic layer was dried over Na_2SO_4, filtered, and concentrated under vacuum. The resulting residue was purified by column chromatography on silica gel eluting with a solvent mixture of pentane/Et_2O (19:1 (v/v)) to afford **Alk-12** (630 mg, 95%) as a colorless liquid.

R*f* = 0.56 (pentane/Et_2O = 19:1 (v/v), $KMnO_4$).

NMR Spectroscopy:

^{1}**H NMR** (500 MHz, $CDCl_3$) δ 5.66 (s, 2H), 3.69 (s, 3H), 3.16 – 3.07 (m, 1H), 2.64 (d, *J* = 8.2 Hz, 4H).

^{13}**C NMR** (126 MHz, $CDCl_3$) δ 176.8, 129.1, 51.9, 41.6, 36.5.

HRMS-EI (m/z) calc'd for $C_7H_{10}O_2$ $[M]^+$, 126.0675; found, 126.0676; deviation: –0.5 ppm.

The obtained data are consistent with those previously reported in the literature.[8]

Synthesis of Alk-15

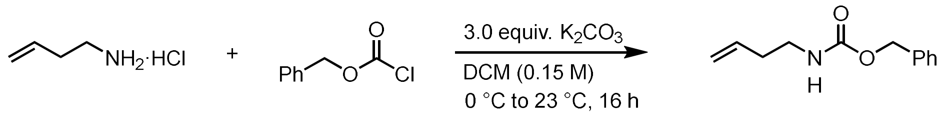

5.0 mmol 7.5 mmol **Alk-15**, 92%

The reaction was carried out according to a previously reported procedure.[9] Under an ambient atmosphere, a 100 mL round-bottom flask equipped with a Teflon-coated magnetic stirring bar was charged with 3-butenylamine hydrochloride (0.53 g, 5.0 mmol, 1.0 equiv.), K_2CO_3 (2.1 g, 15 mmol, 3.0 equiv.) and DCM (33 mL, c = 0.15 M). The resulting mixture was cooled to 0 °C. Under stirring, benzyl chloroformate (1.3 g, 1.0 mL, 7.5 mmol, 1.5 equiv.) was added dropwise (1 drop per second). After 15 min, the resulting mixture was stirred allowed to warm to 23 °C, and stirred for 16 h. Then, water (30 mL) was added, the layers were separated, the aqueous phase was extracted with DCM (2 × 30 mL). The unified organic layer was dried over Na_2SO_4, filtered, and concentrated under vacuum. The resulting residue was purified by column chromatography on silica gel eluting with a solvent mixture of pentane/EtOAc (19:1 → 9:1 (v/v)) to afford **Alk-15** (944 mg, 92%) as a colorless oil.

R_f = 0.32 (pentane/EtOAc = 9:1 (v/v), $KMnO_4$).

NMR Spectroscopy:

^{1}H NMR (500 MHz, $CDCl_3$) δ 7.41 – 7.36 (m, 4H), 7.36 – 7.31 (m, 1H), 5.85 – 5.69 (m, 1H), 5.21 – 5.05 (m, 4H), 4.91 – 4.75 (m, 1H), 3.30 (q, J = 6.5 Hz, 2H), 2.29 (q, J = 6.9 Hz, 2H).

^{13}C NMR (126 MHz, $CDCl_3$) δ 156.5, 136.7, 135.2, 128.6, 128.3, 128.2, 117.5, 66.8, 40.2, 34.2.

HRMS-ESI (m/z) calc'd for $C_{12}H_{15}N_1O_2$ $[M+Na]^+$, 228.0995; found, 228.0995; deviation: –0.1 ppm.

The obtained data are consistent with those previously reported in the literature.[9]

Preparation of alkenyl thianthrenium salts

Alkenyl thianthrenium salt summary

Previously reported alkenyl-TTs:

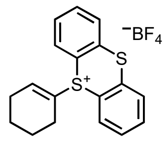

TT-1, 60%

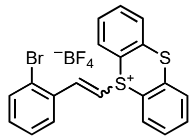

TT-3, 76% (*E*/*Z* > 20:1)

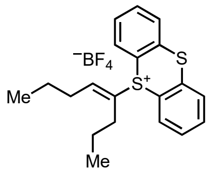

TT-4, 84% (E/Z > 20:1)

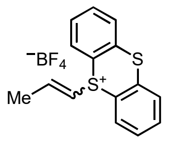

TT-5, 54% (*E*/*Z* = 13:1)

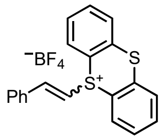

TT-6, 95% (*E*/*Z* > 20:1)

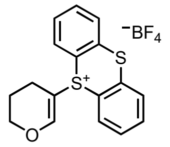

TT-13, 47%

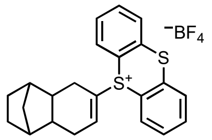

TT-14, 52%

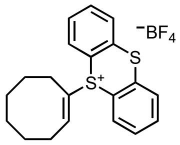

TT-18, 48%

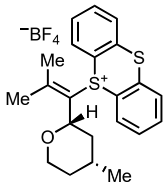

TT-23, 61%

Commercially available:

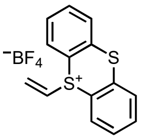

vinyl-TT

Previously unknown alkenyl-TTs:

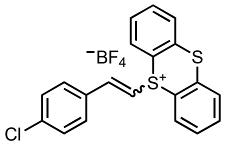

TT-2, 76% (*E*/*Z* > 20:1)

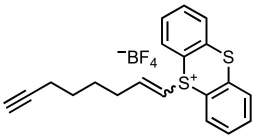

TT-7, 79% (*E*/*Z* = 11:1)

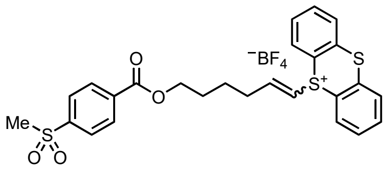

TT-8, 76% (*E*/*Z* = 20:1)

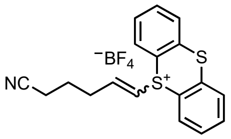

TT-9, 79% (*E*/*Z* = 8:1)

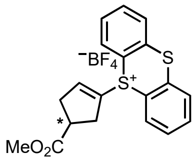

(±)-**TT-12**, 80%

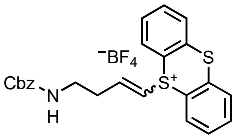

TT-15, 42% (*E*/*Z* > 20:1)

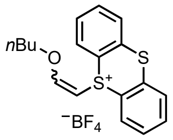

TT-16, 66% (*E*/*Z* = 1:1.6)

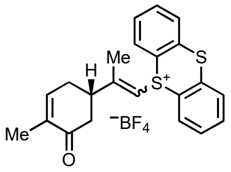

TT-21, 37% (*E*/*Z* = 7:1)

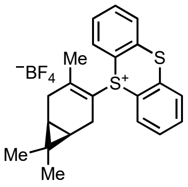

TT-22, 46%

Preparation of thianthrene-S-oxide

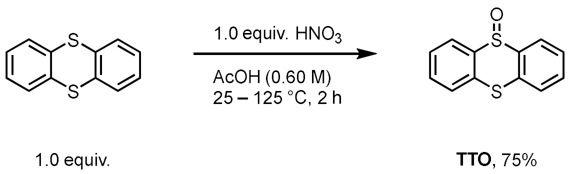

The reaction as carried out according to a previously reported procedure.[10]

A 1 L two-neck round-bottomed flask was charged with a Teflon-coated magnetic stirring bar. Thianthrene (50 g, 0. 23 mol, 1.0 equiv.) and acetic acid (0.60 mol/L, 0.39 L, 14 g, 0.23 mol, 1.0 equiv.) were added to the flask. The central neck of the flask was fitted with a reflux condenser with the outlet passing through a round-bottomed flask before entering a Drechsel bottle containing an aqueous sodium hydroxide solution. The side-neck of the flask was fitted with a 100 mL dropping funnel containing nitric acid (2.7 mol/L, 90 mL, 15 g, 0.24 mol, 1.0 equiv.). The reaction mixture was refluxed at 125 °C (in an oil bath) while the nitric acid was added dropwise over 90 min. Afterwards, the mixture was refluxed for additional 15 min. The yellow solution was cooled down to room temperature and poured into ice water (750 mL). The resulting suspension was filtered and washed with cold water (3 × 100 mL). The solid was dissolved in DCM, poured into a separating funnel and the layers were separated. The organic phase was concentrated under reduced pressure by rotary evaporation. The resulting solid was recrystallized from ethyl acetate. The resulting solution was filtered under vacuum, the obtained solid was dissolved in DCM, and precipitated with a large excess of hexanes. The resulting mixture under vacuum, and the thus obtained solid was dried under vacuum to give **TTO** as a colorless solid (40 g, 75% yield).

NMR Spectroscopy:

^{1}H NMR (600 MHz, CD_3CN, 23 °C, δ): 7.86 (ddd, J = 7.8, 1.4, 0.5 Hz, 2H), 7.73 (ddd, J = 7.7, 1.1, 0.5 Hz, 2H), 7.62 (dd, J = 15.2, 1.1 Hz, 2H), 7.52 (ddd, J = 7.8, 7.4, 1.4 Hz, 2H).

^{13}C NMR (151 MHz, CD_3CN, 23 °C, δ): 142.6, 131.2, 130.3, 129.7, 129.3, 125.1.

HRMS-EI(m/z) calc'd for $C_{12}H_{18}OS_2$ $[M]^+$, 232.0011; found 232.0010; deviation: +0.7 ppm.

The obtained data are consistent with those previously reported in the literature.[10]

Alkenyl thianthrenium salt TT-1

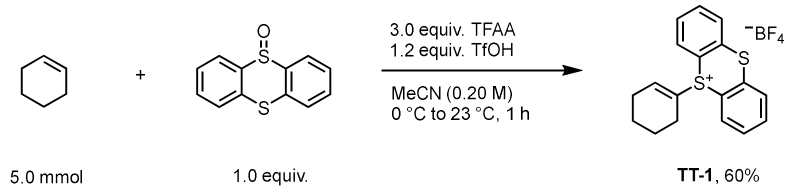

The reaction was carried out according to a previously reported procedure.[11] Under an ambient atmosphere, a 50 mL round-bottom flask with a magnetic stirring bar was charged with cyclohexene (0.41 g, 0.51 mL, 5.0

mmol, 1.0 equiv.), thianthrene-S-oxide (1.16 g, 2.00 mmol, 1.0 equiv.) and MeCN (20 ml, c = 0.25 M). The mixture was cooled to 0 °C using an ice bath. Trifluoroacetic anhydride (3.2 g, 2.1 mL, 15 mmol, 3.0 equiv.) was added dropwise (2 drops per second) leading to a dark purple solution. Triflic acid (0.90 g, 0.53 mL, 6.0 mmol, 1.2 equiv.) was added dropwise (2 drops per second), and the mixture was stirred at 0 °C for 45 min followed by stirring at 25 °C for 15 min. The resulting mixture was diluted with DCM (30 mL) and was poured onto a saturated aqueous $NaHCO_3$ solution (30 mL). The layers were separated and the aqueous layer was further extracted with DCM (2 × 20 mL). The unified organic layer was washed with aqueous $NaBF_4$ solution (2 × 50 mL, 10% w/w). The organic layer was dried with Na_2SO_4, filtered, and the solvent was removed under reduced pressure. The residue was purified by chromatography on silica gel eluting with pentane/EtOAc (3:1, v/v) → DCM/MeOH (19:1, v/v) to afford a slightly yellowish solid residue, which was dissolved in DCM (10 mL), and then precipitated from Et_2O (120 mL) to afford **TT-1** (1.2 g, 60%) as a white powder.

R_f = 0.46 (DCM/MeOH = 19:1 (v/v)).

NMR Spectroscopy:

^{1}H NMR (500 MHz, $CDCl_3$) δ 8.32 (dd, *J* = 7.9, 1.3 Hz, 2H), 7.84 – 7.74 (m, 4H), 7.70 (td, *J* = 7.6, 1.7 Hz, 2H), 6.13 – 6.02 (m, 1H), 2.24 – 2.16 (m, 2H), 2.02 – 1.91 (m, 2H), 1.74 – 1.64 (m, 2H), 1.60 – 1.49 (m, 2H).

^{13}C NMR (126 MHz, $CDCl_3$) δ 141.3, 136.3, 135.2, 134.8, 130.1, 130.0, 123.6, 116.9, 27.2, 25.3, 22.5, 20.3.

^{19}F NMR (471 MHz, $CDCl_3$) δ –151.48, –151.53.

HRMS-ESI (m/z) calc'd for $C_{18}H_{17}S_2$ $[M]^+$, 297.0766; found, 297.0763; deviation: +1.2 ppm.

The obtained data are consistent with those previously reported in the literature.[11]

Alkenyl thianthrenium salt TT-2

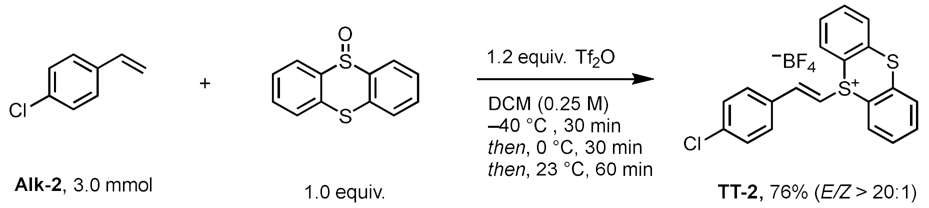

The reaction was carried out according to a previously reported procedure.[12] Under an ambient atmosphere, a 50 mL round-bottom flask with a magnetic stirring bar was charged with thianthrene-S-oxide (0.70 g, 3.0 mmol, 1.0 equiv.), **Alk-2** (0.42 g, 3.0 mmol, 1.0 equiv.) and DCM (12 mL, c = 0.25 M). The mixture was cooled to –40 °C using an acetone/dry ice bath. Triflic anhydride (1.0 g, 0.60 mL, 3.6 mmol, 1.2 equiv.) was added dropwise (2 drops per second), and the resulting purple solution was stirred at –40 °C for 30 min. Then, the cooling bath was exchanged with an ice bath, and the reaction was stirred at 0 °C for 30 min followed by stirring at 23 °C for 2 h. Saturated aqueous $NaHCO_3$ solution (20 mL) was added. The layers were separated and the aqueous layer was further extracted with DCM (2 × 10 mL). The unified organic layer

was washed with aqueous $NaBF_4$ solution (2 × 40 mL, 10% w/w). The organic layer was dried with Na_2SO_4, filtered, and the solvent was removed under reduced pressure. The residue was purified by chromatography on silica gel eluting with pentane/EtOAc (3:1, v/v) → DCM/MeOH (19:1, v/v) to afford **TT-2** (1.01 g, 76%, *E*/*Z* > 20:1) as a beige solid.

R_f = 0.49 (DCM/MeOH = 19:1 (v/v)).

NMR Spectroscopy:

^{1}H NMR (500 MHz, $CDCl_3$) δ 8.34 – 8.27 (m, 2H), 7.96 (d, *J* = 15.1 Hz, 1H), 7.87 – 7.80 (m, 2H), 7.74 (td, *J* = 7.7, 1.4 Hz, 2H), 7.63 (td, *J* = 7.7, 1.4 Hz, 2H), 7.47 – 7.39 (m, 2H), 7.25 – 7.18 (m, 2H), 7.10 (d, *J* = 15.2 Hz, 1H).

^{13}C NMR (126 MHz, $CDCl_3$) δ 150.1, 138.3, 135.9, 134.7, 133.6, 130.7, 130.4, 129.4, 120.4, 107.2.

^{19}F NMR (471 MHz, $CDCl_3$) δ –149.88, –149.91 – –149.95 (m).

HRMS-ESI (m/z) calc'd for $C_{20}H_{14}S_2Cl_1$ $[M]^+$, 353.0220; found, 353.0221; deviation: –0.3 ppm.

Alkenyl thianthrenium salt TT-3

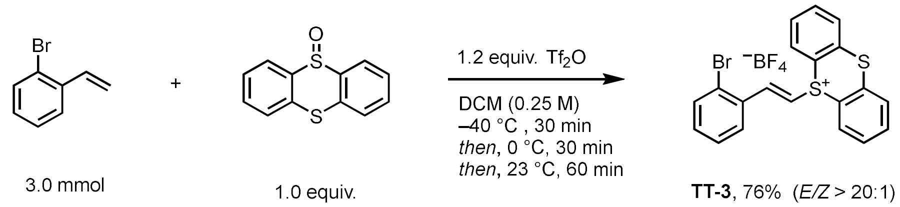

The reaction was carried out according to a previously reported procedure.[12] Under an ambient atmosphere, a 50 mL round-bottom flask with a magnetic stirring bar was charged with thianthrene-S-oxide (0.70 g, 3.0 mmol, 1.0 equiv.), 2-bromostyrene (0.55 g, 3.0 mmol, 1.0 equiv.) and DCM (12 mL, c = 0.25 M). The mixture was cooled to –40 °C using an acetone/dry ice bath. Triflic anhydride (1.0 g, 0.60 mL, 3.6 mmol, 1.2 equiv.) was added dropwise (2 drops per second), and the resulting purple solution was stirred at –40 °C for 30 min. Then, the cooling bath was exchanged with an ice bath, and the reaction was stirred at 0 °C for 30 min followed by stirring at 23 °C for 2 h. Saturated aqueous $NaHCO_3$ solution (20 mL) was added. The layers were separated and the aqueous layer was further extracted with DCM (2 × 10 mL). The unified organic layer was washed with aqueous $NaBF_4$ solution (2 × 40 mL, 10% w/w). The organic layer was dried with Na_2SO_4, filtered, and the solvent was removed under reduced pressure. The residue was purified by chromatography on silica gel eluting with pentane/EtOAc (3:1, v/v) → DCM/MeOH (19:1, v/v) to afford **TT-3** (1.11 g, 76%, *E*/*Z* > 20:1) as a beige solid.

R_f = 0.44 (DCM/MeOH = 19:1 (v/v)).

NMR Spectroscopy:

^{1}H NMR (500 MHz, $CDCl_3$) δ 8.42 – 8.34 (m, 2H), 7.86 (dd, *J* = 8.0, 1.3 Hz, 2H), 7.78 (td, *J* = 7.6, 1.4 Hz, 2H), 7.69 (td, *J* = 7.7, 1.4 Hz, 2H), 7.56 (dd, *J* = 7.8, 1.8 Hz, 1H), 7.50 – 7.42 (m, 2H), 7.25 – 7.22 (m,

1H), 7.21 – 7.16 (m, 1H), 6.96 (d, *J* = 14.9 Hz, 1H).

^{13}C NMR (126 MHz, $CDCl_3$) δ 145.4, 136.4, 134.9, 134.4, 133.4, 132.9, 132.1, 130.4, 130.3, 129.4, 128.4, 125.0, 118.6, 110.4.

^{19}F NMR (471 MHz, $CDCl_3$) δ –150.14, –150.18 – –150.20 (m).

HRMS-ESI (m/z) calc'd for $C_{20}H_{14}Br_1S_2$ $[M]^+$, 396.9715; found 396.9719; deviation: –1.0 ppm.

The obtained data are consistent with those previously reported in the literature.[13]

Alkenyl thianthrenium salt TT-4

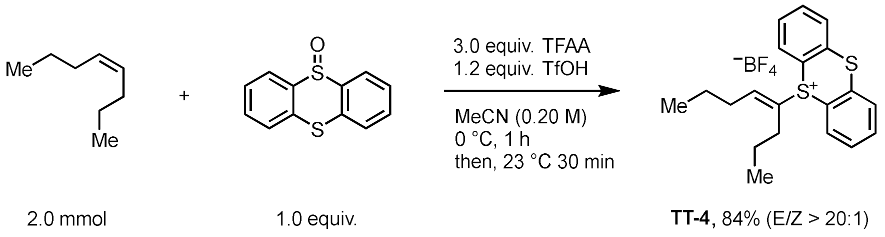

The reaction was carried out according to a previously reported procedure.[11] Under an ambient atmosphere, a 20 mL glass vial with a magnetic stirring bar was charged with *cis*-4-octene (0.22 g, 0.31 mL, 2.0 mmol, 1.0 equiv.), thianthrene-S-oxide (0.46 g, 2.0 mmol, 1.0 equiv.) and MeCN (8.0 mL, c = 0.20 M). The mixture was cooled to 0 °C using an ice bath. Trifluoroacetic anhydride (1.3 g, 0.83 mL 6.0 mmol, 3.0 equiv.) was added dropwise (2 drops per second) leading to a dark purple solution. Trifluoromethanesulfonic acid (0.36 g, 0.21 mL, 2.4 mmol, 1.2 eq.) was added dropwise (2 drops per second), and the mixture was stirred at 0 °C for 60 min followed by stirring at 25 °C for 30 min. The resulting mixture was diluted with DCM (10 mL) and was poured onto a saturated aqueous $NaHCO_3$ solution (20 mL). The layers were separated and the aqueous layer was further extracted with DCM (2 × 10 mL). The unified organic layer was washed with aqueous $NaBF_4$ solution (2 × 20 mL, 10% w/w). The organic layer was dried with Na_2SO_4, filtered, and the solvent was removed under reduced pressure. The residue was purified by chromatography on silica gel eluting with pentane/EtOAc (3:1, v/v) → DCM/MeOH (19:1, v/v) to afford **TT-4** (697 mg, 84%, *E*/*Z* > 20:1) as a colorless solid.

R_f = 0.39 (DCM/MeOH = 19:1 (v/v)).

NMR Spectroscopy:

^{1}H NMR (500 MHz, $CDCl_3$) δ 8.37 (dd, *J* = 7.9, 1.3 Hz, 2H), 7.87 – 7.78 (m, 4H), 7.76 – 7.70 (m, 2H), 5.49 (t, *J* = 7.6 Hz, 1H), 2.20 – 2.07 (m, 4H), 1.38 – 1.22 (m, 4H), 0.86 – 0.74 (m, 6H).

^{13}C NMR (126 MHz, $CDCl_3$) δ 142.2, 136.7, 135.6, 134.9, 130.3, 130.1, 123.7, 117.1, 31.5, 30.1, 21.8, 21.6, 13.7, 13.7.

^{19}F NMR (471 MHz, $CDCl_3$) δ –151.61, –151.66.

HRMS-ESI (m/z) calc'd for $C_{20}H_{23}S_2$ $[M]^+$, 327.1236; found 327.1233; deviation: +0.9 ppm.

The obtained data are consistent with those previously reported in the literature.[11]

Alkenyl thianthrenium salt TT-5

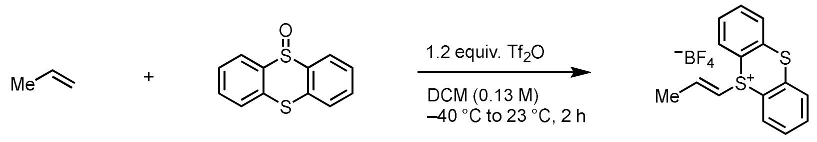

2.0 mmol 1.0 equiv. **TT-5**, 54% (*E*/*Z* = 13:1)

The reaction was carried out according to a previously reported procedure.[14] Under an ambient atmosphere, a 50 mL round-bottom flask with a magnetic stirring bar was charged with thianthrene-S-oxide (0.93 g, 4.0 mmol, 1.0 equiv.) and DCM (30 mL, c = 0.13 M). The mixture was cooled to –40 °C using an acetone/dry ice bath. Propylene was bubbled trough the solution under stirring for 15 min at –40 °C. Then, the bubbling was stopped and a propylene balloon was attached to maintain a constant atmosphere. Triflic anhydride (1.35 g, 0.81 mL 4.8 mmol, 1.2 equiv.) was added dropwise (2 drops per second) leading to a dark purple solution. The resulting mixture was diluted with DCM (10 mL) and was poured onto a saturated aqueous $NaHCO_3$ solution (20 mL). The layers were separated and the aqueous layer was further extracted with DCM (2 × 10 mL). The unified organic layer was washed with aqueous $NaBF_4$ solution (2 × 20 mL, 10% w/w). The organic layer was dried with Na_2SO_4, filtered, and the solvent was removed under reduced pressure. The residue was purified by chromatography on silica gel eluting with pentane/EtOAc (3:1, v/v) → DCM/MeOH (19:1, v/v) to afford **TT-5** (747 mg, 54%, *E*/*Z* = 13:1) as an ocher solid.

R*f* = 0.44 (DCM/MeOH = 19:1 (v/v)).

NMR Spectroscopy:

^{1}H NMR (500 MHz, $CDCl_3$) δ 8.24 (dd, *J* = 7.9, 1.4 Hz, 2H), 7.82 (dd, *J* = 8.0, 1.4 Hz, 2H), 7.73 (td, *J* = 7.7, 1.4 Hz, 2H), 7.62 (td, *J* = 7.7, 1.4 Hz, 2H), 7.30 – 7.17 (m, 1H), 6.65 – 6.56 (m, 1H), 1.94 (dd, *J* = 7.0, 1.7 Hz, 3H).

^{13}C NMR (126 MHz, $CDCl_3$) δ 153.2, 135.5, 134.6, 133.3, 130.3, 130.2, 120.4, 110.4, 19.2.

^{19}F NMR (471 MHz, $CDCl_3$) δ –150.82, –150.87.

HRMS-ESI (m/z) calc'd for $C_{15}H_{13}S_2$ $[M]^+$, 257.0453; found, 257.0451; deviation: +0.7 ppm.

The obtained data are consistent with those previously reported in the literature.[14]

Alkenyl thianthrenium salt TT-6

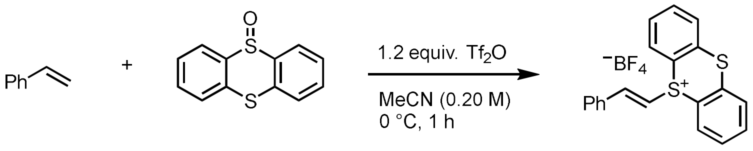

2.0 mmol 1.0 equiv. **TT-6**, 95% (*E*/*Z* > 20:1)

The reaction was carried out according to a previously reported procedure.[12] Under an ambient atmosphere, a 20 mL glass vial with a magnetic stirring bar was charged with styrene (0.21 g, 2.0 mmol, 1.0 equiv.), thianthrene-S-oxide (0.46 g, 2.0 mmol, 1.0 equiv.) and MeCN (8.0 mL, c = 0.20 M). The mixture was cooled to 0 °C using an ice bath. Triflic anhydride (0.68 g, 0.40 mL, 2.4 mmol, 1.2 equiv.) was added dropwise (2 drops per second), and the mixture was stirred at 0 °C for 60 min. The resulting mixture was diluted with DCM (10 mL) and was poured onto a saturated aqueous $NaHCO_3$ solution (20 mL). The layers were separated and the aqueous layer was further extracted with DCM (2 × 10 mL). The unified organic layer was washed with aqueous $NaBF_4$ solution (2 × 20 mL, 10% w/w). The organic layer was dried with Na_2SO_4, filtered, and the solvent was removed under reduced pressure. The residue was purified by chromatography on silica gel eluting with pentane/EtOAc (3:1, v/v) → DCM/MeOH (19:1, v/v) to afford **TT-6** (771 mg, 95%, *E*/*Z* > 20:1) as a grey solid.

R*f* = 0.46 (DCM/MeOH = 19:1 (v/v)).

NMR Spectroscopy:

^{1}H NMR (500 MHz, $CDCl_3$) δ 8.44 (dd, *J* = 7.9, 1.4 Hz, 2H), 8.12 (d, *J* = 15.1 Hz, 1H), 7.87 (dd, *J* = 7.9, 1.3 Hz, 2H), 7.76 (td, *J* = 7.7, 1.4 Hz, 2H), 7.69 (td, *J* = 7.7, 1.4 Hz, 2H), 7.54 – 7.48 (m, 2H), 7.44 – 7.38 (m, 1H), 7.38 – 7.32 (m, 2H), 7.13 (d, *J* = 15.1 Hz, 1H).

^{13}C NMR (126 MHz, $CDCl_3$) δ 152.0, 135.8, 134.5, 134.0, 132.5, 132.2, 130.6, 130.2, 129.3, 129.2, 121.0, 106.2.

^{19}F NMR (471 MHz, $CDCl_3$) δ –150.08, –150.09 – –150.17 (m).

HRMS-ESI (m/z) calc'd for $C_{20}H_{15}S_2$ $[M]^+$, 319.0610; found 319.0609; deviation: +0.2 ppm.

The obtained data are consistent with those previously reported in the literature.[12]

Alkenyl thianthrenium salt TT-7

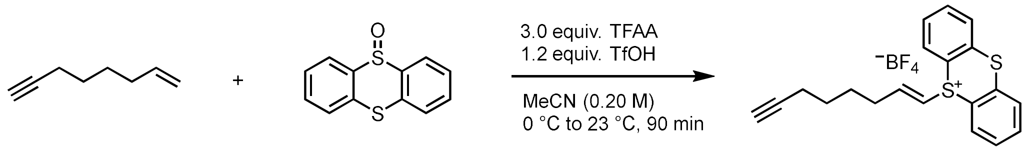

2.0 mmol 1.0 equiv. **TT-7**, 79% (*E*/*Z* = 11:1)

Under an ambient atmosphere, a 20 mL glass vial with a magnetic stirring bar was charged with 1-octen-1-yne (0.22 g, 0.23 mL, 2.0 mmol, 1.0 equiv.), thianthrene-S-oxide (0.46 g, 2.0 mmol, 1.0 equiv.) and MeCN (8.0 mL, c = 0.20 M). The mixture was cooled to 0 °C using an ice bath. Trifluoroacetic anhydride (1.3 g, 0.83 mL 6.0 mmol, 3.0 equiv.) was added dropwise (2 drops per second) leading to a dark purple solution. Trifluoromethanesulfonic acid (0.36 g, 0.21 mL, 2.4 mmol, 1.2 eq.) was added dropwise (2 drops per second), and the mixture was stirred at 0 °C for 60 min followed by stirring at 25 °C for 30 min. The resulting mixture was diluted with DCM (10 mL) and was poured onto a saturated aqueous $NaHCO_3$ solution (20 mL). The layers were separated and the aqueous layer was further extracted with DCM (2 × 10 mL). The unified

organic layer was washed with aqueous $NaBF_4$ solution (2 × 20 mL, 10% w/w). The organic layer was dried with Na_2SO_4, filtered, and the solvent was removed under reduced pressure. The residue was purified by chromatography on silica gel eluting with pentane/EtOAc (3:1, v/v) → DCM/MeOH (19:1, v/v) to afford **TT-7** (508 mg, 79%, *E*/*Z* = 11:1) as a colorless oil.

R*f* = 0.46 (DCM/MeOH = 19:1 (v/v)).

NMR Spectroscopy:

^{1}H NMR (500 MHz, $CDCl_3$) δ 8.36 (dd, *J* = 7.9, 1.4 Hz, 2H), 7.83 (dd, *J* = 7.9, 1.4 Hz, 2H), 7.74 (td, *J* = 7.7, 1.4 Hz, 2H), 7.67 (td, *J* = 7.7, 1.4 Hz, 2H), 7.31 – 7.24 (m, 1H), 6.58 (dt, *J* = 14.8, 1.5 Hz, 1H), 2.32 – 2.24 (m, 2H), 2.13 (td, *J* = 6.8, 2.6 Hz, 2H), 1.90 (t, *J* = 2.7 Hz, 1H), 1.59 – 1.50 (m, 2H), 1.50 – 1.41 (m, 2H).

^{13}C NMR (126 MHz, $CDCl_3$) δ 156.4, 135.6, 134.6, 134.6, 133.6, 133.5, 130.3, 130.3, 120.3, 120.3, 109.7, 83.8, 68.9, 32.7, 27.6, 26.2, 18.0.

^{19}F NMR (471 MHz, $CDCl_3$) δ –150.59, –150.64

HRMS-ESI (m/z) calc'd for $C_{20}H_{19}S_2$ $[M]^+$, 323.0923; found, 323.0921; deviation: +0.4 ppm.

Alkenyl thianthrenium salt TT-8

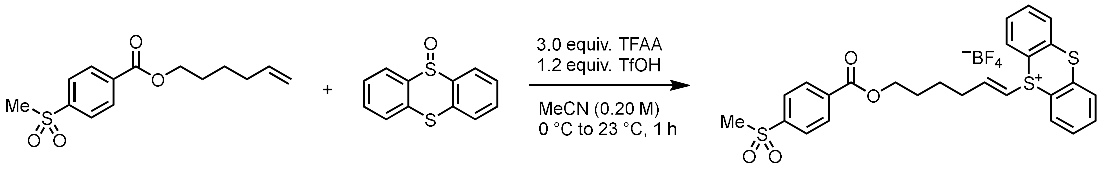

Alk-8, 2.0 mmol   1.0 equiv.   **TT-8**, 76% (*E*/*Z* = 20:1)

Under an ambient atmosphere, a 20 mL glass vial with a magnetic stirring bar was charged with **Alk-8** (0.56 g, 2.0 mmol, 1.0 equiv.), thianthrene-S-oxide (0.46 g, 2.0 mmol, 1.0 equiv.) and MeCN (8.0 mL, c = 0.20 M). The mixture was cooled to 0 °C using an ice bath. Trifluoroacetic anhydride (1.3 g, 0.83 mL 6.0 mmol, 3.0 equiv.) was added dropwise (2 drops per second) leading to a dark purple solution. Trifluoromethanesulfonic acid (0.36 g, 0.21 mL, 2.4 mmol, 1.2 eq.) was added dropwise (2 drops per second), and the mixture was stirred at 0 °C for 45 min followed by stirring at 25 °C for 15 min. The resulting mixture was diluted with DCM (10 mL) and was poured onto a saturated aqueous $NaHCO_3$ solution (20 mL). The layers were separated and the aqueous layer was further extracted with DCM (2 × 10 mL). The unified organic layer was washed with aqueous $NaBF_4$ solution (2 × 20 mL, 10% w/w). The organic layer was dried with Mg_2SO_4, filtered, and the solvent was removed under reduced pressure. The residue was purified by chromatography on silica gel eluting with pentane/EtOAc (3:1, v/v) → DCM/MeOH (19:1, v/v) to afford **TT-8** (930 mg, 76%, *E*/*Z* = 20:1) as a grey solid.

R*f* = 0.41 (DCM/MeOH = 19:1 (v/v)).

NMR Spectroscopy:

^{1}H NMR (500 MHz, $CDCl_3$) δ 8.39 – 8.29 (m, 2H), 8.23 – 8.14 (m, 2H), 8.04 – 7.95 (m, 2H), 7.85 – 7.77 (m, 2H), 7.73 (td, *J* = 7.7, 1.4 Hz, 2H), 7.69 – 7.60 (m, 2H), 7.34 – 7.26 (m, 1H), 6.60 (dd, *J* = 14.8, 1.5 Hz, 1H), 4.34 – 4.24 (m, 2H), 3.09 (s, 3H), 2.39 – 2.27 (m, 2H), 1.75 – 1.69 (m, 2H), 1.66 – 1.56 (m, 2H).

^{13}C NMR (126 MHz, $CDCl_3$) δ 165.1, 156.4, 144.3, 135.6, 135.0, 134.6, 133.8, 130.7, 130.5, 130.2, 127.6, 120.5, 110.0, 65.2, 44.4, 33.0, 28.0, 24.0.

^{19}F NMR (471 MHz, $CDCl_3$) δ –151.03, –151.09.

HRMS-ESI (m/z) calc'd for $C_{26}H_{25}O_4S_3$ $[M]^+$, 497.0910; found, 497.0914; deviation: –0.87 ppm.

Alkenyl thianthrenium salt TT-9

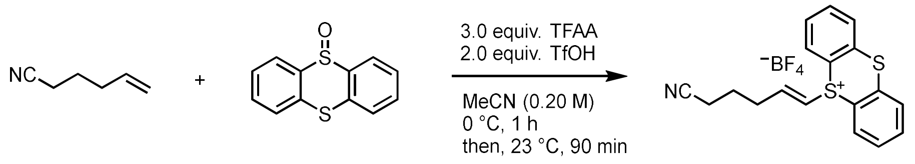

2.0 mmol 1.0 equiv. **TT-9**, 79% (*E*/*Z* = 8:1)

The reaction was carried out according to a previously reported procedure.[15] Under an ambient atmosphere, a 20 mL glass vial with a magnetic stirring bar was charged with 5-cyano-1-pentene (0.19 g, 0.23 mL, 2.0 mmol, 1.0 equiv.), thianthrene-S-oxide (0.46 g, 2.0 mmol, 1.0 equiv.) and MeCN (8.0 mL, c = 0.20 M). The mixture was cooled to 0 °C using an ice bath. Trifluoroacetic anhydride (1.3 g, 0.83 mL 6.0 mmol, 3.0 equiv.) was added dropwise (2 drops per second) leading to a dark purple solution. Trifluoromethanesulfonic acid (0.60 g, 0.35 mL, 2.0 mmol, 2.0 eq.) was added dropwise (2 drops per second), and the mixture was stirred at 0 °C for 60 min followed by stirring at 25 °C for 90 min. The resulting mixture was diluted with DCM (10 mL) and was poured onto a saturated aqueous $NaHCO_3$ solution (20 mL). The layers were separated and the aqueous layer was further extracted with DCM (2 × 10 mL). The unified organic layer was washed with aqueous $NaBF_4$ solution (2 × 20 mL, 10% w/w). The organic layer was dried with Na_2SO_4, filtered, and the solvent was removed under reduced pressure. The residue was purified by chromatography on silica gel eluting with pentane/EtOAc (3:1, v/v) → DCM/MeOH (19:1, v/v) to afford **TT-9** (629 mg, 79%, *E*/*Z* = 8:1) as a colorless solid.

R_f = 0.39 (DCM/MeOH = 19:1 (v/v)).

NMR Spectroscopy:

^{1}H NMR (500 MHz, $CDCl_3$) δ 8.33 (dd, *J* = 8.0 Hz, 1.4 Hz, 2H), 7.87 (dd, *J* = 7.9 Hz, 1.4 Hz, 2H), 7.78 (td,*J* = 7.6 Hz, 1.4 Hz, 2H), 7.70 (td, *J* = 7.6 Hz, 1.4 Hz, 2H), 7.23 (dt,*J* = 14.5 Hz, 7.1 Hz, 1H), 6.69 (dt, *J* = 14.8 Hz, 1.4 Hz, 1H), 2.47 (qd, *J* = 7.2 Hz, 1.5 Hz, 2H), 2.41 (t, *J* = 7.0 Hz, 2 H), 1.86 (p, 7.1 Hz, 2H).

^{13}C NMR (126 MHz, $CDCl_3$) δ 154.4, 135.7, 134.7, 133.8, 130.5, 130.3, 120.3, 119.3, 111.3, 31.8, 23.1, 16.3

^{19}F NMR (471 MHz, $CDCl_3$) δ –151.03, –151.04

HRMS-ESI (m/z) calc'd for $C_{18}H_{16}N_1S_2$ $[M]^+$, 310.0719; found 310.0719; deviation: ±0.0 ppm.

Alkenyl thianthrenium salt (±)-TT-12

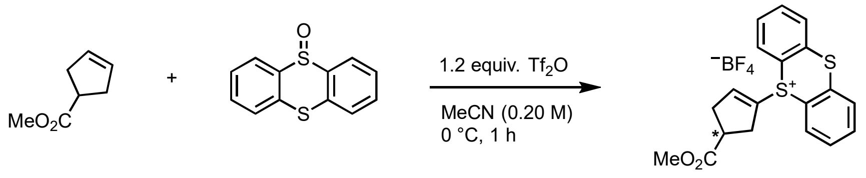

Alk-12, 2.0 mmol 1.0 equiv. (±)-**TT-12**, 80%

Under an ambient atmosphere, a 20 mL glass vial with a Teflon-coated magnetic stirring bar was charged with **Alk-12** (0.25 g, 2.0 mmol, 1.0 equiv.), thianthrene-S-oxide (0.46 g, 2.0 mmol, 1.0 equiv.) and MeCN (8.0 ml, c = 0.25 M). The mixture was cooled to 0 °C using an ice bath. Under stirring, triflic anhydride (0.68 g, 0.40 mL, 2.4 mmol, 1.2 equiv.) was added dropwise (2 drops per second) leading to a dark purple solution. The mixture was stirred at 0 °C for 1 h. Then, the mixture was diluted with DCM (10 mL) and was poured onto a saturated aqueous $NaHCO_3$ solution (20 mL). The layers were separated and the aqueous layer was further extracted with DCM (2 × 5 mL). The unified organic layer was washed with aqueous $NaBF_4$ solution (2 × 30 mL, 10% w/w). The organic layer was dried with Na_2SO_4, filtered, and the solvent was removed under reduced pressure. The residue was purified by chromatography on silica gel eluting with pentane/EtOAc (4:1, v/v) → DCM/MeOH (50:1, v/v) to afford (±)-**TT-12** (685 mg, 80%) as a colorless sticky oil.

$\mathbf{R}_f$ = 0.41 (DCM/MeOH = 19:1 (v/v)).

NMR Spectroscopy:

^{1}**H NMR** (500 MHz, $CDCl_3$) δ 8.44 – 8.34 (m, 2H), 7.84 – 7.73 (m, 4H), 7.73 – 7.67 (m, 2H), 6.32 – 6.24 (m, 1H), 3.62 (s, 3H), 3.38 – 3.27 (m, 1H), 2.92 – 2.80 (m, 2H), 2.80 – 2.71 (m, 1H), 2.65 – 2.55 (m, 1H).

^{13}**C NMR** (126 MHz, $CDCl_3$) δ 173.4, 145.7, 136.1, 136.1, 135.0, 134.9, 134.2, 134.2, 130.2, 130.1, 130.0, 123.0, 117.1, 116.7, 52.3, 41.6, 36.8, 35.2.

^{19}**F NMR** (471 MHz, $CDCl_3$) δ –151.06 – –151.12 (m), –151.16.

HRMS-ESI (m/z) calc'd for $C_{19}H_{17}O_2S_2$ $[M]^+$, 341.0664; found 341.0662; deviation: +0.8 ppm.

Alkenyl thianthrenium salt TT-13

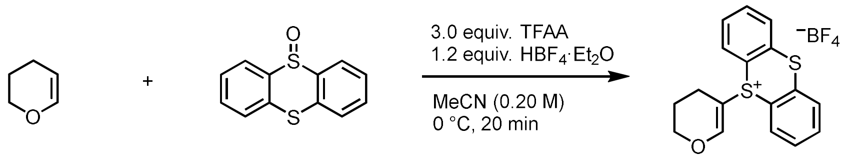

2.0 mmol 1.0 equiv. **TT-13**, 47%

The reaction was carried out according to a previously reported procedure.[11] Under an ambient atmosphere, a 20 mL glass vial with a magnetic stirring bar was charged with 3,4-dihydro-2H-pyrane (0.17 g, 2.0 mmol,

1.0 equiv.), thianthrene-S-oxide (0.46 g, 2.0 mmol, 1.0 equiv.) and MeCN (8.0 mL, c = 0.20 M). The mixture was cooled to 0 °C using an ice bath. Trifluoroacetic anhydride (1.3 g, 0.83 mL 6.0 mmol, 3.0 equiv.) was added dropwise (2 drops per second) leading to a dark purple solution. $HBF_4 \cdot Et_2O$ (0.39 g, 0.33 mL, 2.4 mmol, 1.2 eq.) was added dropwise (2 drops per second), and the mixture was stirred at 0 °C for 20 min. The resulting mixture was diluted with DCM (10 mL) and was poured onto a saturated aqueous $NaHCO_3$ solution (20 mL). The layers were separated and the aqueous layer was further extracted with DCM (2 × 10 mL). The unified organic layer was washed with aqueous $NaBF_4$ solution (2 × 20 mL, 10% w/w). The organic layer was dried with Na_2SO_4, filtered, and the solvent was removed under reduced pressure. The residue was purified by chromatography on silica gel eluting with pentane/EtOAc (3:1, v/v) → DCM/MeOH (19:1, v/v) to afford **TT-13** (363 mg, 47%) as a faint yellow solid.

R*f* = 0.46 (DCM/MeOH = 19:1 (v/v)).

NMR Spectroscopy:

^{1}H NMR (500 MHz, $CDCl_3$) δ 8.19 – 8.15 (m, 2H), 7.87 (s, 1H), 7.72 – 7.68 (m, 4H), 7.66 (ddd, *J* = 8.6, 5.5, 3.2 Hz, 2H), 4.18 – 4.11 (m, 2H), 2.20 (t, *J* = 6.3 Hz, 2H), 1.98 – 1.89 (m, 2H).

^{13}C NMR (126 MHz, $CDCl_3$) δ 159.6, 135.5, 134.1, 133.1, 130.4, 129.3, 118.5, 102.3, 67.5, 21.4, 19.7.

^{19}F NMR (471 MHz, $CDCl_3$) δ –151.10, –151.15.

HRMS-ESI (m/z) calc'd for $C_{17}H_{15}O_1S_2$ $[M]^+$, 299.0559; found 299.0558; deviation: +0.3 ppm.

The obtained data are consistent with those previously reported in the literature.[11]

Alkenyl thianthrenium salt TT-14

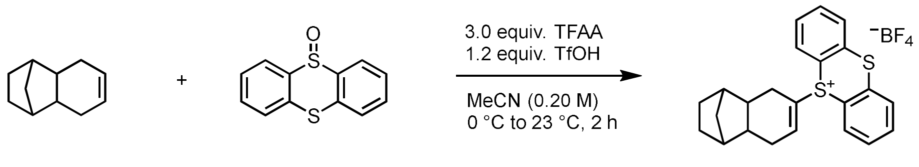

2.0 mmol 1.0 equiv. **TT-14**, 52%

The reaction was carried out according to a previously reported procedure.[11] Under an ambient atmosphere, a 20 mL glass vial with a magnetic stirring bar was charged with tricyclo[6.2.1.0(2,7)]undeca-4-ene (0.30 g, 0.30 mL, 2.0 mmol, 1.0 equiv.), thianthrene-S-oxide (0.46 g, 2.0 mmol, 1.0 equiv.) and MeCN (8.0 mL, c = 0.20 M). The mixture was cooled to 0 °C using an ice bath. Trifluoroacetic anhydride (1.3 g, 0.83 mL 6.0 mmol, 3.0 equiv.) was added dropwise (2 drops per second) leading to a dark purple solution. Triflic acid (0.36 g, 0.21 mL, 2.4 mmol, 1.2 eq.) was added dropwise (2 drops per second), and the mixture was stirred at 0 °C for 1 h followed by stirring at 23 °C for 1 h. The resulting mixture was diluted with DCM (10 mL) and was poured onto a saturated aqueous $NaHCO_3$ solution (20 mL). The layers were separated and the aqueous layer was further extracted with DCM (2 × 10 mL). The unified organic layer was washed with aqueous $NaBF_4$ solution (2 × 20 mL, 10% w/w). The organic layer was dried with Na_2SO_4, filtered, and the solvent was removed under reduced pressure. The residue was purified by chromatography on silica gel

eluting with pentane/EtOAc (3:1, v/v) → DCM/MeOH (19:1, v/v) to afford **TT-14** (469.9 mg, 52%) as a colorless solid.

R_f = 0.46 (DCM/MeOH = 19:1 (v/v)).

NMR Spectroscopy:

^{1}H NMR (500 MHz, $CDCl_3$) δ 8.39 – 8.28 (m, 2H), 7.87 – 7.75 (m, 4H), 7.75 – 7.64 (m, 2H), 6.03 – 5.92 (m, 1H), 2.50 – 2.37 (m, 1H), 2.28 – 2.19 (m, 1H), 1.87 – 1.83 (m, 1H), 1.64 – 1.52 (m, 4H), 1.49 – 1.38 (m, 3H), 1.16 – 1.06 (m, 2H), 0.99 (d, J = 10.4 Hz, 1H).

^{13}C NMR (126 MHz, $CDCl_3$) δ 141.7, 136.2, 135.6, 135.3, 134.7, 134.6, 130.2, 130.2, 130.0, 129.9, 121.5, 117.6, 117.5, 43.8, 43.1, 42.9, 42.2, 33.4, 30.7, 29.3, 29.2, 29.1.

^{19}F NMR (471 MHz, $CDCl_3$) δ –151.20, –151.26.

HRMS-ESI (m/z) calc'd for $C_{23}H_{23}S_2$ $[M]^+$,363.1236; found 363.1237; deviation: –0.3 ppm.

The obtained data are consistent with those previously reported in the literature.[11]

Alkenyl thianthrenium salt TT-15

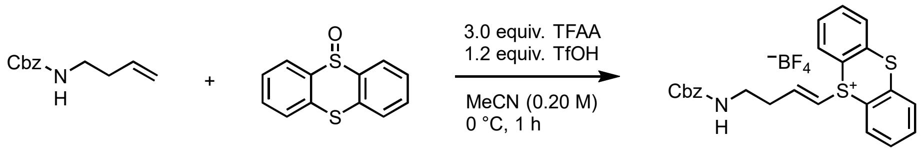

Alk-15, 2.0 mmol 1.0 equiv. **TT-15**, 42% (E/Z > 20:1)

Under an ambient atmosphere, a 20 mL glass vial with a magnetic stirring bar was charged with **Alk-15** (0.41 g, 2.0 mmol, 1.0 equiv.), thianthrene-S-oxide (0.46 g, 2.0 mmol, 1.0 equiv.) and MeCN (8.0 mL, c = 0.20 M). The mixture was cooled to 0 °C using an ice bath. Trifluoroacetic anhydride (1.3 g, 0.83 mL 6.0 mmol, 3.0 equiv.) was added dropwise (2 drops per second) leading to a dark purple solution. Triflic acid (0.36 mg, 0.21 mL, 2.4 mmol, 1.2 eq.) was added dropwise (2 drops per second), and the mixture was stirred at 0 °C for 60 min. The resulting mixture was diluted with DCM (10 mL) and was poured onto a saturated aqueous $NaHCO_3$ solution (20 mL). The layers were separated and the aqueous layer was further extracted with DCM (2 × 10 mL). The unified organic layer was washed with aqueous $NaBF_4$ solution (2 × 20 mL, 10% w/w). The organic layer was dried with Na_2SO_4, filtered, and the solvent was removed under reduced pressure. The residue was purified by chromatography on silica gel eluting with pentane/EtOAc (3:1, v/v) → DCM/MeOH (19:1, v/v) to afford **TT-15** (421 mg, 42%, E/Z > 20:1) as a colorless sticky oil.

R_f = 0.46 (DCM/MeOH = 19:1 (v/v)).

NMR Spectroscopy:

^{1}H NMR (500 MHz, $CDCl_3$) δ 8.28 – 8.19 (m, 2H), 7.78 (d, J = 7.8 Hz, 2H), 7.68 (t, J = 7.6 Hz, 2H), 7.65 – 7.58 (m, 2H), 7.27 – 7.17 (m, 5H), 6.58 (d, J = 14.8 Hz, 1H), 5.74 (t, J = 6.2 Hz, 1H), 4.95 (s, 2H), 3.38 – 3.25 (m, 2H), 2.49 (q, J = 6.6 Hz, 2H), 1.88 (s, 1H).

^{13}C NMR (126 MHz, $CDCl_3$) δ 156.8, 154.9, 136.8, 135.5, 134.5, 134.4, 133.6, 133.5, 130.4, 130.4, 130.2, 130.2, 128.5, 128.0, 127.8, 120.7, 120.6, 110.9, 66.4, 38.8, 34.0.

^{19}F NMR (471 MHz, $CDCl_3$) δ –150.23, –150.29.

HRMS-ESI (m/z) calc'd for $C_{24}H_{22}N_1O_2S_2$ $[M]^+$, 420.1087; found 420.1084; deviation: +0.7 ppm.

Alkenyl thianthrenium salt TT-16

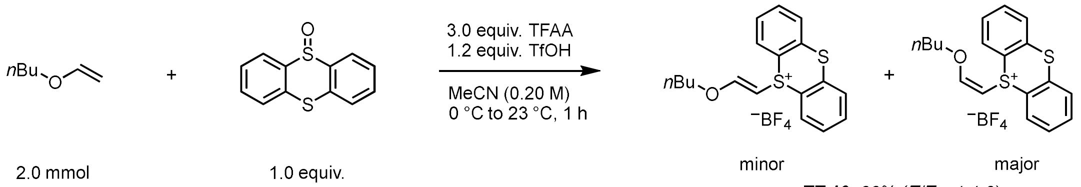

TT-16, 66% (*E*/*Z* = 1:1.6)

Under an ambient atmosphere, a 20 mL glass vial with a magnetic stirring bar was charged with *n*-butylvinylether (0.20 g, 0.26 mL, 2.0 mmol, 1.0 equiv.), thianthrene-S-oxide (0.46 g, 2.0 mmol, 1.0 equiv.) and MeCN (8.0 mL, c = 0.20 M). The mixture was cooled to 0 °C using an ice bath. Trifluoroacetic anhydride (1.3 g, 0.83 mL 6.0 mmol, 3.0 equiv.) was added dropwise (2 drops per second) leading to a dark purple solution. Trifluoromethanesulfonic acid (360 mg, 0.21 mL, 2.4 mmol, 1.2 eq.) was added dropwise (2 drops per second), and the mixture was stirred at 0 °C for 60 min. The resulting mixture was diluted with DCM (10 mL) and was poured onto a saturated aqueous $NaHCO_3$ solution (20 mL). The layers were separated and the aqueous layer was further extracted with DCM (2 × 10 mL). The unified organic layer was washed with aqueous $NaBF_4$ solution (2 × 20 mL, 10% w/w). The organic layer was dried with Mg_2SO_4, filtered, and the solvent was removed under reduced pressure. The residue was purified by chromatography on silica gel eluting with EtOAc (100%) → DCM/MeOH (19:1, v/v) to afford **TT-16** (527 mg, 66%, *E*/*Z* = 1:1.6) as a sticky orange oil.

R_f = 0.40 (DCM/MeOH = 19:1 (v/v)).

NMR Spectroscopy:

Z-isomer :

^{1}H NMR (600 MHz, $CDCl_3$) δ 8.04 – 8.01 (m, 2H), 7.83 – 7.80 (m, 2H), 7.69 (td, *J* = 7.7, 1.4 Hz, 2H), 7.70 – 7.64 (m, 1H), 7.65 – 7.61 (m, 2H), 7.63 (d, *J* = 4.8 Hz, 1H), 5.59 (d, *J* = 4.7 Hz, 1H), 4.25 (t, *J* = 6.6 Hz, 2H), 1.71 – 1.64 (m, 2H), 1.32 – 1.28 (m, 2H), 0.89 (t, *J* = 7.4 Hz, 3H).

^{13}C NMR (151 MHz, $CDCl_3$) δ 166.8, 134.8, 133.8, 130.8, 130.6, 130.1, 123.2, 80.7, 77.4, 31.5, 18.6, 13.7.

E-isomer :

^{1}H NMR (600 MHz, $CDCl_3$) δ 8.20 (ddd, *J* = 7.9, 1.4, 0.4 Hz, 2H), 8.16 (d, *J* = 12.1 Hz, 1H), 7.80 – 7.76 (m, 2H), 7.70 – 7.64 (m, 2H), 7.60 (ddd, *J* = 7.9, 7.5, 1.3 Hz, 2H), 6.03 (d, *J* = 12.1 Hz, 1H), 4.08 (t, *J* = 6.5 Hz, 2H), 1.61 (ddt, *J* = 9.2, 7.8, 6.4 Hz, 2H), 1.32 – 1.28 (m, 2H), 0.85 (t, *J* = 7.4 Hz, 3H).

^{13}C NMR (151 MHz, $CDCl_3$) δ 168.5, 135.6, 133.8, 132.1, 130.2, 130.2, 123.5, 84.0, 75.1, 31.0, 18.7, 13.6.

^{19}F NMR (471 MHz, $CDCl_3$) δ –151.57, –151.62.

HRMS-ESI (m/z) calc'd for $C_{18}H_{19}O_1S_2$ $[M]^+$, 315.0872; found, 315.0870; deviation: +0.7 ppm.

Alkenyl thianthrenium salt TT-18

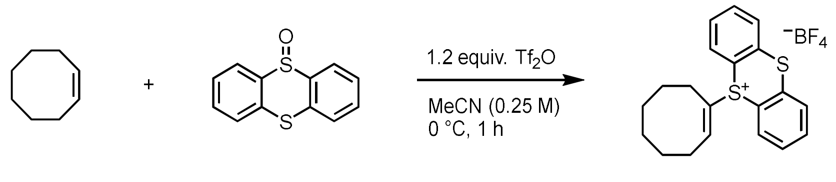

The reaction was carried out according to a previously reported procedure[11] that was modified as follows: Under an ambient atmosphere, a 20 mL glass vial with a Teflon-coated magnetic stirring bar was charged with cyclooctene (0.23 g, 2.0 mmol, 1.0 equiv.), thianthrene-S-oxide (0.46 g, 2.0 mmol, 1.0 equiv.) and MeCN (4.0 ml, c = 0.25 M). Under stirring, triflic anhydride (0.68 g, 0.40 mL, 2.4 mmol, 1.2 equiv.) was added dropwise (2 drops per second) leading to a dark purple solution. The mixture was stirred at 0 °C for 1 h. Then, the mixture was diluted with DCM (5 mL) and was poured onto a saturated aqueous $NaHCO_3$ solution (10 mL). The layers were separated and the aqueous layer was further extracted with DCM (2 × 5 mL). The unified organic layer was washed with aqueous $NaBF_4$ solution (2 × 20 mL, 10% w/w). The organic layer was dried with Na_2SO_4, filtered, and the solvent was removed under reduced pressure. The residue was purified by chromatography on silica gel eluting with pentane/EtOAc (4:1, v/v) → DCM/MeOH (50:1, v/v) to afford a yellow oil, which was dissolved in DCM (4 mL) and precipitated from Et_2O (50 mL) to afford **TT-18** (398 mg, 48%) as a white solid.

R_f = 0.40 (DCM/MeOH = 19:1 (v/v)).

NMR Spectroscopy:

^{1}H NMR (500 MHz, $CDCl_3$) δ 8.41 – 8.31 (m, 2H), 7.86 – 7.75 (m, 4H), 7.75 – 7.66 (m, 2H), 5.78 (t, *J* = 8.4 Hz, 1H), 2.56 – 2.45 (m, 2H), 2.29 – 2.18 (m, 2H), 1.60 – 1.49 (m, 2H), 1.41 – 1.27 (m, 4H), 1.04 – 0.93 (m, 2H).

^{13}C NMR (126 MHz, $CDCl_3$) δ 142.6, 136.6, 135.5, 134.9, 130.3, 129.9, 124.0, 116.9, 29.1, 28.2, 28.0, 27.9, 25.9, 25.2.

^{19}F NMR (471 MHz, $CDCl_3$) δ –151.56, –151.61.

HRMS-ESI (m/z) calc'd for $C_{20}H_{21}S_2$ $[M]^+$, 325.1079; found 325.1080; deviation: –0.3 ppm.

The obtained data are consistent with those previously reported in the literature.[11]

Alkenyl thianthrenium salt TT-21

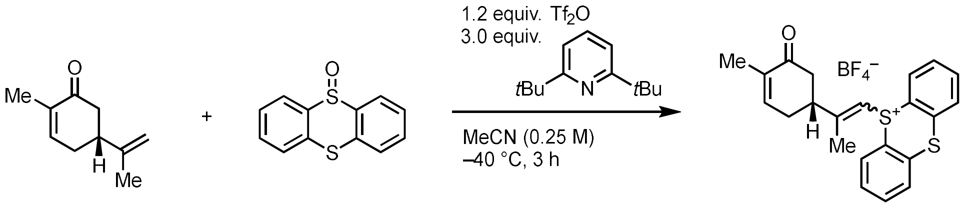

2.0 mmol 1.0 equiv. **TT-21**, 37% (*E*/*Z* = 7:1)

Under an ambient atmosphere, a 20 mL glass vial with a magnetic stirring bar was charged with *L*-(+)-carvone (0.47 mL, 0.45 g, 3.0 mmol, 1.0 equiv.), thianthrene-S-oxide (0.70 g, 3.0 mmol, 1.0 equiv.) and MeCN (12.0 mL, c = 0.25 M). The mixture was cooled to –40 °C using a cryocooler. Under stirring, 2,6-di-*tert*-butylpyridine (2.0 mL, 1.7 g, 9.0 mmol, 3.0 equiv.) was added in one shot, followed by the dropwise (2 drops per second) addition of triflic anhydride (1.0 g, 0.61 mL 3.6 mmol, 1.2 equiv.) was added dropwise (2 drops per second) leading to a dark purple solution. The mixture was stirred at –40 °C for 3 h. The resulting mixture was diluted with DCM (10 mL) and was poured onto a saturated aqueous $NaHCO_3$ solution (20 mL). The layers were separated and the aqueous layer was further extracted with DCM (2 × 10 mL). The unified organic layer was washed with aqueous $NaBF_4$ solution (2 × 30 mL, 10% w/w). The organic layer was dried with Na_2SO_4, filtered, and the solvent was removed under reduced pressure. The residue was purified by chromatography on silica gel eluting with pentane/EtOAc (3:1, v/v) → EtOAc (100%) → DCM/*i*PrOH (100:2, v/v) to afford a yellow oil. A 100 mL round-bottom flask with a magnetic stir bar was charged with diethylether (50 mL), the yellow oil obtained in the previous step was dissolved in DCM (5 mL), and added dropwise to the round-bottom flask, resulting in the precipitation of a beige solid. The solid was collected and dried in vacuo to afford **TT-21** (503 mg, 37%, *E*/*Z* = 7:1) as a beige solid.

R_f = 0.54 (DCM/*i*PrOH = 19:1 (v/v)).

NMR Spectroscopy:

^{1}H NMR (500 MHz, $CDCl_3$) δ 8.30 (dd, *J* = 8.0, 1.4 Hz, 2H), 7.84 (dd, *J* = 7.9, 1.3 Hz, 2H), 7.72 (td, *J* = 7.7, 1.4 Hz, 2H), 7.65 (td, *J* = 7.7, 1.4 Hz, 2H), 6.68 – 6.62 (m, 1H), 6.44 – 6.40 (m, 1H), 2.98 – 2.82 (m, 1H), 2.52 – 2.40 (m, 3H), 2.40 – 2.36 (m, 3H), 2.36 – 2.27 (m, 3H), 1.70 – 1.63 (m, 3H).

^{13}C NMR (126 MHz, $CDCl_3$) δ 197.0, 166.4, 143.3, 135.8, 135.6, 134.4, 133.5, 130.5, 130.5, 121.6, 106.9, 44.5, 41.5, 29.9, 18.2, 15.6.

^{19}F NMR (471 MHz, $CDCl_3$) δ –149.81 – –149.96 (m).

HRMS-ESI (m/z) calc'd for $C_{22}H_{21}O_1S_2$ $[M]^+$, 365.1028; found 365.1027; deviation: +0.3 ppm.

Alkenyl thianthrenium salt TT-22

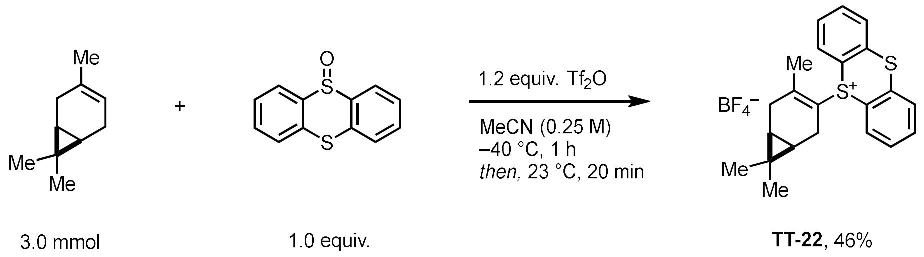

Under an ambient atmosphere, a 20 mL glass vial with a magnetic stirring bar was charged with 3-carene (0.48 mL, 0.41 g, 3.0 mmol, 1.0 equiv.), thianthrene-S-oxide (0.70 g, 3.0 mmol, 1.0 equiv.) and MeCN (12.0 mL, c = 0.25 M). The mixture was cooled to –40 °C using an acetone/dry ice bath. Under stirring, triflic anhydride (1.0 g, 0.61 mL 3.6 mmol, 1.2 equiv.) was added dropwise (2 drops per second) leading to a dark purple solution. The mixture was stirred at –40 °C for 1 h. Then, the cooling bath was removed, and the mixture was stirred for 20 min. The resulting mixture was diluted with DCM (10 mL) and was poured onto a saturated aqueous $NaHCO_3$ solution (20 mL). The layers were separated and the aqueous layer was further extracted with DCM (2 × 10 mL). The unified organic layer was washed with aqueous $NaBF_4$ solution (2 × 30 mL, 10% w/w). The organic layer was dried with Na_2SO_4, filtered, and the solvent was removed under reduced pressure. The residue was purified by chromatography on silica gel eluting with pentane/EtOAc (3:1, v/v) → EtOAc (100%) → DCM/*i*PrOH (100:2, v/v) to afford a yellow oil. A 100 mL round-bottom flask with a magnetic stir bar was charged with diethylether (50 mL), the yellow oil obtained in the previous step was dissolved in DCM (5 mL), and added dropwise to the round-bottom flask, resulting in the precipitation of a yellow solid. The solid was collected and dried in vacuo to afford **TT-22** (605 mg, 46%) as a yellow solid.

$\mathbf{R_f}$ = 0.51 (DCM/*i*PrOH = 19:1 (v/v)).

NMR Spectroscopy:

^{1}H NMR (500 MHz, $CDCl_3$) δ 8.06 (dd, *J* = 7.9, 1.3 Hz, 1H), 8.01 (dd, *J* = 7.9, 1.3 Hz, 1H), 7.75 – 7.69 (m, 2H), 7.69 – 7.63 (m, 3H), 7.63 – 7.57 (m, 1H), 2.77 – 2.62 (m, 1H), 2.56 – 2.48 (m, 3H), 2.48 – 2.41 (m, 1H), 2.36 – 2.22 (m, 1H), 2.14 – 2.04 (m, 1H), 0.91 (s, 3H), 0.80 – 0.67 (m, 2H), 0.44 (s, 3H).

^{13}C NMR (126 MHz, $CDCl_3$) δ 155.8, 135.8, 135.5, 134.4, 134.4, 133.9, 133.5, 130.3, 130.2, 129.2, 129.1, 124.0, 116.0, 115.8, 31.3, 27.5, 22.4, 21.4, 18.1, 17.9, 17.2, 13.2.

^{19}F NMR (471 MHz, $CDCl_3$) δ –152.34, –152.39.

HRMS-ESI (m/z) calc'd for $C_{22}H_{23}S_2$ $[M]^+$, 351.1236; found 351.1237; deviation: –0.3 ppm.

Alkenyl thianthrenium salt TT-23

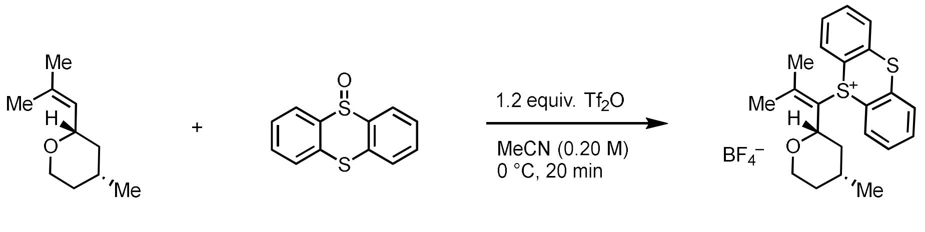

2.0 mmol 1.0 equiv. **TT-23**, 61%

The reaction was carried out according to a previously published procedure that was modified as follows[11]: Under an ambient atmosphere, a 20 mL glass vial with a magnetic stirring bar was charged with *cis*-(-)-rose oxide (0.36 mL, 0.31 g, 2.0 mmol, 1.0 equiv.), thianthrene-S-oxide (0.46 g, 2.0 mmol, 1.0 equiv.) and MeCN (8.0 mL, c = 0.25 M). The mixture was cooled to 0 °C using an ice bath. Under stirring, triflic anhydride (1.0 g, 0.61 mL 3.6 mmol, 1.2 equiv.) was added dropwise (2 drops per second) leading to a dark purple solution. The mixture was stirred at 0 °C for 20 min. The resulting mixture was diluted with DCM (10 mL) and was poured onto a saturated aqueous $NaHCO_3$ solution (20 mL). The layers were separated and the aqueous layer was further extracted with DCM (2 × 10 mL). The unified organic layer was washed with aqueous $NaBF_4$ solution (2 × 30 mL, 10% w/w). The organic layer was dried with Na_2SO_4, filtered, and the solvent was removed under reduced pressure. The residue was purified by chromatography on silica gel eluting with pentane/EtOAc (3:1, v/v) → EtOAc (100%) → DCM/*i*PrOH (100:2, v/v) to afford a yellow oil. A 100 mL round-bottom flask with a magnetic stir bar was charged with diethylether (50 mL), the colorless oil obtained in the previous step was dissolved in DCM (5 mL), and added dropwise to the round-bottom flask, resulting in the precipitation of a colorless solid. The solid was collected and dried in vacuo to afford **TT-23** (552 mg, 61%) as a colorless solid.

$\mathbf{R}_f$ = 0.51 (DCM/*i*PrOH = 19:1 (v/v)).

NMR Spectroscopy:

^{1}H NMR (500 MHz, $CDCl_3$) δ 7.93 – 7.87 (m, 1H), 7.83 – 7.77 (m, 2H), 7.77 – 7.71 (m, 2H), 7.71 – 7.62 (m, 3H), 4.83 (dd, *J* = 11.7, 2.4 Hz, 1H), 4.02 – 3.92 (m, 1H), 3.54 (td, *J* = 12.1, 2.2 Hz, 1H), 2.42 (s, 3H), 2.10 – 1.99 (m, 1H), 1.76 (s, 4H), 1.57 – 1.49 (m, 1H), 1.17 – 1.04 (m, 1H), 1.04 – 0.93 (m, 1H), 0.91 (d, *J* = 6.5 Hz, 3H).

^{13}C NMR (126 MHz, $CDCl_3$) δ 164.4, 133.3, 133.2, 131.4, 131.3, 130.4, 130.4, 129.7, 129.7, 128.9, 128.8, 124.0, 123.7, 119.2, 75.2, 68.6, 38.9, 33.6, 29.8, 26.7, 24.5, 22.0.

^{19}F NMR (471 MHz, $CDCl_3$) δ –152.90, –152.95.

HRMS-ESI (m/z) calc'd for $C_{22}H_{25}O_1S_2$ $[M]^+$, 369.1341; found 369.1340; deviation: +0.4 ppm.

Syntheses of 1,3-dienes

1,3-Diene 1a

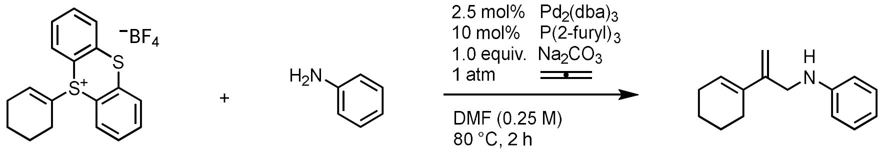

TT-1, 0.300 mmol   1.0 equiv.   **1a**, 75%

Caution: Propadiene/allene is a flammable gas and should be handled with appropriate care. Guidelines regarding the safe handling of flammable gases can be found in "Prudent Practices in the Laboratory" (Chapter 4.D).[2]

A 4 mL borosilicate vial equipped with a Teflon-coated magnetic stirring bar was charged with $Pd_2(dba)_3$ (6.9 mg, 7.5 μmol, 2.5 mol%), tri(2-furyl)phosphine (7.0 mg, 30 μmol, 10 mol%), alkenyl thianthrenium salt **TT-1** (115 mg, 0.300 mmol, 1.0 equiv.), Na_2CO_3 (31.8 mg, 0.300 mmol, 1.0 equiv.) and aniline (27.9 mg, 27.3 μL, 0.300 mmol, 1.0 equiv.). Dry DMF (1.2 mL, c = 0.25 M) was added and the vial was sealed with a septum cap. The septum was pierced with an outlet needle (Φ 0.80 × 40 mm) and another needle (Φ 0.80 × 120 mm), which was connected to an allene-containing balloon. A gentle stream of allene gas was passed through the mixture for 2 min. Both needles were removed and the septum cap was quickly wrapped with parafilm. The vial was transferred to a heating block, which had been preheated at 80 °C, and the reaction mixture was stirred at 1000 rpm for 2 h. Then, the vial was removed from the heating block, the stirring bar was removed, and the solvent evaporated using a Biotage V10. The residue was purified by chromatography on silica gel eluting with pentane/EtOAc (80:1 → 50:1 → 30:1, v/v) to afford **1a** (47.9 mg, 75%) as a brown oil.

R*f* = 0.55 (pentane/EtOAc = 19:1 (v/v), $KMnO_4$).

NMR Spectroscopy:

^{1}H NMR (500 MHz, $CDCl_3$) δ 7.23 – 7.13 (m, 2H), 6.71 (td, *J* = 7.3, 1.2 Hz, 1H), 6.65 – 6.56 (m, 2H), 5.97 (t, *J* = 4.2 Hz, 1H), 5.12 (d, *J* = 14.8 Hz, 2H), 3.94 (s, 2H), 3.80 (br s, 1H), 2.28 – 2.20 (m, 2H), 2.20 – 2.13 (m, 2H), 1.72 (qd, *J* = 7.7, 3.8 Hz, 2H), 1.68 – 1.56 (m, 2H).

^{13}C NMR (126 MHz, $CDCl_3$) δ 148.4, 144.8, 134.8, 129.3, 125.2, 117.3, 112.8, 110.6, 47.1, 26.1, 26.0, 22.9, 22.3.

HRMS-EI (m/z) calc'd for $C_{15}H_{19}N_1$ $[M]^+$, 213.1512; found, 213.1513; deviation: –0.3 ppm.

The obtained data are consistent with those previously reported in the literature.[3]

1,3-Diene 2

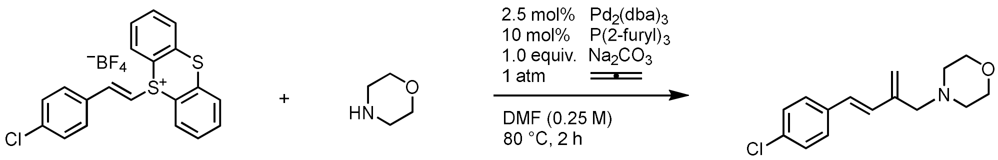

TT-2, 0.300 mmol (*E*/*Z* > 20:1) — 1.0 equiv. — **2**, 75% (*E*/*Z* > 20:1)

Caution: Propadiene/allene is a flammable gas and should be handled with appropriate care. Guidelines regarding the safe handling of flammable gases can be found in "Prudent Practices in the Laboratory" (Chapter 4.D).[2]

A 4 mL borosilicate vial equipped with a Teflon-coated magnetic stirring bar was charged with $Pd_2(dba)_3$ (6.9 mg, 7.5 µmol, 2.5 mol%), tri(2-furyl)phosphine (7.0 mg, 30 µmol, 10 mol%), alkenyl thianthrenium salt **TT-2** (132 mg, 0.300 mmol, 1.0 equiv.), Na_2CO_3 (31.8 mg, 0.300 mmol, 1.0 equiv.) and morpholine (26.1 mg, 26.2 µL, 0.300 mmol, 1.0 equiv.). Dry DMF (1.2 mL, c = 0.25 M) was added and the vial was sealed with a septum cap. The septum was pierced with an outlet needle (Φ 0.80 × 40 mm) and another needle (Φ 0.80 × 120 mm), which was connected to an allene-containing balloon. A gentle stream of allene gas was passed through the mixture for 2 min. Both needles were removed and the septum cap was quickly wrapped with parafilm. The vial was transferred to a heating block, which had been preheated at 80 °C, and the reaction mixture was stirred at 1000 rpm for 2 h. Then, the vial was removed from the heating block, the stirring bar was removed, and the solvent evaporated using a Biotage V10. The residue was purified by chromatography on silica gel eluting with hexanes/EtOAc (20:1 → 10:1, v/v) to afford **2** (59.4 mg, 75%, *E*/*Z* > 20:1) as a faint yellow solid.

$\mathbf{R}_f$ = 0.15 (Hex/EtOAc = 10:1 (v/v), $KMnO_4$).

NMR Spectroscopy:

^{1}**H NMR** (500 MHz, $CDCl_3$) δ 7.35 (d, *J* = 8.5 Hz, 2H), 7.31 – 7.27 (m, 2H), 6.85 (d, *J* = 16.2 Hz, 1H), 6.76 (d, *J* = 16.2 Hz, 1H), 5.29 (d, *J* = 2.0 Hz, 1H), 5.24 (d, *J* = 1.9 Hz, 1H), 3.71 (t, *J* = 4.6 Hz, 4H), 3.20 (s, 2H), 2.53 – 2.38 (m, 4H).

^{13}**C NMR** (126 MHz, $CDCl_3$) δ 141.7, 136.1, 133.1, 130.3, 128.9, 128.1, 127.8, 119.2, 67.2, 61.5, 53.8.

HRMS-EI (m/z) calc'd for $C_{15}H_{18}N_1O_1Cl_1$ $[M]^+$, 263.1071; found, 263.1072; deviation: –0.2 ppm.

1,3-Diene 3

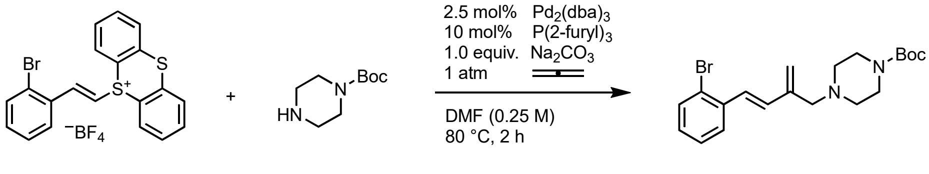

TT-3, 0.300 mmol (*E*/*Z* > 20:1) — 1.0 equiv. — **3**, 95% (*E*/*Z* > 20:1)

Caution: Propadiene/allene is a flammable gas and should be handled with appropriate care. Guidelines regarding the safe handling of flammable gases can be found in “Prudent Practices in the Laboratory” (Chapter 4.D).[2]

A 4 mL borosilicate vial equipped with a Teflon-coated magnetic stirring bar was charged with $Pd_2(dba)_3$ (6.9 mg, 7.5 µmol, 2.5 mol%), tri(2-furyl)phosphine (7.0 mg, 30 µmol, 10 mol%), alkenyl thianthrenium salt **TT-3** (136 mg, 0.300 mmol, 1.0 equiv.), Na_2CO_3 (31.8 mg, 0.300 mmol, 1.0 equiv.) and *N-tert*-butoxycarbonylpiperazine (55.9 mg, 0.300 mmol, 1.0 equiv.). Dry DMF (1.2 mL, c = 0.25 M) was added and the vial was sealed with a septum cap. The septum was pierced with an outlet needle (Φ 0.80 × 40 mm) and another needle (Φ 0.80 × 120 mm), which was connected to an allene-containing balloon. A gentle stream of allene gas was passed through the mixture for 2 min. Both needles were removed and the septum cap was quickly wrapped with parafilm. The vial was transferred to a heating block, which had been preheated at 80 °C, and the reaction mixture was stirred at 1000 rpm for 2 h. Then, the vial was removed from the heating block, the stirring bar was removed, and the solvent evaporated using a Biotage V10. The residue was purified by chromatography on silica gel eluting with hexanes/EtOAc (30:1 → 20:1 → 10:1, v/v) to afford **3** (116 mg, 95%, *E*/*Z* > 20:1) as a colorless oil.

$\mathbf{R}_f$ = 0.24 (Hex/EtOAc = 9:1 (v/v)).

NMR Spectroscopy:

^{1}**H NMR** (500 MHz, $CDCl_3$) δ 7.59 – 7.51 (m, 2H), 7.38 (d, *J* = 16.1 Hz, 1H), 7.30 – 7.25 (m, 1H), 7.12 – 7.05 (m, 1H), 6.71 (d, *J* = 16.1 Hz, 1H), 5.29 (d, *J* = 2.0 Hz, 1H), 5.24 (d, *J* = 1.9 Hz, 1H), 3.54 – 3.36 (m, 4H), 3.25 (s, 2H), 2.42 (m, 4H), 1.46 (s, 9H).

^{13}**C NMR** (126 MHz, $CDCl_3$) δ 154.9, 142.3, 137.4, 133.1, 132.0, 128.9, 128.8, 127.5, 126.5, 124.4, 119.8, 79.6, 61.4, 52.9, 28.6.

HRMS-ESI (m/z) calc’d for $C_{20}H_{28}Br_1N_2O_2$ $[M+H]^+$, 407.1329; found, 407.1331; deviation: –0.5 ppm.

1,3-Diene 4

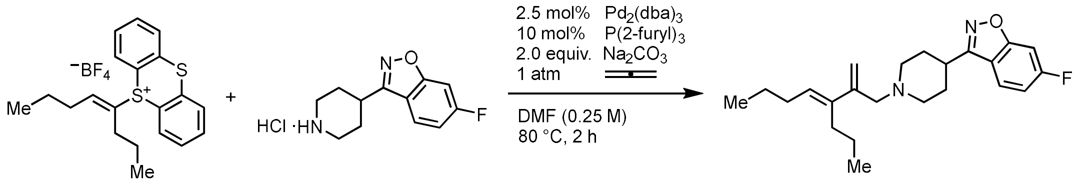

TT-4, 0.300 mmol (*E*/*Z* > 20:1)   1.0 equiv.   **4**, 61% (*E*/*Z* > 20:1)

Caution: Propadiene/allene is a flammable gas and should be handled with appropriate care. Guidelines regarding the safe handling of flammable gases can be found in “Prudent Practices in the Laboratory” (Chapter 4.D).[2]

A 4 mL borosilicate vial equipped with a Teflon-coated magnetic stirring bar was charged with $Pd_2(dba)_3$ (6.9 mg, 7.5 µmol, 2.5 mol%), tri(2-furyl)phosphine (7.0 mg, 30 µmol, 10 mol%), alkenyl thianthrenium salt

TT-4 (124 mg, 0.300 mmol, 1.0 equiv.), Na_2CO_3 (63.6 mg, 0.300 mmol, 2.0 equiv.) and 6-fluoro-3-(4-piperidinyl)-1,2-benzisoxazole hydrochloride (96.0 mg, 0.300 mmol, 1.0 equiv.). Dry DMF (1.2 mL, c = 0.25 M) was added and the vial was sealed with a septum cap. The septum was pierced with an outlet needle (Φ 0.80 × 40 mm) and another needle (Φ 0.80 × 120 mm), which was connected to an allene-containing balloon. A gentle stream of allene gas was passed through the mixture for 2 min. Both needles were removed and the septum cap was quickly wrapped with parafilm. The vial was transferred to a heating block, which had been preheated at 80 °C, and the reaction mixture was stirred at 1000 rpm for 2 h. Then, the vial was removed from the heating block, the stirring bar was removed, and the solvent evaporated using a Biotage V10. The residue was purified by chromatography on silica gel eluting with pentane/EtOAc (50:1 → 40:1 → 30:1 → 20:1 → 15:1, v/v) to afford **4** (68.1 mg, 61%, *E*/*Z* > 20:1) as a yellow oil.

R_f = 0.24 (pentane/EtOAc = 9:1 (v/v), $KMnO_4$).

NMR Spectroscopy:

^{1}H NMR (500 MHz, $CDCl_3$) δ 7.67 (dd, *J* = 8.7, 5.1 Hz, 1H), 7.25 – 7.17 (m, 1H), 7.08 – 6.99 (m, 1H), 5.67 (t, *J* = 7.3 Hz, 1H), 5.11 – 5.00 (m, 2H), 3.12 (s, 2H), 3.07 – 2.97 (m, 3H), 2.26 – 2.20 (m, 2H), 2.13 – 2.06 (m, 4H), 2.06 – 1.97 (m, 4H), 1.47 – 1.34 (m, 4H), 0.94 (t, *J* = 7.4 Hz, 3H), 0.90 (t, *J* = 7.4 Hz, 3H).

^{13}C NMR (126 MHz, $CDCl_3$) δ 165.2, 164.0 (d, *J* = 13.7 Hz), 163.2, 161.5, 146.0, 139.3, 122.8 (d, *J* = 11.2 Hz), 117.5, 112.7, 112.3 (d, *J* = 25.4 Hz), 97.5 (d, *J* = 26.8 Hz), 62.7, 53.6, 34.9, 30.8, 30.4, 30.3, 23.2, 22.3, 14.3, 14.0.

^{19}F NMR (476 MHz, $CDCl_3$) δ –109.76 – –109.85 (m, 1F).

HRMS-EI (m/z) calc'd for $C_{23}H_{31}N_2O_1F_1$ $[M]^+$, 370.2415; found, 370.2419; deviation: –1.1 ppm.

1,3-Diene 5

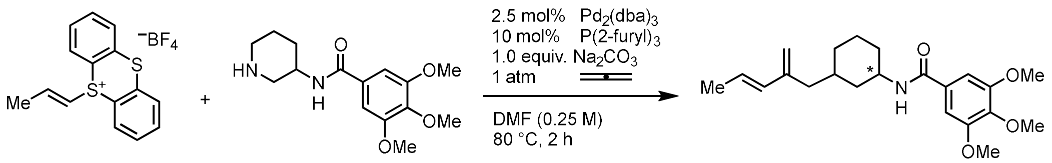

TT-5, 0.300 mmol (*E*/*Z* = 13:1) 1.0 equiv. (±)-**5**, 54% (*E*/*Z* > 20:1)

Caution: Propadiene/allene is a flammable gas and should be handled with appropriate care. Guidelines regarding the safe handling of flammable gases can be found in "Prudent Practices in the Laboratory" (Chapter 4.D).[2]

A 4 mL borosilicate vial equipped with a Teflon-coated magnetic stirring bar was charged with $Pd_2(dba)_3$ (6.9 mg, 7.5 µmol, 2.5 mol%), tri(2-furyl)phosphine (7.0 mg, 30 µmol, 10 mol%), alkenyl thianthrenium salt **TT-5** (103 mg, 0.300 mmol, 1.0 equiv.), Na_2CO_3 (31.8 mg, 0.300 mmol, 1.0 equiv.) and (±)-troxipide (88.3 mg, 0.300 mmol, 1.0 equiv.). Dry DMF (1.2 mL, c = 0.25 M) was added and the vial was sealed with a septum cap. The septum was pierced with an outlet needle (Φ 0.80 × 40 mm) and another needle (Φ 0.80 × 120 mm), which was connected to an allene-containing balloon. A gentle stream of allene gas was passed

through the mixture for 2 min. Both needles were removed and the septum cap was quickly wrapped with parafilm. The vial was transferred to a heating block, which had been preheated at 80 °C, and the reaction mixture was stirred at 1000 rpm for 2 h. Then, the vial was removed from the heating block, the stirring bar was removed, and the solvent evaporated using a Biotage V10. The residue was purified by chromatography on silica gel eluting with pentane/EtOAc (9:1 → 1:1 → 1:1 + 2.5% NEt_3, v/v) to afford (±)-**5** (60.1 mg, 54%, *E*/*Z* > 20:1) as a white solid.

R_f = 0.36 (pentane/EtOAc/NEt_3 = 10:10:1 (v/v)).

NMR Spectroscopy:

^{1}H NMR (500 MHz, $CDCl_3$) δ 6.98 (s, 2H), 6.77 (s, 1H), 6.08 (d, *J* = 15.7 Hz, 1H), 5.99 – 5.85 (m, 1H), 5.01 (s, 2H), 4.29 – 4.18 (m, 1H), 3.91 (s, 6H), 3.88 (s, 3H), 3.14 (d, *J* = 13.5 Hz, 1H), 2.99 (d, *J* = 13.4 Hz, 1H), 2.84 – 2.60 (m, 2H), 2.34 (s, 1H), 2.13 (s, 1H), 1.87 (s, 1H), 1.70 (d, *J* = 6.7 Hz, 3H), 1.59 (d, *J* = 17.2 Hz, 3H).

^{13}C NMR (126 MHz, $CDCl_3$) δ 166.2, 153.3, 142.5, 141.1, 132.3, 130.8, 126.1, 115.5, 104.7, 61.1, 61.0, 57.9, 56.5, 54.4, 45.6, 28.7, 22.0, 18.5.

HRMS-ESI (m/z) calc'd for $C_{21}H_{31}N_2O_4$ $[M+H]^+$, 375.2278; found, 375.2275; deviation: +0.8 ppm.

1,3-Diene 6

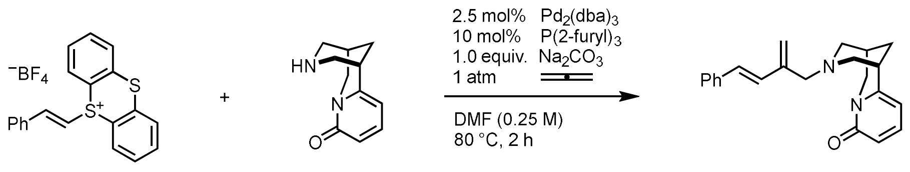

TT-6, 0.300 mmol (*E*/*Z* > 20:1) 1.0 equiv. **6**, 77% (*E*/*Z* > 20:1)

Caution: Propadiene/allene is a flammable gas and should be handled with appropriate care. Guidelines regarding the safe handling of flammable gases can be found in "Prudent Practices in the Laboratory" (Chapter 4.D).[2]

A 4 mL borosilicate vial equipped with a Teflon-coated magnetic stirring bar was charged with $Pd_2(dba)_3$ (6.9 mg, 7.5 µmol, 2.5 mol%), tri(2-furyl)phosphine (7.0 mg, 30 µmol, 10 mol%), alkenyl thianthrenium salt **TT-6** (122 mg, 0.300 mmol, 1.0 equiv.), Na_2CO_3 (31.8 mg, 0.300 mmol, 1.0 equiv.) and (-)-cytisine (57.1 mg, 0.300 mmol, 1.0 equiv.). Dry DMF (1.2 mL, c = 0.25 M) was added and the vial was sealed with a septum cap. The septum was pierced with an outlet needle (Φ 0.80 × 40 mm) and another needle (Φ 0.80 × 120 mm), which was connected to an allene-containing balloon. A gentle stream of allene gas was passed through the mixture for 2 min. Both needles were removed and the septum cap was quickly wrapped with parafilm. The vial was transferred to a heating block, which had been preheated at 80 °C, and the reaction mixture was stirred at 1000 rpm for 2 h. Then, the vial was removed from the heating block, the stirring bar was removed, and the solvent evaporated using a Biotage V10. The residue was purified by column chromatography on silica gel eluting with DCM/iPrOH (100:2, v/v) to afford **6** (76.6 mg, 77%, *E*/*Z* > 20:1) as a

yellow oil.

R*f* = 0.16 (DCM/iPrOH = 100:2 (v/v), $KMnO_4$).

NMR Spectroscopy:

^{1}H NMR (500 MHz, $CDCl_3$) δ 7.27 – 7.18 (m, 4H), 7.19 – 7.12 (m, 1H), 7.10 – 7.02 (m, 1H), 6.62 – 6.53 (m, 1H), 6.53 – 6.45 (m, 1H), 6.22 – 6.14 (m, 1H), 5.92 – 5.83 (m, 1H), 5.09 (s, 1H), 4.94 (s, 1H), 4.10 – 3.99 (m, 1H), 3.93 – 3.82 (m, 1H), 3.21 – 3.11 (m, 1H), 3.06 – 2.95 (m, 2H), 2.95 – 2.86 (m, 2H), 2.46 – 2.38 (m, 1H), 2.29 (dd, *J* = 10.9, 3.2 Hz, 1H), 2.22 (dd, *J* = 10.4, 3.0 Hz, 1H), 1.92 – 1.82 (m, 1H), 1.82 – 1.72 (m, 1H).

^{13}C NMR (126 MHz, $CDCl_3$) δ 163.5, 151.2, 142.1, 138.3, 137.2, 129.5, 128.8, 128.4, 127.4, 126.6, 118.2, 116.7, 104.5, 61.0, 60.4, 60.0, 50.1, 35.5, 28.2, 26.0.

HRMS-ESI (m/z) calc'd for $C_{22}H_{24}N_2O_1$ $[M+Na]^+$, 355.1781; found, 355.1781; deviation: +0.0 ppm.

1,3-Diene 7

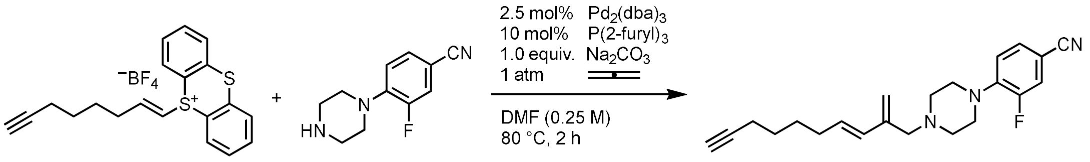

TT-7, 0.300 mmol (*E*/*Z* > 11:1) 1.0 equiv. **7**, 73% (*E*/*Z* > 20:1)

Caution: Propadiene/allene is a flammable gas and should be handled with appropriate care. Guidelines regarding the safe handling of flammable gases can be found in "Prudent Practices in the Laboratory" (Chapter 4.D).[2]

A 4 mL borosilicate vial equipped with a Teflon-coated magnetic stirring bar was charged with $Pd_2(dba)_3$ (6.9 mg, 7.5 µmol, 2.5 mol%), tri(2-furyl)phosphine (7.0 mg, 30 µmol, 10 mol%), alkenyl thianthrenium salt **TT-7** (123 mg, 0.300 mmol, 1.0 equiv.), Na_2CO_3 (31.8 mg, 0.300 mmol, 1.0 equiv.) and 3-fluoro-4-(piperazin-1-yl)benzonitrile (61.6 mg, 0.300 mmol, 1.0 equiv.). Dry DMF (1.2 mL, c = 0.25 M) was added and the vial was sealed with a septum cap. The septum was pierced with an outlet needle (Φ 0.80 × 40 mm) and another needle (Φ 0.80 × 120 mm), which was connected to an allene-containing balloon. A gentle stream of allene gas was passed through the mixture for 2 min. Both needles were removed and the septum cap was quickly wrapped with parafilm. The vial was transferred to a heating block, which had been preheated at 80 °C, and the reaction mixture was stirred at 1000 rpm for 2 h. Then, the vial was removed from the heating block, the stirring bar was removed, and the solvent evaporated using a Biotage V10. The residue was purified by chromatography on silica gel eluting with pentane/EtOAc (9:1 → 7:1, v/v) to afford **7** (76.9 mg, 73%, *E*/*Z* > 20:1) as a colorless oil.

R*f* = 0.22 (pentane/EtOAc = 7:1 (v/v), $KMnO_4$).

NMR Spectroscopy:

^{1}H NMR (500 MHz, $CDCl_3$) δ 7.34 (dd, *J* = 8.5, 1.9 Hz, 1H), 7.28 – 7.23 (m, 1H), 6.90 (t, *J* = 8.5 Hz, 1H), 6.08 (d, *J* = 15.8 Hz, 1H), 5.95 (dt, *J* = 15.7, 6.8 Hz, 1H), 5.11 – 4.99 (m, 2H), 3.22 (t, *J* = 4.9 Hz, 4H), 3.14 (s, 2H), 2.58 (t, *J* = 4.9 Hz, 4H), 2.23 – 2.16 (m, 2H), 2.16 – 2.08 (m, 2H), 1.93 (t, *J* = 2.6 Hz, 1H), 1.59 – 1.48 (m, 4H).

^{13}C NMR (126 MHz, $CDCl_3$) δ 155.0, 153.1, 144.4 (d, *J* = 7.7 Hz), 142.1, 131.2, 131.0, 129.5, 129.5, 119.8 (d, *J* = 24.9 Hz), 118.8 (d, *J* = 4.2 Hz), 118.6 (d, *J* = 2.2 Hz), 115.9, 103.4 (d, *J* = 9.5 Hz), 84.7, 68.4, 61.0, 53.0, 49.9, 49.9, 32.6, 28.5, 28.1, 18.4.

^{19}F NMR (471 MHz, $CDCl_3$) δ –119.20 – –119.28 (m, 1F).

HRMS-ESI (m/z) calc'd for $C_{22}H_{27}F_1N_3$ $[M+H]^+$, 352.2183; found, 352.2182; deviation: +0.4 ppm.

1,3-Diene 8

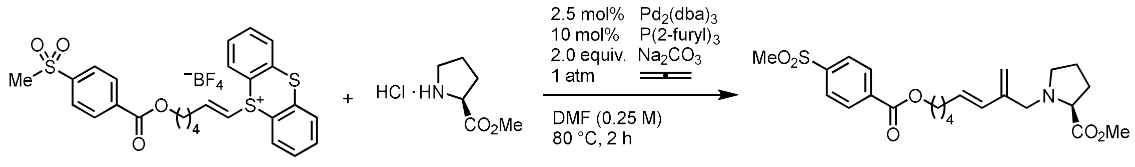

TT-8, 0.300 mmol (*E*/*Z* = 20:1) 1.0 equiv. **8**, 46% (*E*/*Z* > 20:1)

Caution: Propadiene/allene is a flammable gas and should be handled with appropriate care. Guidelines regarding the safe handling of flammable gases can be found in "Prudent Practices in the Laboratory" (Chapter 4.D).[2]

A 4 mL borosilicate vial equipped with a Teflon-coated magnetic stirring bar was charged with $Pd_2(dba)_3$ (6.9 mg, 7.5 µmol, 2.5 mol%), tri(2-furyl)phosphine (7.0 mg, 30 µmol, 10 mol%), alkenyl thianthrenium salt **TT-8** (175 mg, 0.300 mmol, 1.0 equiv.), Na_2CO_3 (63.6 mg, 0.300 mmol, 2.0 equiv..) and *L*-proline methyl ester hydrochloride (49.7 mg, 0.300 mmol, 1.0 equiv.). Dry DMF (1.2 mL, c = 0.25 M) was added and the vial was sealed with a septum cap. The septum was pierced with an outlet needle (Φ 0.80 × 40 mm) and another needle (Φ 0.80 × 120 mm), which was connected to an allene-containing balloon. A gentle stream of allene gas was passed through the mixture for 2 min. Both needles were removed and the septum cap was quickly wrapped with parafilm. The vial was transferred to a heating block, which had been preheated at 80 °C, and the reaction mixture was stirred at 1000 rpm for 2 h. Then, the vial was removed from the heating block, the stirring bar was removed, and the solvent evaporated using a Biotage V10. The residue was purified by chromatography on silica gel eluting with DCM:MeOH (10:1, v/v) to afford **8** (62.2 mg, 46%, *E*/*Z* > 20:1) as a white solid.

R_f = 0.49 (DCM/MeOH = 10:1 (v/v)).

NMR Spectroscopy:

^{1}H NMR (500 MHz, $CDCl_3$) δ 8.20 (d, *J* = 8.2 Hz, 2H), 8.00 (d, *J* = 8.1 Hz, 2H), 6.08 – 5.95 (m, 2H), 5.01 – 4.93 (m, 2H), 4.35 (t, *J* = 6.6 Hz, 2H), 3.66 (s, 3H), 3.52 (d, *J* = 13.0 Hz, 1H), 3.18 (dd, *J* = 8.8, 5.9 Hz, 1H), 3.05 (s, 3H), 3.04 – 2.97 (m, 2H), 2.32 (q, *J* = 8.2 Hz, 1H), 2.16 (q, *J* = 7.0 Hz, 2H), 2.12 – 2.02 (m,

1H), 1.94 – 1.85 (m, 1H), 1.85 – 1.69 (m, 4H), 1.60 – 1.49 (m, 2H).

^{13}C NMR (126 MHz, $CDCl_3$) δ 174.7, 165.0, 144.2, 143.1, 135.3, 131.2, 130.7, 130.6, 127.5, 115.7, 65.9, 65.6, 56.9, 53.2, 51.6, 44.4, 32.6, 29.4, 28.2, 25.7, 23.2.

HRMS-ESI (m/z) calc'd for $C_{23}H_{31}N_1O_6S_1$ $[M+H]^+$, 450.1945; found, 450.1945; deviation: –0.1 ppm.

1,3-Diene 9

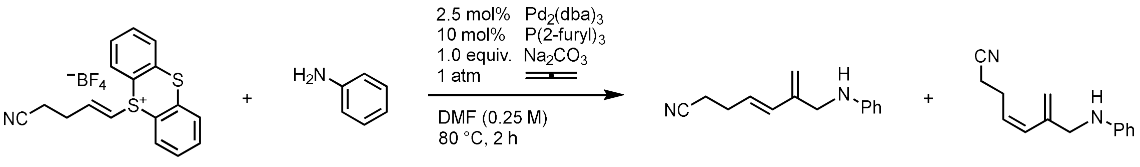

TT-9, 0.300 mmol (*E/Z* = 9:1) 1.0 equiv. *major* minor **9**, 62% (*E/Z* = 13:1)

Caution: Propadiene/allene is a flammable gas and should be handled with appropriate care. Guidelines regarding the safe handling of flammable gases can be found in "Prudent Practices in the Laboratory" (Chapter 4.D).[2]

A 4 mL borosilicate vial equipped with a Teflon-coated magnetic stirring bar was charged with $Pd_2(dba)_3$ (6.9 mg, 7.5 µmol, 2.5 mol%), tri(2-furyl)phosphine (7.0 mg, 30 µmol, 10 mol%), alkenyl thianthrenium salt **TT-9** (103 mg, 0.300 mmol, 1.0 equiv.), Na_2CO_3 (31.8 mg, 0.300 mmol, 1.0 equiv.) and aniline (27.9 mg, 27.3 µL, 0.300 mmol, 1.0 equiv.). Dry DMF (1.2 mL, c = 0.25 M) was added and the vial was sealed with a septum cap. The septum was pierced with an outlet needle (Φ 0.80 × 40 mm) and another needle (Φ 0.80 × 120 mm), which was connected to an allene-containing balloon. A gentle stream of allene gas was passed through the mixture for 2 min. Both needles were removed and the septum cap was quickly wrapped with parafilm. The vial was transferred to a heating block, which had been preheated at 80 °C, and the reaction mixture was stirred at 1000 rpm for 2 h. Then, the vial was removed from the heating block, the stirring bar was removed, and the solvent evaporated using a Biotage V10. The residue was purified by chromatography on silica gel eluting with pentane/EtOAc (5:1, v/v) to afford **9** (46.9 mg, 62%, *E/Z* = 13:1) as an orange oil.

R_f = 0.22 (pentane/EtOAc = 10:1 (v/v)).

NMR Spectroscopy:

E-isomer:

^{1}H NMR (600 MHz, $CDCl_3$) δ 7.21 – 7.17 (m, 2H), 6.74 – 6.69 (m, 1H), 6.64 – 6.58 (m, 2H), 6.22 (dq, *J* = 16.0, 1.2 Hz, 1H), 5.72 (dt, *J* = 16.0, 7.0 Hz, 1H), 5.19 (q, *J* = 1.5 Hz, 1H), 5.11 (dt, *J* = 1.6, 0.8 Hz, 1H), 3.91 (t, *J* = 1.2 Hz, 2H), 2.34 (t, *J* = 7.1 Hz, 2H), 2.29 (qd, *J* = 7.2, 1.4 Hz, 2H), 1.79 (p, *J* = 7.2 Hz, 2H).

^{13}C NMR (151 MHz, $CDCl_3$) δ 148.2, 142.2, 132.5, 129.3, 127.8, 119.6, 117.5, 115.8, 112.8, 45.8, 31.8, 24.9, 16.5.

Z-isomer:

^{1}H NMR (600 MHz, $CDCl_3$) δ 7.21 – 7.17 (m, 2H), 6.74 – 6.69 (m, 1H), 6.64 – 6.58 (m, 2H), 5.98 – 5.94

(m, 1H), 5.50 (dt, J = 11.7, 7.3 Hz, 1H), 5.32 (qd, J = 1.7, 0.5 Hz, 1H), 3.79 (t, J = 1.1 Hz, 1H), 2.37 (ddd, J = 14.7, 7.3, 1.8 Hz, 2H), 2.23 (t, J = 7.2 Hz, 2H), 1.70 (p, J = 7.3 Hz, 2H).

^{13}C NMR (151 MHz, $CDCl_3$) δ 147.9, 142.3, 131.2, 129.9, 129.3, 119.7, 117.6, 115.6, 113.0, 49.3, 27.6, 25.6, 16.6.

HRMS-EI (m/z) calc'd for $C_{15}H_{18}N_1$ $[M]^+$, 226.1464; found, 226.1465; deviation: –0.2 ppm.

1,3-Diene 10

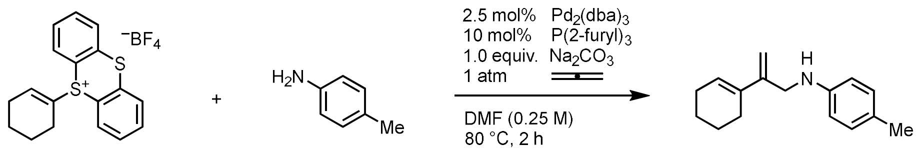

TT-1, 0.300 mmol 1.0 equiv. **10**, 81%

Caution: Propadiene/allene is a flammable gas and should be handled with appropriate care. Guidelines regarding the safe handling of flammable gases can be found in "Prudent Practices in the Laboratory" (Chapter 4.D).[2]

A 4 mL borosilicate vial equipped with a Teflon-coated magnetic stirring bar was charged with $Pd_2(dba)_3$ (6.9 mg, 7.5 µmol, 2.5 mol%), tri(2-furyl)phosphine (7.0 mg, 30 µmol, 10 mol%), alkenyl thianthrenium salt **TT-1** (115 mg, 0.300 mmol, 1.0 equiv.), Na_2CO_3 (31.8 mg, 0.300 mmol, 1.0 equiv.) and *p*-toluidine (32.1 mg, 0.300 mmol, 1.0 equiv.). Dry DMF (1.2 mL, c = 0.25 M) was added and the vial was sealed with a septum cap. The septum was pierced with an outlet needle (Φ 0.80 × 40 mm) and another needle (Φ 0.80 × 120 mm), which was connected to an allene-containing balloon. A gentle stream of allene gas was passed through the mixture for 2 min. Both needles were removed and the septum cap was quickly wrapped with parafilm. The vial was transferred to a heating block, which had been preheated at 80 °C, and the reaction mixture was stirred at 1000 rpm for 2 h. Then, the vial was removed from the heating block, the stirring bar was removed, and the solvent evaporated using a Biotage V10. The residue was purified by chromatography on silica gel eluting with pentane/EtOAc (100:1 → 80:1, v/v) to afford **10** (55.3 mg, 81%) as a colorless oil.

R_f = 0.87 (pentane/EtOAc = 9:1 (v/v), $KMnO_4$).

NMR Spectroscopy:

^{1}H NMR (500 MHz, $CDCl_3$) δ 7.00 (d, J = 8.0 Hz, 2H), 6.60 – 6.46 (m, 2H), 5.97 (d, J = 2.0 Hz, 1H), 5.11 (d, J = 14.2 Hz, 2H), 3.92 (s, 2H), 3.69 (br s, 1H), 2.25 (s, 3H), 2.24 – 2.20 (m, 2H), 2.20 – 2.13 (m, 2H), 1.75 – 1.68 (m, 2H), 1.65 – 1.58 (m, 2H).

^{13}C NMR (126 MHz, $CDCl_3$) δ 146.2, 145.0, 134.9, 129.8, 126.5, 125.2, 113.0, 110.5, 47.4, 26.1, 26.0, 23.0, 22.3, 20.5.

HRMS-EI (m/z) calc'd for $C_{16}H_{21}N_1$ $[M]^+$, 227.1668; found, 227.1669; deviation: –0.1 ppm.

1,3-Diene 11a

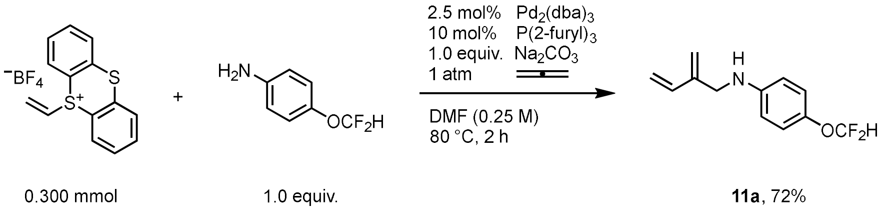

Caution: Propadiene/allene is a flammable gas and should be handled with appropriate care. Guidelines regarding the safe handling of flammable gases can be found in "Prudent Practices in the Laboratory" (Chapter 4.D).[2]

A 4 mL borosilicate vial equipped with a Teflon-coated magnetic stirring bar was charged with $Pd_2(dba)_3$ (6.9 mg, 7.5 μmol, 2.5 mol%), tri(2-furyl)phosphine (7.0 mg, 30 μmol, 10 mol%), vinyl-TT (99.1 mg, 0.300 mmol, 1.0 equiv.), Na_2CO_3 (31.8 mg, 0.300 mmol, 1.0 equiv.) and 4-(difluoromethoxy)aniline (47.7 mg, 43.6 μL, 0.300 mmol, 1.0 equiv.). Dry DMF (1.2 mL, c = 0.25 M) was added and the vial was sealed with a septum cap. The septum was pierced with an outlet needle (Φ 0.80 × 40 mm) and another needle (Φ 0.80 × 120 mm), which was connected to an allene-containing balloon. A gentle stream of allene gas was passed through the mixture for 2 min. Both needles were removed and the septum cap was quickly wrapped with parafilm. The vial was transferred to a heating block, which had been preheated at 80 °C, and the reaction mixture was stirred at 1000 rpm for 2 h. Then, the vial was removed from the heating block, the stirring bar was removed, and the solvent evaporated using a Biotage V10. The residue was purified by chromatography on silica gel eluting with pentane/EtOAc (70:1, v/v) to afford **11a** (48.8 mg, 72%) as a yellow oil.

R_f = 0.53 (pentane/EtOAc = 9:1 (v/v), $KMnO_4$).

NMR Spectroscopy:

^{1}H NMR (500 MHz, $CDCl_3$) δ 7.02 – 6.95 (m, 2H), 6.60 – 6.56 (m, 2H), 6.48 (dd, *J* = 17.8, 11.0 Hz, 1H), 6.55– 6.25 (t, *J* = 74.9 Hz, 1H), 5.33 (d, *J* = 17.8 Hz, 1H), 5.28 – 5.24 (m, 1H), 5.21 – 5.15 (m, 2H), 3.94 (s, 2H), 3.89 (s, 1H).

^{13}C NMR (126 MHz, $CDCl_3$) δ 146.2, 142.6, 142.5, 137.3, 121.6, 118.8 – 114.7 (t, = 253.0 Hz), 117.0, 114.22, 113.4, 45.6.

^{19}F NMR (476 MHz, $CDCl_3$) δ –79.97 (d, *J* = 74.9 Hz, 2F).

HRMS-EI (m/z) calc'd for $C_{12}H_{13}N_1O_1F_2$ $[M]^+$, 225.0960; found, 225.0964; deviation: –1.8 ppm.

Scale-up synthesis 1,3-diene 11a

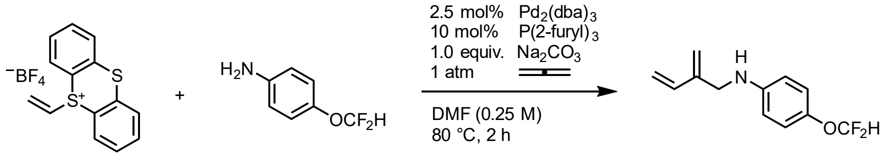

0.300 mmol 1.0 equiv. **11a**, 74%

Caution: Propadiene/allene is a flammable gas and should be handled with appropriate care. Guidelines regarding the safe handling of flammable gases can be found in "Prudent Practices in the Laboratory" (Chapter 4.D).[2]

Under an ambient atmosphere, a 50 mL Schlenk tube vial equipped with a Teflon-coated magnetic stirring bar was charged with $Pd_2(dba)_3$ (45.7 mg, 50.0 µmol, 2.50 mol%), tri(2-furyl)phosphine (46.3 mg, 200 µmol, 10.0 mol%), vinyl-TT (660 mg, 2.00 mmol, 1.0 equiv.), Na_2CO_3 (212 mg, 2.00 mmol, 1.0 equiv.) and 4-(difluoromethoxy)aniline (47.7 mg, 231 µL. 0.300 mmol, 1.0 equiv.) (see image **A**). Dry DMF (8.0 mL, c = 0.25 M) was added and the Schlenk tube was sealed with a rubber septum. The septum was pierced with an outlet needle (Φ 0.80 × 40 mm) and another needle, which was connected to an allene-containing balloon. A gentle stream of allene gas was passed through the mixture for 3 min (see image **B**). Both needles were removed and the Schlenk tube was immersed in a silicon oil bath, which had been preheated at 80 °C. A blast shield was placed in front of the reaction vessel and the reaction mixture was stirred at 600 rpm for 2 h (see image **C**). Then, heating was stopped, the Schlenk tube was lifted out of the oil bath, and allowed to cool to 23 °C. The stirring bar was removed and the solvent evaporated using a Biotage V10. The residue was purified by chromatography on silica gel eluting with pentane/EtOAc (100:1 → 70:1, v/v) to afford **11a** (335 mg, 74%) as a yellow oil.

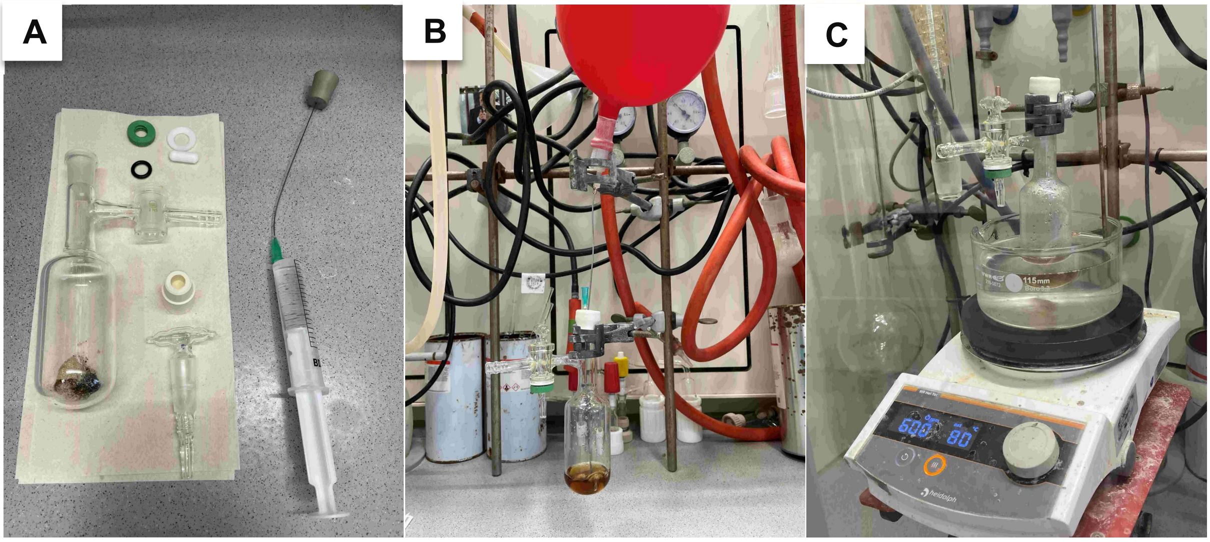

1,3-Diene 11b

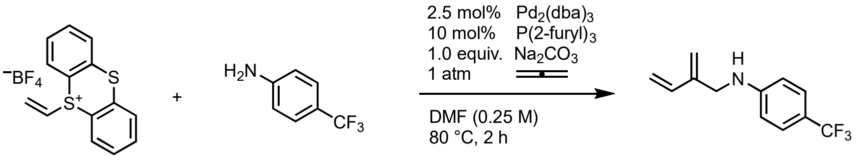

0.300 mmol | 1.0 equiv. | **11b**, 57%

Caution: Propadiene/allene is a flammable gas and should be handled with appropriate care. Guidelines regarding the safe handling of flammable gases can be found in “Prudent Practices in the Laboratory” (Chapter 4.D).[2]

A 4 mL borosilicate vial equipped with a Teflon-coated magnetic stirring bar was charged with $Pd_2(dba)_3$ (6.9 mg, 7.5 μmol, 2.5 mol%), tri(2-furyl)phosphine (7.0 mg, 30 μmol, 10 mol%), vinyl-TT (99.1 mg, 0.300 mmol, 1.0 equiv.), Na_2CO_3 (31.8 mg, 0.300 mmol, 1.0 equiv.) and 4-(trifluoromethyl)aniline (48.3 mg, 0.300 mmol, 1.0 equiv.). Dry DMF (1.2 mL, c = 0.25 M) was added and the vial was sealed with a septum cap. The septum was pierced with an outlet needle (Φ 0.80 × 40 mm) and another needle (Φ 0.80 × 120 mm), which was connected to an allene-containing balloon. A gentle stream of allene gas was passed through the mixture for 2 min. Both needles were removed and the septum cap was quickly wrapped with parafilm. The vial was transferred to a heating block, which had been preheated at 80 °C, and the reaction mixture was stirred at 1000 rpm for 2 h. Then, the vial was removed from the heating block, the stirring bar was removed, and the solvent evaporated using a Biotage V10. The residue was purified by chromatography on silica gel eluting with pentane/EtOAc (150:1, v/v) to afford **11b** (39.1 mg, 57%) as a yellow oil.

R*f* = 0.60 (pentane/EtOAc = 9:1 (v/v), $KMnO_4$).

NMR Spectroscopy:

^{1}H NMR (500 MHz, $CDCl_3$) δ 7.41 (d, *J* = 8.6 Hz, 2H), 6.60 (d, *J* = 8.6 Hz, 2H), 6.47 (dd, *J* = 17.8, 11.0 Hz, 1H), 5.30 (d, *J* = 17.8 Hz, 1H), 5.24 – 5.21 (m, 1H), 5.21 – 5.15 (m, 2H), 4.26 – 4.12 (m, 1H), 3.98 (d, *J* = 5.3 Hz, 2H).

^{13}C NMR (126 MHz, $CDCl_3$) δ 150.6, 141.9, 137.1, 126.7 (q, *J* = 3.9 Hz), 125.2 (q, *J* = 270.3 Hz), 118.9 (q, *J* = 32.8 Hz), 117.2, 114.4, 112.0, 44.8.

^{19}F NMR (476 MHz, $CDCl_3$) δ –61.00 (s, 3F).

HRMS-EI (m/z) calc'd for $C_{12}H_{12}N_1F_3$ $[M]^+$, 227.0916; found, 227.0920; deviation: –1.5 ppm.

1,3-Diene 11c

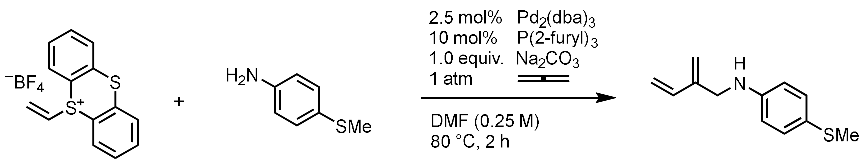

0.300 mmol 1.0 equiv. **11c**, 77%

Caution: Propadiene/allene is a flammable gas and should be handled with appropriate care. Guidelines regarding the safe handling of flammable gases can be found in "Prudent Practices in the Laboratory" (Chapter 4.D).[2]

A 4 mL borosilicate vial equipped with a Teflon-coated magnetic stirring bar was charged with $Pd_2(dba)_3$ (6.9 mg, 7.5 µmol, 2.5 mol%), tri(2-furyl)phosphine (7.0 mg, 30 µmol, 10 mol%), vinyl-TT (99.1 mg, 0.300 mmol, 1.0 equiv.), Na_2CO_3 (31.8 mg, 0.300 mmol, 1.0 equiv.) and 4-(methylmercapto)aniline (41.8 mg, 0.300 mmol, 1.0 equiv.). Dry DMF (1.2 mL, c = 0.25 M) was added and the vial was sealed with a septum cap. The septum was pierced with an outlet needle (Φ 0.80 × 40 mm) and another needle (Φ 0.80 × 120 mm), which was connected to an allene-containing balloon. A gentle stream of allene gas was passed through the mixture for 2 min. Both needles were removed and the septum cap was quickly wrapped with parafilm. The vial was transferred to a heating block, which had been preheated at 80 °C, and the reaction mixture was stirred at 1000 rpm for 2 h. Then, the vial was removed from the heating block, the stirring bar was removed, and the solvent evaporated using a Biotage V10. The residue was purified by chromatography on silica gel eluting with pentane/EtOAc (200:1, v/v) to afford **11c** (47.4 mg, 77%) as a yellow oil.

R_f = 0.60 (pentane/EtOAc = 9:1 (v/v), $KMnO_4$).

NMR Spectroscopy:

^{1}H NMR (500 MHz, $CDCl_3$) δ 7.24 – 7.22 (m, 1H), 7.22 – 7.20 (m, 1H), 6.57 – 6.55 (m, 1H), 6.55 – 6.53 (m, 1H), 6.46 (dd, J = 17.8, 11.0 Hz, 1H), 5.33 – 5.26 (m, 1H), 5.26 – 5.21 (m, 1H), 5.18 – 5.13 (m, 2H), 3.93 (t, J = 1.3 Hz, 2H), 3.88 (s, 1H), 2.41 (s, 3H).

^{13}C NMR (126 MHz, $CDCl_3$) δ 147.1, 142.5, 137.3, 131.6, 124.4, 117.0, 114.2, 113.5, 45.2, 19.3.

HRMS-EI (m/z) calc'd for $C_{12}H_{15}N_1S_1$ $[M]^+$, 205.0920; found, 205.0923; deviation: –1.5 ppm.

1,3-Diene (±)-12

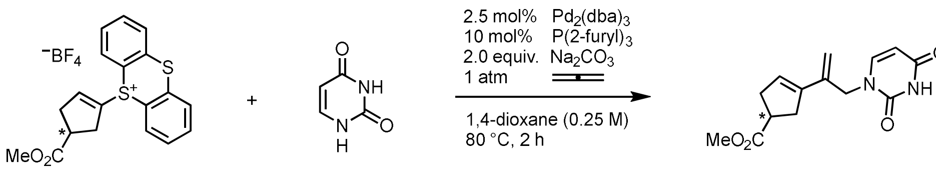

(±)-**TT-12**, 0.300 mmol 1.0 equiv. (±)-**12**, 50%

Caution: Propadiene/allene is a flammable gas and should be handled with appropriate care. Guidelines

regarding the safe handling of flammable gases can be found in “Prudent Practices in the Laboratory” (Chapter 4.D).[2]

A 4 mL borosilicate vial equipped with a Teflon-coated magnetic stirring bar was charged with $Pd_2(dba)_3$ (6.9 mg, 7.5 µmol, 2.5 mol%), tri(2-furyl)phosphine (7.0 mg, 30 µmol, 10 mol%), alkenyl thianthrenium salt (±)-**TT-12** (128 mg, 0.300 mmol, 1.0 equiv.), Na_2CO_3 (63.6 mg, 0.600 mmol, 2.0 equiv..), and uracil (33.6 mg, 0.300 mmol, 1.0 equiv.). Dry 1,4-dioxane (1.2 mL, c = 0.25 M) was added and the vial was sealed with a septum cap. The septum was pierced with an outlet needle (Φ 0.80 × 40 mm) and another needle (Φ 0.80 × 120 mm), which was connected to an allene-containing balloon. A gentle stream of allene gas was passed through the mixture for 2 min. Both needles were removed and the septum cap was quickly wrapped with parafilm. The vial was transferred to a heating block, which had been preheated at 80 °C, and the reaction mixture was stirred at 1000 rpm for 2 h. Then, the vial was removed from the heating block, the stirring bar was removed, and the solvent evaporated using a Biotage V10. The residue was purified by column chromatography on silica gel eluting with pentane/EtOAc (2:1 → 1:1 → 1:2, v/v) to afford (±)-**12** (41.3 mg, 50%) as a yellow oil.

R*f* = 0.30 (pentane/EtOAc = 1:2 (v/v), $KMnO_4$).

NMR Spectroscopy:

^{1}H NMR (600 MHz, $CDCl_3$) δ 8.91 (s, 1H), 7.13 (d, *J* = 8.0 Hz, 1H), 5.81 – 5.77 (m, 1H), 5.71 (d, *J* = 7.9 Hz, 1H), 5.24 – 5.20 (m, 1H), 5.05 – 5.02 (m, 1H), 4.66 – 4.60 (m, 1H), 4.52 – 4.46 (m, 1H), 3.70 (s, 3H), 3.23 – 3.15 (m, 1H), 2.89 – 2.80 (m, 2H), 2.80 – 2.77 (m, 2H).

^{13}C NMR (151 MHz, $CDCl_3$) δ 176.1, 163.5, 150.9, 143.4, 138.9, 137.7, 127.2, 116.1, 102.6, 52.1, 49.6, 41.2, 37.3, 36.5.

HRMS-EI (m/z) calc’d for $C_{14}H_{16}N_2O_4$ $[M]^+$, 276.1105; found, 276.1104; deviation: +0.1 ppm.

1,3-Diene (±)-13

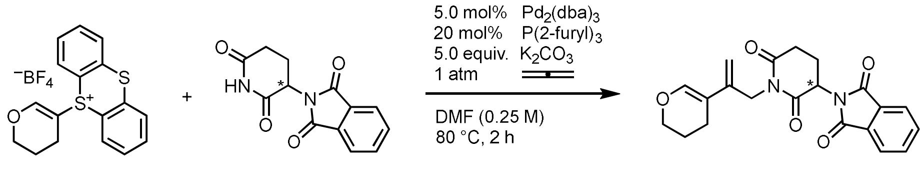

TT-13, 0.300 mmol 1.0 equiv. (±)-**13**, 48%

Caution: Propadiene/allene is a flammable gas and should be handled with appropriate care. Guidelines regarding the safe handling of flammable gases can be found in “Prudent Practices in the Laboratory” (Chapter 4.D).[2]

A 4 mL borosilicate vial equipped with a Teflon-coated magnetic stirring bar was charged with $Pd_2(dba)_3$ (13.7 mg, 15.0 µmol, 5.00 mol%), tri(2-furyl)phosphine (13.9 mg, 60 µmol, 20 mol%), alkenyl thianthrenium salt **TT-13** (99.1 mg, 0.300 mmol, 1.0 equiv.), K_2CO_3 (207 mg, 1.50 mmol, 5.0 equiv.), and (±)-thalidomide

(77.5 mg, 0.300 mmol, 1.0 equiv.). Dry DMF (1.2 mL, c = 0.25 M) was added and the vial was sealed with a septum cap. The septum was pierced with an outlet needle (Φ 0.80 × 40 mm) and another needle (Φ 0.80 × 120 mm), which was connected to an allene-containing balloon. A gentle stream of allene gas was passed through the mixture for 2 min. Both needles were removed and the septum cap was quickly wrapped with parafilm. The vial was transferred to a heating block, which had been preheated at 80 °C, and the reaction mixture was stirred at 1000 rpm for 2 h. Then, the vial was removed from the heating block, the stirring bar was removed, and the solvent evaporated using a Biotage V10. The residue was purified by chromatography on silica gel eluting with pentane/EtOAc (3:1 → 1:1, v/v) to afford (±)-**13** (55.2 mg, 48%) as a yellow oil.

R*f* = 0.60 (pentane/EtOAc = 1:1 (v/v), $KMnO_4$).

NMR Spectroscopy:

^{1}**H NMR** (500 MHz, $CDCl_3$) δ 7.93 – 7.82 (m, 2H), 7.82 – 7.68 (m, 2H), 6.70 (s, 1H), 5.05 (m, 1H), 4.89 (s, 1H), 4.60 (s, 1H), 4.56 (s, 2H), 3.94 (t, *J* = 5.3 Hz, 2H), 3.09 – 3.00 (m, 1H), 2.94 – 2.78 (m, 2H), 2.24 – 2.14 (m, 3H), 1.94 – 1.84 (m, 2H).

^{13}**C NMR** (126 MHz, $CDCl_3$) δ 170.7, 168.4, 167.5, 142.5, 138.8, 134.5, 131.9, 123.9, 112.2, 104.5, 65.8, 50.3, 41.4, 32.2, 22.2, 22.1, 21.3.

HRMS-EI (m/z) calc'd for $C_{21}H_{20}N_2O_5$ $[M]^+$, 380.1367; found, 380.1365; deviation: +0.4 ppm.

1,3-Diene 14

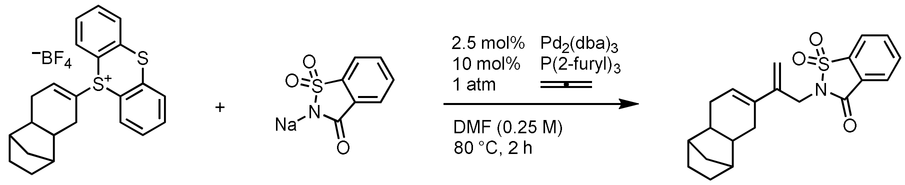

TT-11, 0.300 mmol 1.0 equiv. **14**, 83%

Caution: Propadiene/allene is a flammable gas and should be handled with appropriate care. Guidelines regarding the safe handling of flammable gases can be found in "Prudent Practices in the Laboratory" (Chapter 4.D).[2]

A 4 mL borosilicate vial equipped with a Teflon-coated magnetic stirring bar was charged with with $Pd_2(dba)_3$ (6.9 mg, 7.5 μmol, 2.5 mol%), tri(2-furyl)phosphine (7.0 mg, 30 μmol, 10 mol%), alkenyl thianthrenium salt **TT-14** (135 mg, 0.300 mmol, 1.0 equiv.), and saccharin's sodium salt (61.5 mg, 0.300 mmol, 1.0 equiv.). Dry DMF (1.2 mL, c = 0.25 M) was added and the vial was sealed with a septum cap. The septum was pierced with an outlet needle (Φ 0.80 × 40 mm) and another needle (Φ 0.80 × 120 mm), which was connected to an allene-containing balloon. A gentle stream of allene gas was passed through the mixture for 2 min. Both needles were removed and the septum cap was quickly wrapped with parafilm. The vial was transferred to a heating block, which had been preheated at 80 °C, and the reaction mixture was stirred at 1000 rpm for 2 h. Then, the vial was removed from the heating block, the stirring bar was removed, and the solvent evaporated

using a Biotage V10. The residue was purified by chromatography on silica gel eluting with Hex/EtOAc (15:1 → 10:1, v/v) to afford **14** (91.4 mg, 83%) as a colorless oil.

R*f* = 0.21 (Hex/EtOAc = 9:1 (v/v), $KMnO_4$).

NMR Spectroscopy:

^{1}H NMR (500 MHz, $CDCl_3$) δ 8.07 (d, *J* = 7.5 Hz, 1H), 7.92 (d, *J* = 7.4 Hz, 1H), 7.89 – 7.78 (m, 2H), 6.14 – 6.02 (m, 1H), 5.28 (s, 1H), 5.15 (s, 1H), 4.59 (q, *J* = 16.2 Hz, 2H), 2.66 – 2.49 (m, 1H), 2.40 – 2.24 (m, 1H), 1.96 (d, *J* = 20.2 Hz, 2H), 1.69 – 1.55 (m, 4H), 1.51 (d, *J* = 8.4 Hz, 3H), 1.19 (d, *J* = 7.6 Hz, 2H), 1.03 (d, *J* = 10.1 Hz, 1H).

^{13}C NMR (126 MHz, $CDCl_3$) δ 159.1, 139.7, 138.1, 137.3, 134.9, 134.4, 127.3, 125.8, 125.4, 121.1, 112.2, 43.4, 43.3, 43.0, 42.7, 41.6, 33.4, 29.8, 29.7, 28.9, 28.8.

HRMS-EI (m/z) calc'd for $C_{21}H_{23}N_1O_3S_1$ $[M]^+$, 369.1393; found, 369.1392; deviation: +0.1 ppm.

1,3-Diene 15

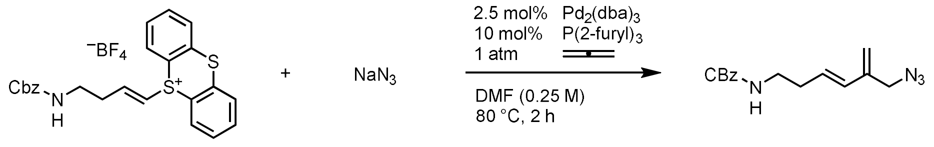

TT-15, 0.300 mmol (*E/Z* > 20:1) 1.0 equiv. **15**, 73% (*E/Z* > 20:1)

Caution: Propadiene/allene is a flammable gas and should be handled with appropriate care. Guidelines regarding the safe handling of flammable gases can be found in "Prudent Practices in the Laboratory" (Chapter 4.D).[2]

A 4 mL borosilicate vial equipped with a Teflon-coated magnetic stirring bar was charged with $Pd_2(dba)_3$ (6.9 mg, 7.5 µmol, 2.5 mol%), tri(2-furyl)phosphine (7.0 mg, 30 µmol, 10 mol%), alkenyl thianthrenium salt **TT-15** (152 mg, 0.300 mmol, 1.0 equiv.) and sodium azide (19.5 mg, 0.300 mmol, 1.0 equiv.). Dry DMF (1.2 mL, c = 0.25 M) was added and the vial was sealed with a septum cap. The septum was pierced with an outlet needle (Φ 0.80 × 40 mm) and another needle (Φ 0.80 × 120 mm), which was connected to an allene-containing balloon. A gentle stream of allene gas was passed through the mixture for 2 min. Both needles were removed and the septum cap was quickly wrapped with parafilm. The vial was transferred to a heating block, which had been preheated at 80 °C, and the reaction mixture was stirred at 1000 rpm for 2 h. Then, the vial was removed from the heating block, the stirring bar was removed, and the solvent evaporated using a Biotage V10. The residue was purified by column chromatography on silica gel eluting with pentane/EtOAc (8:2, v/v) to afford **15** (62.4 mg, 73%, *E/Z* > 20:1) as a yellow oil.

R*f* = 0.25 (pentane/EtOAc = 5:1 (v/v), $KMnO_4$).

NMR Spectroscopy:

^{1}H NMR (500 MHz, $CDCl_3$) δ 7.35 (d, *J* = 4.3 Hz, 4H), 7.33 – 7.29 (m, 1H), 6.14 (d, *J* = 15.9 Hz, 1H),

5.81 – 5.61 (m, 1H), 5.26 – 5.02 (m, 4H), 4.82 (s, 1H), 3.90 (s, 2H), 3.30 (q, *J* = 6.5 Hz, 2H), 2.34 (q, *J* = 6.9 Hz, 2H).

^{13}C NMR (126 MHz, $CDCl_3$) δ 156.4, 139.5, 136.7, 131.8, 128.7, 128.3, 128.3, 118.3, 66.8, 52.8, 40.5, 33.5.

HRMS-ESI (m/z) calc'd for $C_{15}H_{18}N_4O_2$ $[M+Na]^+$, 309.1322; found, 309.1323; deviation: –0.3 ppm.

1,3-Diene 16

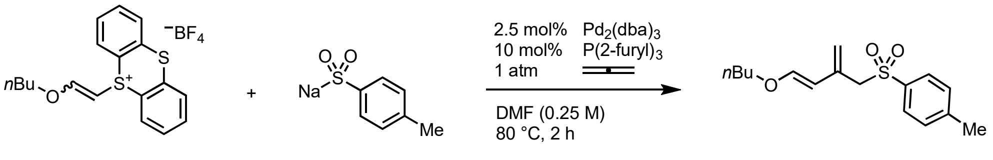

TT-16, 0.300 mmol (*E/Z* = 1:1.6) 1.0 equiv. **16**, 71% (*E/Z* > 20:1)

Caution: Propadiene/allene is a flammable gas and should be handled with appropriate care. Guidelines regarding the safe handling of flammable gases can be found in "Prudent Practices in the Laboratory" (Chapter 4.D).[2]

A 4 mL borosilicate vial equipped with a Teflon-coated magnetic stirring bar was charged with $Pd_2(dba)_3$ (6.9 mg, 7.5 µmol, 2.5 mol%), tri(2-furyl)phosphine (7.0 mg, 30 µmol, 10 mol%), alkenyl thianthrenium salt **TT-16** (121 mg, 0.300 mmol, 1.0 equiv.) and sodium *p*-toluenesulfinate (53.5 mg, 0.300 mmol, 1.0 equiv.). Dry DMF (1.2 mL, c = 0.25 M) was added and the vial was sealed with a septum cap. The septum was pierced with an outlet needle (Φ 0.80 × 40 mm) and another needle (Φ 0.80 × 120 mm), which was connected to an allene-containing balloon. A gentle stream of allene gas was passed through the mixture for 2 min. Both needles were removed and the septum cap was quickly wrapped with parafilm. The vial was transferred to a heating block, which had been preheated at 80 °C, and the reaction mixture was stirred at 1000 rpm for 2 h. Then, the vial was removed from the heating block, the stirring bar was removed, and the solvent evaporated using a Biotage V10. The residue was purified by chromatography on silica gel eluting with hexanes/EtOAc (30:1 → 20:1 → 10:1, v/v) to afford **16** (63.0 mg, 71%, *E*/*Z* > 20:1) as a colorless oil.

R_f = 0.25 (Hex/EtOAc = 9:1 (v/v), $KMnO_4$).

NMR Spectroscopy:

^{1}H NMR (500 MHz, $CDCl_3$) δ 7.78 – 7.72 (m, 2H), 7.32 (d, *J* = 8.0 Hz, 2H), 6.57 (d, *J* = 12.8 Hz, 1H), 5.45 (d, *J* = 12.8 Hz, 1H), 5.00 (s, 1H), 4.58 (s, 1H), 3.88 (s, 2H), 3.67 (t, *J* = 6.6 Hz, 2H), 2.44 (s, 3H), 1.65 – 1.56 (m, 2H), 1.43 – 1.35 (m, 2H), 0.93 (t, *J* = 7.4 Hz, 3H).

^{13}C NMR (126 MHz, $CDCl_3$) δ 150.1, 144.7, 135.5, 132.4, 129.6, 128.9, 118.0, 106.2, 70.2, 61.3, 31.4, 21.7, 19.2, 13.9.

HRMS-ESI (m/z) calc'd for $C_{16}H_{22}S_1O_3$ $[M+Na]^+$, 317.1182; found, 317.1182; deviation: ±0.0 ppm.

1,3-Diene 17

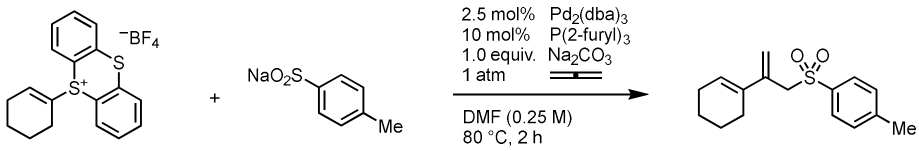

TT-1, 0.300 mmol + 1.0 equiv. → **17**, 62%

Caution: Propadiene/allene is a flammable gas and should be handled with appropriate care. Guidelines regarding the safe handling of flammable gases can be found in "Prudent Practices in the Laboratory" (Chapter 4.D).[2]

A 4 mL borosilicate vial equipped with a Teflon-coated magnetic stirring bar was charged with $Pd_2(dba)_3$ (6.9 mg, 7.5 µmol, 2.5 mol%), tri(2-furyl)phosphine (7.0 mg, 30 µmol, 10 mol%), alkenyl thianthrenium salt **TT-1** (115 mg, 0.300 mmol, 1.0 equiv.), Na_2CO_3 (31.8 mg, 0.300 mmol, 1.0 equiv.) and *p*-toluenesulfinic acid sodium salt (32.1 mg, 0.300 mmol, 1.0 equiv.). Dry DMF (1.2 mL, c = 0.25 M) was added and the vial was sealed with a septum cap. The septum was pierced with an outlet needle (Φ 0.80 × 40 mm) and another needle (Φ 0.80 × 120 mm), which was connected to an allene-containing balloon. A gentle stream of allene gas was passed through the mixture for 2 min. Both needles were removed and the septum cap was quickly wrapped with parafilm. The vial was transferred to a heating block, which had been preheated at 80 °C, and the reaction mixture was stirred at 1000 rpm for 2 h. Then, the vial was removed from the heating block, the stirring bar was removed, and the solvent evaporated using a Biotage V10. The residue was purified by chromatography on silica gel eluting with pentane/EtOAc (19:1 → 9:1, v/v) to afford **17** (51.5 mg, 62%) as a colorless oil.

$\mathbf{R_f}$ = 0.31 (pentane/EtOAc = 9:1 (v/v), $KMnO_4$).

NMR Spectroscopy:

^{1}**H NMR** (500 MHz, $CDCl_3$) δ 7.72 – 7.67 (m, 2H), 7.29 (d, *J* = 8.0 Hz, 2H), 5.70 (t, *J* = 4.2 Hz, 1H), 5.24 (s, 1H), 4.97 (s, 1H), 4.03 (s, 2H), 2.42 (s, 3H), 2.05 – 1.98 (m, 2H), 1.98 – 1.88 (m, 2H), 1.57 – 1.49 (m, 2H), 1.46 – 1.38 (m, 2H).

^{13}**C NMR** (126 MHz, $CDCl_3$) δ 144.5, 136.8, 136.1, 134.0, 129.5, 128.9, 127.2, 117.9, 60.6, 26.0, 25.9, 22.7, 21.8, 21.7.

HRMS-ESI (m/z) calc'd for $C_{16}H_{20}S_1N_2$ $[M+Na]^+$, 299.1076; found, 299.1080; deviation: –1.3 ppm.

1,3-Diene 18a

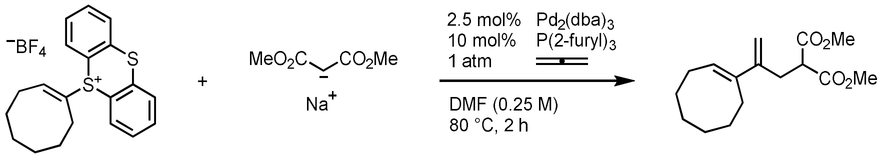

TT-18, 0.300 mmol + 1.50 equiv. → **18a**, 93%

Caution: Propadiene/allene is a flammable gas and should be handled with appropriate care. Guidelines regarding the safe handling of flammable gases can be found in "Prudent Practices in the Laboratory" (Chapter 4.D).[2]

Caution: Heated mixtures of DMF and sodium hydride are known to be potentially explosive.[5] Careful risk assessment should be carried out before performing reactions involving DMF, NaH and heat. Substitution of DMF with 1,4-dioxane was not feasible due to the formation of a thick white goo that was ineffective in the reaction.

A 4 mL borosilicate vial equipped with a Teflon-coated magnetic stirring bar was charged with sodium hydride (18.0 mg, 0.450 mmol, 1.50 equiv., 60% dispersion in mineral oil) and dry DMF (1.2 mL). Under stirring, diethyl malonate (59.5 mg, 51.7 µL, 0.450 mmol, 1.50 equiv.) was added dropwise using a 100 µL Hamilton syringe, and the resulting mixture was stirred for 15 min at 23 °C. Another 4 mL borosilicate vial equipped with a Teflon-coated magnetic stirring bar was charged with $Pd_2(dba)_3$ (6.9 mg, 7.5 µmol, 2.5 mol%), tri(2-furyl)phosphine (7.0 mg, 30 µmol, 10 mol%) and alkenyl thianthrenium salt **TT-18** (124 mg, 0.300 mmol, 1.0 equiv.). To the same vial, the solution from the first vial was added and the vial was sealed with a septum cap. The septum was pierced with an outlet needle (Φ 0.80 × 40 mm) and another needle (Φ 0.80 × 120 mm), which was connected to an allene-containing balloon. A gentle stream of allene gas was passed through the mixture for 2 min. Both needles were removed and the septum cap was quickly wrapped with parafilm. The vial was transferred to a heating block, which had been preheated at 80 °C. A blast shield was placed in front of the stirring plate, and the reaction mixture was stirred at 1000 rpm for 2 h. Then, the vial was removed from the heating block, the stirring bar was removed, and the solvent evaporated using a Biotage V10. The residue was purified by column chromatography on silica gel eluting with pentane/EtOAc (75:1 → 50:1 → 30:1, v/v) to afford **18a** (77.8 mg, 93%) as a yellow oil.

R_f = 0.20 (pentane/EtOAc = 50:1 (v/v), $KMnO_4$).

NMR Spectroscopy:

^{1}H NMR (500 MHz, $CDCl_3$) δ 5.82 (t, *J* = 8.2 Hz, 1H), 5.06 (s, 1H), 4.90 (s, 1H), 3.71 (s, 6H), 3.64 (t, *J* = 7.7 Hz, 1H), 2.89 (d, *J* = 7.6 Hz, 2H), 2.46 – 2.32 (m, 2H), 2.32 – 2.12 (m, 2H), 1.56 – 1.37 (m, 8H).

^{13}C NMR (126 MHz, $CDCl_3$) δ 169.7, 143.7, 138.4, 127.9, 112.7, 52.5, 51.4, 33.3, 30.4, 29.0, 27.5, 27.2, 26.1, 25.8.

HRMS-ESI (m/z) calc'd for $C_{16}H_{24}O_4$ $[M+Na]^+$, 303.1567; found, 303.1568; deviation: –0.3 ppm.

1,3-Diene 18b

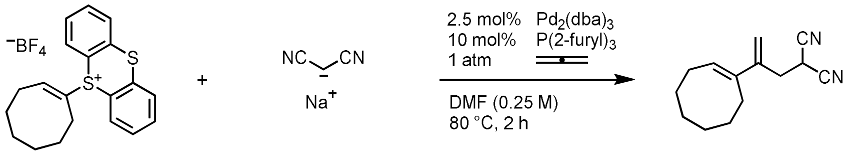

TT-18, 0.300 mmol 1.50 equiv. **18b**, 45%

Caution: Propadiene/allene is a flammable gas and should be handled with appropriate care. Guidelines regarding the safe handling of flammable gases can be found in "Prudent Practices in the Laboratory" (Chapter 4.D).[2]

Caution: Heated mixtures of DMF and sodium hydride are known to be potentially explosive.[5] Careful risk assessment should be carried out before performing reactions involving DMF, NaH and heat. Substitution of DMF with 1,4-dioxane was not feasible due to the formation of a thick white goo that was ineffective in the reaction.

A 4 mL borosilicate vial equipped with a Teflon-coated magnetic stirring bar was charged with sodium hydride (18.0 mg, 0.450 mmol, 1.50 equiv., 60% dispersion in mineral oil) and malonitrile (29.7 mg, 0.450 mmol, 1.50 equiv.). Dry DMF (1.2 mL) was added and the resulting mixture was stirred for 15 min at 23 °C. Another 4 mL borosilicate vial equipped with a Teflon-coated magnetic stirring bar was charged with $Pd_2(dba)_3$ (6.9 mg, 7.5 μmol, 2.5 mol%), tri(2-furyl)phosphine (7.0 mg, 30 μmol, 10 mol%) and alkenyl thianthrenium salt **TT-18** (124 mg, 0.300 mmol, 1.0 equiv.). To the same vial, the solution from the first vial was added and the vial was sealed with a septum cap. The septum was pierced with an outlet needle (Φ 0.80 × 40 mm) and another needle (Φ 0.80 × 120 mm), which was connected to an allene-containing balloon. A gentle stream of allene gas was passed through the mixture for 2 min. Both needles were removed and the septum cap was quickly wrapped with parafilm. The vial was transferred to a heating block, which had been preheated at 80 °C. A blast shield was placed in front of the stirring plate, and the reaction mixture was stirred at 1000 rpm for 2 h. Then, the vial was removed from the heating block, the stirring bar was removed, and the solvent evaporated using a Biotage V10. The residue was purified by column chromatography on silica gel eluting with hexanes/EtOAc (30:1, v/v) to afford **18b** (28.9 mg, 45%) as a colorless oil.

R*f* = 0.25 (hexanes/EtOAc = 19:1 (v/v), $KMnO_4$).

NMR Spectroscopy:

^{1}H NMR (500 MHz, $CDCl_3$) δ 5.76 (t, *J* = 8.2 Hz, 1H), 5.35 (s, 1H), 5.19 (s, 1H), 3.90 (t, *J* = 7.7 Hz, 1H), 2.98 (d, *J* = 7.7 Hz, 2H), 2.49 – 2.33 (m, 2H), 2.31 – 2.16 (m, 2H), 1.62 – 1.52 (m, 4H), 1.52 – 1.39 (m, 4H).

^{13}C NMR (126 MHz, $CDCl_3$) δ 140.1, 137.5, 129.0, 116.9, 112.6, 35.8, 30.2, 29.0, 27.5, 27.2, 26.1, 22.7.

HRMS-ESI (m/z) calc'd for $C_{14}H_{17}N_2$ $[M]^+$, 213.1397; found, 213.1398; deviation: –0.5 ppm.

1,3-Diene (±)-18c

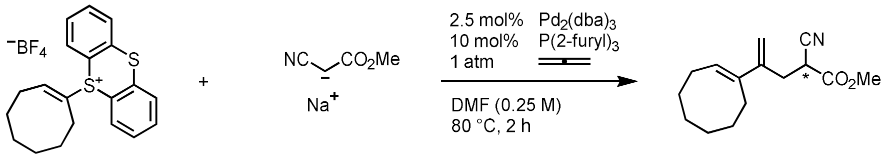

TT-18, 0.300 mmol 1.50 equiv. (±)-**18c**, 37%

Caution: Propadiene/allene is a flammable gas and should be handled with appropriate care. Guidelines

regarding the safe handling of flammable gases can be found in "Prudent Practices in the Laboratory" (Chapter 4.D).[2]

Caution: Heated mixtures of DMF and sodium hydride are known to be potentially explosive.[5] Careful risk assessment should be carried out before performing reactions involving DMF, NaH and heat. Substitution of DMF with 1,4-dioxane was not feasible due to the formation of a thick white goo that was ineffective in the reaction.

A 4 mL borosilicate vial equipped with a Teflon-coated magnetic stirring bar was charged with sodium hydride (18.0 mg, 0.450 mmol, 1.50 equiv., 60% dispersion in mineral oil) and dry DMF (1.2 mL). Under stirring, methyl cyanoacetate (44.6 mg, 39.8 µL, 0.450 mmol, 1.50 equiv.) was added dropwise using a 100 µL Hamilton syringe, and the resulting mixture was stirred for 15 min at 23 °C. Another 4 mL borosilicate vial equipped with a Teflon-coated magnetic stirring bar was charged with $Pd_2(dba)_3$ (6.9 mg, 7.5 µmol, 2.5 mol%), tri(2-furyl)phosphine (7.0 mg, 30 µmol, 10 mol%) and alkenyl thianthrenium salt **TT-18** (124 mg, 0.300 mmol, 1.0 equiv.). To the same vial, the solution from the first vial was added and the vial was sealed with a septum cap. The septum was pierced with an outlet needle (Φ 0.80 × 40 mm) and another needle (Φ 0.80 × 120 mm), which was connected to an allene-containing balloon. A gentle stream of allene gas was passed through the mixture for 2 min. Both needles were removed and the septum cap was quickly wrapped with parafilm. The vial was transferred to a heating block, which had been preheated at 80 °C. A blast shield was placed in front of the stirring plate, and the reaction mixture was stirred at 1000 rpm for 2 h. Then, the vial was removed from the heating block, the stirring bar was removed, and the solvent evaporated using a Biotage V10. The residue was purified by column chromatography on silica gel eluting with hexanes/EtOAc (40:1, v/v) to afford (±)-**18c** (28.0 mg, 37%) as a colorless oil.

R_f = 0.22 (hexanes/EtOAc = 19:1 (v/v), $KMnO_4$).

NMR Spectroscopy:

^{1}H NMR (500 MHz, $CDCl_3$) δ 5.80 (t, *J* = 8.2 Hz, 1H), 5.23 (s, 1H), 5.07 (s, 1H), 3.81 (s, 3H), 3.71 (dd, *J* = 9.6, 5.7 Hz, 1H), 3.05 (dd, *J* = 14.1, 5.8 Hz, 1H), 2.74 (dd, *J* = 14.2, 9.6 Hz, 1H), 2.42 (t, *J* = 6.3 Hz, 2H), 2.26 – 2.18 (m, 2H), 1.62 – 1.50 (m, 4H), 1.50 – 1.36 (m, 4H).

^{13}C NMR (151 MHz, $CDCl_3$) δ 166.6, 141.8, 138.0, 128.5, 116.3, 115.0, 53.6, 37.5, 34.8, 30.3, 29.0, 27.5, 27.2, 26.1, 26.0.

HRMS-ESI (m/z) calc'd for $C_{26}H_{28}O_2$ $[M+Na]^+$, 395.1982; found, 395.1980; deviation: +0.3 ppm.

1,3-Diene 18d

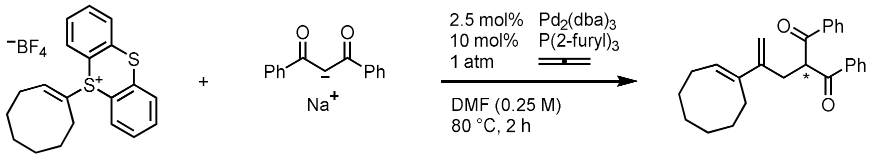

TT-18, 0.300 mmol 1.50 equiv. **18d**, 93%

Caution: Propadiene/allene is a flammable gas and should be handled with appropriate care. Guidelines regarding the safe handling of flammable gases can be found in “Prudent Practices in the Laboratory” (Chapter 4.D).[2]

Caution: Heated mixtures of DMF and sodium hydride are known to be potentially explosive.[5] Careful risk assessment should be carried out before performing reactions involving DMF, NaH and heat. Substitution of DMF with 1,4-dioxane was not feasible due to the formation of a thick white goo that was ineffective in the reaction.

A 4 mL borosilicate vial equipped with a Teflon-coated magnetic stirring bar was charged with sodium hydride (18.0 mg, 0.450 mmol, 1.50 equiv., 60% dispersion in mineral oil) and 1,3-diphenyl-1,3-propanedione (29.7 mg, 0.450 mmol, 1.50 equiv.). Dry DMF (1.2 mL) was added and the resulting mixture was stirred for 15 min at 23 °C. Another 4 mL borosilicate vial equipped with a Teflon-coated magnetic stirring bar was charged with $Pd_2(dba)_3$ (6.9 mg, 7.5 µmol, 2.5 mol%), tri(2-furyl)phosphine (7.0 mg, 30 µmol, 10 mol%) and alkenyl thianthrenium salt **TT-18** (124 mg, 0.300 mmol, 1.0 equiv.). To the same vial, the solution from the first vial was added and the vial was sealed with a septum cap. The septum was pierced with an outlet needle (Φ 0.80 × 40 mm) and another needle (Φ 0.80 × 120 mm), which was connected to an allene-containing balloon. A gentle stream of allene gas was passed through the mixture for 2 min. Both needles were removed and the septum cap was quickly wrapped with parafilm. The vial was transferred to a heating block, which had been preheated at 80 °C. A blast shield was placed in front of the stirring plate, and the reaction mixture was stirred at 1000 rpm for 2 h. Then, the vial was removed from the heating block, the stirring bar was removed, and the solvent evaporated using a Biotage V10. The residue was purified by column chromatography on silica gel eluting with hexanes/EtOAc (75:1 → 60:1, v/v) to afford **18d** (104 mg, 93%) as a faint yellow oil.

R_f = 0.33 (hexanes/EtOAc = 19:1 (v/v), $KMnO_4$).

NMR Spectroscopy:

^{1}H NMR (500 MHz, $CDCl_3$) δ 7.95 – 7.87 (m, 4H), 7.57 – 7.50 (m, 2H), 7.42 (t, *J* = 7.8 Hz, 4H), 5.83 (t, *J* = 8.2 Hz, 1H), 5.44 (t, *J* = 6.6 Hz, 1H), 5.02 – 4.95 (m, 1H), 4.82 (s, 1H), 3.12 (dd, *J* = 6.6, 0.9 Hz, 2H), 2.38 – 2.29 (m, 2H), 2.26 – 2.15 (m, 2H), 1.56 – 1.38 (m, 8H).

^{13}C NMR (151 MHz, $CDCl_3$) δ 196.1, 144.7, 139.2, 136.5, 133.5, 128.8, 128.8, 128.8, 128.3, 113.2, 56.2, 33.9, 30.3, 29.2, 27.5, 27.2, 26.3, 26.2.

HRMS-ESI (m/z) calc’d for $C_{26}H_{28}O_2$ $[M+Na]^+$, 395.1980; found, 395.1982; deviation: +0.3 ppm.

1,3-Diene 21a and allylic amine 21a

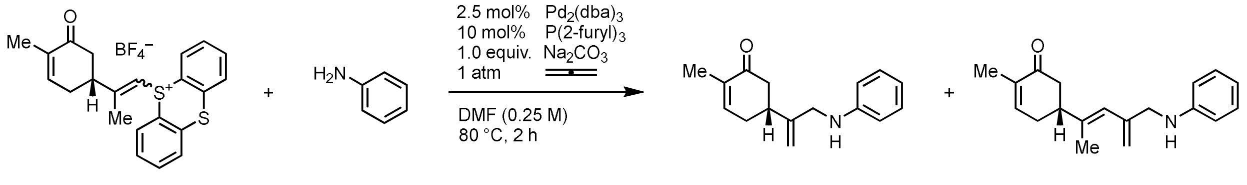

TT-21, 0.200 mmol (*E*/*Z* = 7:1)

1.0 equiv.

21b, 13%

21a, 24% (*E*/*Z* > 20:1)

Caution: Propadiene/allene is a flammable gas and should be handled with appropriate care. Guidelines regarding the safe handling of flammable gases can be found in “Prudent Practices in the Laboratory” (Chapter 4.D).[2]

A 4 mL borosilicate vial equipped with a Teflon-coated magnetic stirring bar was charged with $Pd_2(dba)_3$ (4.6 mg, 5.0 μmol, 2.5 mol%), tri(2-furyl)phosphine (4.6 mg, 20 μmol, 10 mol%), alkenyl thianthrenium salt **TT-21** (90.5 mg, 0.200 mmol, 1.0 equiv.) and aniline (18 μL, 18.6 mg, 0.200 mmol, 1.0 equiv.). Dry DMF (0.8 mL, c = 0.25 M) was added and the vial was sealed with a septum cap. The septum was pierced with an outlet needle (Φ 0.80 × 40 mm) and another needle (Φ 0.80 × 120 mm), which was connected to an allene-containing balloon. A gentle stream of allene gas was passed through the mixture for 2 min. Both needles were removed and the septum cap was quickly wrapped with parafilm. The vial was transferred to a heating block, which had been preheated at 80 °C, and the reaction mixture was stirred at 1000 rpm for 2 h. Then, the vial was removed from the heating block, the stirring bar was removed, and the solvent evaporated using a Biotage V10. The residue was purified by column chromatography on silica gel eluting with pentane/EtOAc (12:1, v/v) to afford **21a** (13.6 mg, 24%, *E*/*Z* > 20:1) and **21b** (6.3 mg, 13%) as a yellow oil.

*1,3-diene **18**:*

$\mathbf{R_f}$ = 0.30 (pentane/EtOAc = 5:1 (v/v), $KMnO_4$).

NMR Spectroscopy:

^{1}**H NMR** (500 MHz, $CDCl_3$) δ 7.20 – 7.12 (m, 2H), 6.78 – 6.73 (m, 1H), 6.73 – 6.66 (m, 1H), 6.63 – 6.55 (m, 2H), 5.76 – 5.67 (m, 1H), 5.33 – 5.25 (m, 1H), 5.02 – 4.93 (m, 1H), 3.94 – 3.78 (m, 1H), 3.74 (s, 2H), 2.78 – 2.69 (m, 1H), 2.59 – 2.50 (m, 1H), 2.44 – 2.28 (m, 3H), 1.83 – 1.78 (m, 6H).

^{13}**C NMR** (126 MHz, $CDCl_3$) δ 199.8, 148.2, 144.7, 142.7, 141.2, 135.5, 129.3, 123.9, 117.6, 114.7, 113.1, 49.8, 45.0, 43.2, 31.4, 16.1, 15.8.

HRMS-EI (m/z) calc'd for $C_{19}H_{23}N_1O_1$ $[M]^+$, 281.1774; found, 281.1777; deviation: –0.8 ppm.

*Allylic amine **18a**:*

$\mathbf{R_f}$ = 0.23 (pentane/EtOAc = 5:1 (v/v), $KMnO_4$).

NMR Spectroscopy:

^{1}**H NMR** (500 MHz, $CDCl_3$) δ 7.20 – 7.13 (m, 2H), 6.78 – 6.74 (m, 1H), 6.74 – 6.68 (m, 1H), 6.62 – 6.55 (m, 2H), 5.16 (s, 1H), 4.98 (s, 1H), 3.84 (s, 1H), 3.77 (s, 2H), 2.89 – 2.77 (m, 1H), 2.72 – 2.62 (m, 1H), 2.60 – 2.49 (m, 1H), 2.47 – 2.40 (m, 1H), 2.36 (ddt, *J* = 18.2, 10.7, 2.6 Hz, 1H), 1.84 – 1.75 (m, 3H).

^{13}**C NMR** (151 MHz, $CDCl_3$) δ 199.6, 148.3, 148.1, 144.5, 135.7, 129.4, 117.8, 113.0, 111.0, 47.9, 43.5, 39.2, 31.8, 15.9.

HRMS-EI (m/z) calc'd for $C_{16}H_{19}N_1O_1$ $[M]^+$, 241.1461; found, 241.1461; deviation: ±0.0 ppm.

Allylic amine 21c

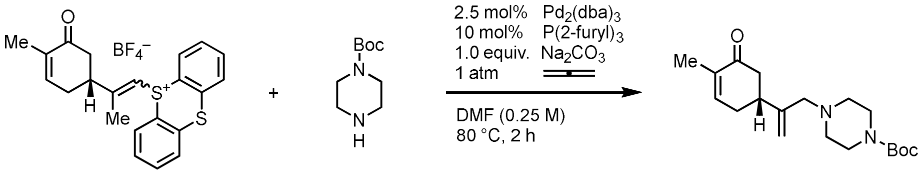

TT-21, 0.200 mmol (*E/Z* = 7:1) 1.0 equiv. **21c**, 34%

Caution: Propadiene/allene is a flammable gas and should be handled with appropriate care. Guidelines regarding the safe handling of flammable gases can be found in "Prudent Practices in the Laboratory" (Chapter 4.D).[2]

A 4 mL borosilicate vial equipped with a Teflon-coated magnetic stirring bar was charged with $Pd_2(dba)_3$ (4.6 mg, 5.0 µmol, 2.5 mol%), tri(2-furyl)phosphine (4.6 mg, 20 µmol, 10 mol%), alkenyl thianthrenium salt **TT-21** (90.5 mg, 0.200 mmol, 1.0 equiv.) and N-*tert*-butoxycarbonylpiperazine (37.3 mg, 0.200 mmol, 1.0 equiv.). Dry DMF (0.8 mL, c = 0.25 M) was added and the vial was sealed with a septum cap. The septum was pierced with an outlet needle (Φ 0.80 × 40 mm) and another needle (Φ 0.80 × 120 mm), which was connected to an allene-containing balloon. A gentle stream of allene gas was passed through the mixture for 2 min. Both needles were removed and the septum cap was quickly wrapped with parafilm. The vial was transferred to a heating block, which had been preheated at 80 °C, and the reaction mixture was stirred at 1000 rpm for 2 h. Then, the vial was removed from the heating block, the stirring bar was removed, and the solvent evaporated using a Biotage V10. The residue was purified by column chromatography on silica gel eluting with pentane/EtOAc (9:1, v/v) to afford **21c** (22.6 mg, 34%) as a colorless oil.

R_f = 0.23 (pentane/EtOAc = 9:1 (v/v), $KMnO_4$).

NMR Spectroscopy:

^{1}H NMR (500 MHz, $CDCl_3$) δ 6.75 (d, *J* = 5.8 Hz, 1H), 5.02 (s, 1H), 4.94 (s, 1H), 3.39 (s, 4H), 3.02 – 2.80 (m, 3H), 2.66 – 2.56 (m, 1H), 2.55 – 2.45 (m, 1H), 2.45 – 2.34 (m, 2H), 2.34 – 2.24 (m, 4H), 1.82 – 1.74 (m, 3H), 1.45 (s, 9H).

^{13}C NMR (151 MHz, $CDCl_3$) δ 200.0, 154.9, 144.7, 135.6, 79.8, 63.3, 53.0, 43.5, 38.9, 31.7, 28.6, 15.9.

HRMS-EI (m/z) calc'd for $C_{19}H_{31}N_2O_3$ $[M]^+$, 335.2329; found, 335.2328; deviation: +0.3 ppm.

SPECTROSCOPIC DATA

^{1}H NMR of (±)-CP-1

CDCl$_3$, 500 MHz, 23 °C

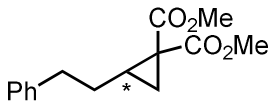

(±)-**CP-1**

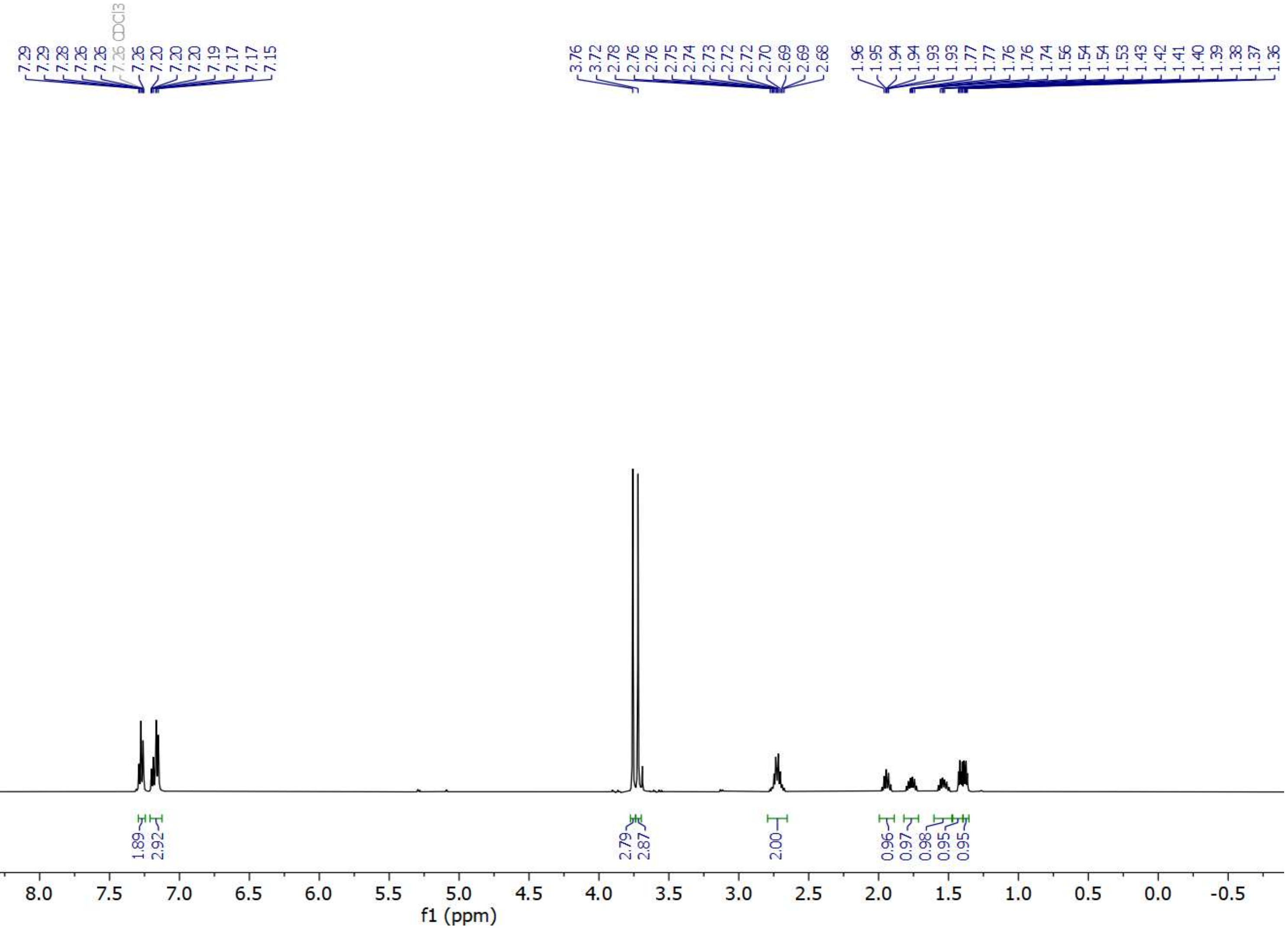

^{13}C NMR of (±)-CP-1

$CDCl_3$, 126 MHz, 23 °C

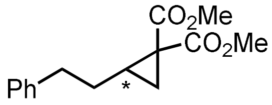

(±)-**CP-1**

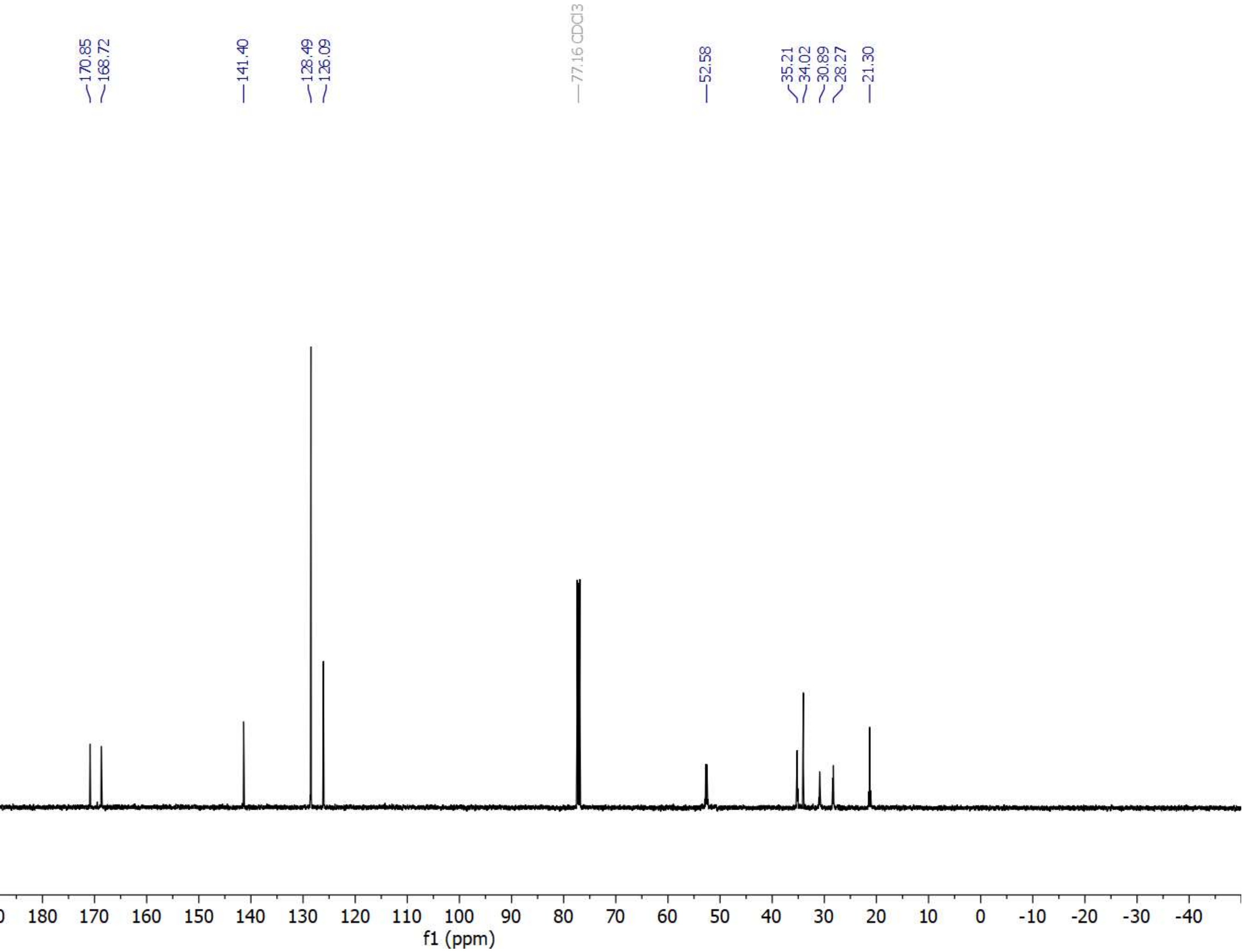

^{1}H NMR of 16-Alk

CDCl$_3$, 500 MHz, 23 °C

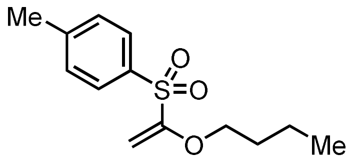

16-Alk

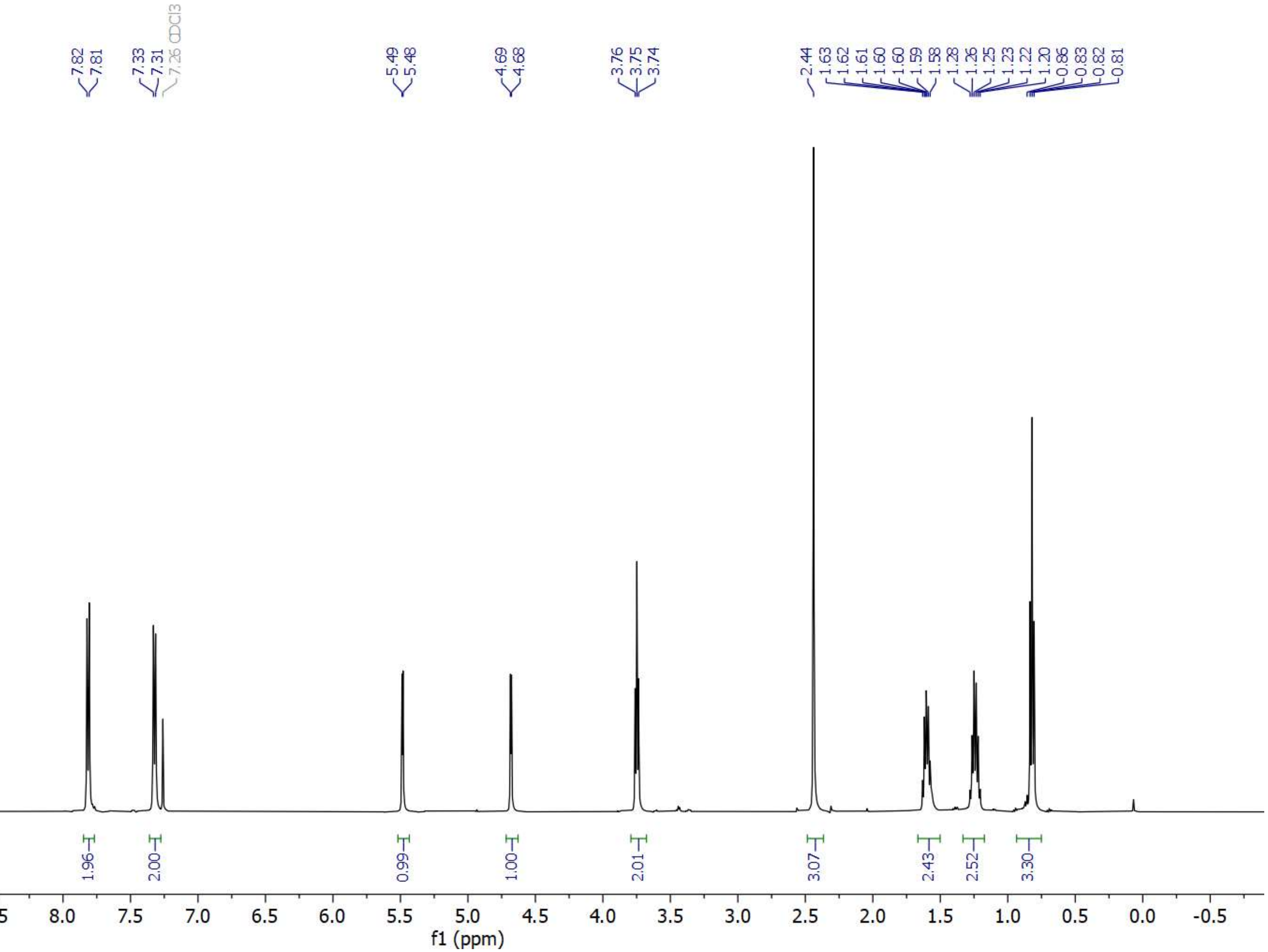

^{13}C NMR of 16-Alk

$CDCl_3$, 151 MHz, 23 °C

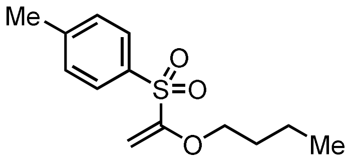

16-Alk

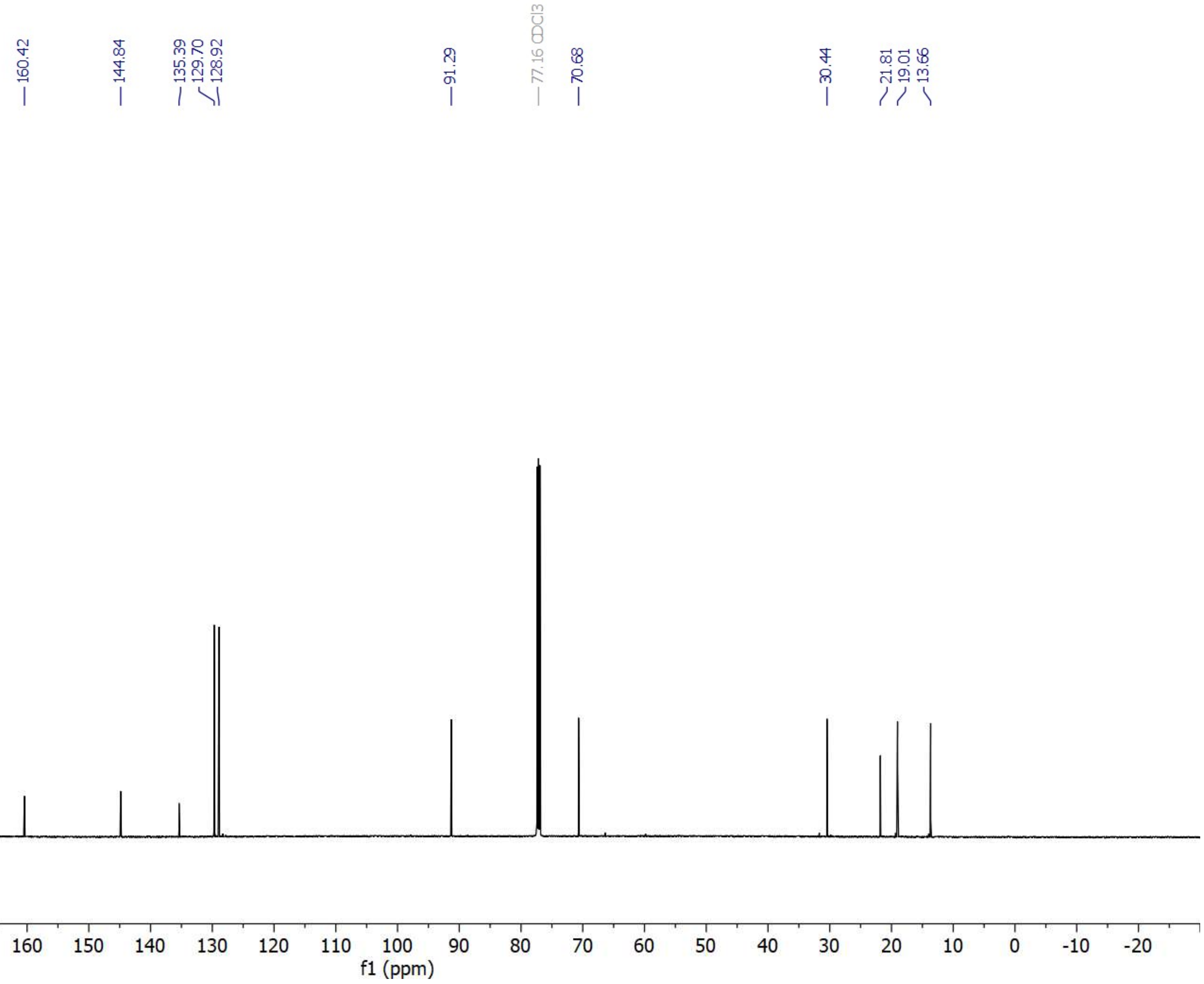

^{1}H NMR of Alk-8

CDCl$_3$, 500 MHz, 23 °C

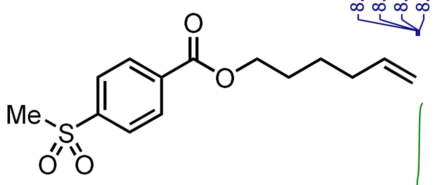

Alk-8

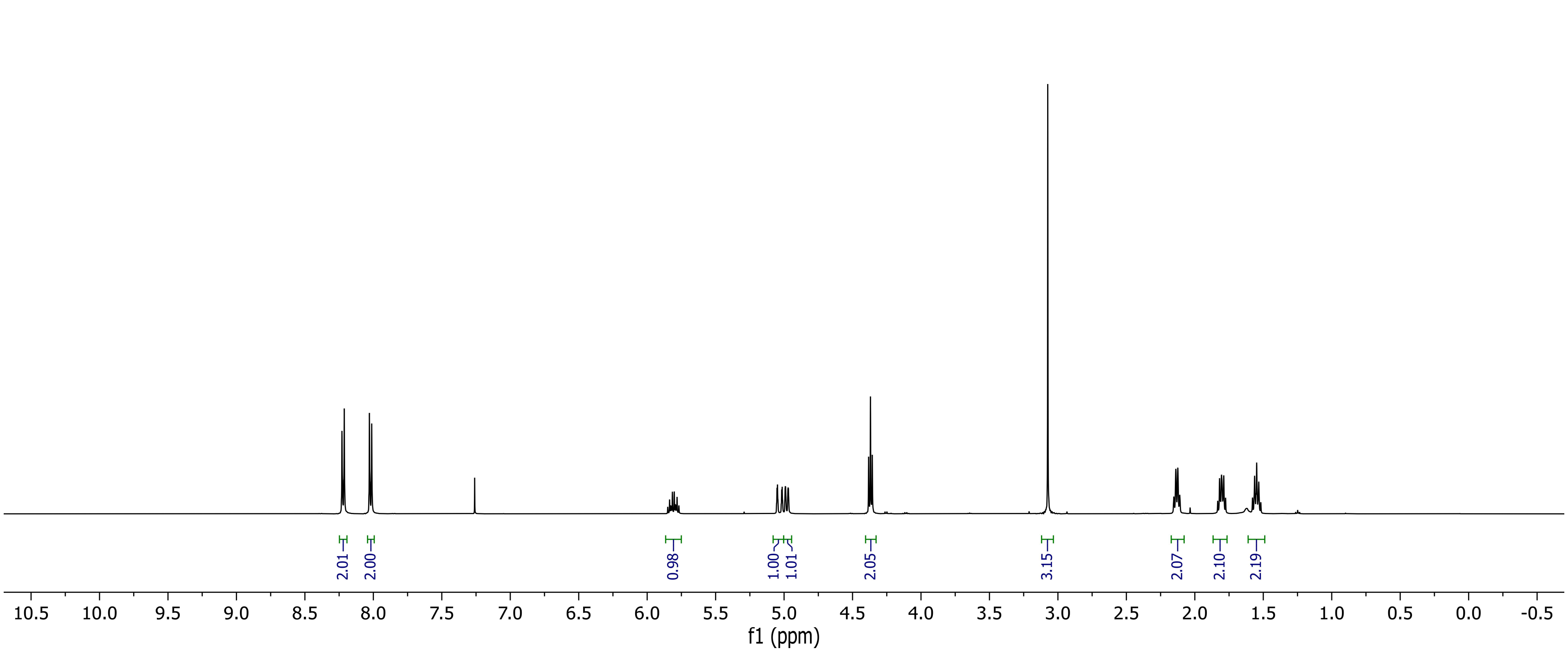

^{13}C NMR of Alk-8

$CDCl_3$, 126 MHz, 23 °C

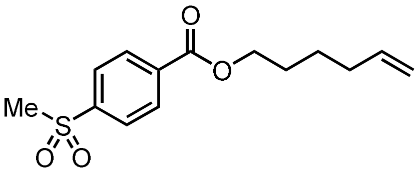

Alk-8

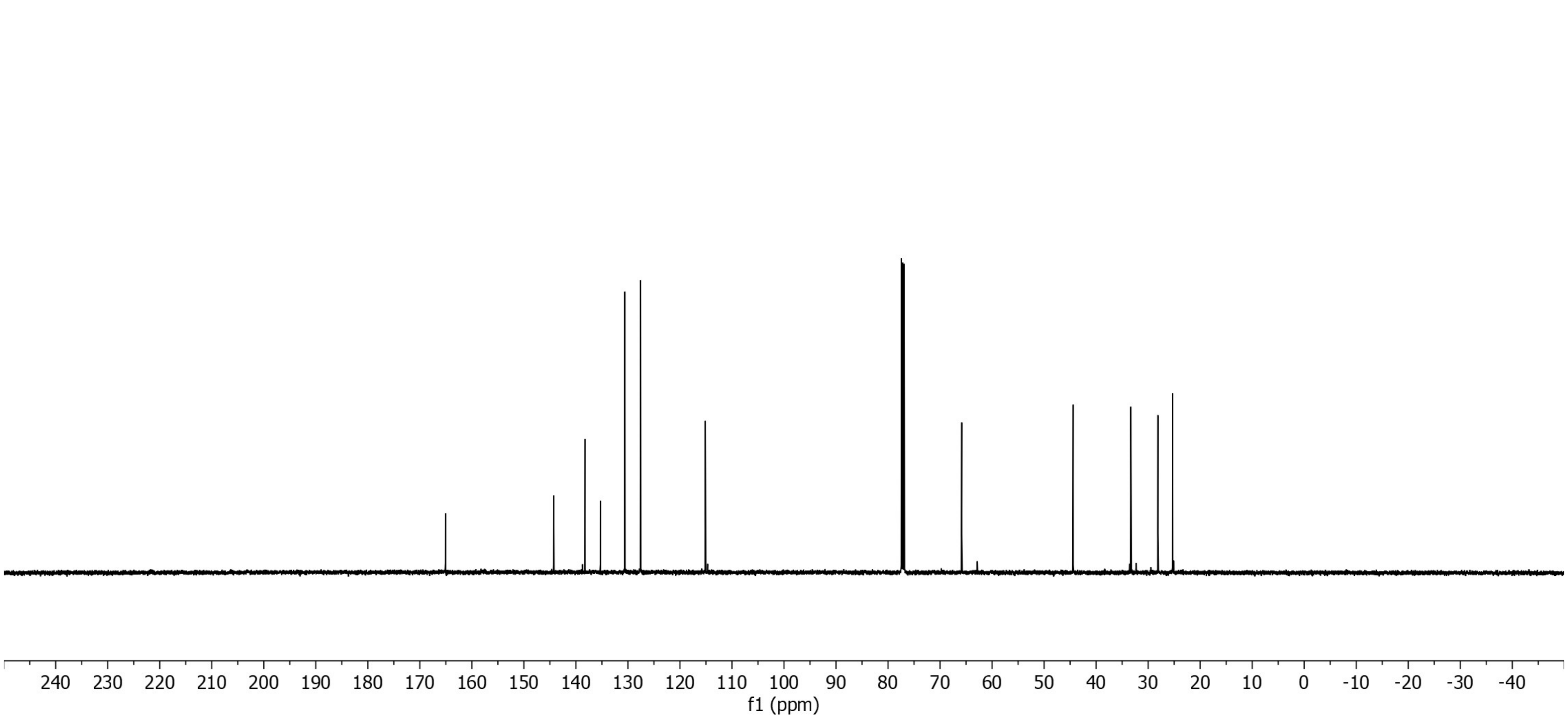

^{1}H NMR of alkenyl thianthrenium salt TT-2

$CDCl_3$, 500 MHz, 23 °C

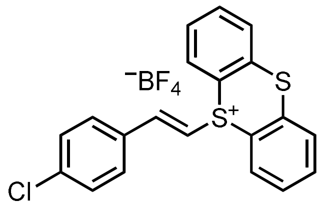

TT-2

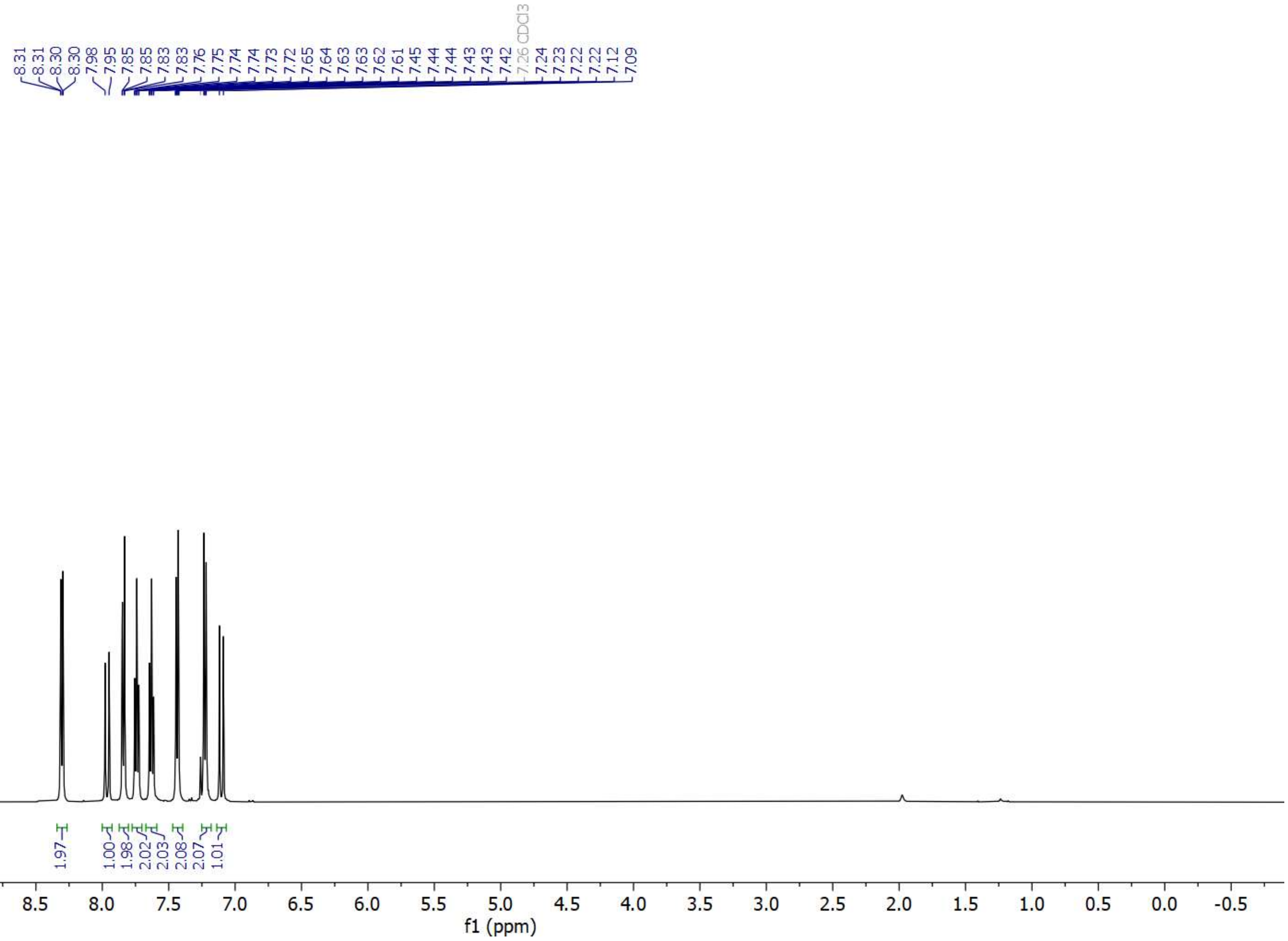

^{19}F NMR of alkenyl thianthrenium salt TT-2

CDCl3, 471 MHz, 23 °C

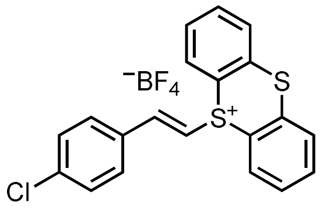

TT-2

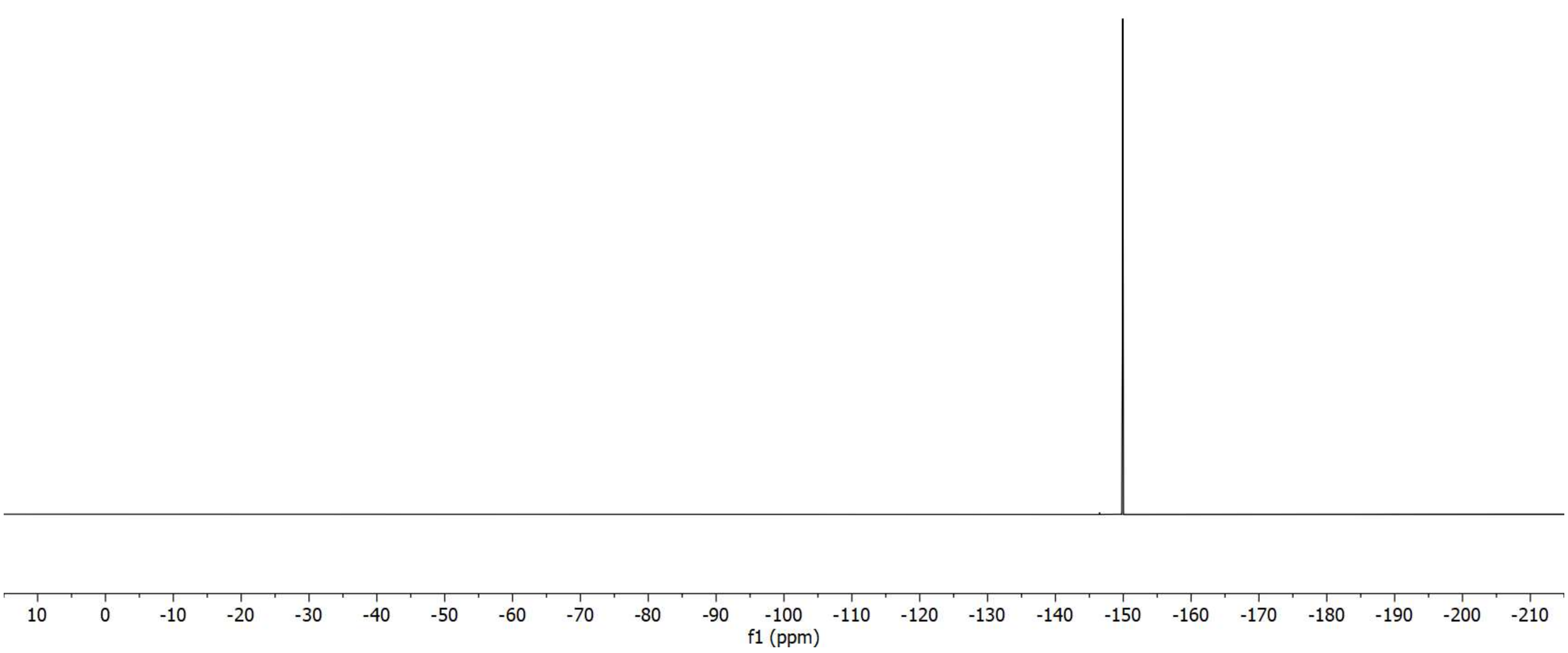

^{13}C NMR of alkenyl thianthrenium salt TT-2

$CDCl_3$, 126 MHz, 23 °C

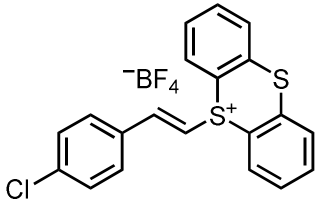

TT-2

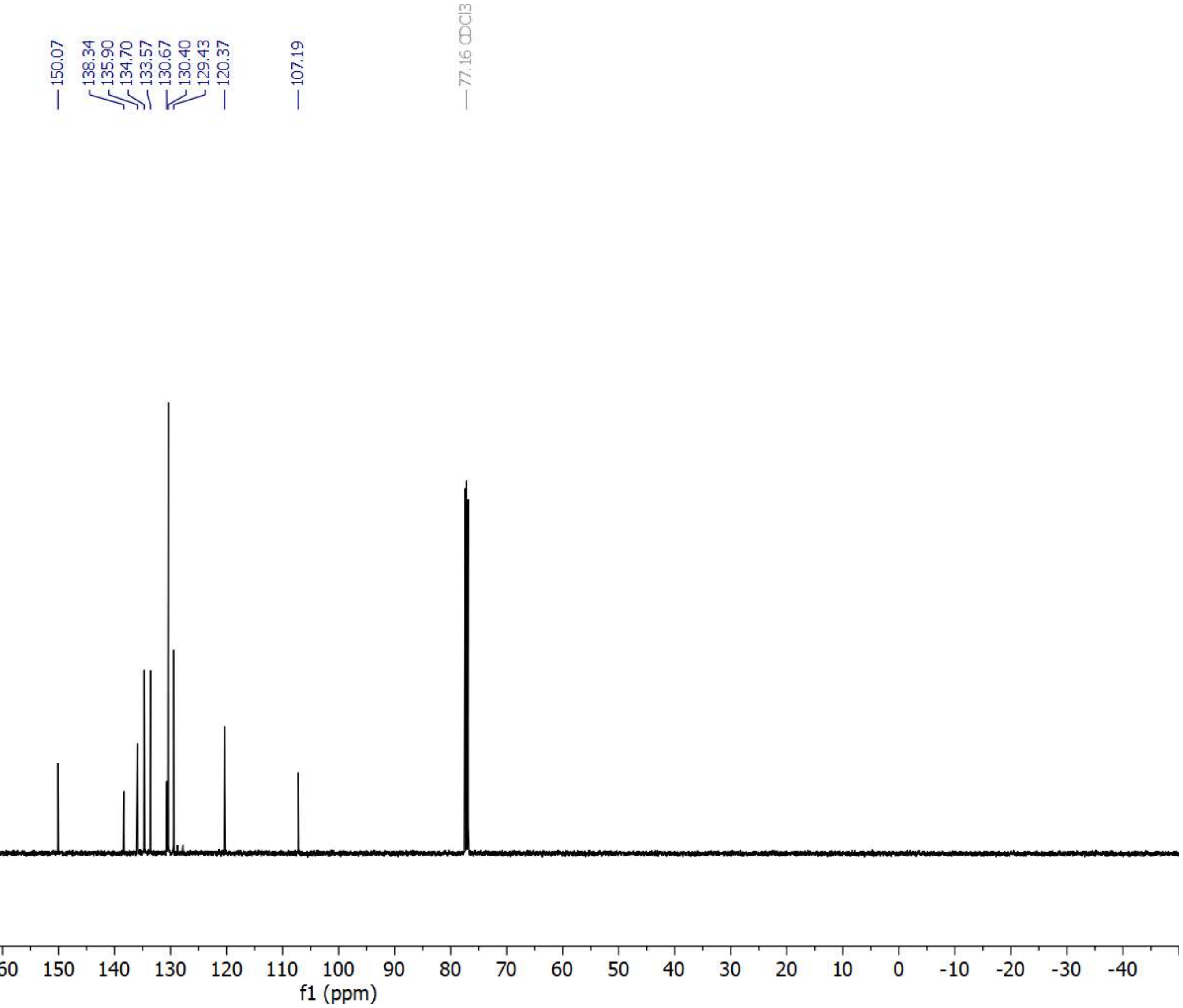

^{1}H NMR of alkenyl thianthrenium salt TT-7

$CDCl_3$, 500 MHz, 23 °C

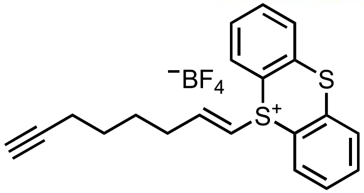

TT-7

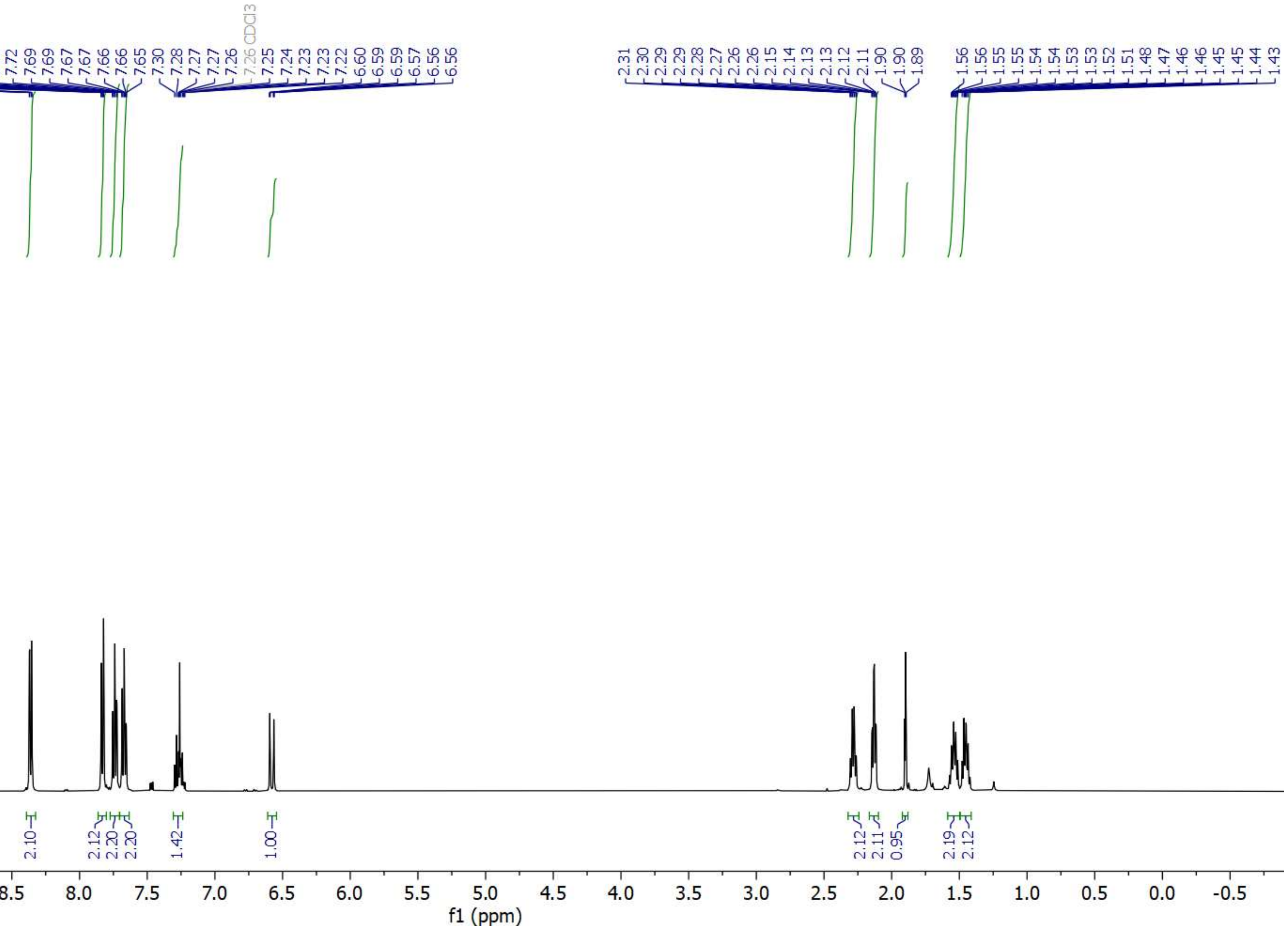

^{19}F NMR of alkenyl thianthrenium salt TT-7

CDCl$_3$, 471 MHz, 23 °C

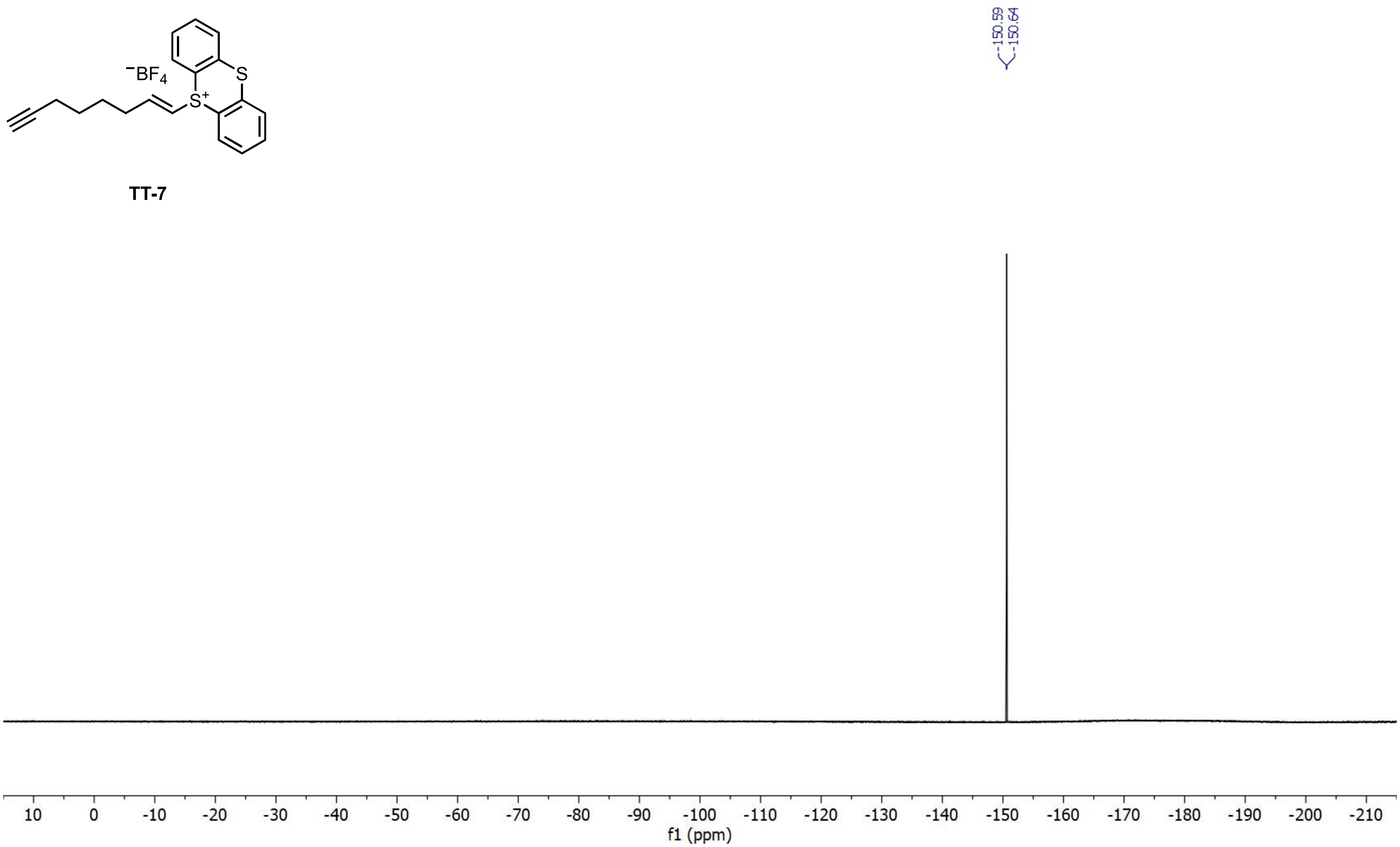

^{13}C NMR of alkenyl thianthrenium salt TT-7

CDCl$_3$, 471 MHz, 23 °C

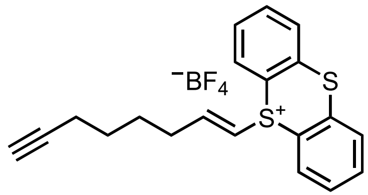

TT-7

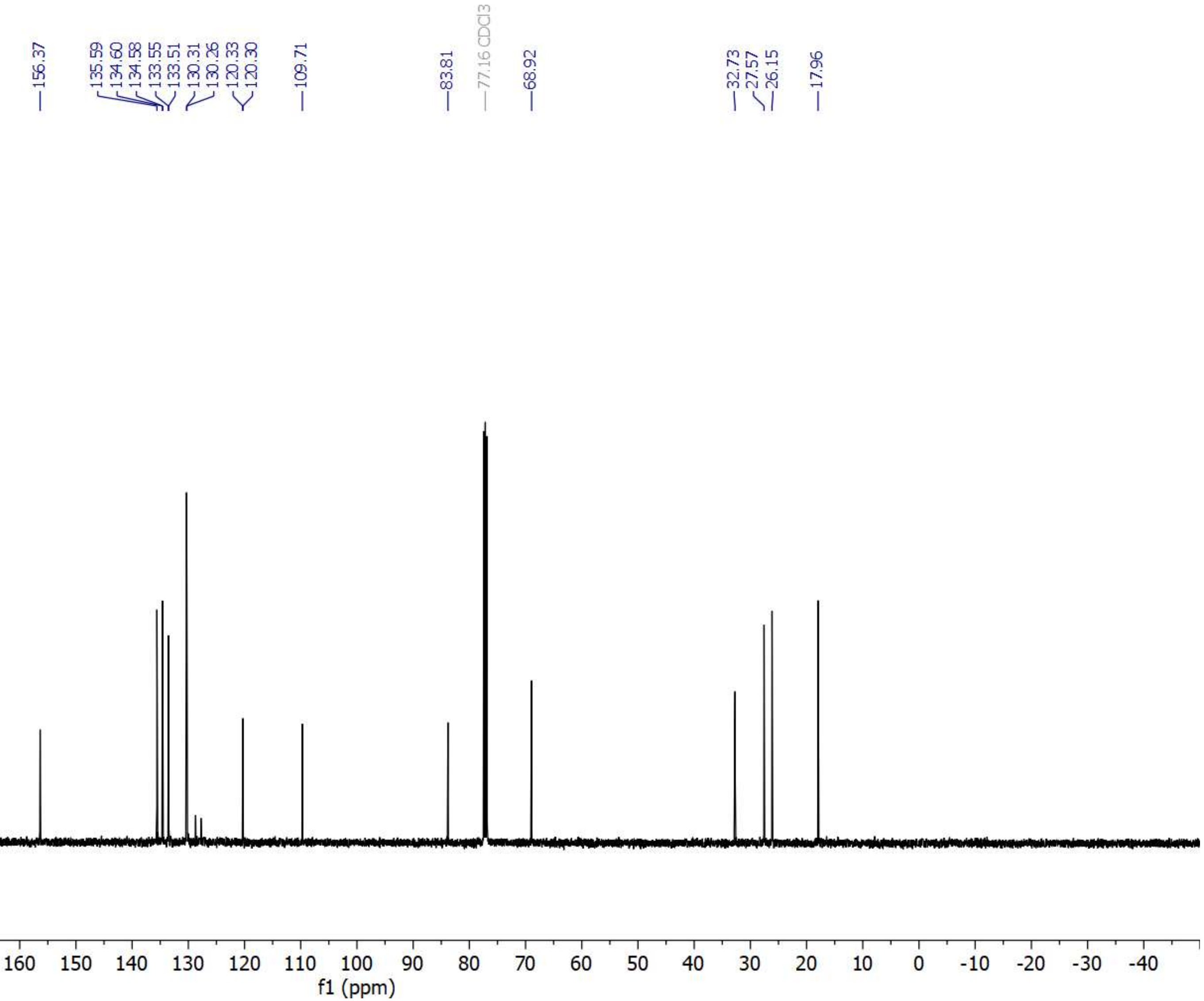

^{1}H NMR of alkenyl thianthrenium salt TT-8

CDCl$_3$, 500 MHz, 23 °C

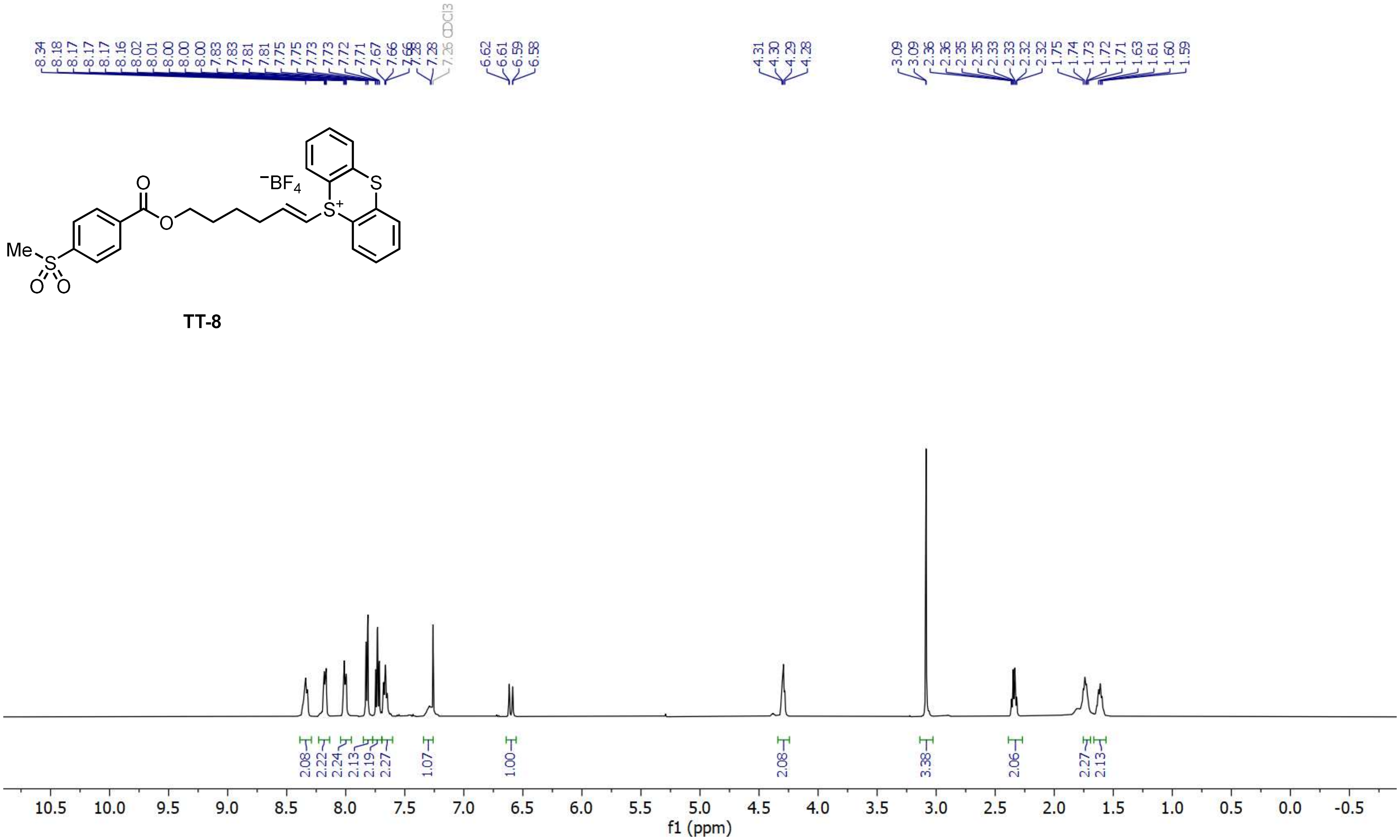

^{19}F NMR of alkenyl thianthrenium salt TT-8

CDCl$_3$, 471 MHz, 23 °C

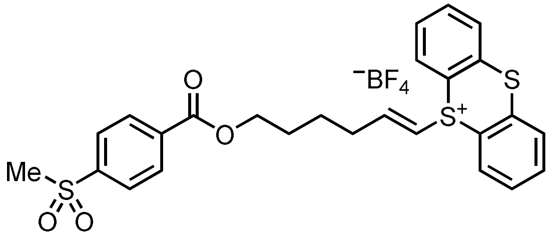

TT-8

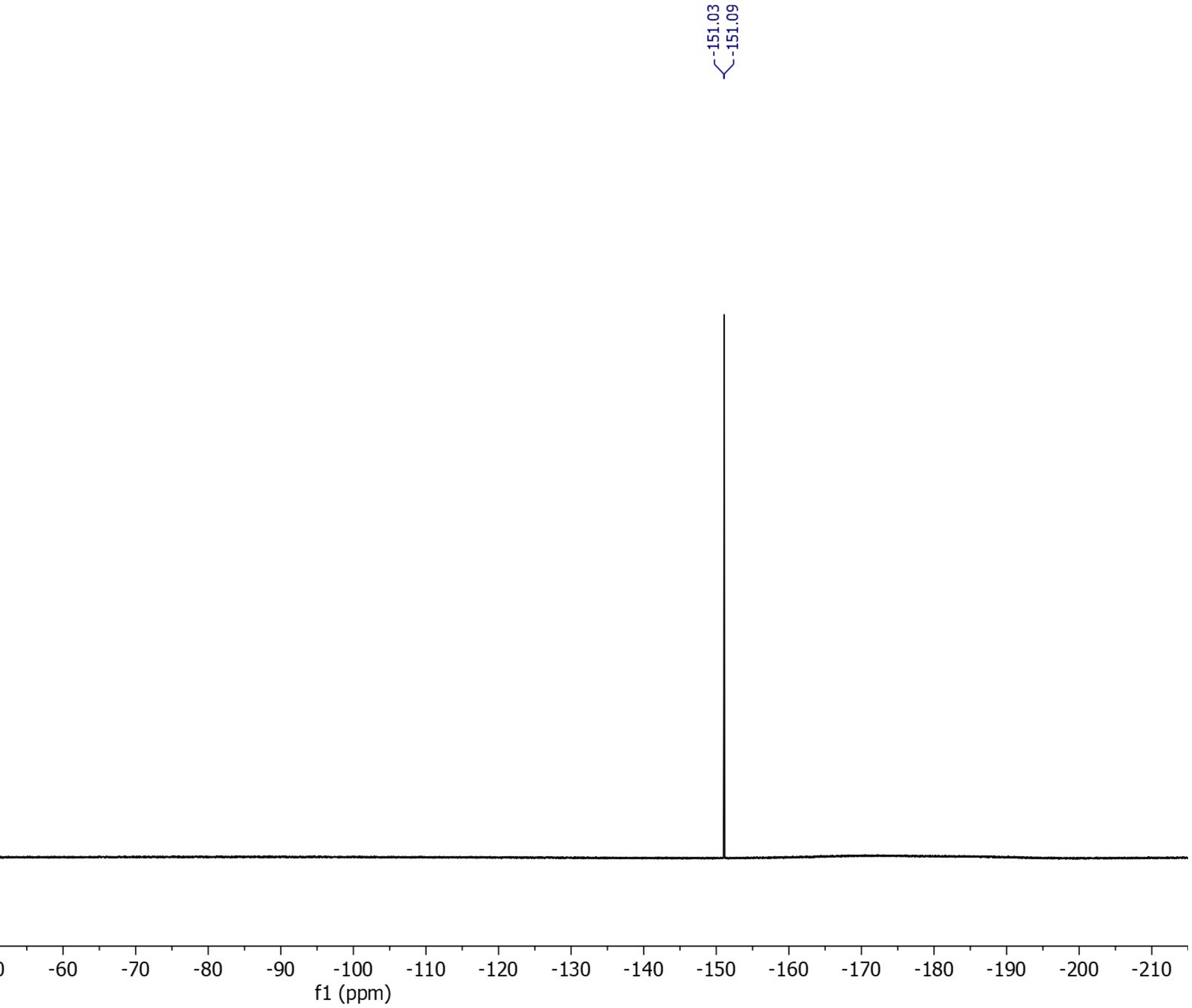

^{13}C NMR of alkenyl thianthrenium salt TT-8

CDCl3, 126 MHz, 23 °C

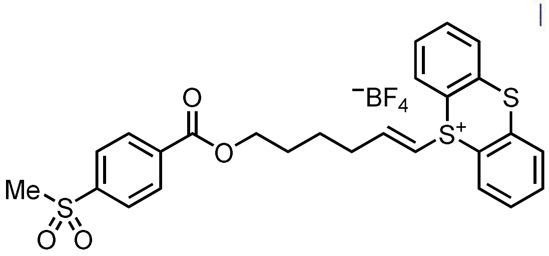

TT-8

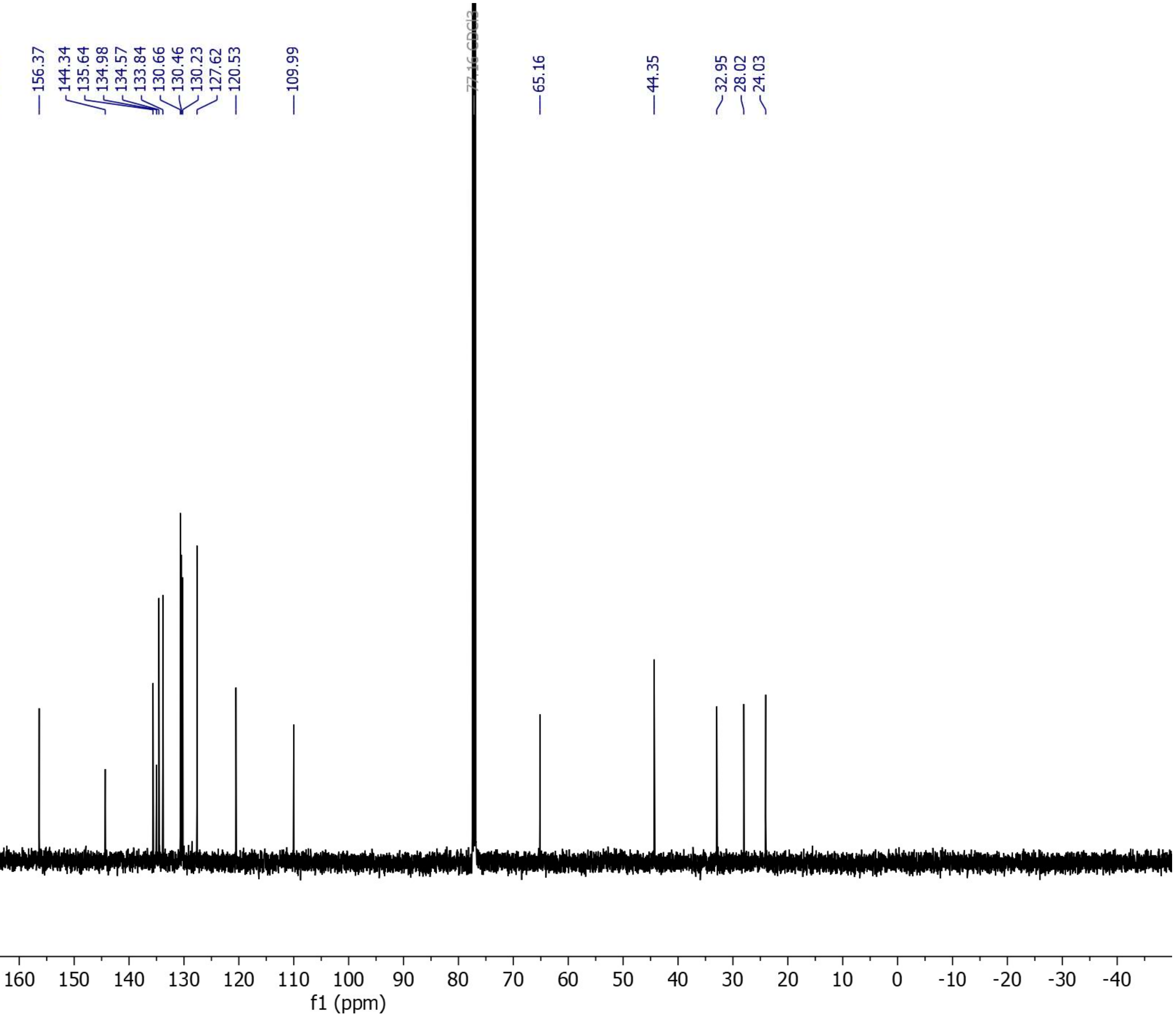

^{1}H NMR of alkenyl thianthrenium salt (±)-TT-12

CDCl₃, 500 MHz, 23 °C

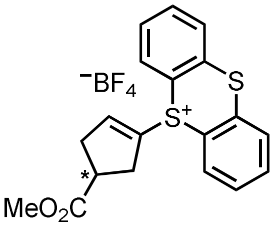

(±)-**TT-12**

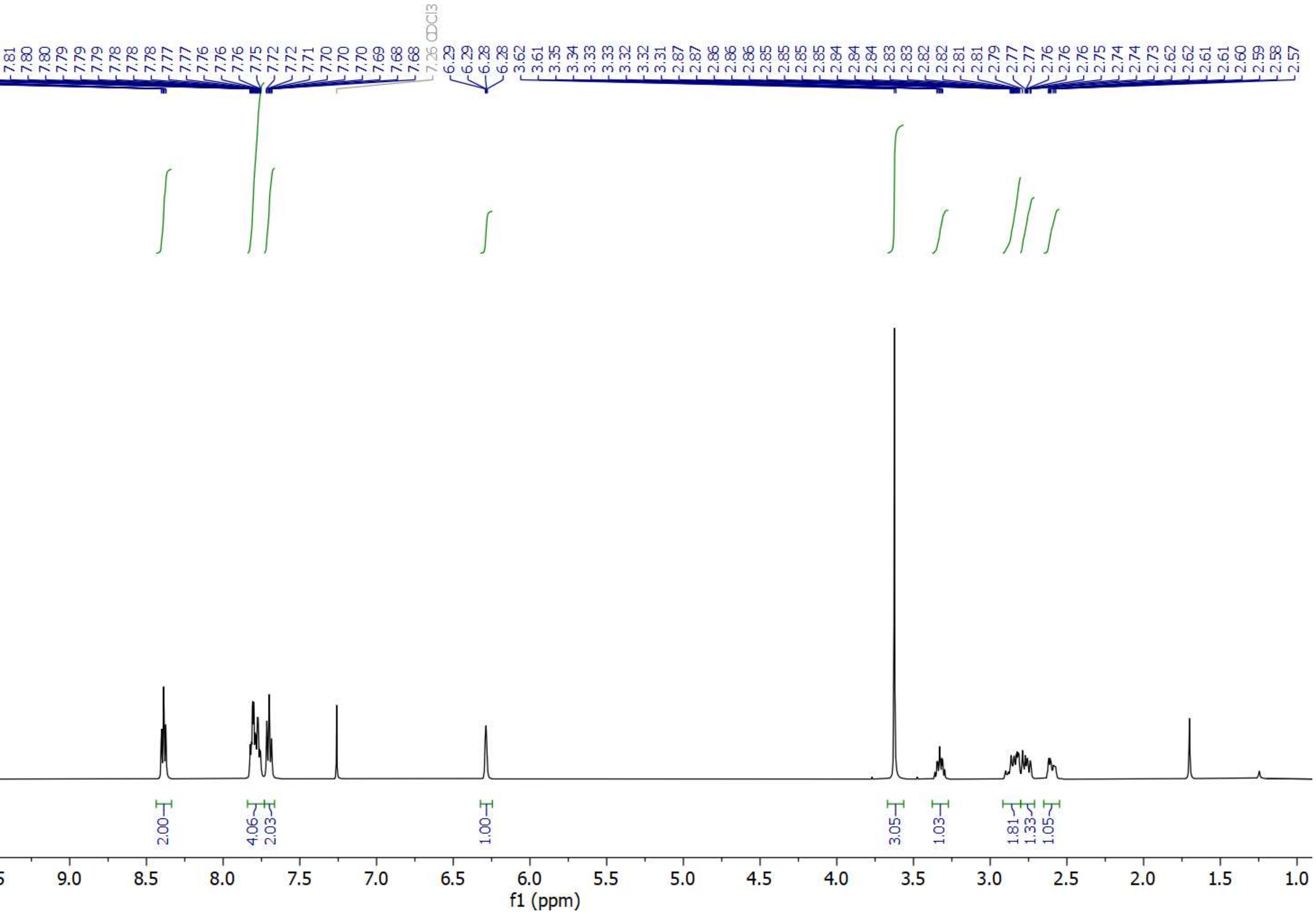

^{19}F NMR of alkenyl thianthrenium salt (±)-TT-12

$CDCl_3$, 471 MHz, 23 °C

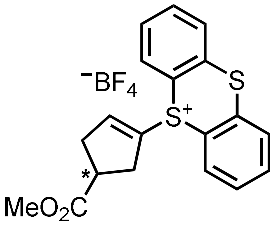

(±)-**TT-12**

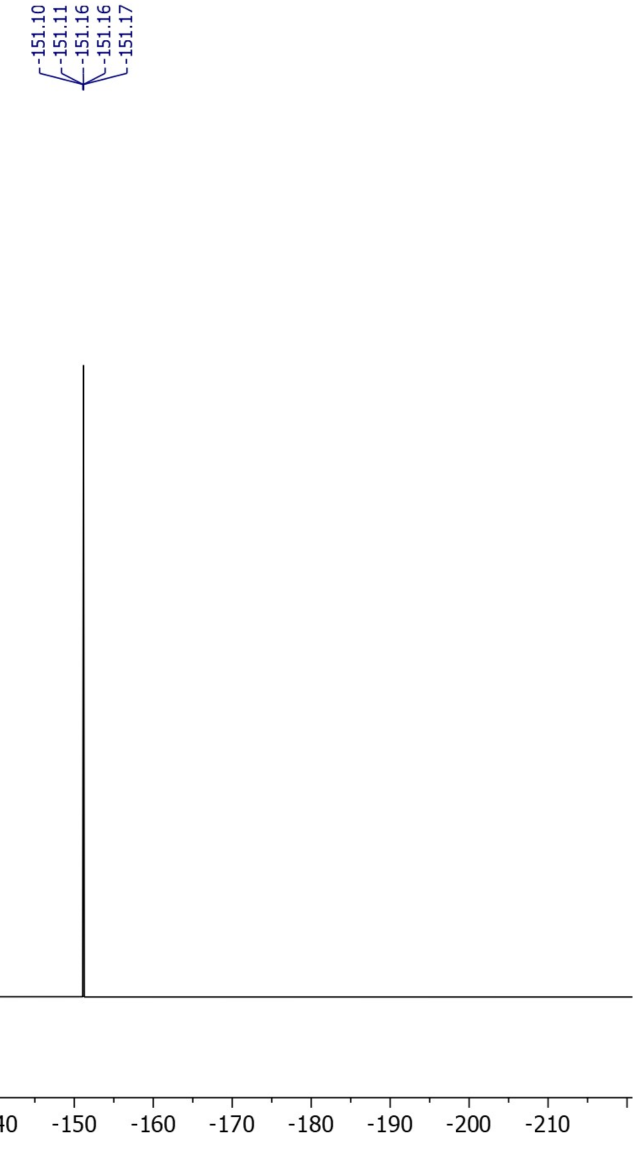

^{13}C NMR of alkenyl thianthrenium salt (±)-TT-12

$CDCl_3$, 126 MHz, 23 °C

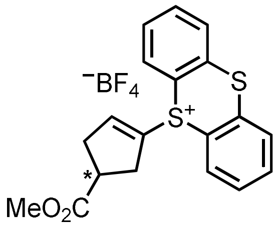

(±)-**TT-12**

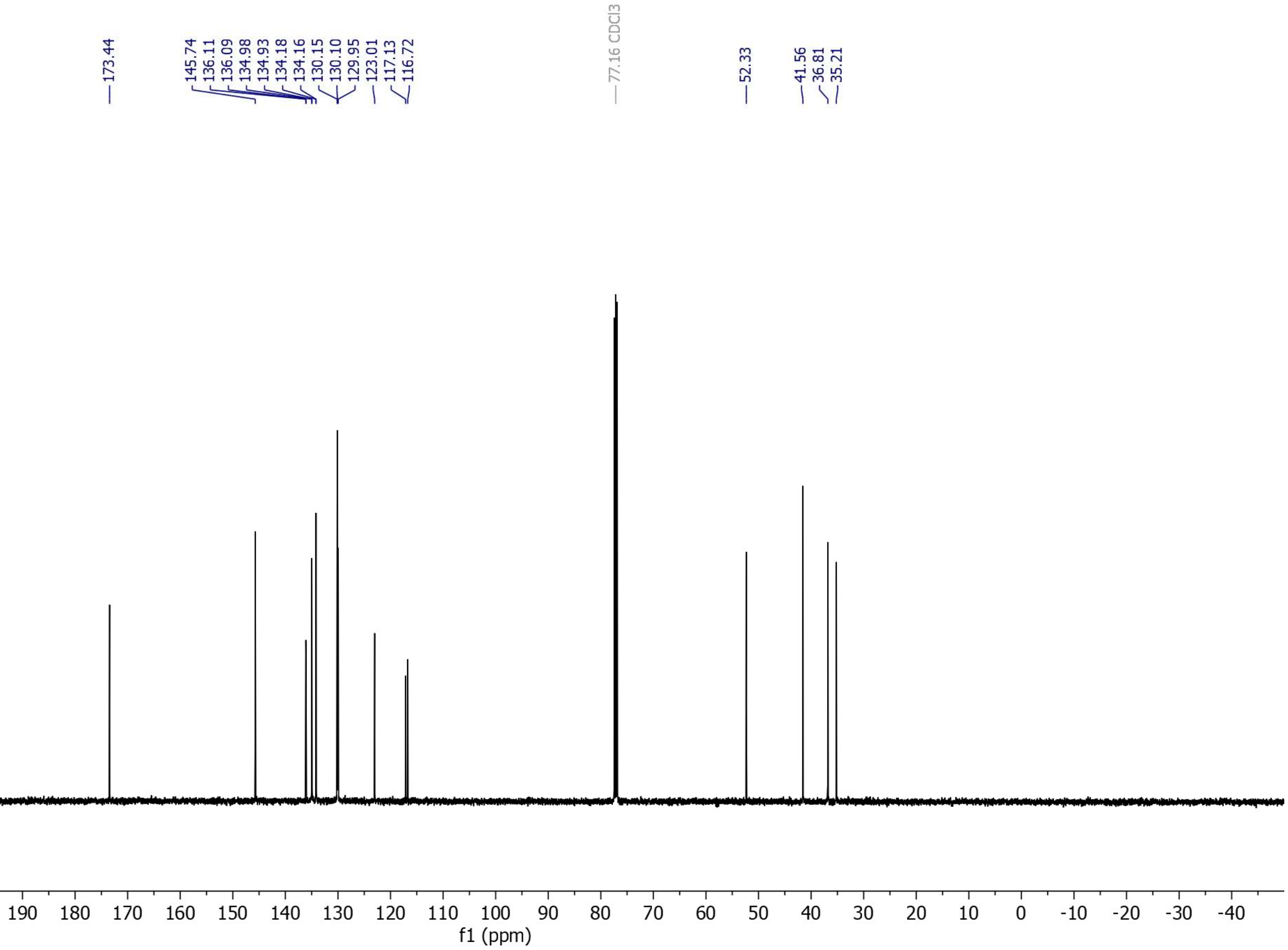

^{1}H NMR of alkenyl thianthrenium salt TT-15

CDCl$_3$, 500 MHz, 23 °C

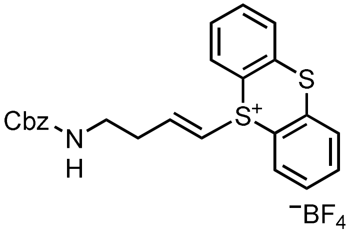

TT-15

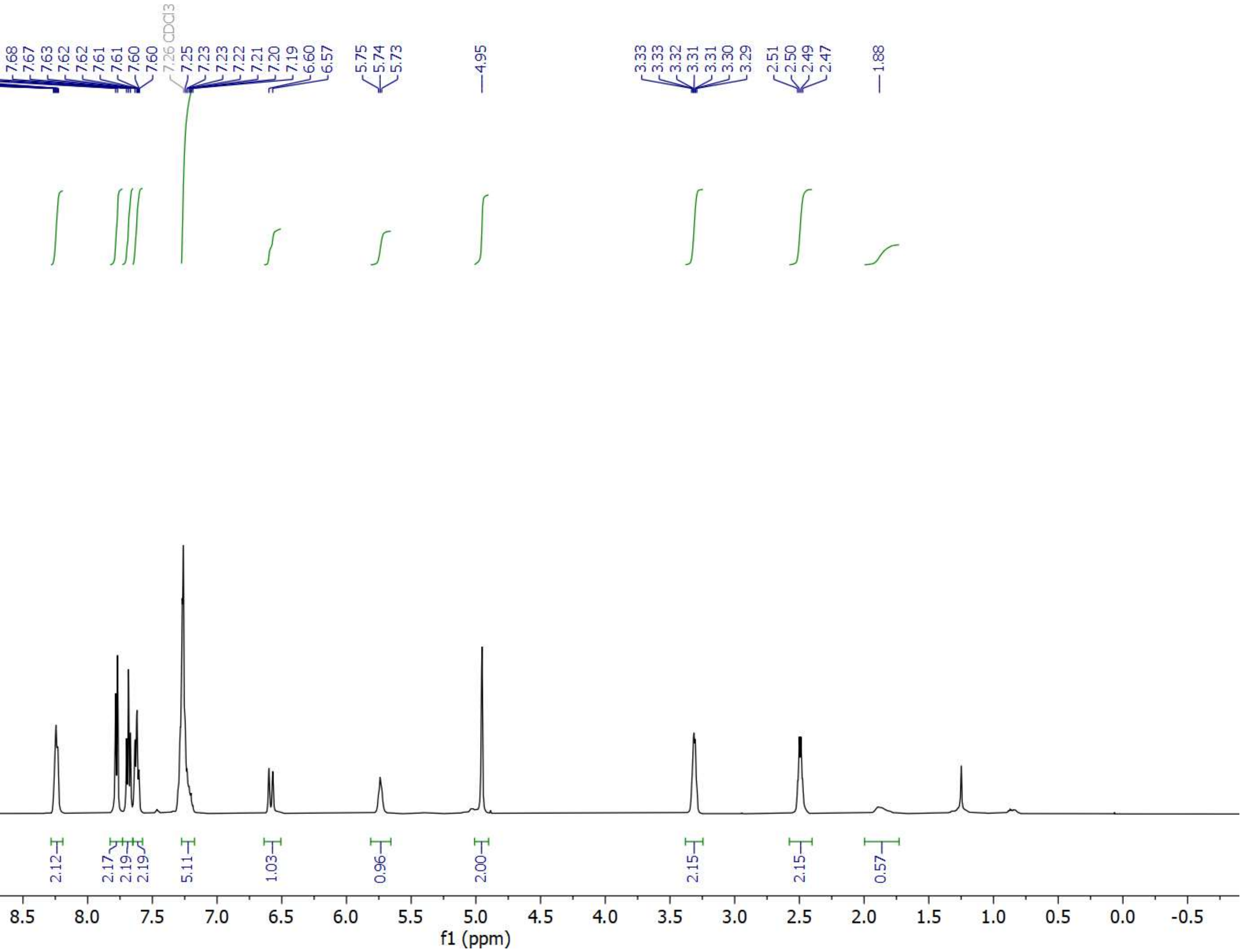

^{19}F NMR of alkenyl thianthrenium salt TT-15

$CDCl_3$, 471 MHz, 23 °C

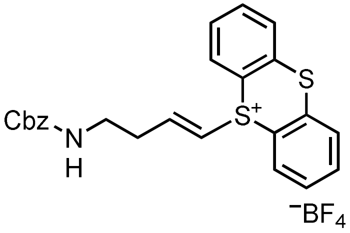

TT-15

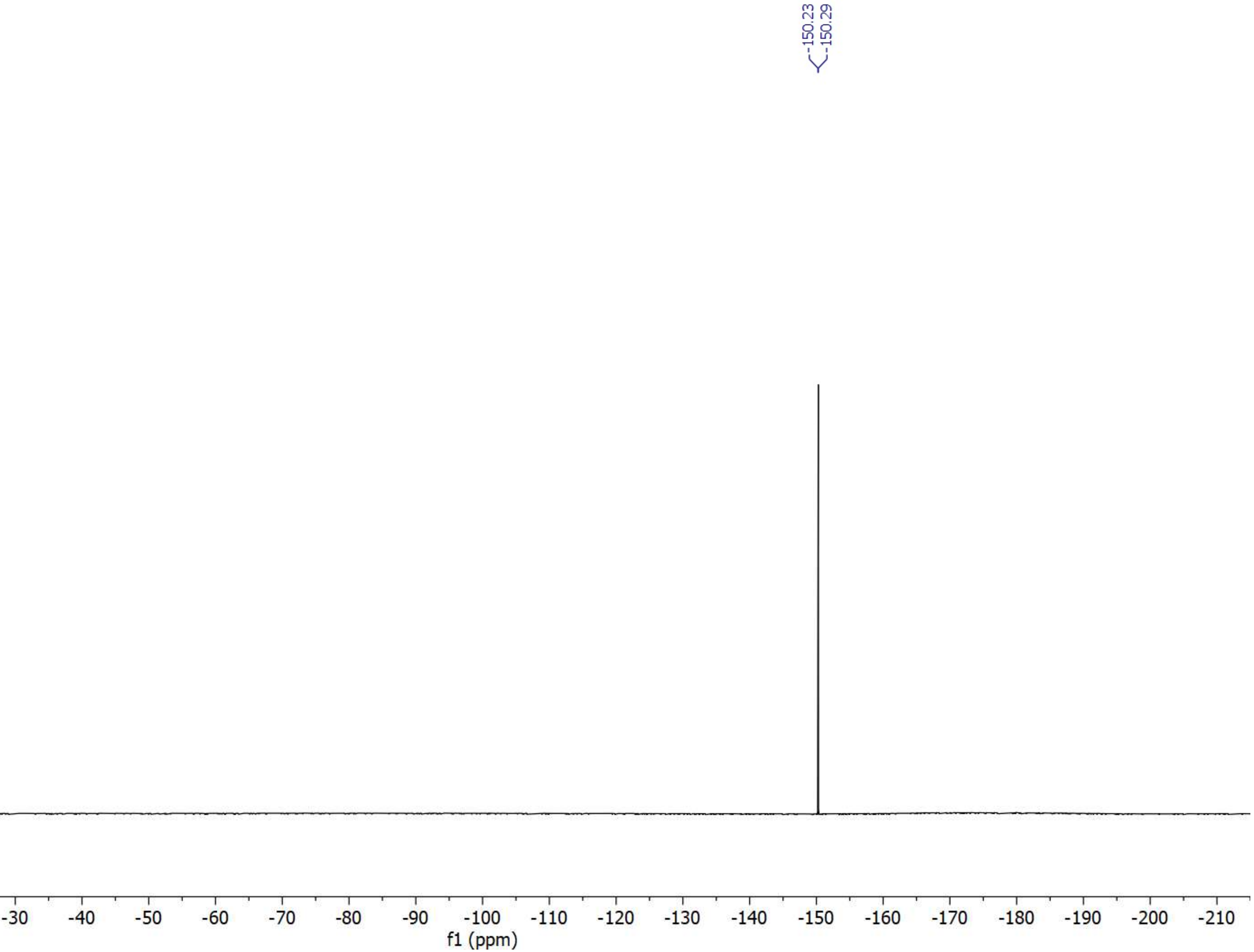

^{13}C NMR of alkenyl thianthrenium salt TT-15

$CDCl_3$, 126 MHz, 23 °C

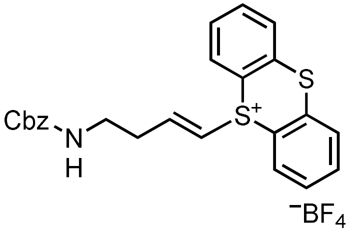

TT-15

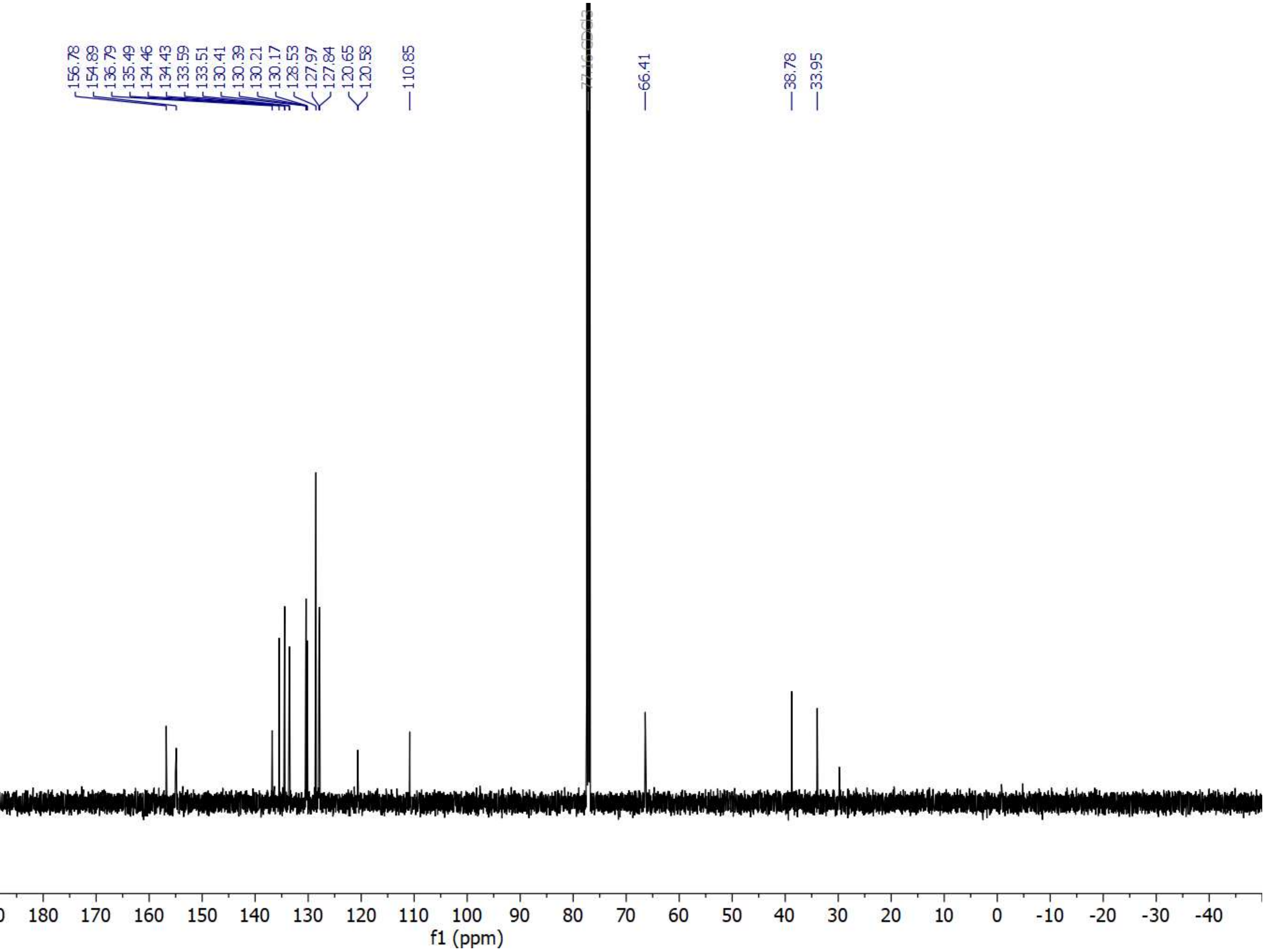

^{1}H NMR of alkenyl thianthrenium salt TT-16

$CDCl_3$, 600 MHz, 23 °C

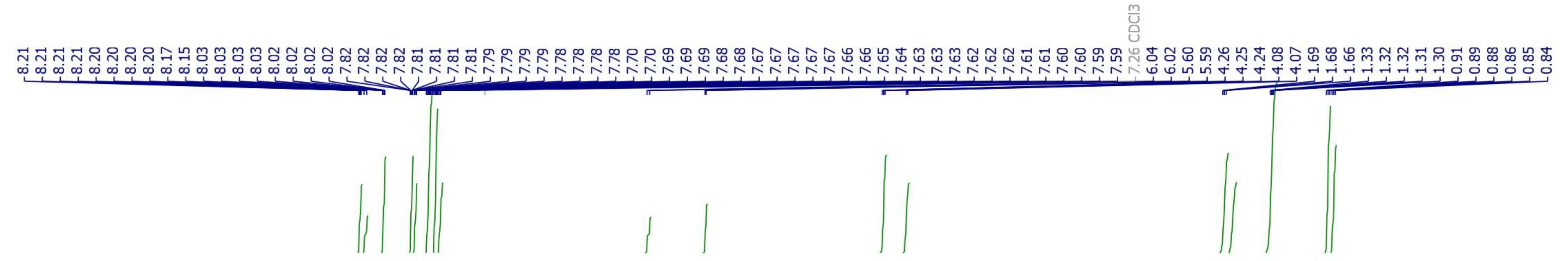

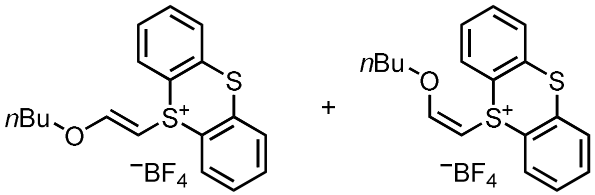

minor

major

TT-16

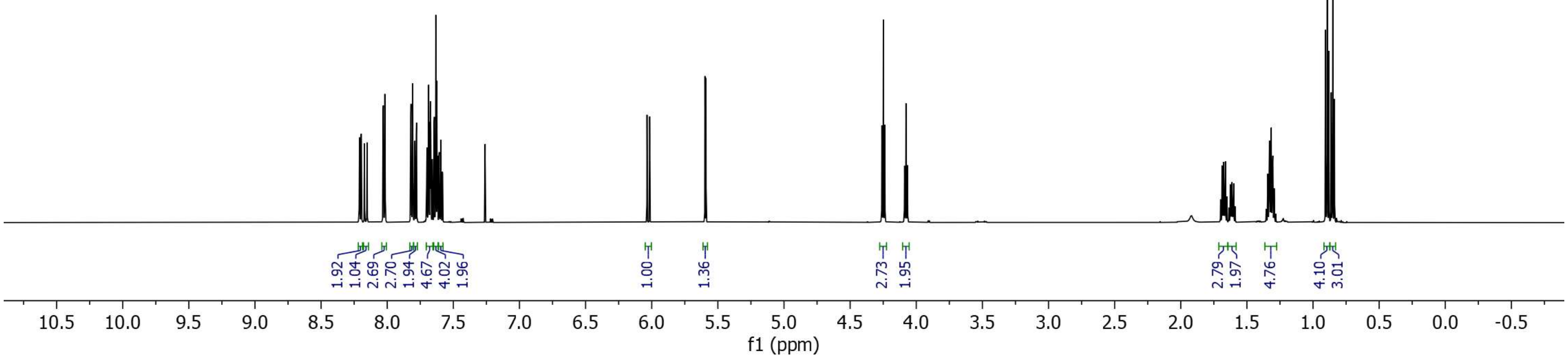

^{19}F NMR of alkenyl thianthrenium salt TT-16

CDCl$_3$, 471 MHz, 23 °C

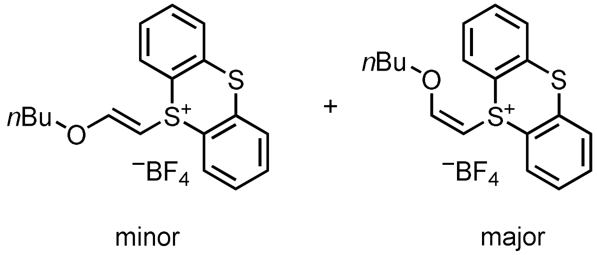

TT-16

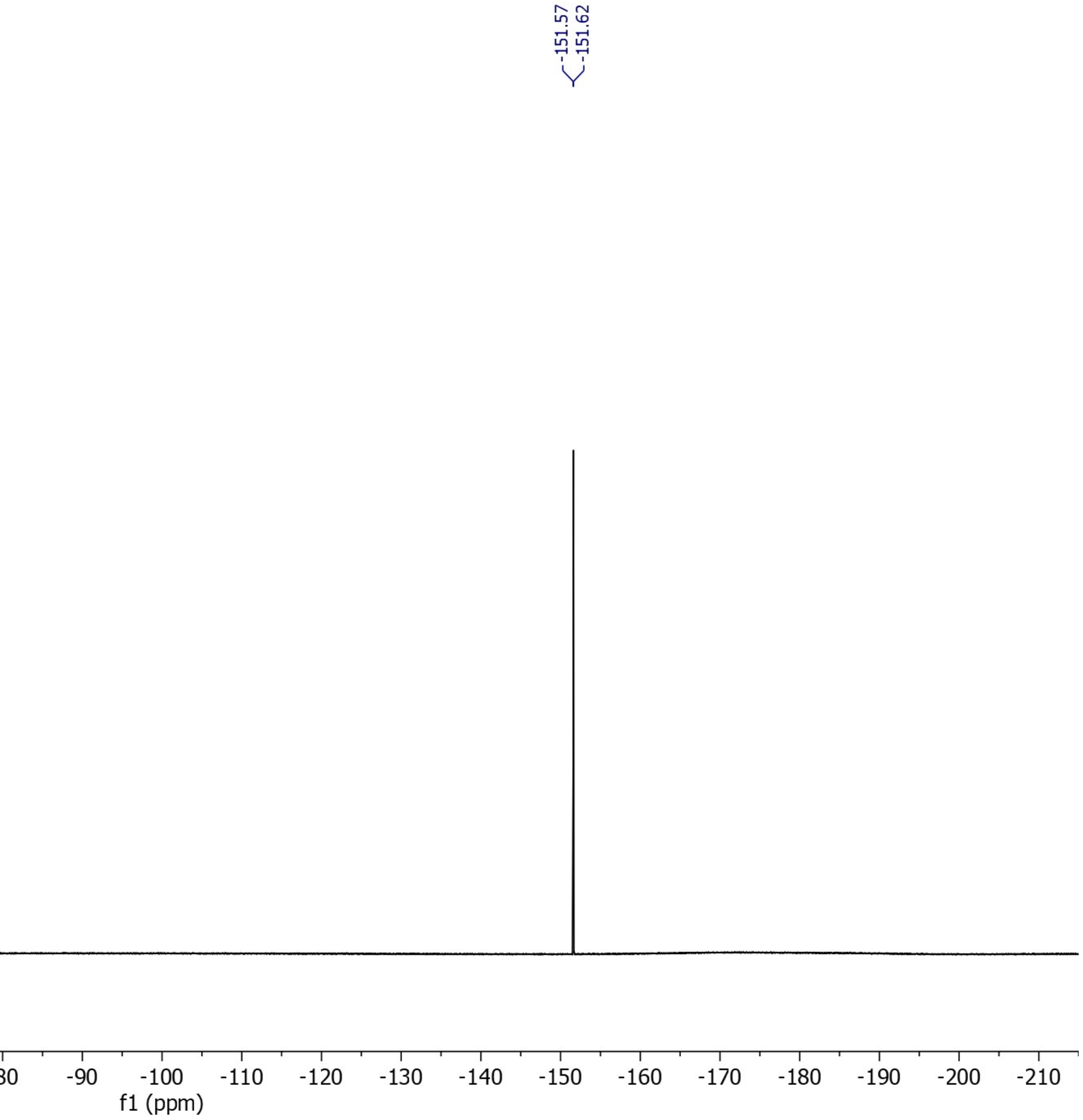

^{13}C NMR of alkenyl thianthrenium salt TT-16

$CDCl_3$, 151 MHz, 23 °C

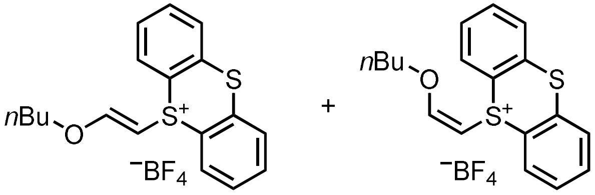

minor **TT-16** major

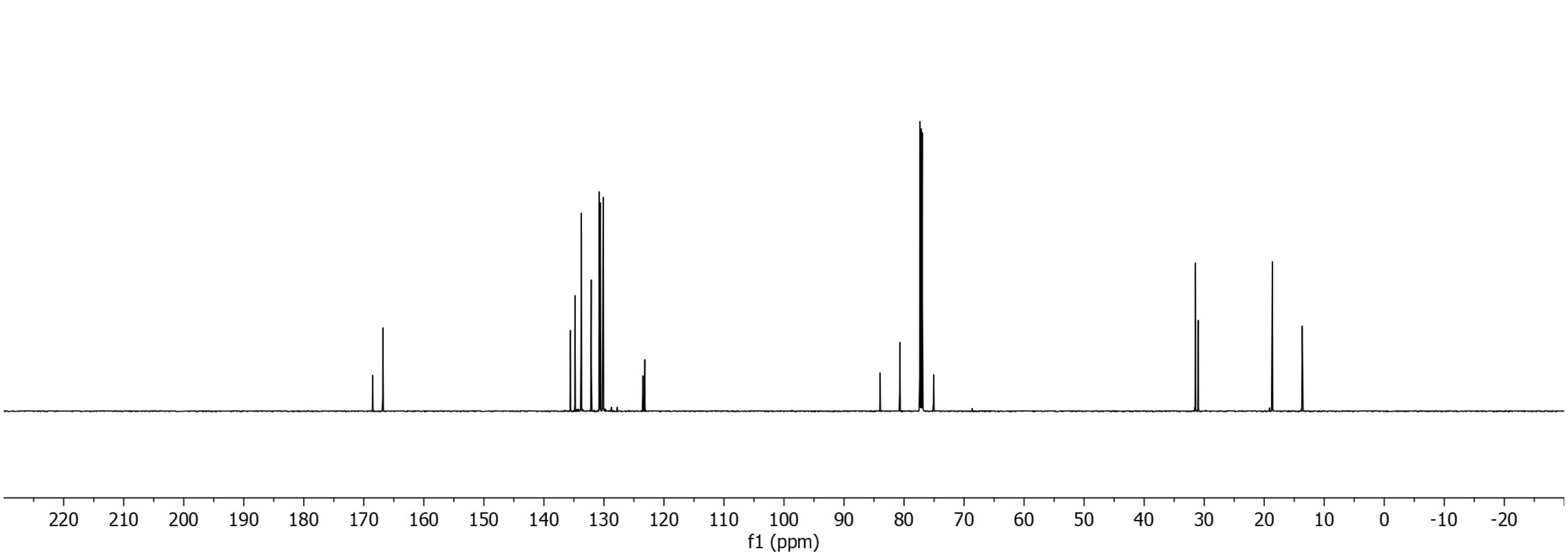

^{1}H NMR of alkenyl thianthrenium salt TT-21

$CDCl_3$, 500 MHz, 23 °C

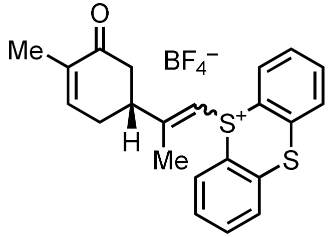

TT-21

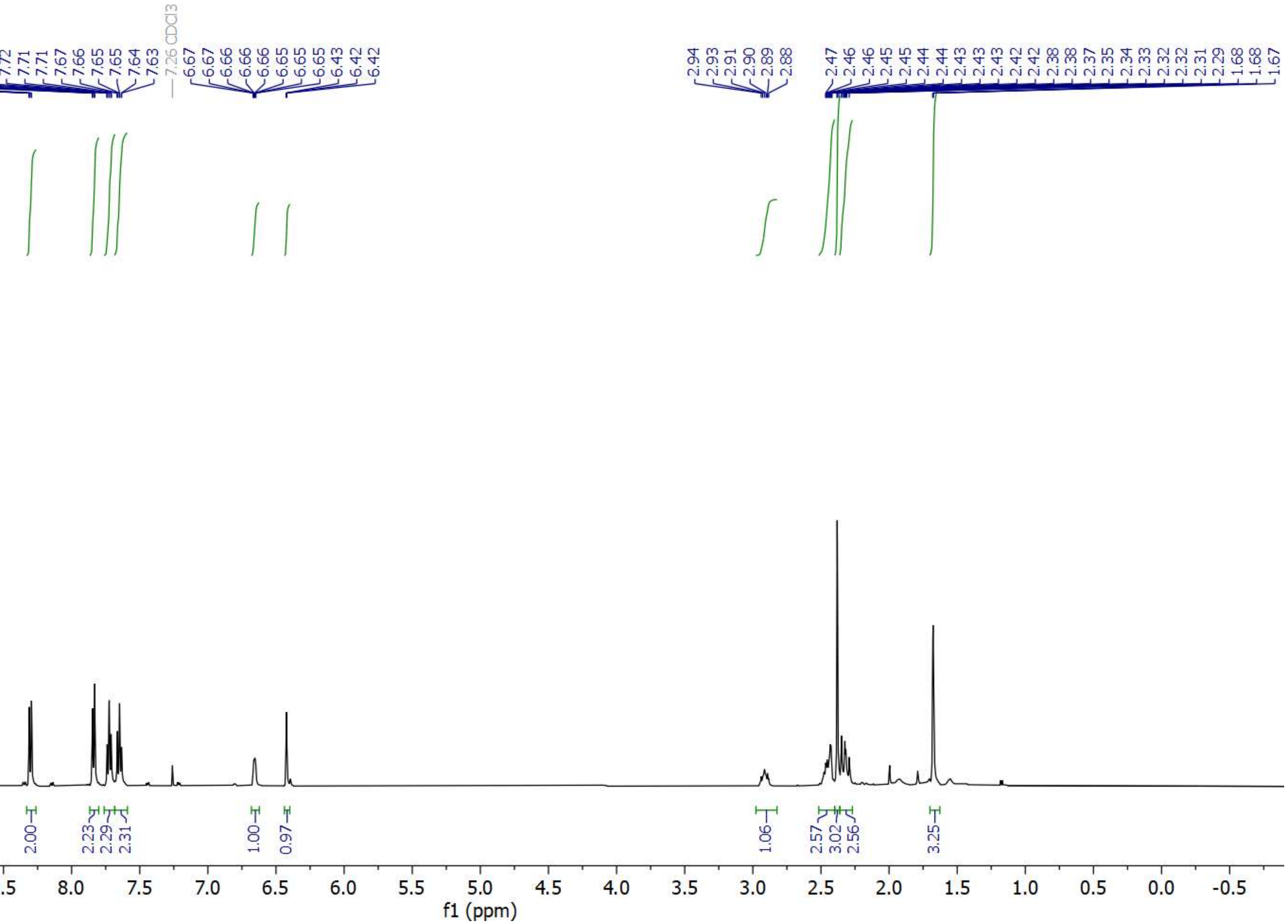

^{19}F NMR of alkenyl thianthrenium salt TT-21

$CDCl_3$, 471 MHz, 23 °C

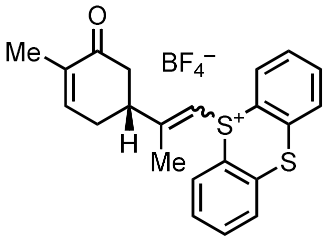

TT-21

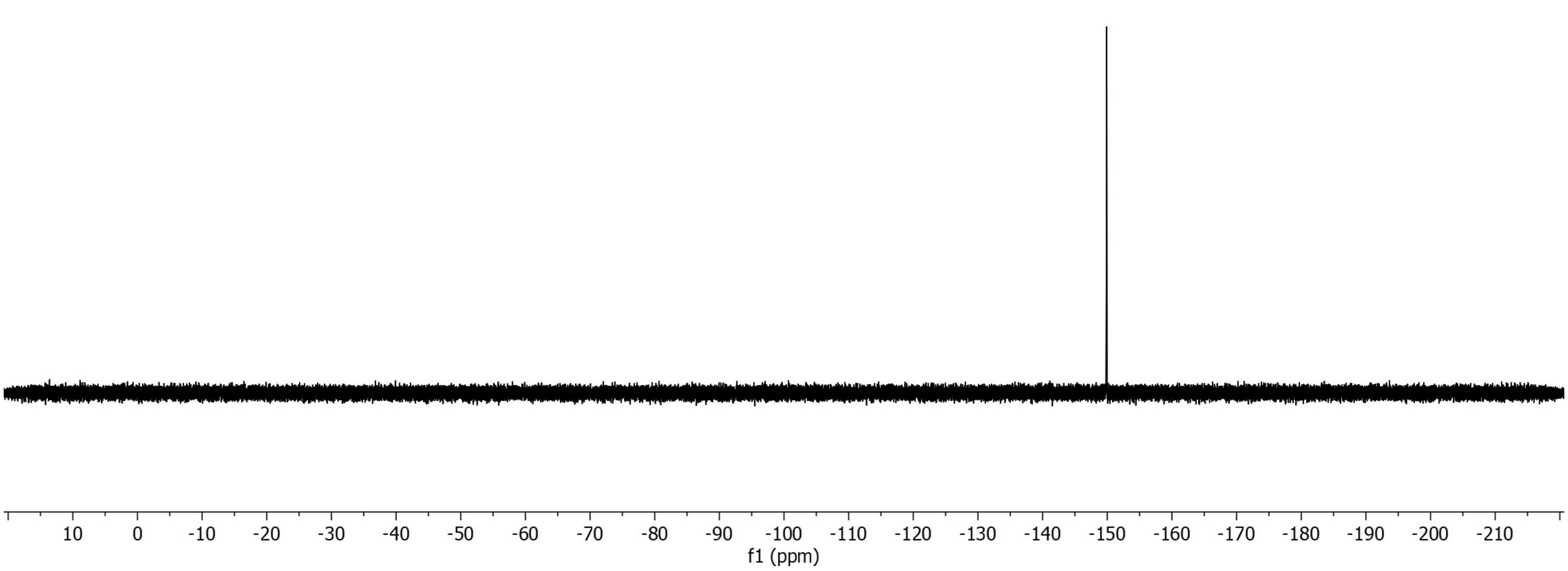

^{13}C NMR of alkenyl thianthrenium salt TT-21

$CDCl_3$, 126 MHz, 23 °C

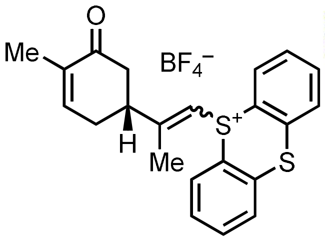

TT-21

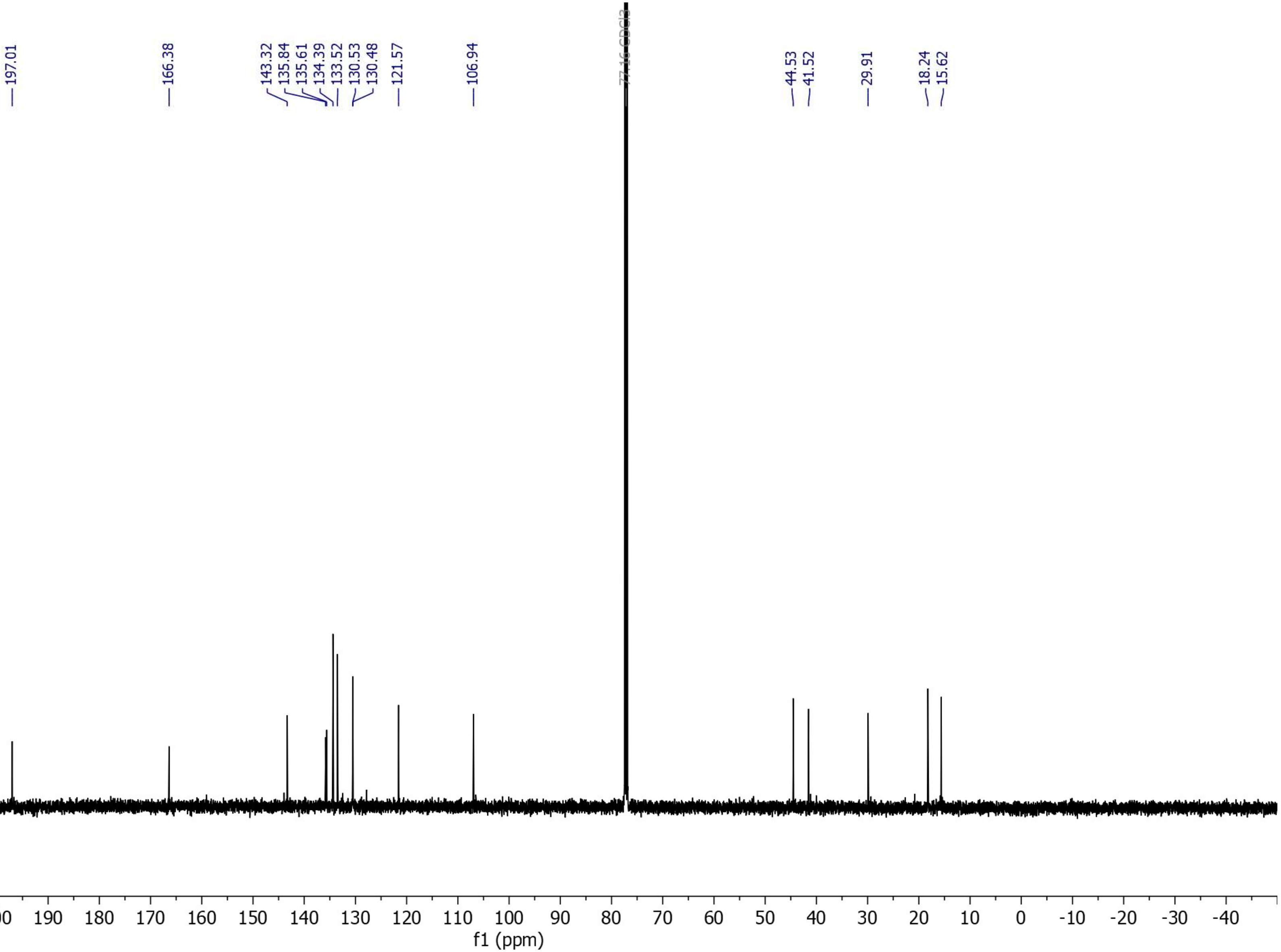

^{1}H NMR of alkenyl thianthrenium salt TT-22

$CDCl_3$, 500 MHz, 23 °C

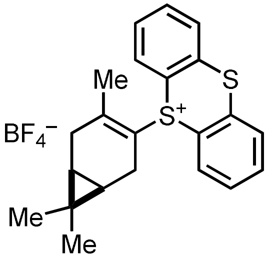

TT-22

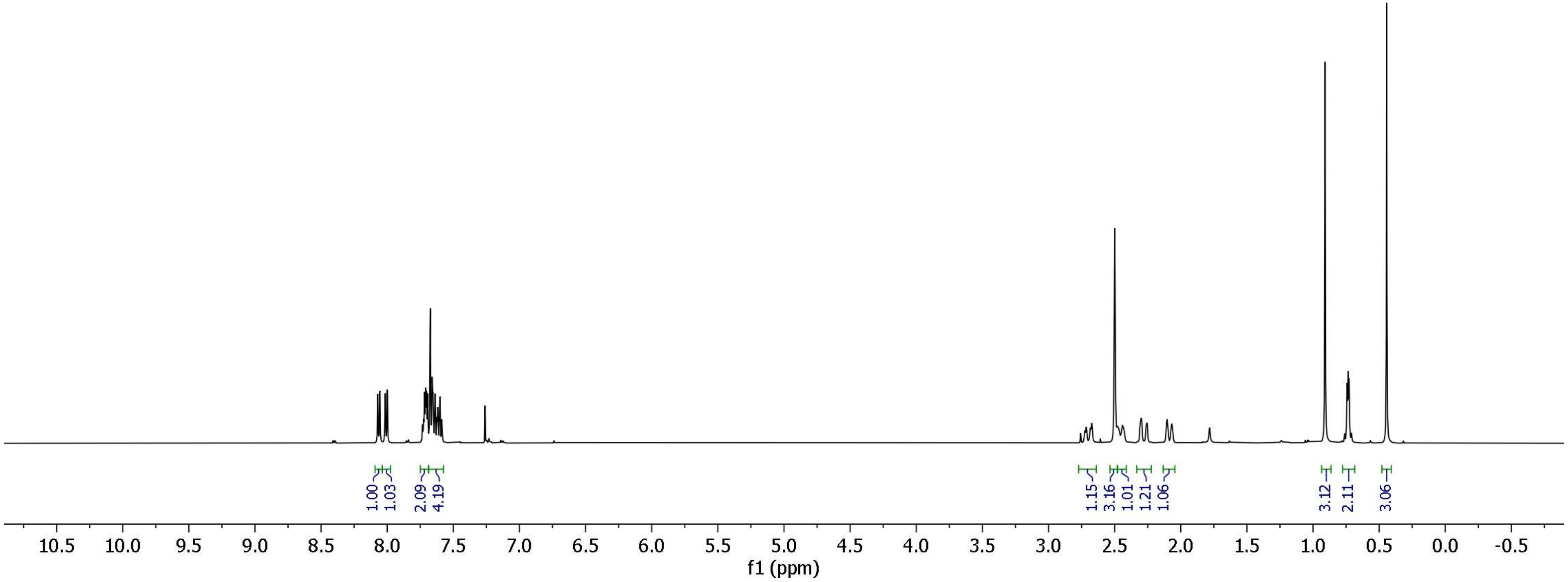

^{19}F NMR of alkenyl thianthrenium salt TT-22

$CDCl_3$, 471 MHz, 23 °C

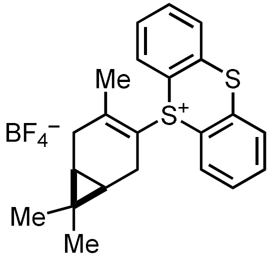

TT-22

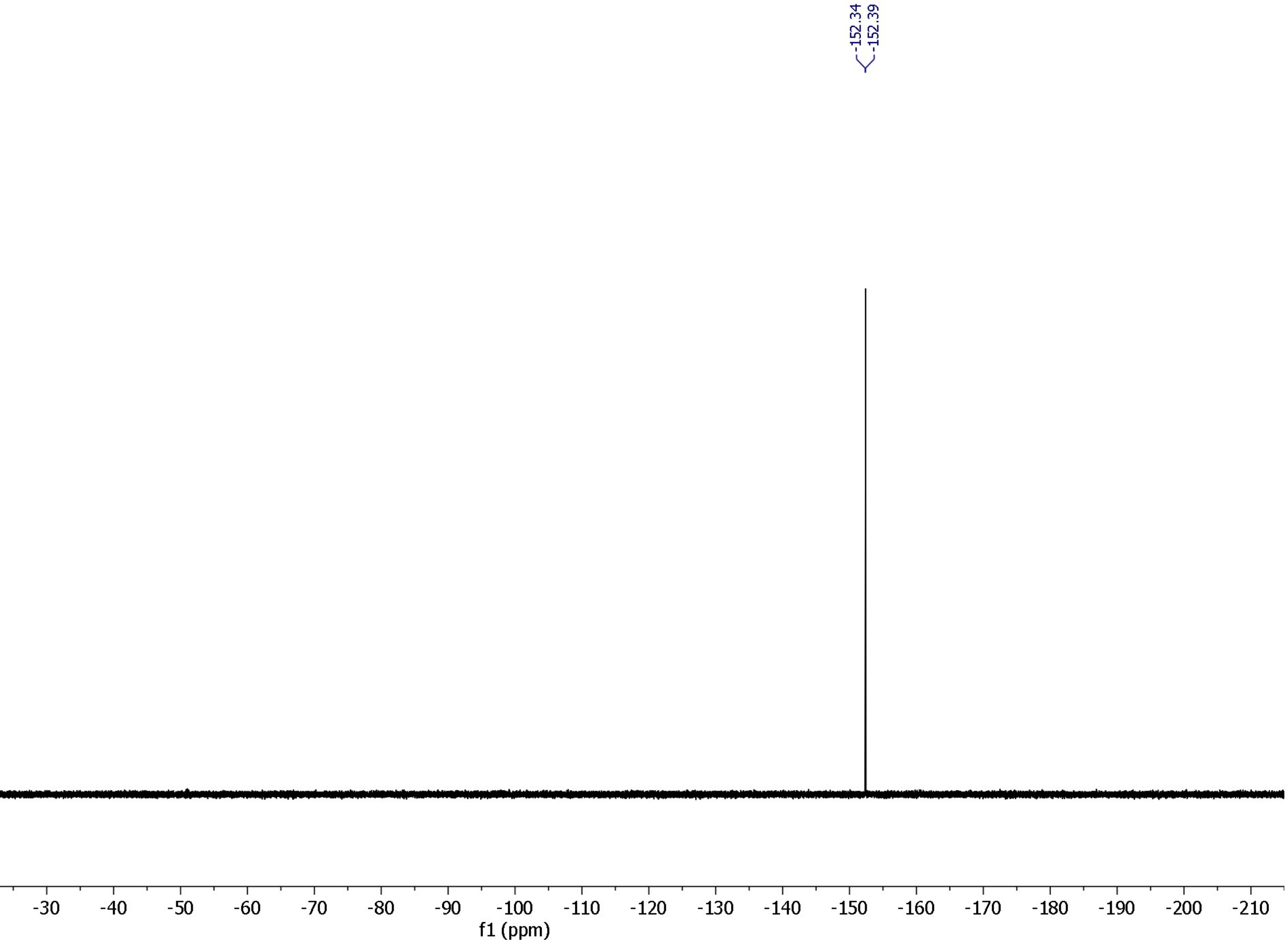

^{13}C NMR of alkenyl thianthrenium salt TT-22

$CDCl_3$, 126 MHz, 23 °C

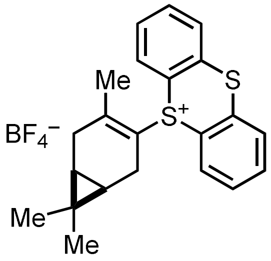

TT-22

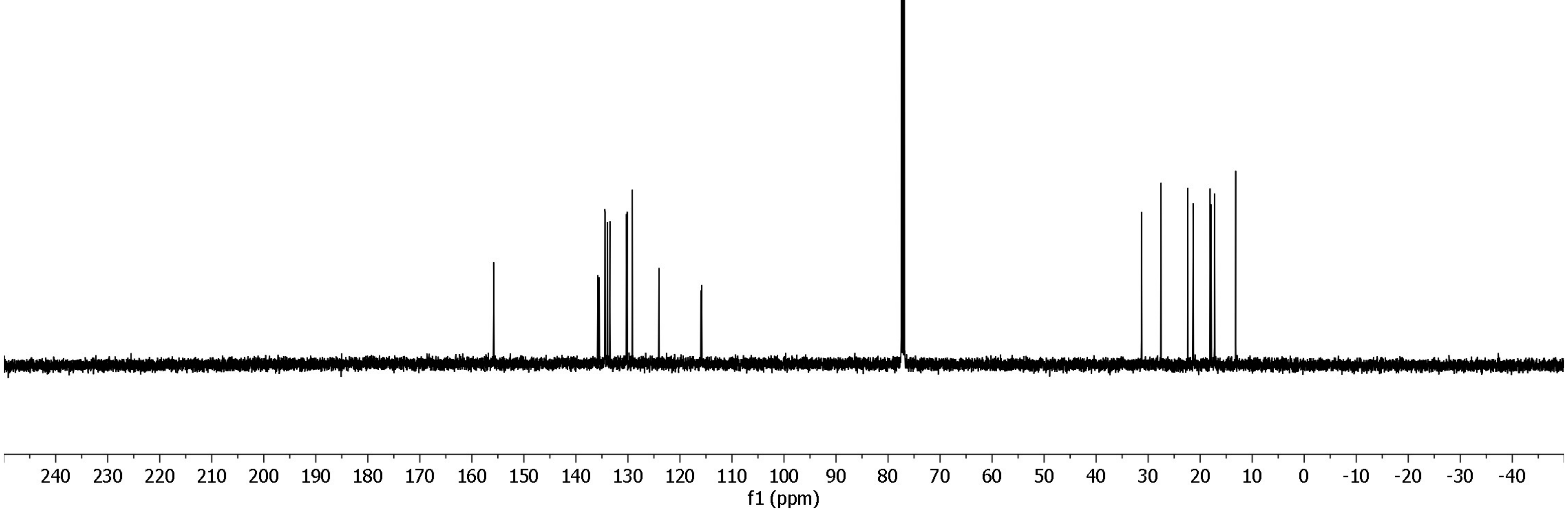

^{1}H NMR of alkenyl thianthrenium salt TT-23

$CDCl_3$, 500 MHz, 23 °C

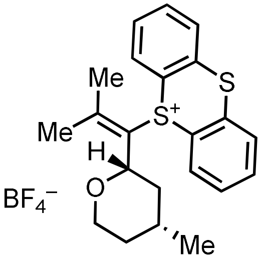

TT-23

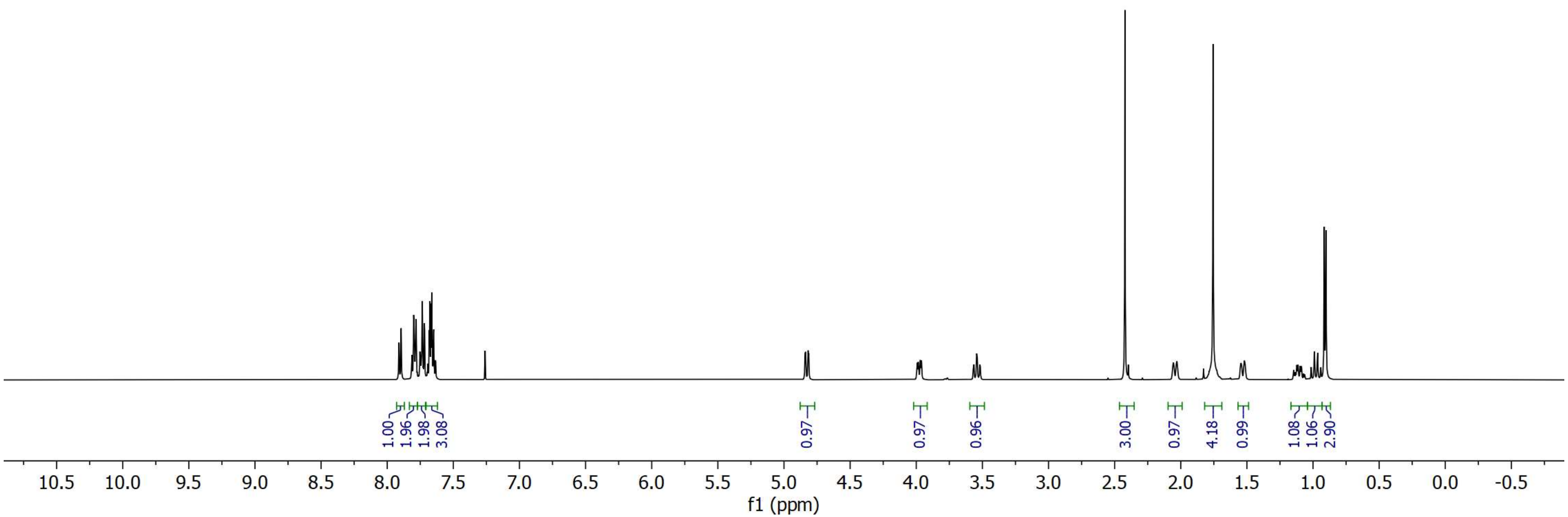

^{19}F NMR of alkenyl thianthrenium salt TT-23

$CDCl_3$, 471 MHz, 23 °C

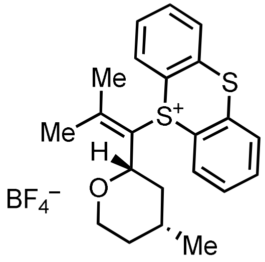

TT-23

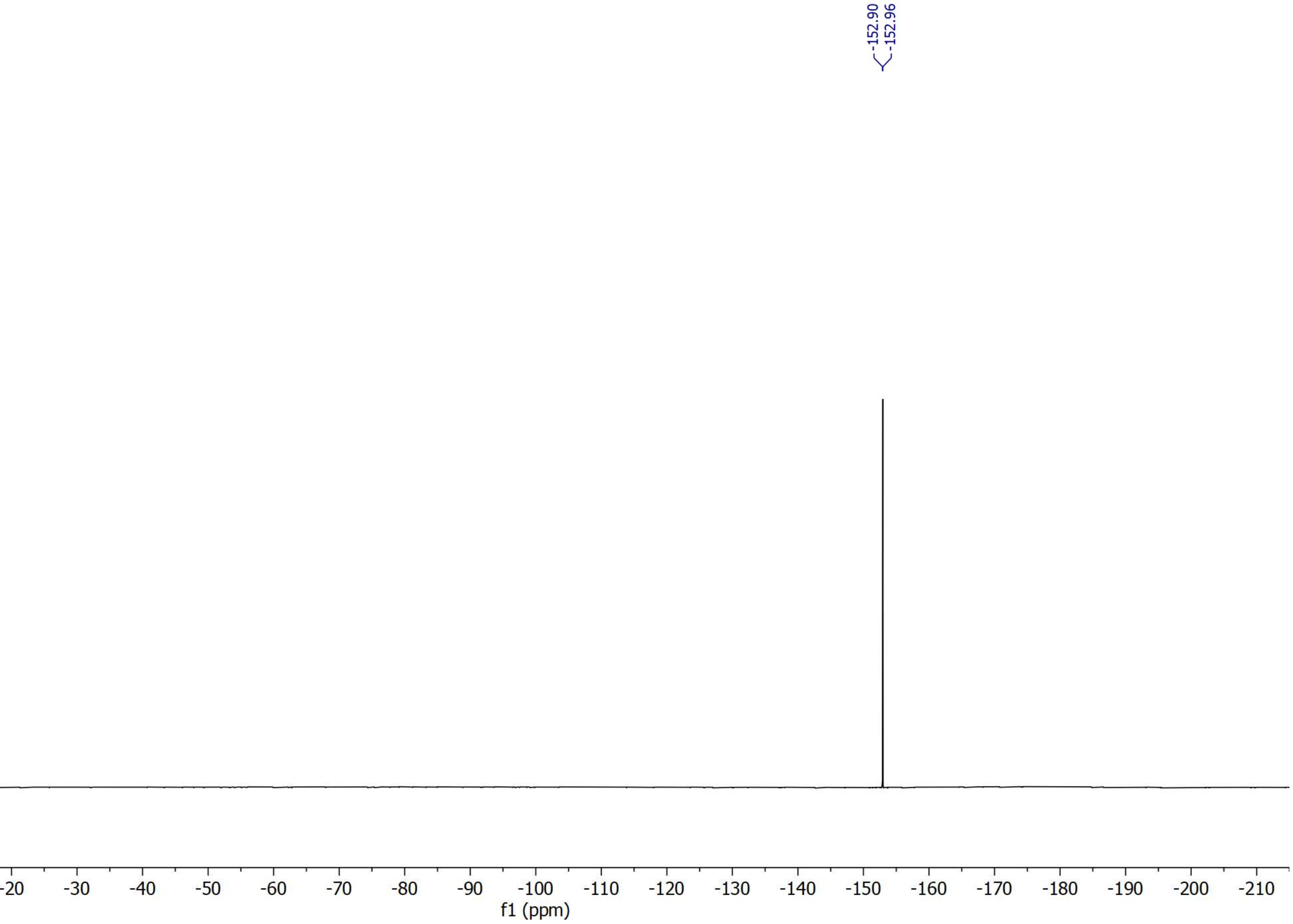

^{13}C NMR of alkenyl thianthrenium salt TT-23

$CDCl_3$, 126 MHz, 23 °C

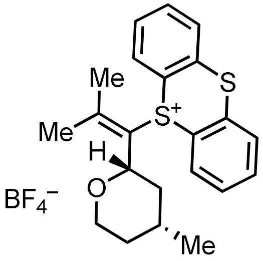

TT-23

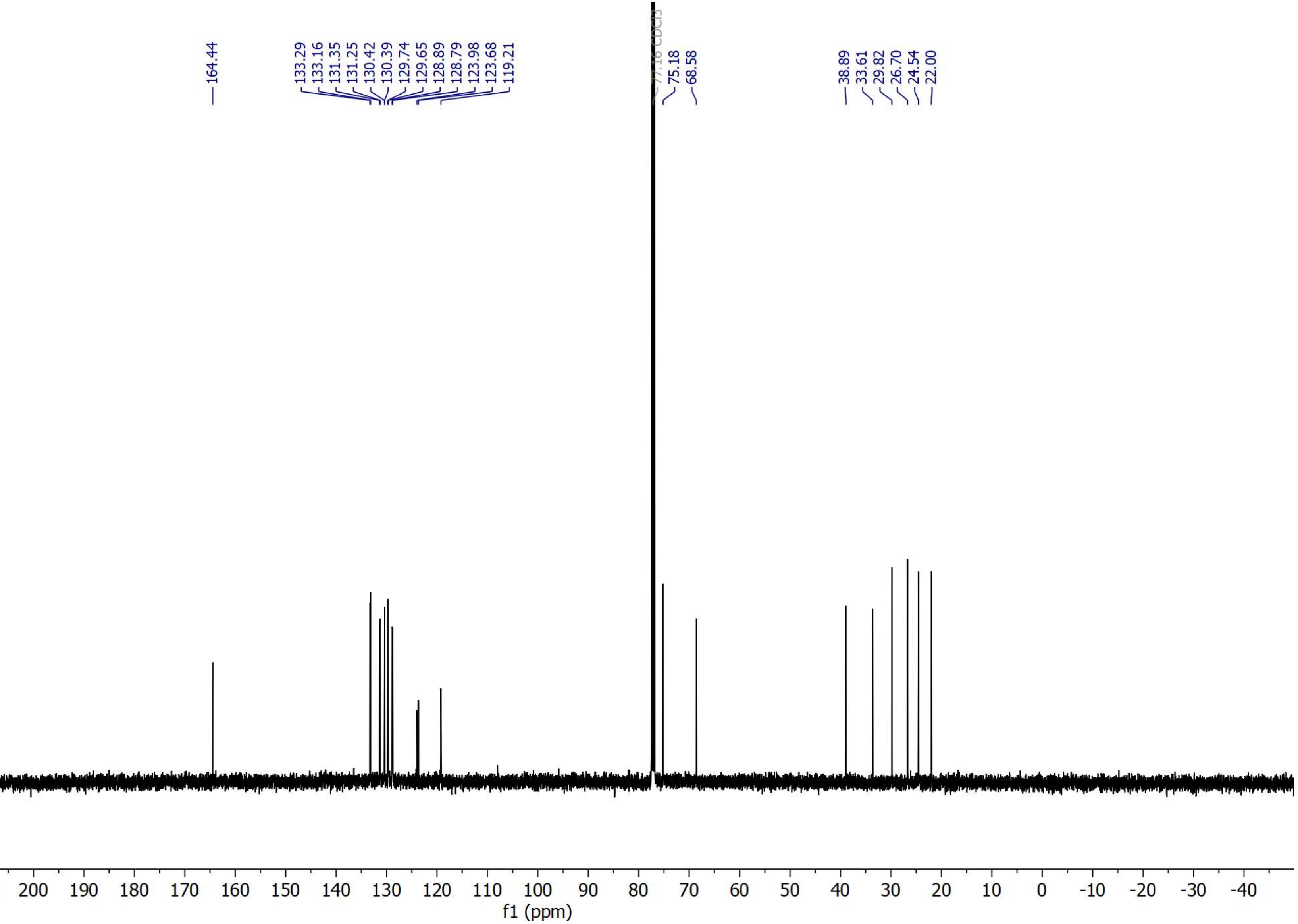

^{1}H NMR of 1,3-diene 1a

$CDCl_3$, 500 MHz, 23 °C

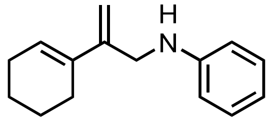

1a

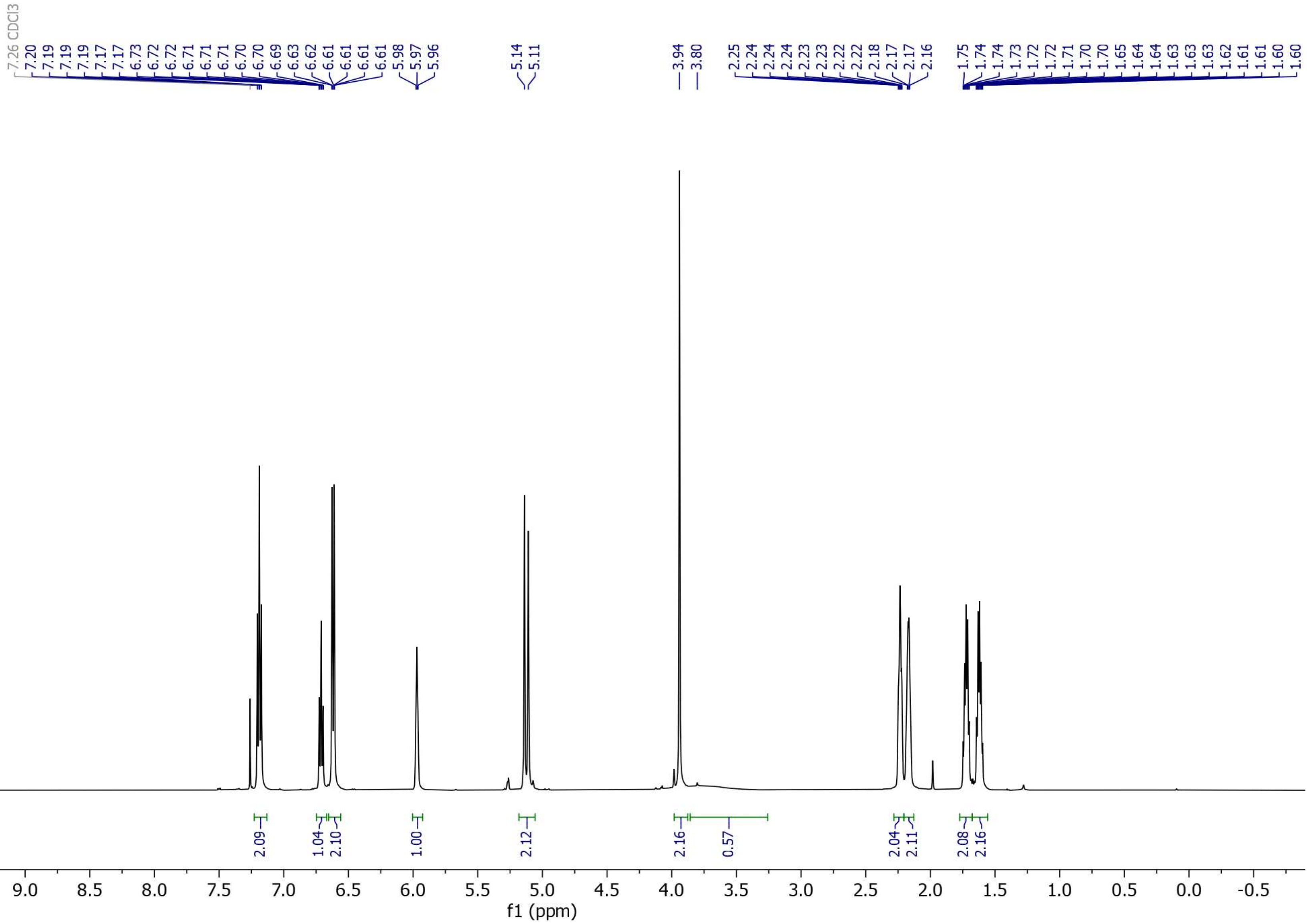

^{13}C NMR of 1,3-diene 1a

$CDCl_3$, 126 MHz, 23 °C

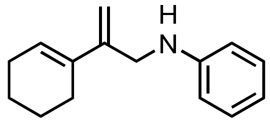

1a

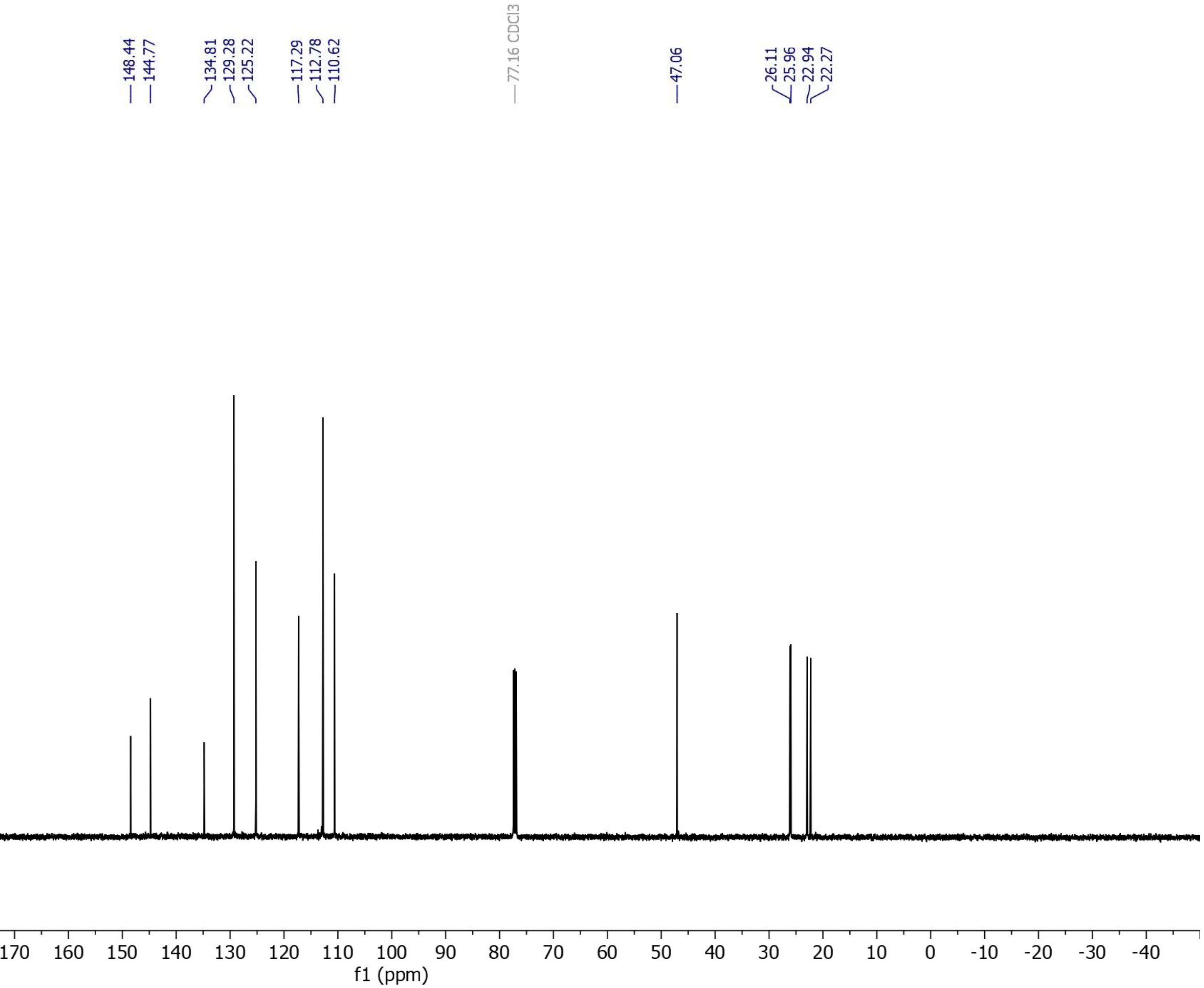

^{1}H NMR of 1,3-diene 1c

CDCl₃, 500 MHz, 23 °C

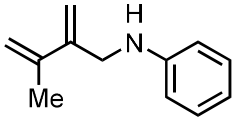

1c

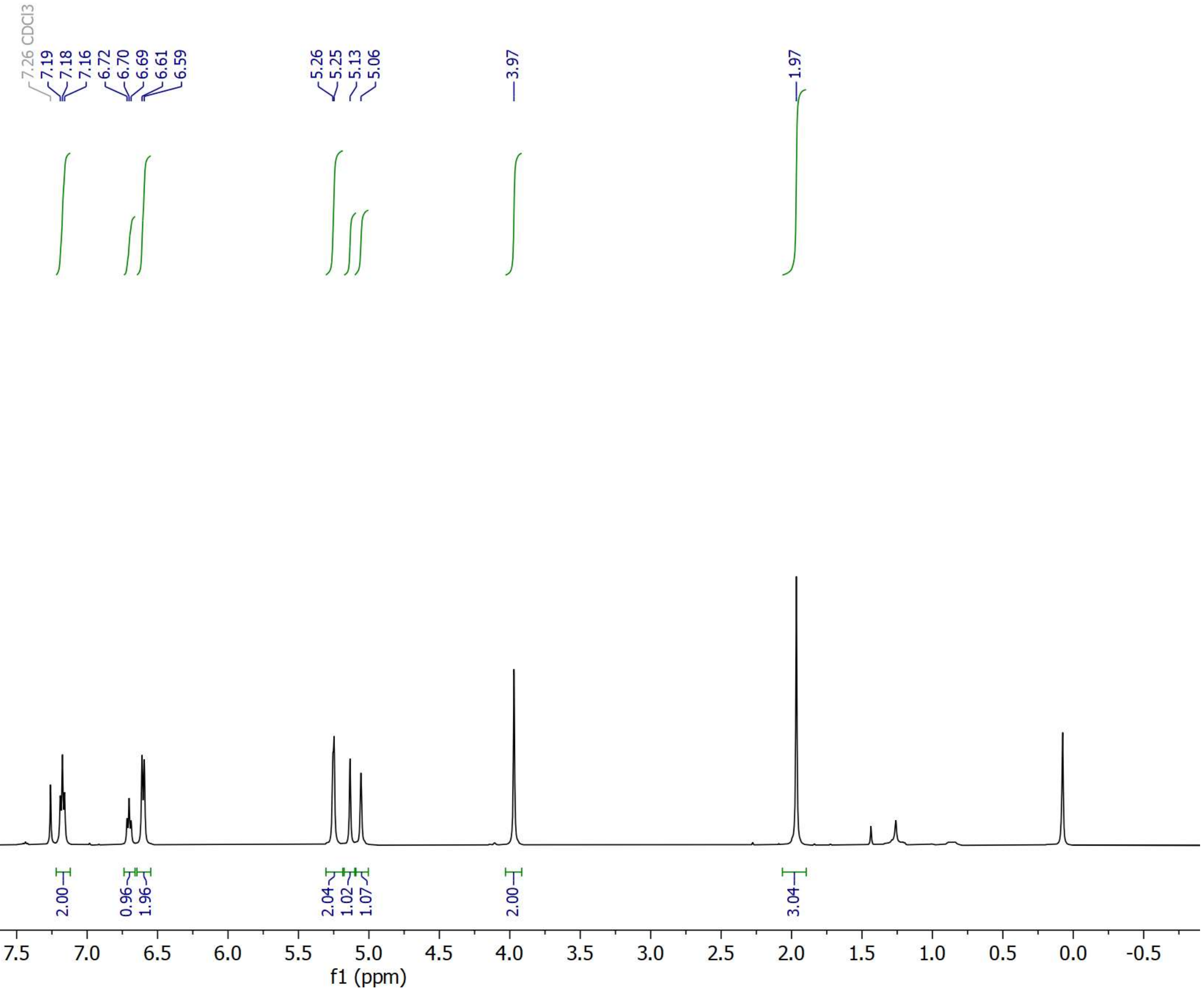

^{13}C NMR of 1,3-diene 1c

$CDCl_3$, 151 MHz, 23 °C

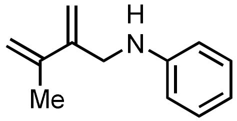

1c

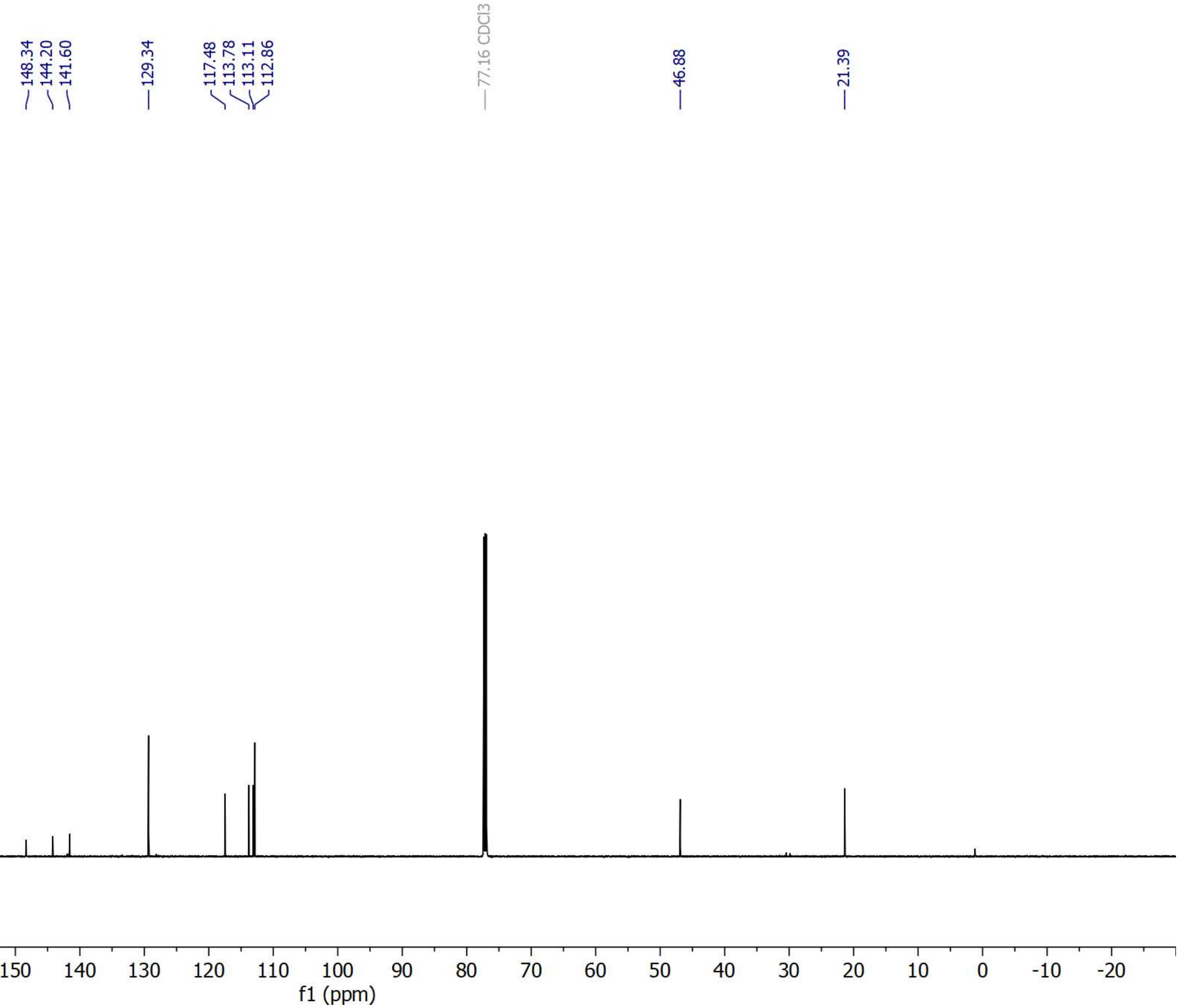

^{1}H NMR of 1,3-diene 2

CDCl$_3$, 500 MHz, 23 °C

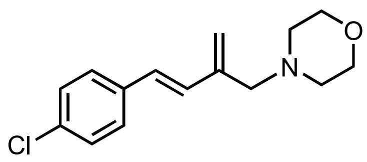

2

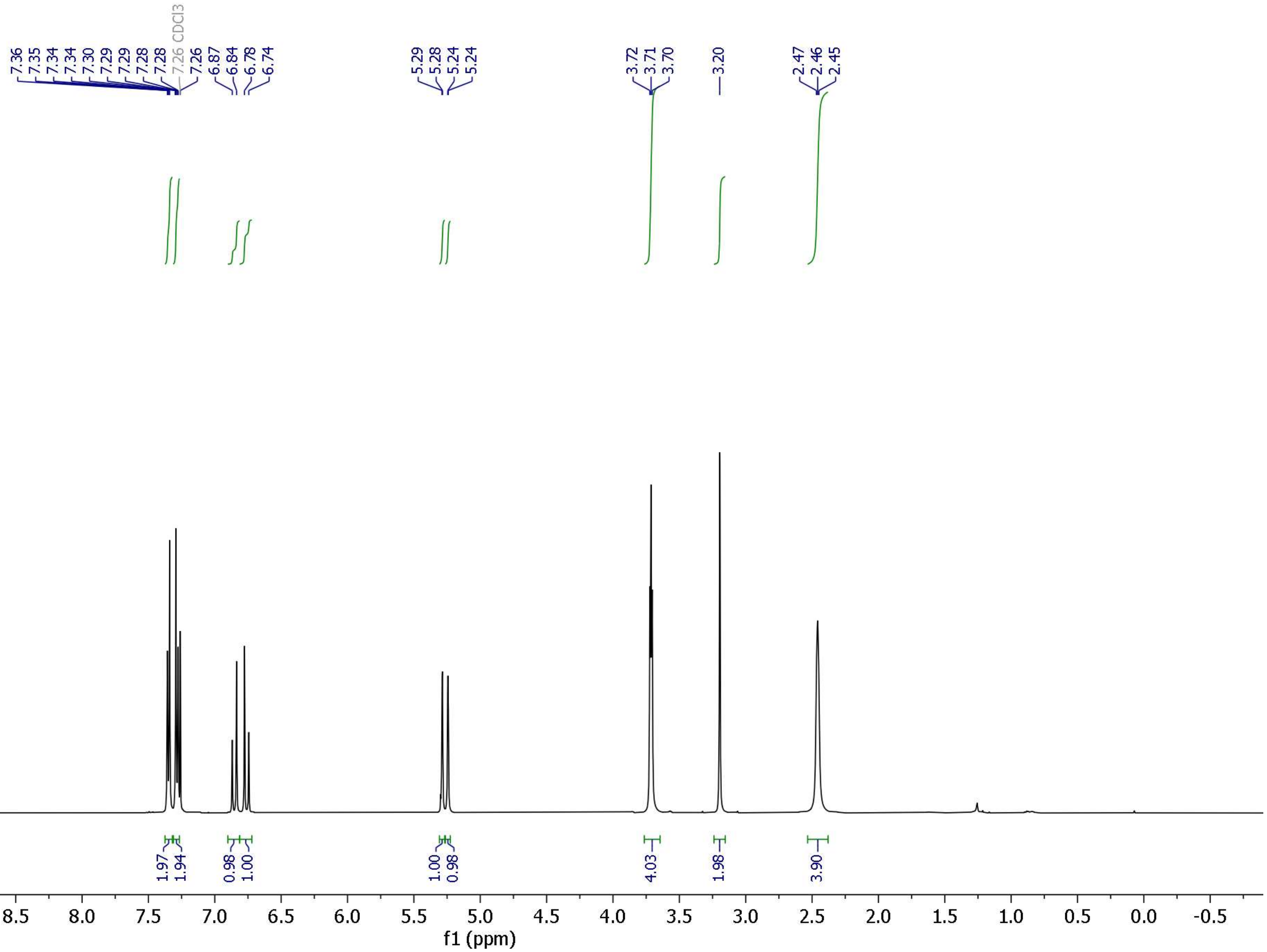

^{13}C NMR of 1,3-diene 2

CDCl$_3$, 126 MHz, 23 °C

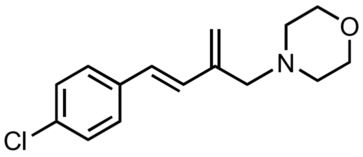

2

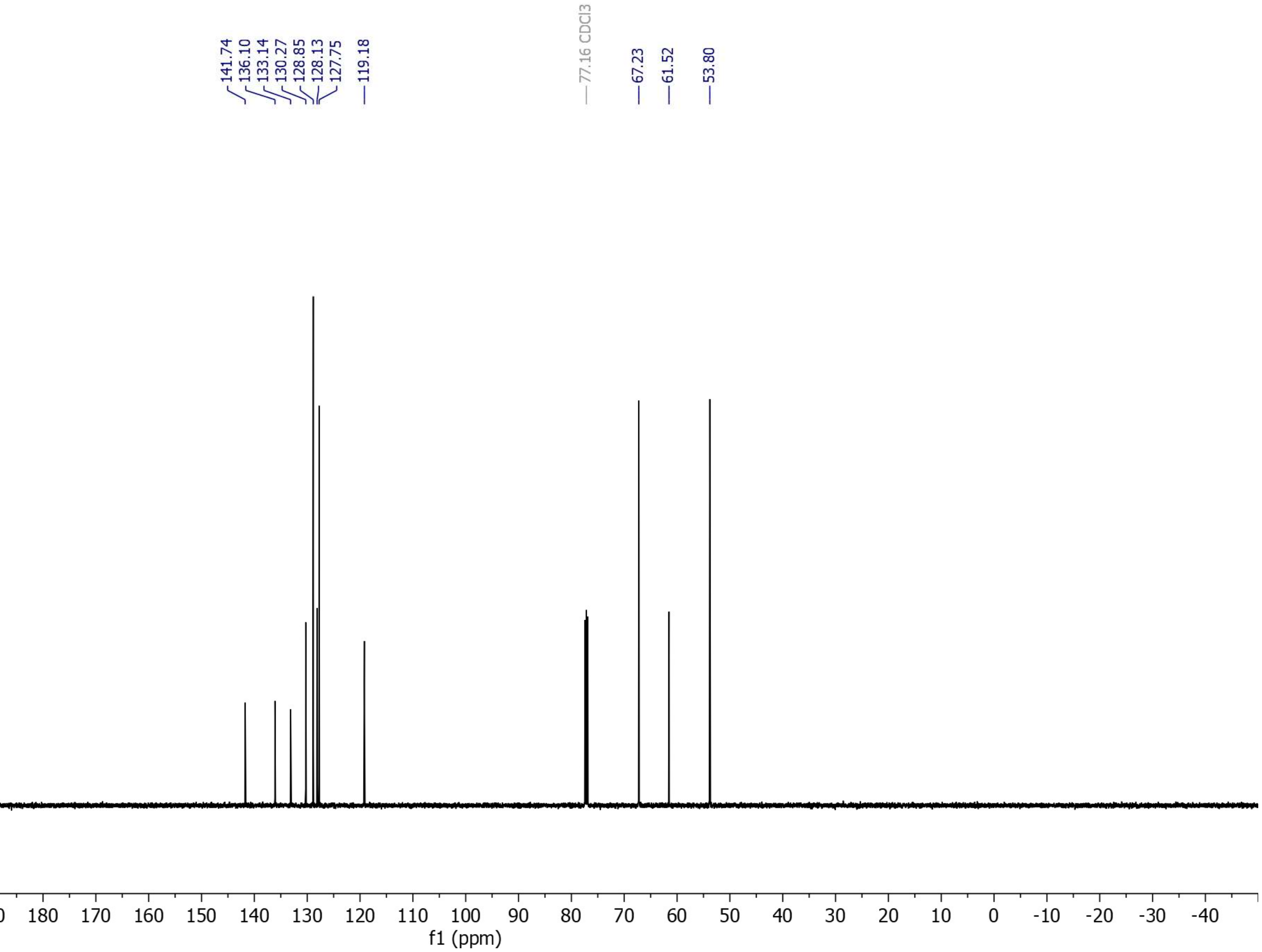

^{1}H NMR of 1,3-diene 3

$CDCl_3$, 500 MHz, 23 °C

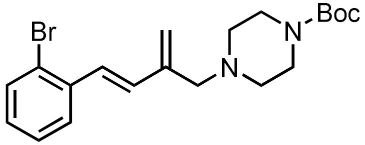

3

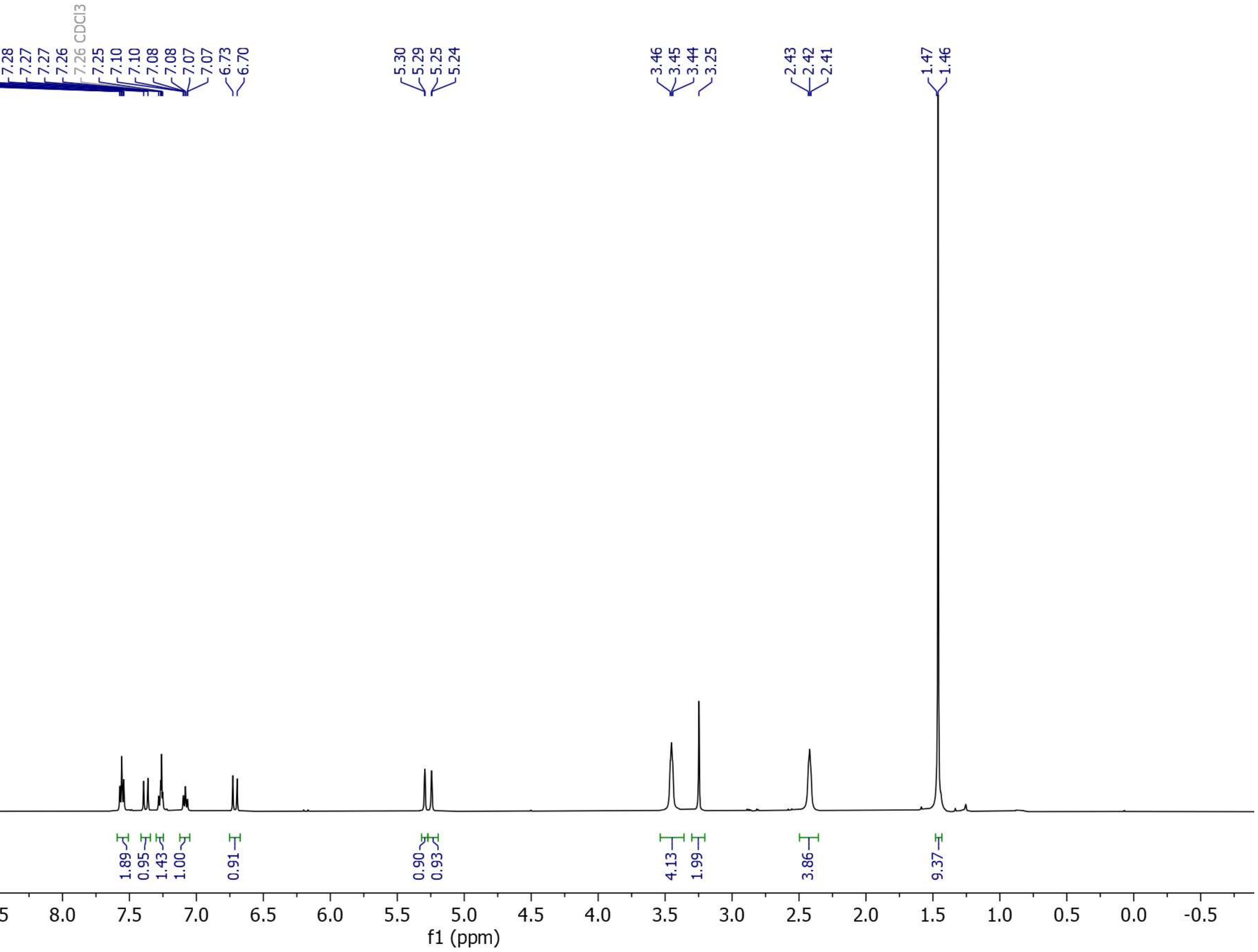

^{13}C NMR of 1,3-diene 3

CDCl$_3$, 126 MHz, 23 °C

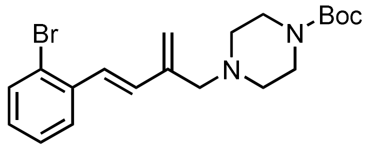

3

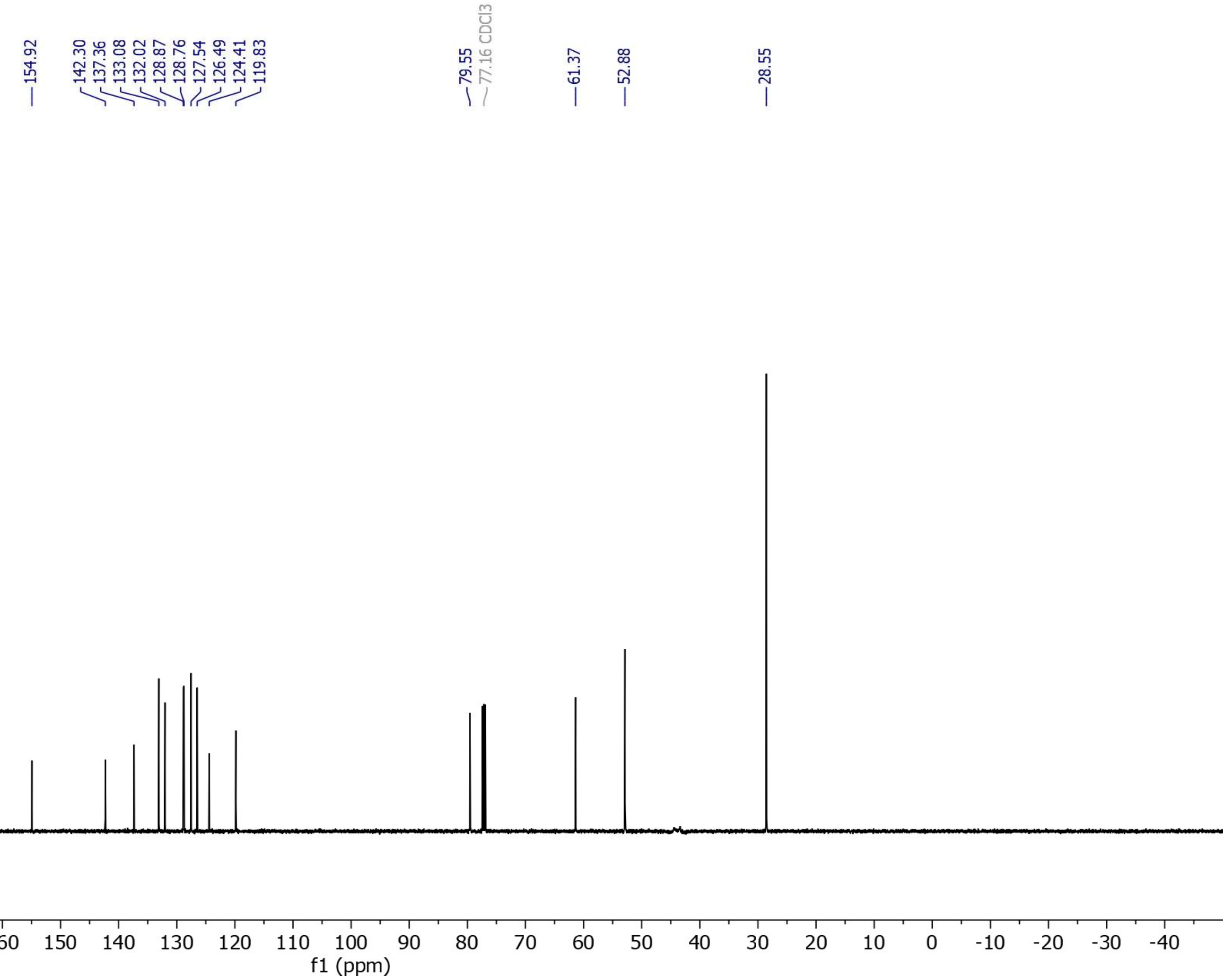

^{1}H NMR of 1,3-diene 4

CDCl$_3$, 500 MHz, 23 °C

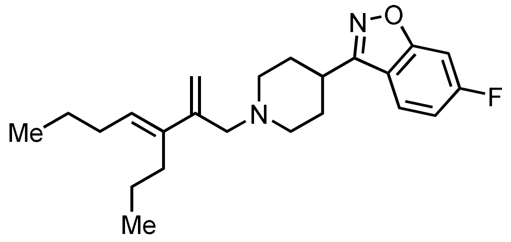

4

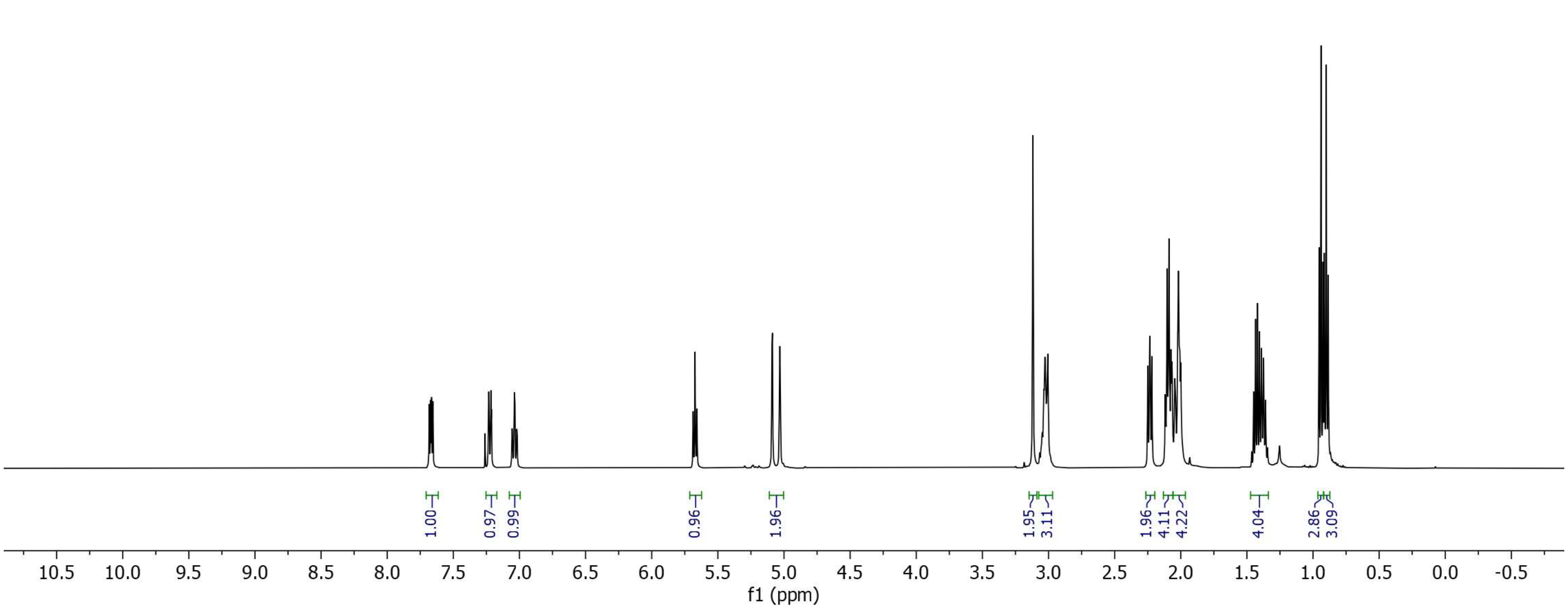

^{13}C NMR of 1,3-diene 4

$CDCl_3$, 126 MHz, 23 °C

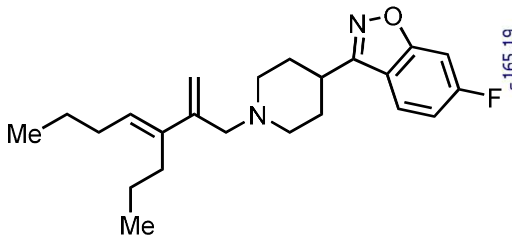

4

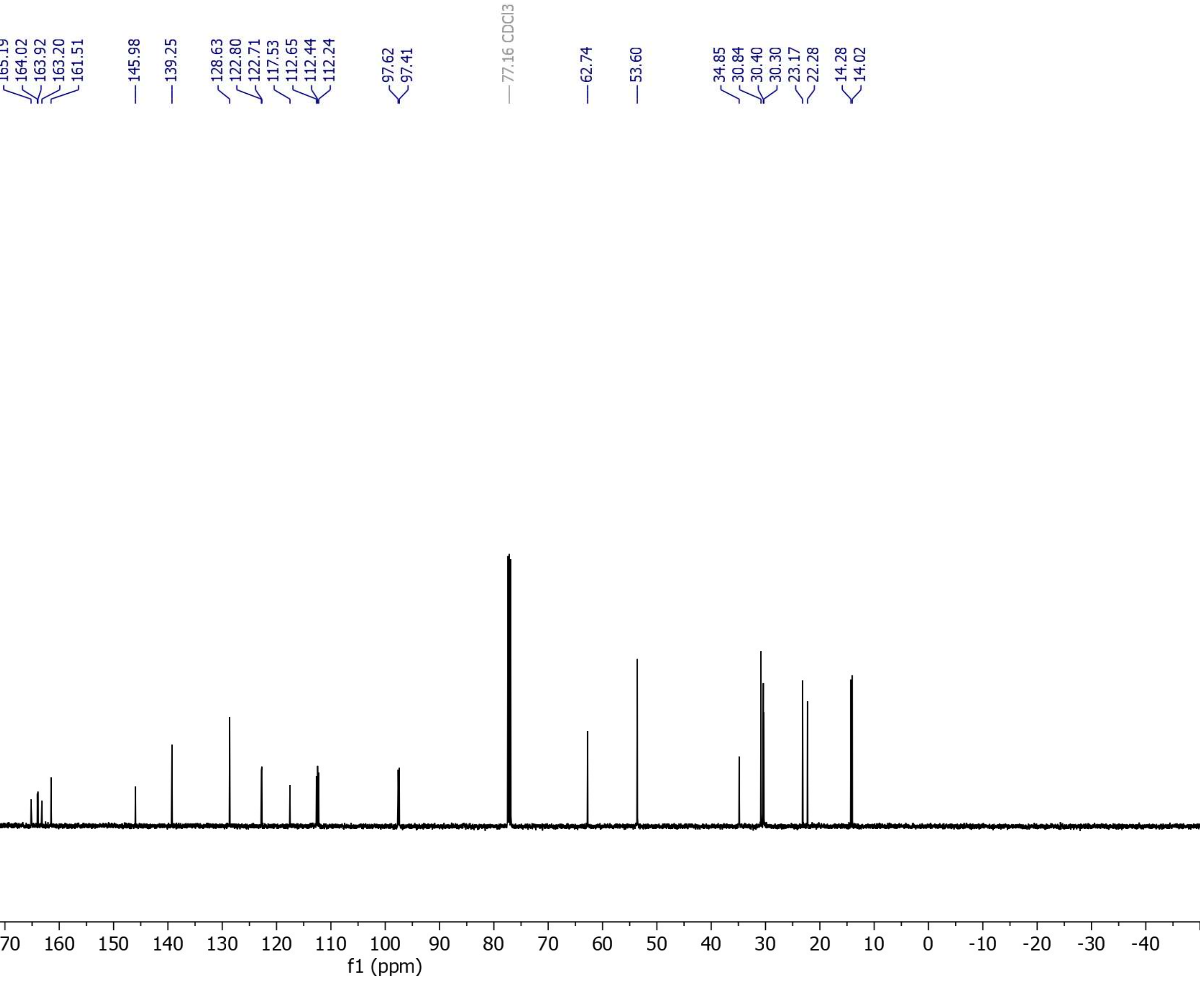

^{19}F NMR of 1,3-diene 4

$CDCl_3$, 476 MHz, 23 °C

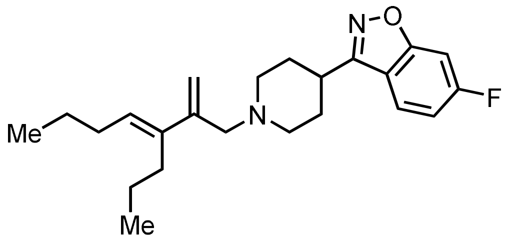

4

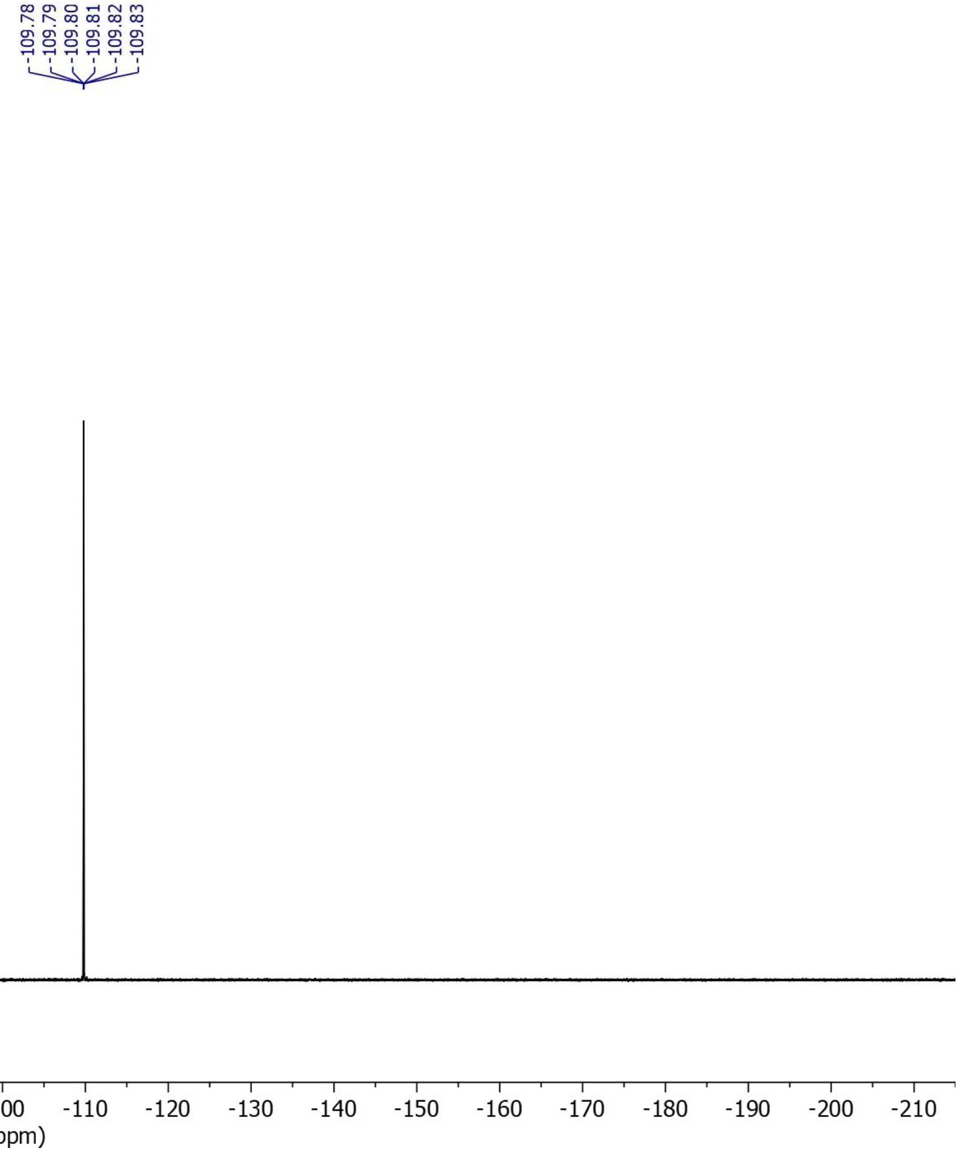

^{1}H NMR of 1,3-diene (±)-5

$CDCl_3$, 500 MHz, 23 °C

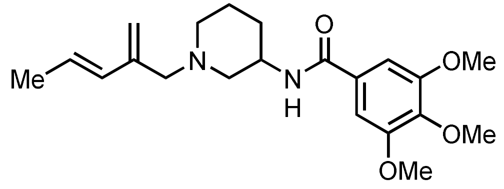

(±)-**5**

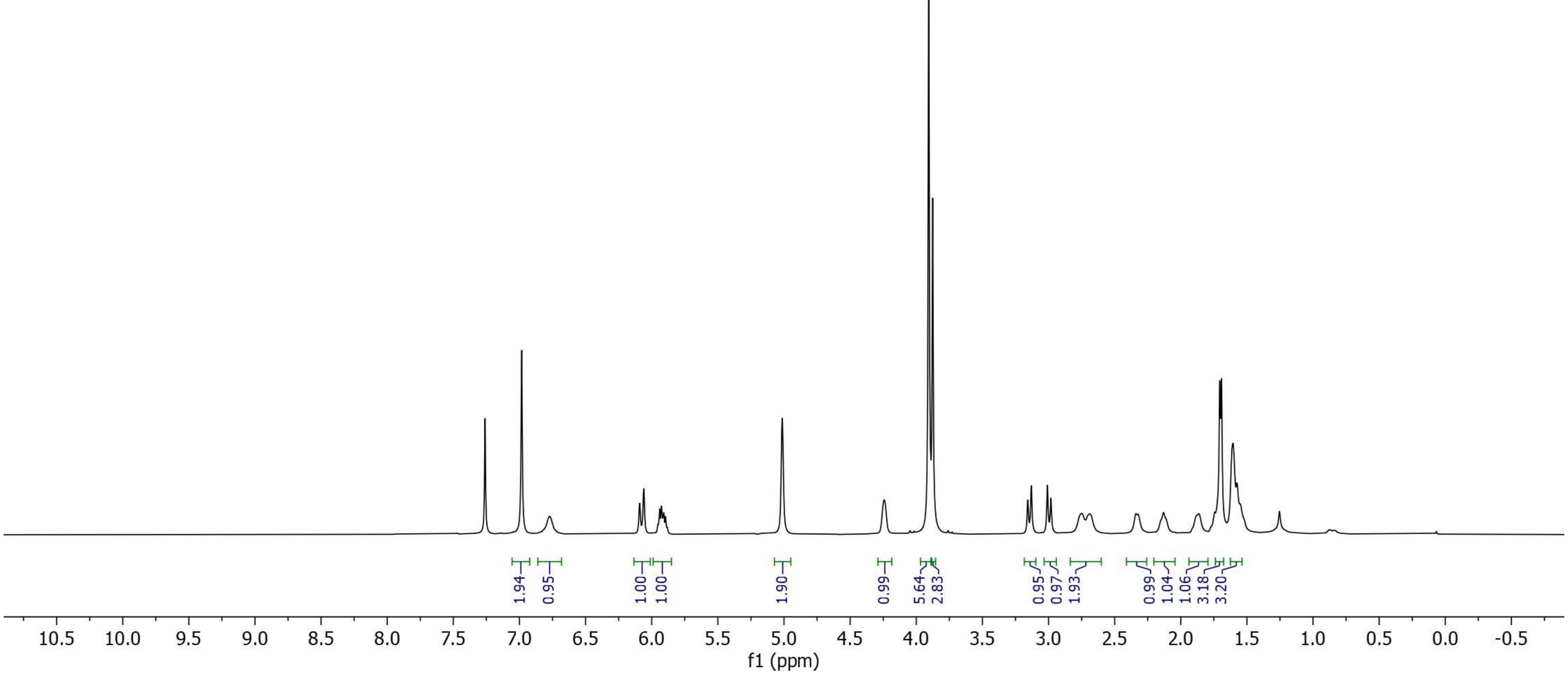

^{13}C NMR of 1,3-diene (±)-5

CDCl$_3$, 126 MHz, 23 °C

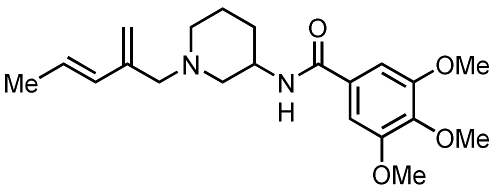

(±)-**5**

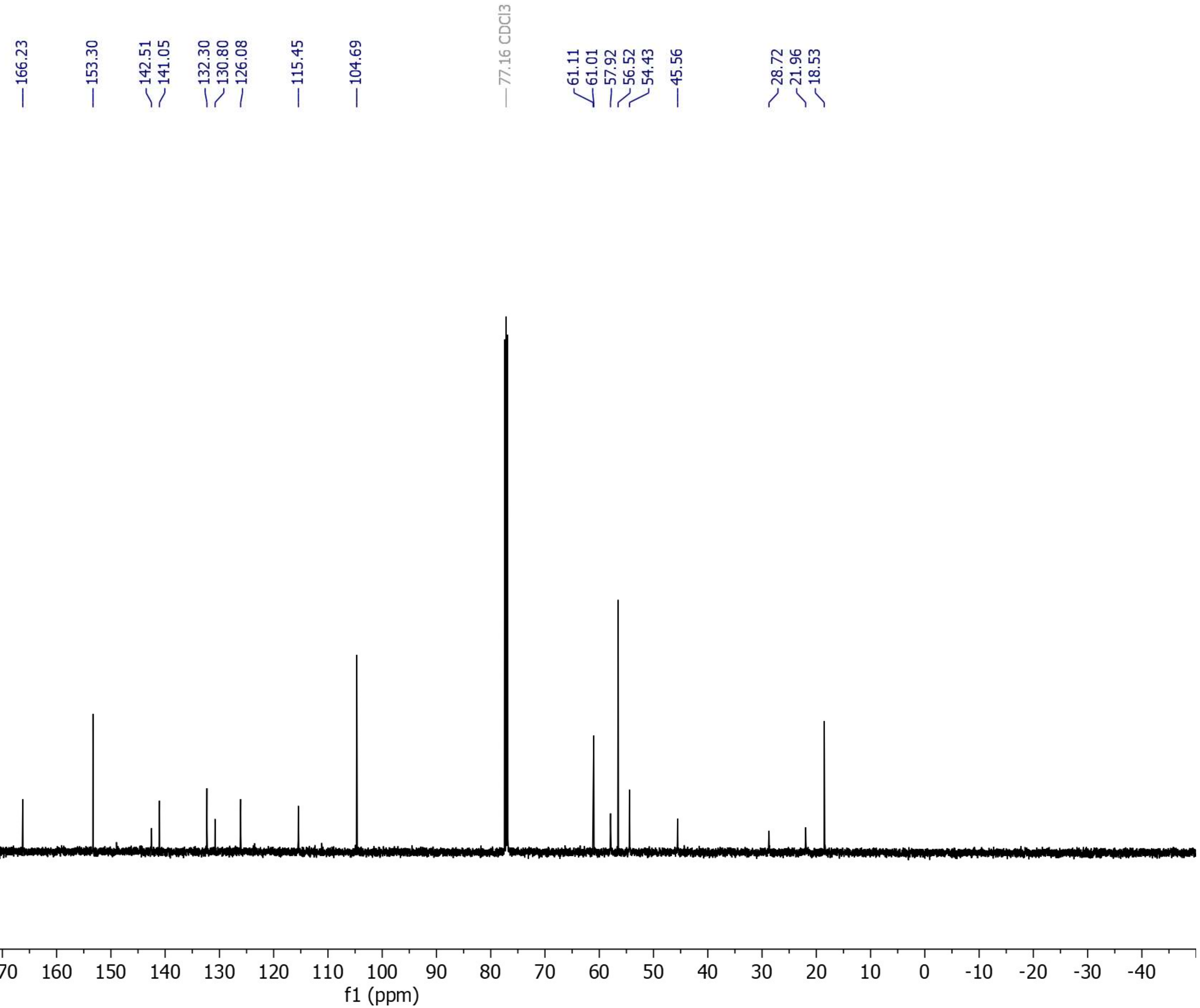

^{1}H NMR of 1,3-diene 6

$CDCl_3$, 500 MHz, 23 °C

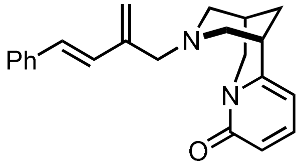

6

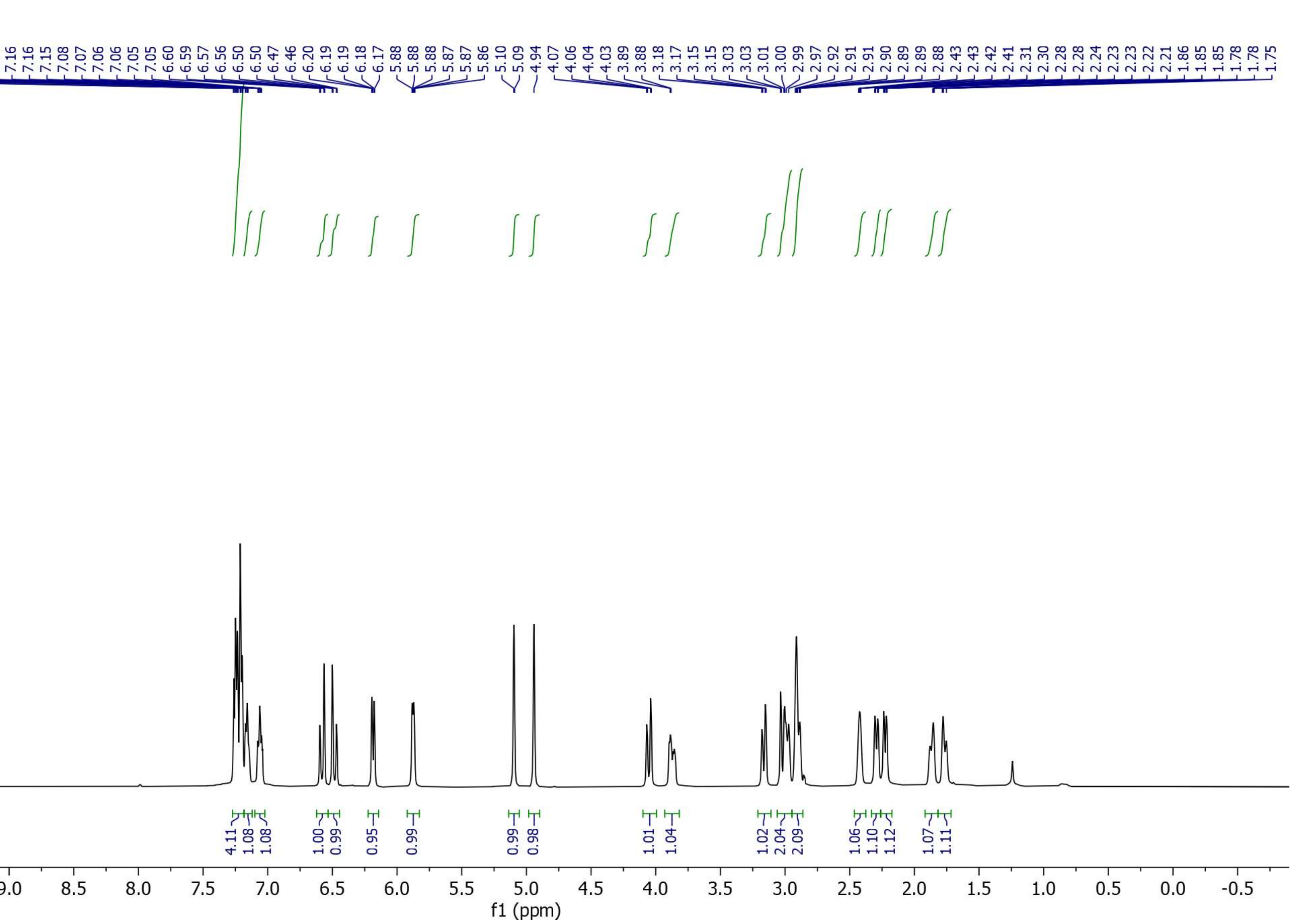

^{13}C NMR of 1,3-diene 6

CDCl$_3$, 126 MHz, 23 °C

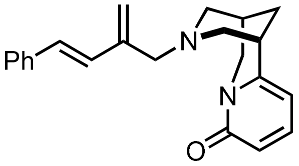

6

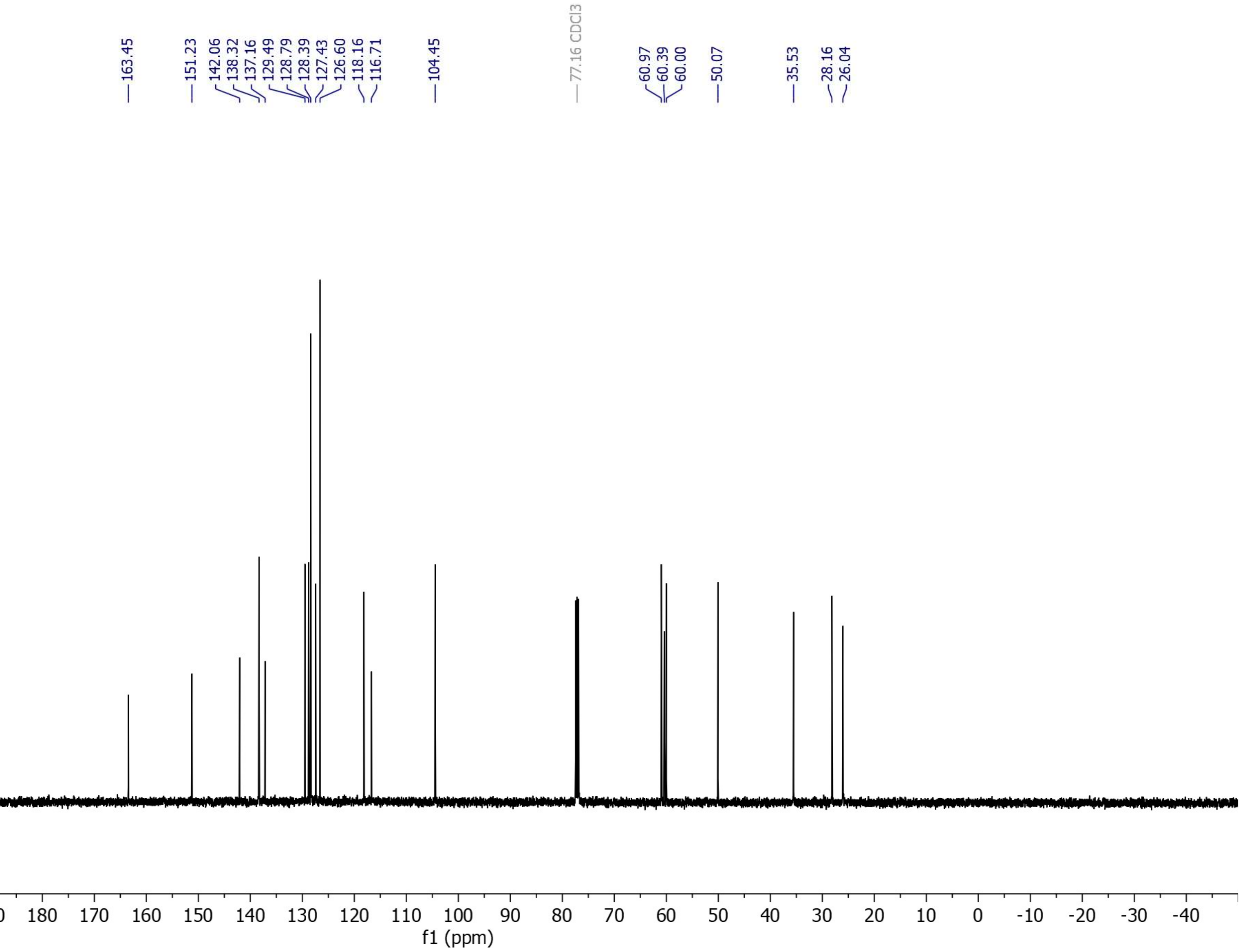

^{1}H NMR of 1,3-diene 7

$CDCl_3$, 500 MHz, 23 °C

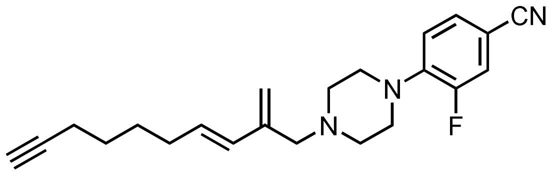

7

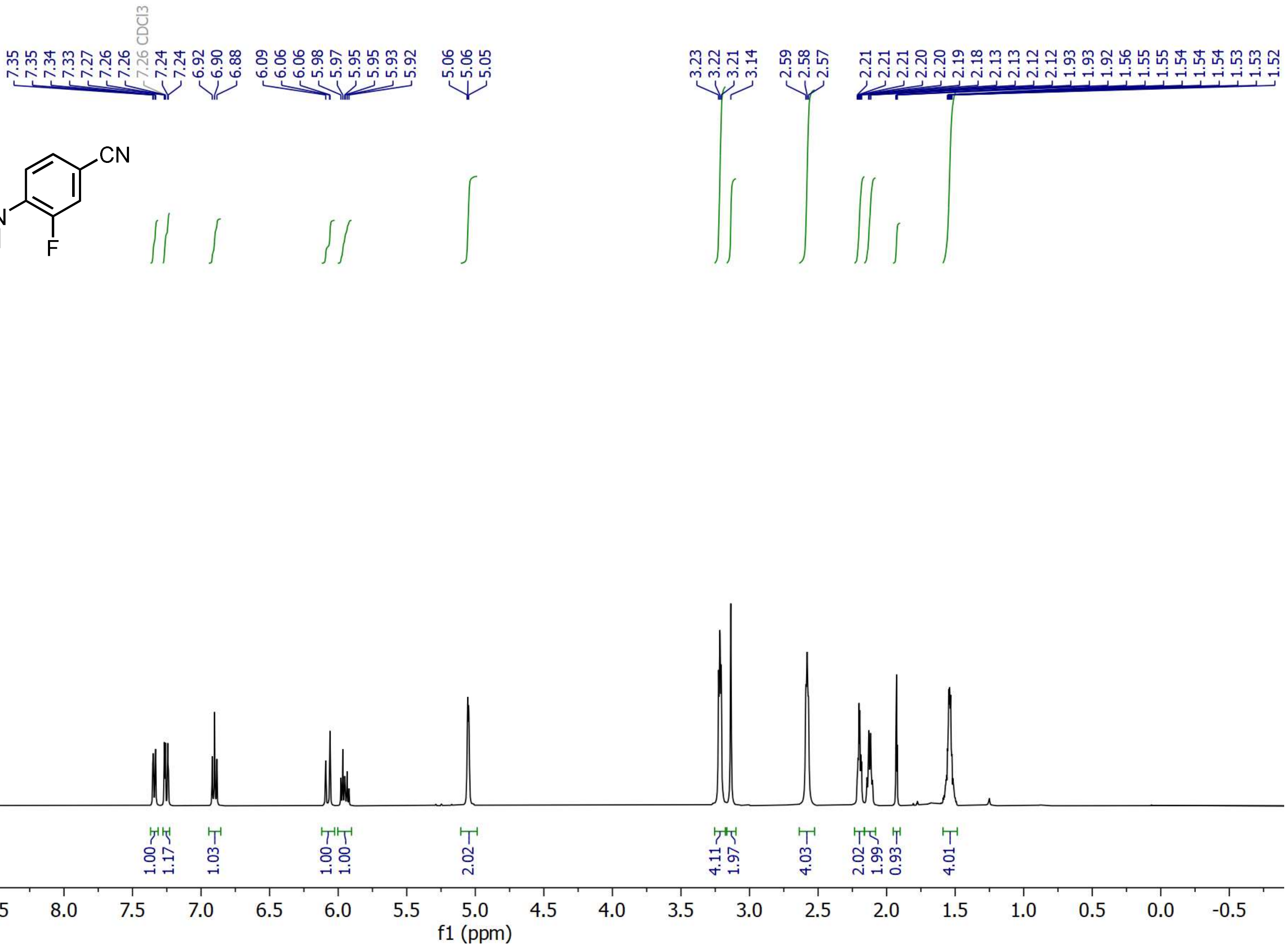

^{13}C NMR of 1,3-diene 7

$CDCl_3$, 126 MHz, 23 °C

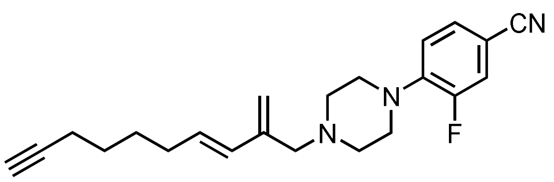

7

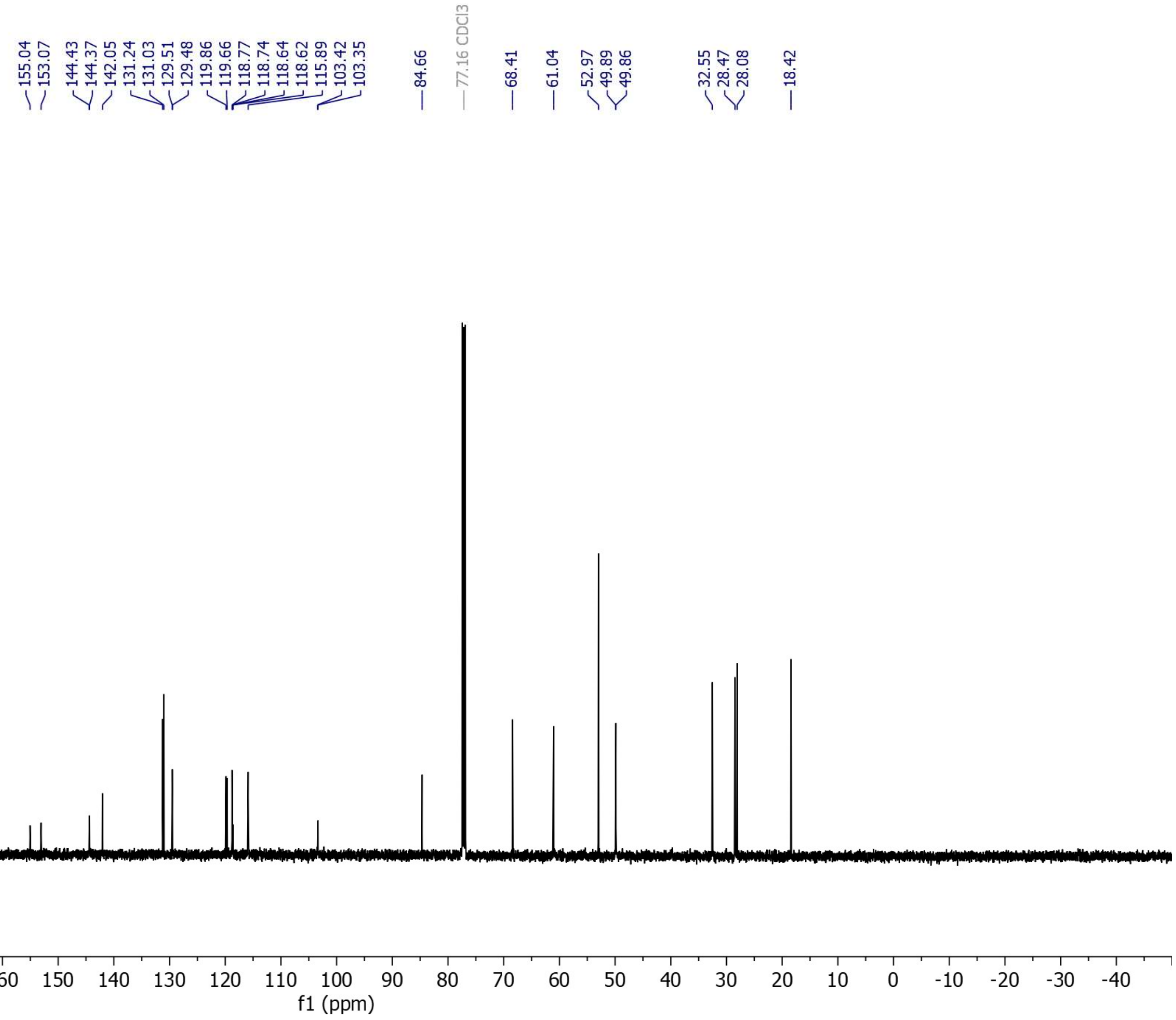

^{19}F NMR of 1,3-diene 7

CDCl$_3$, 476 MHz, 23 °C

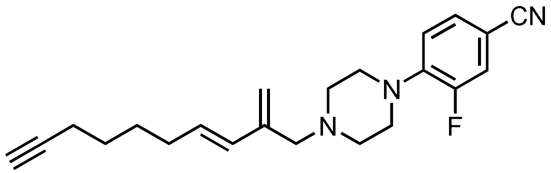

7

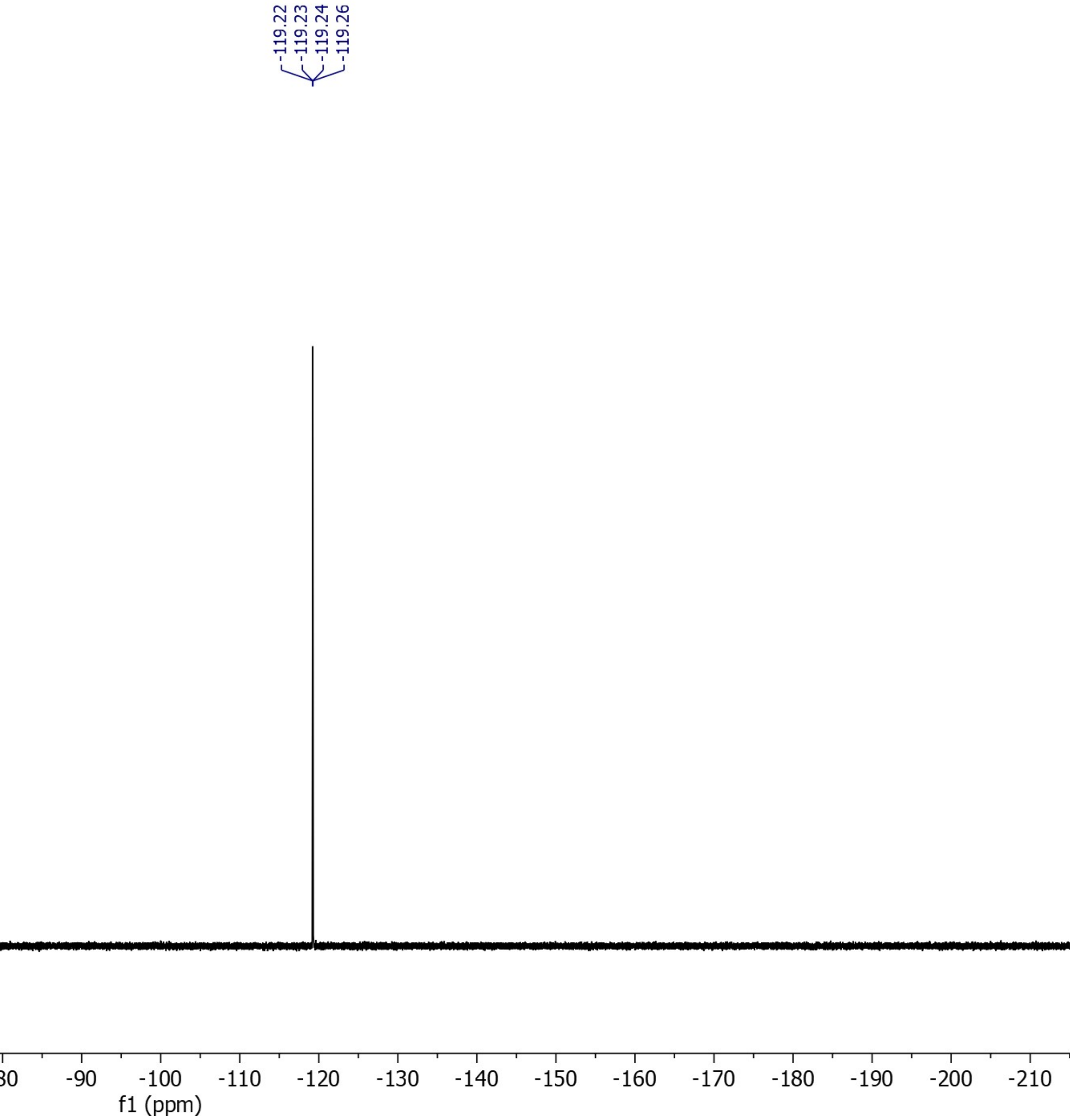

^{1}H NMR of 1,3-diene 8

$CDCl_3$, 500 MHz, 23 °C

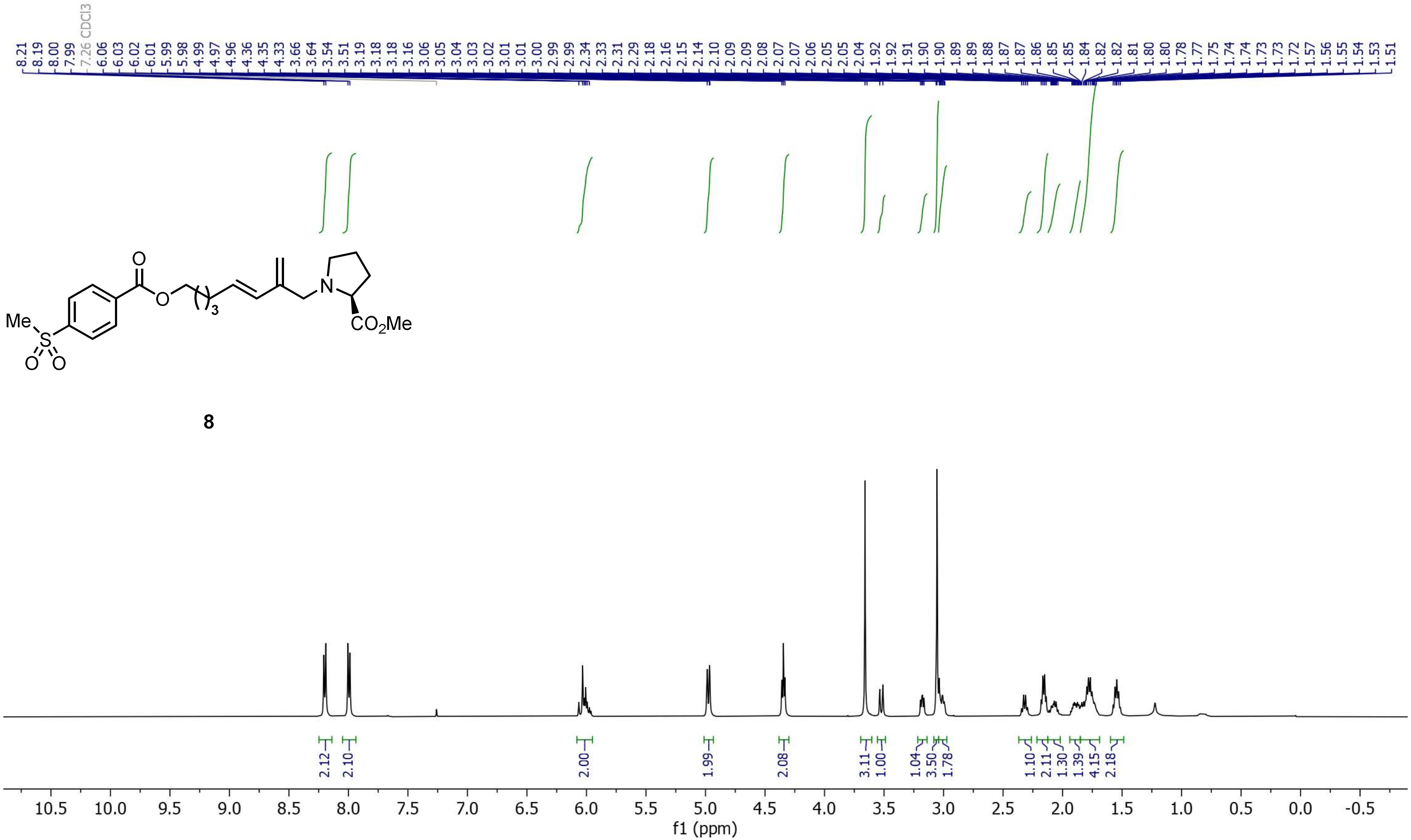

^{13}C NMR of 1,3-diene 8

CDCl$_3$, 126 MHz, 23 °C

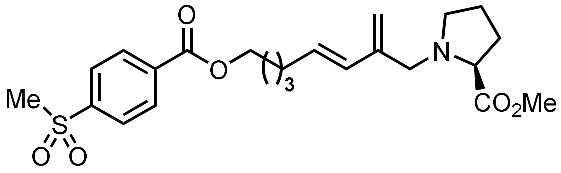

8

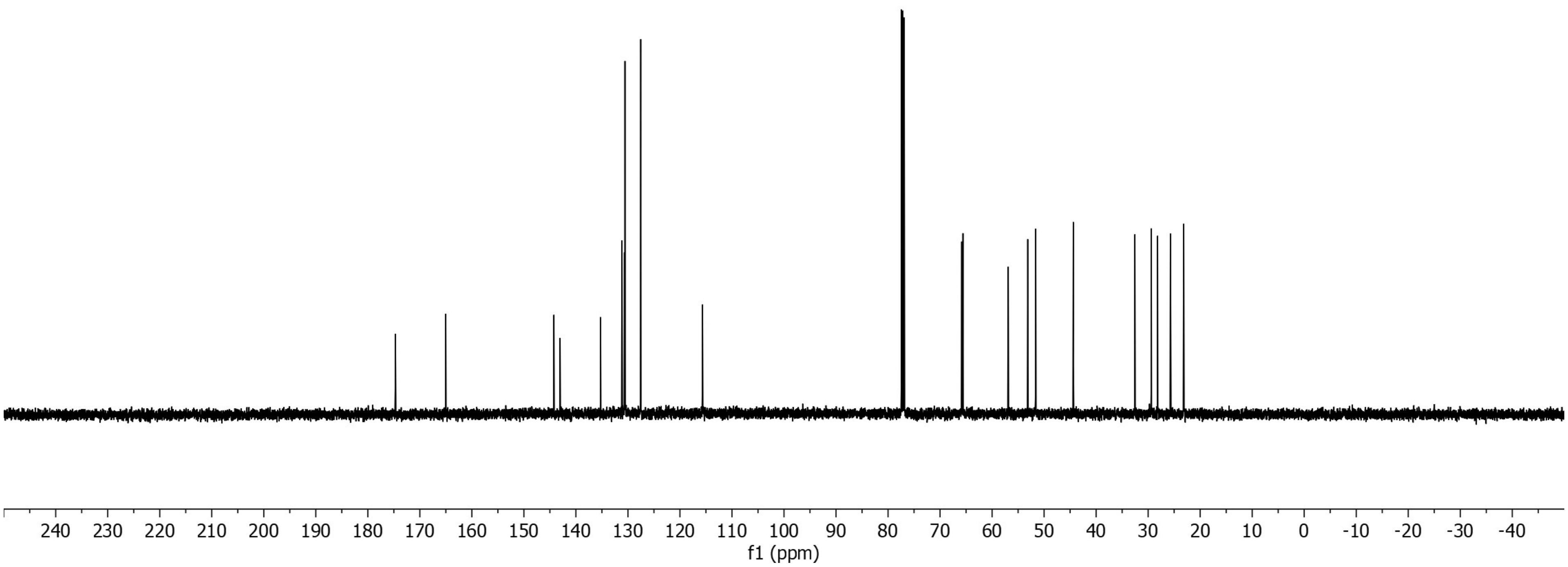

^{1}H NMR of 1,3-diene 9

CDCl$_3$, 600 MHz, 23 °C

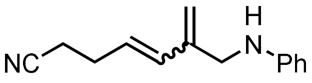

9

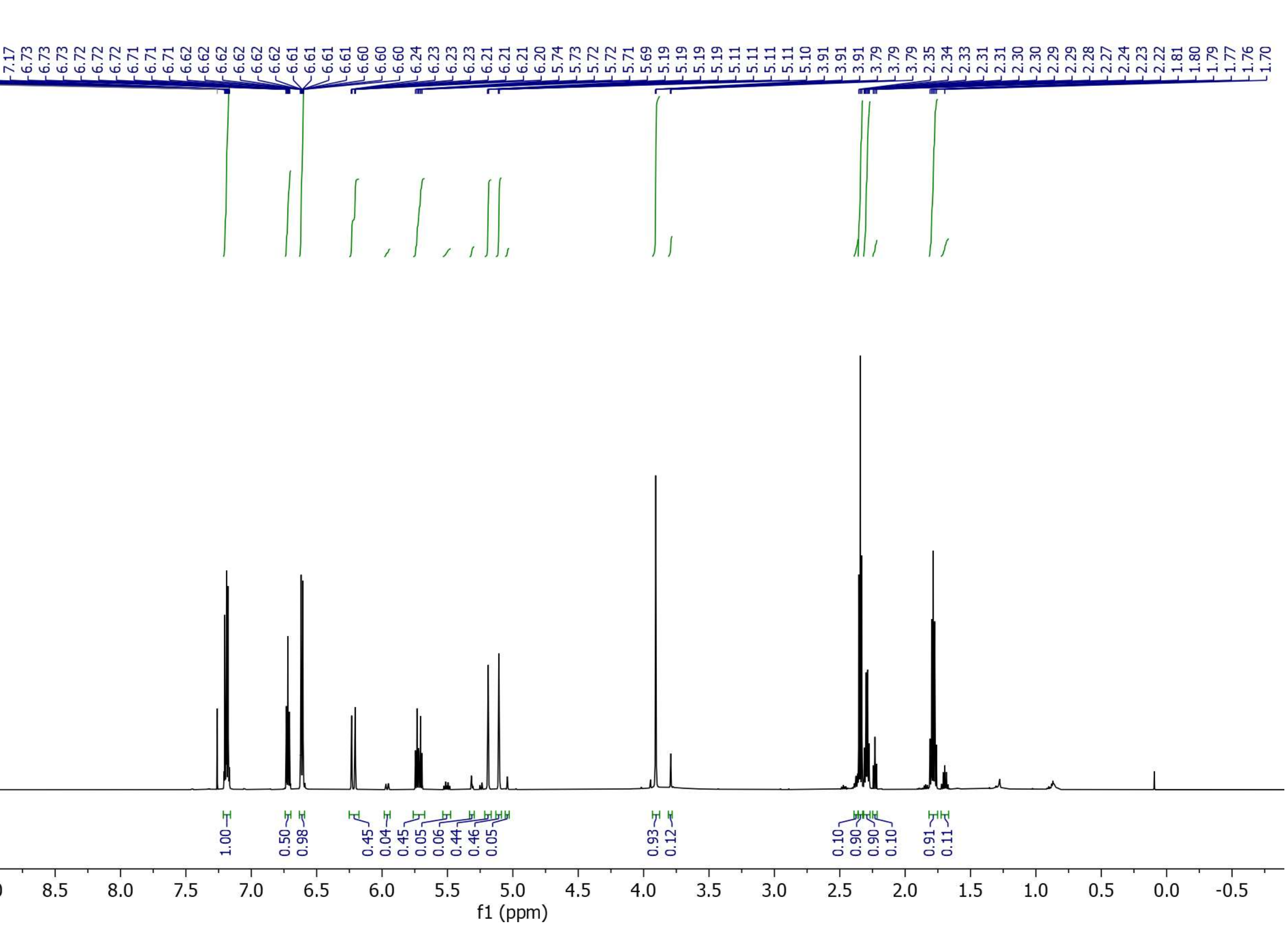

^{13}C NMR of 1,3-diene 9

$CDCl_3$, 151 MHz, 23 °C

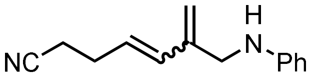

9

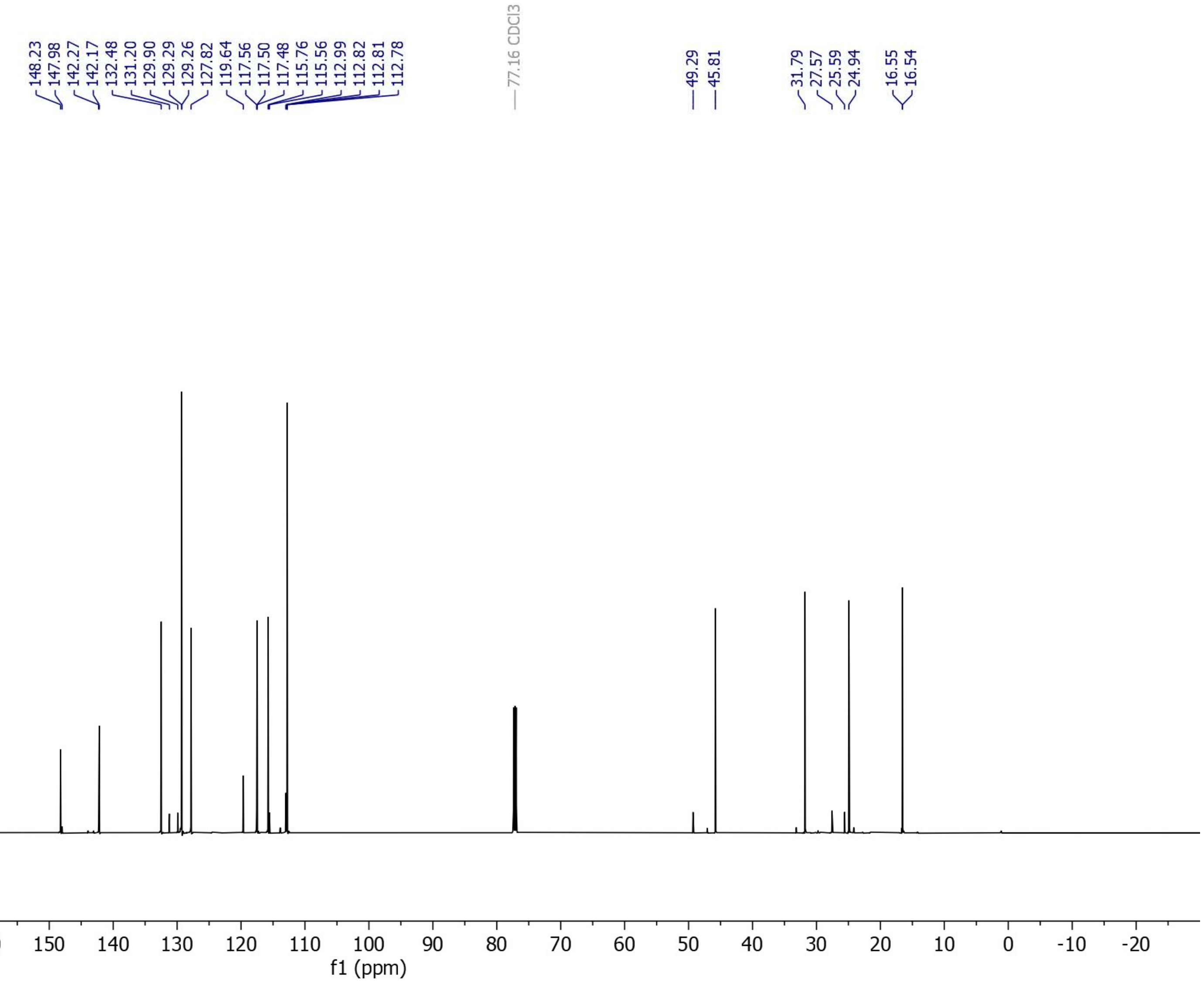

^{1}H NMR of 1,3-diene 10

CDCl$_3$, 500 MHz, 23 °C

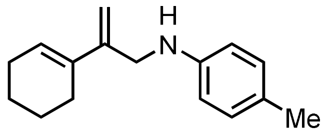

10

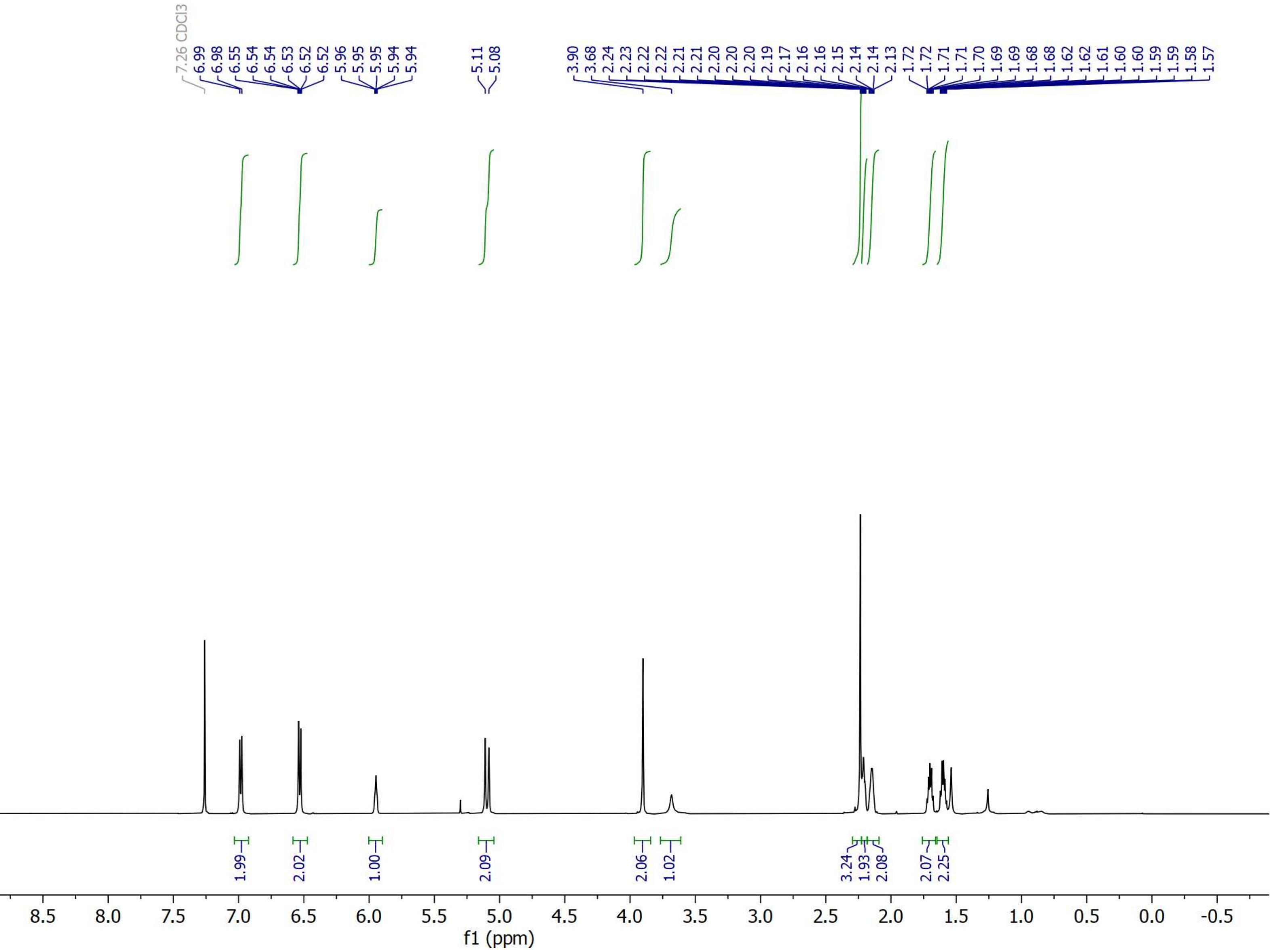

^{13}C NMR of 1,3-diene 10

$CDCl_3$, 126 MHz, 23 °C

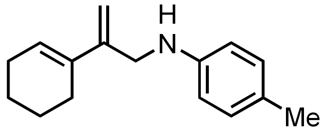

10

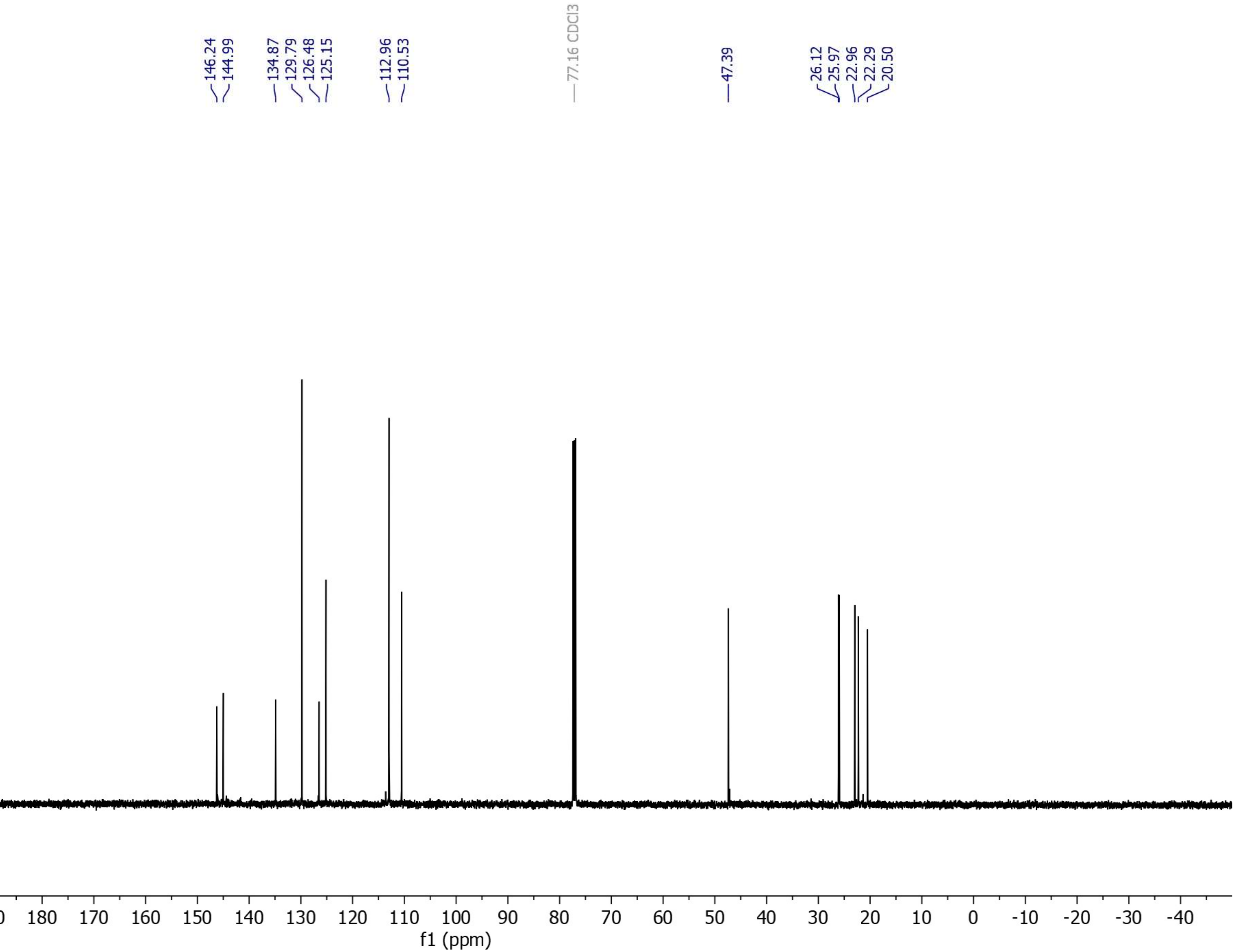

^{1}H NMR of 1,3-diene 11a

$CDCl_3$, 500 MHz, 23 °C

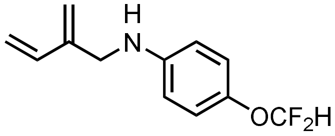

11a

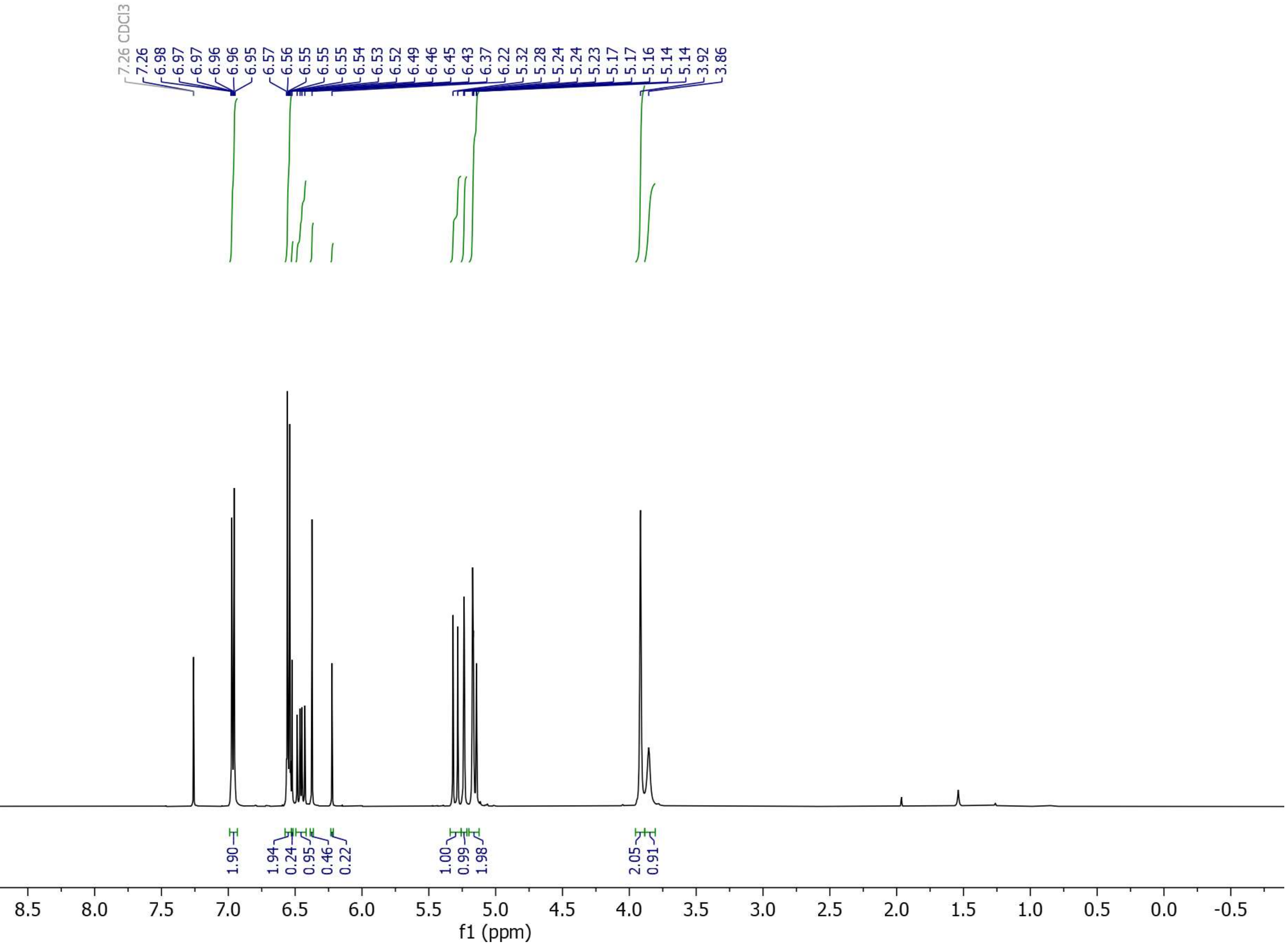

^{13}C NMR of 1,3-diene 11a

$CDCl_3$, 126 MHz, 23 °C

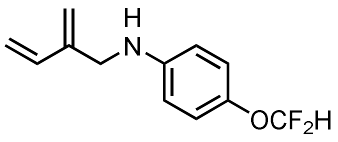

11a

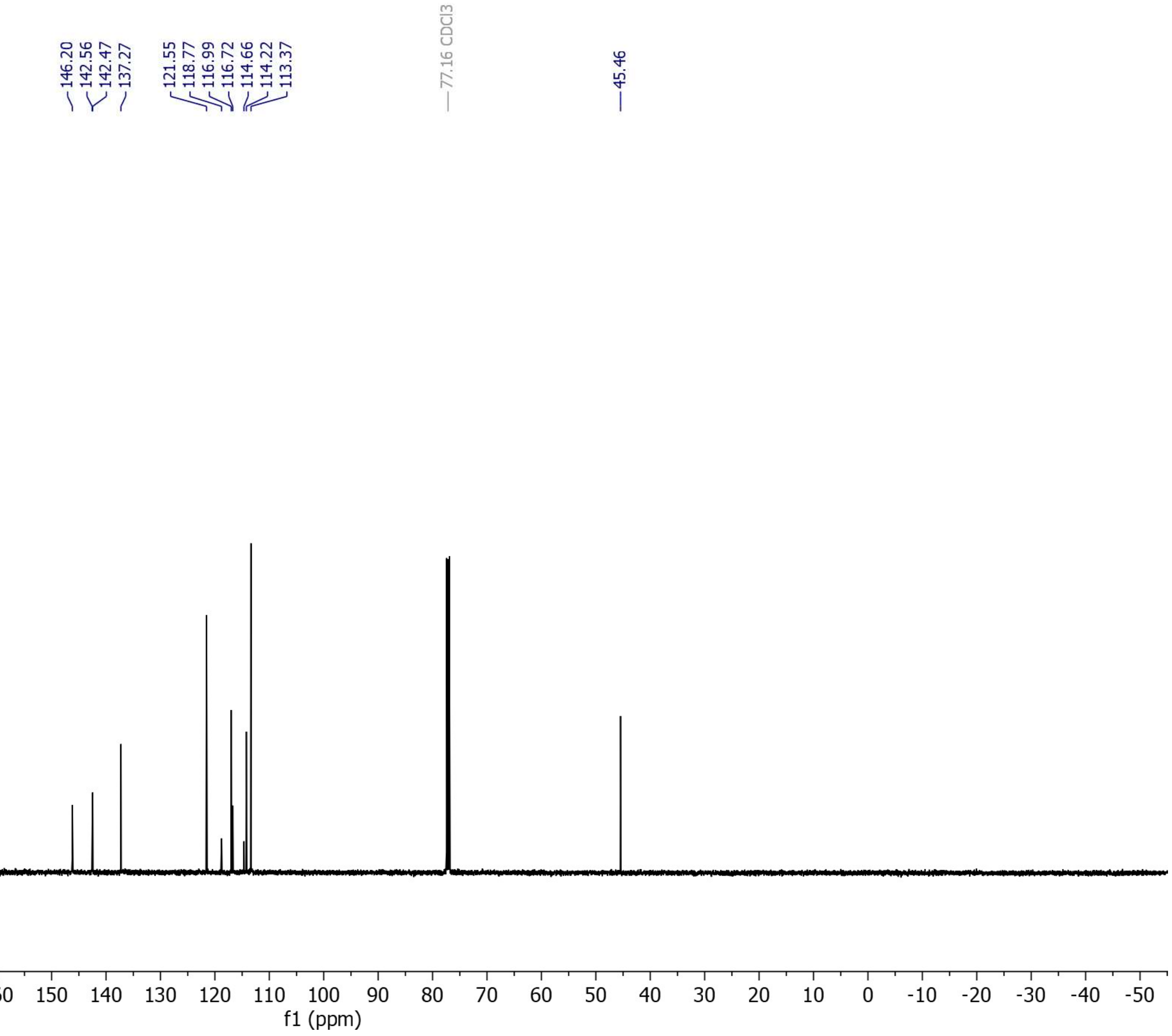

^{19}F NMR of 1,3-diene 11a

$CDCl_3$, 476 MHz, 23 °C

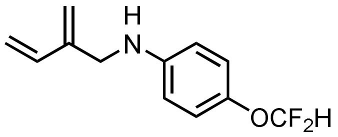

11a

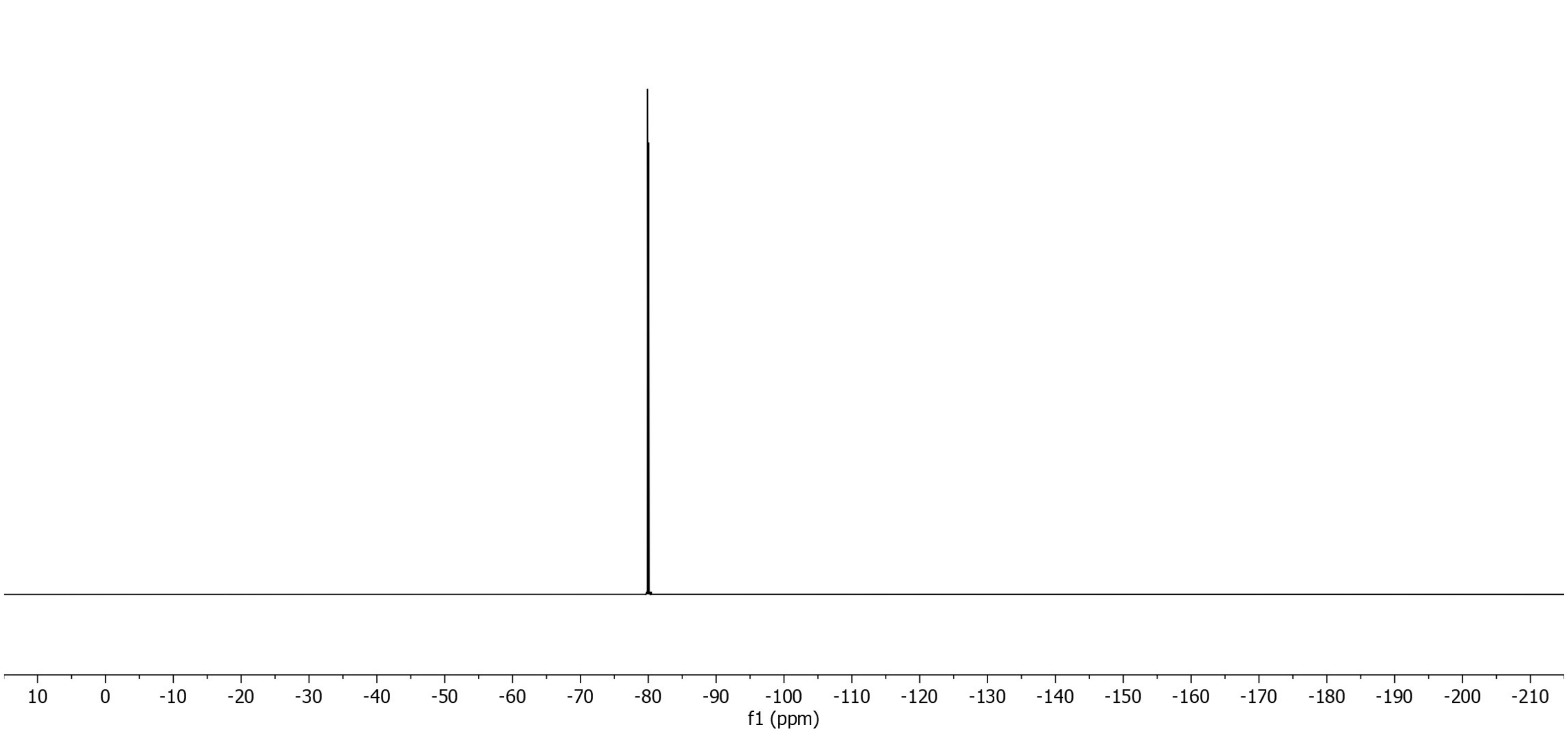

^{1}H NMR of 1,3-diene 11b

CDCl$_3$, 500 MHz, 23 °C

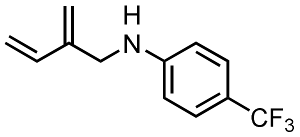

11b

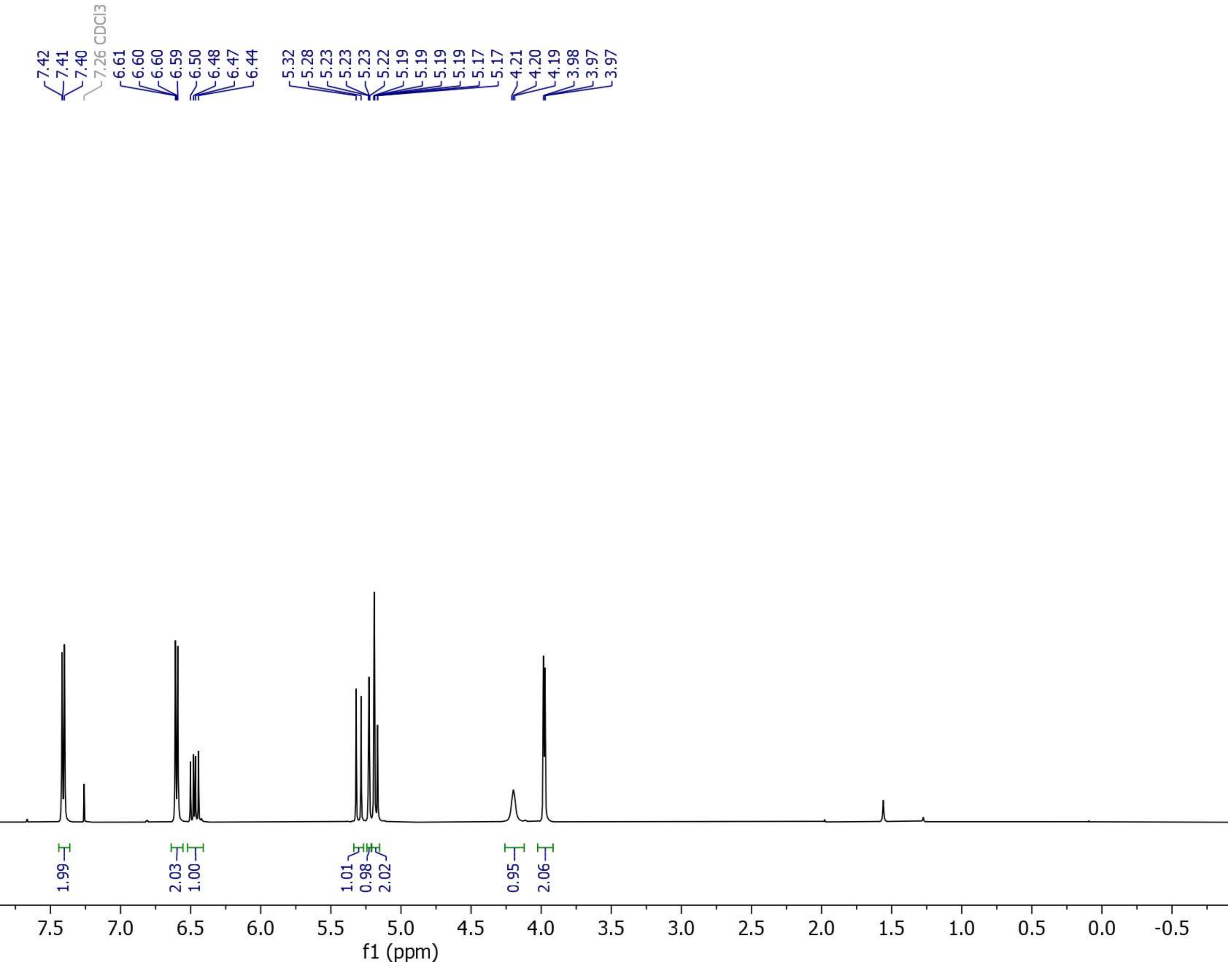

^{13}C NMR of 1,3-diene 11b

$CDCl_3$, 126 MHz, 23 °C

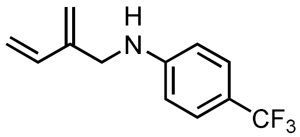

11b

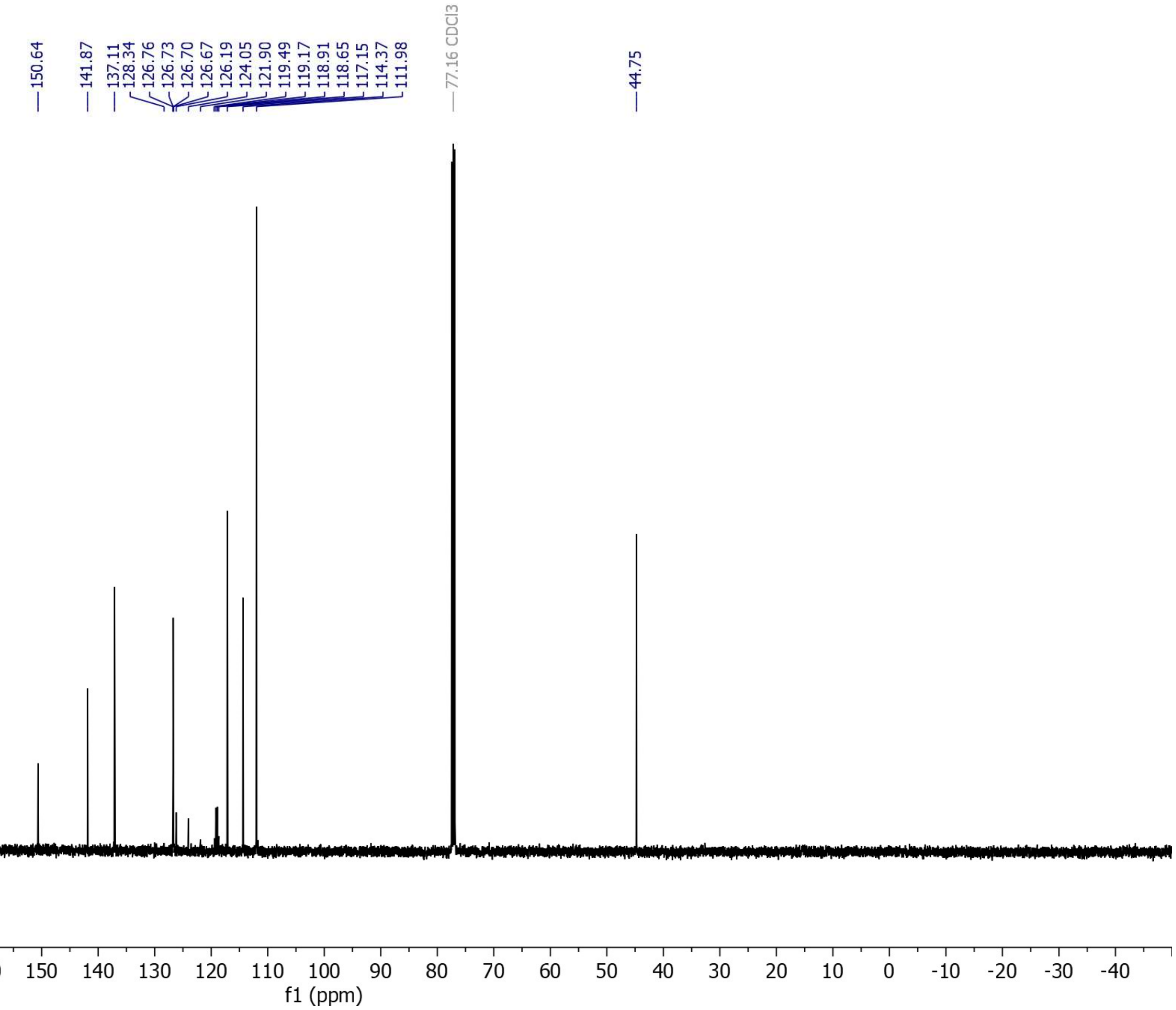

^{19}F NMR of 1,3-diene 11b

$CDCl_3$, 476 MHz, 23 °C

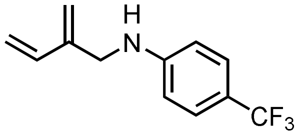

11b

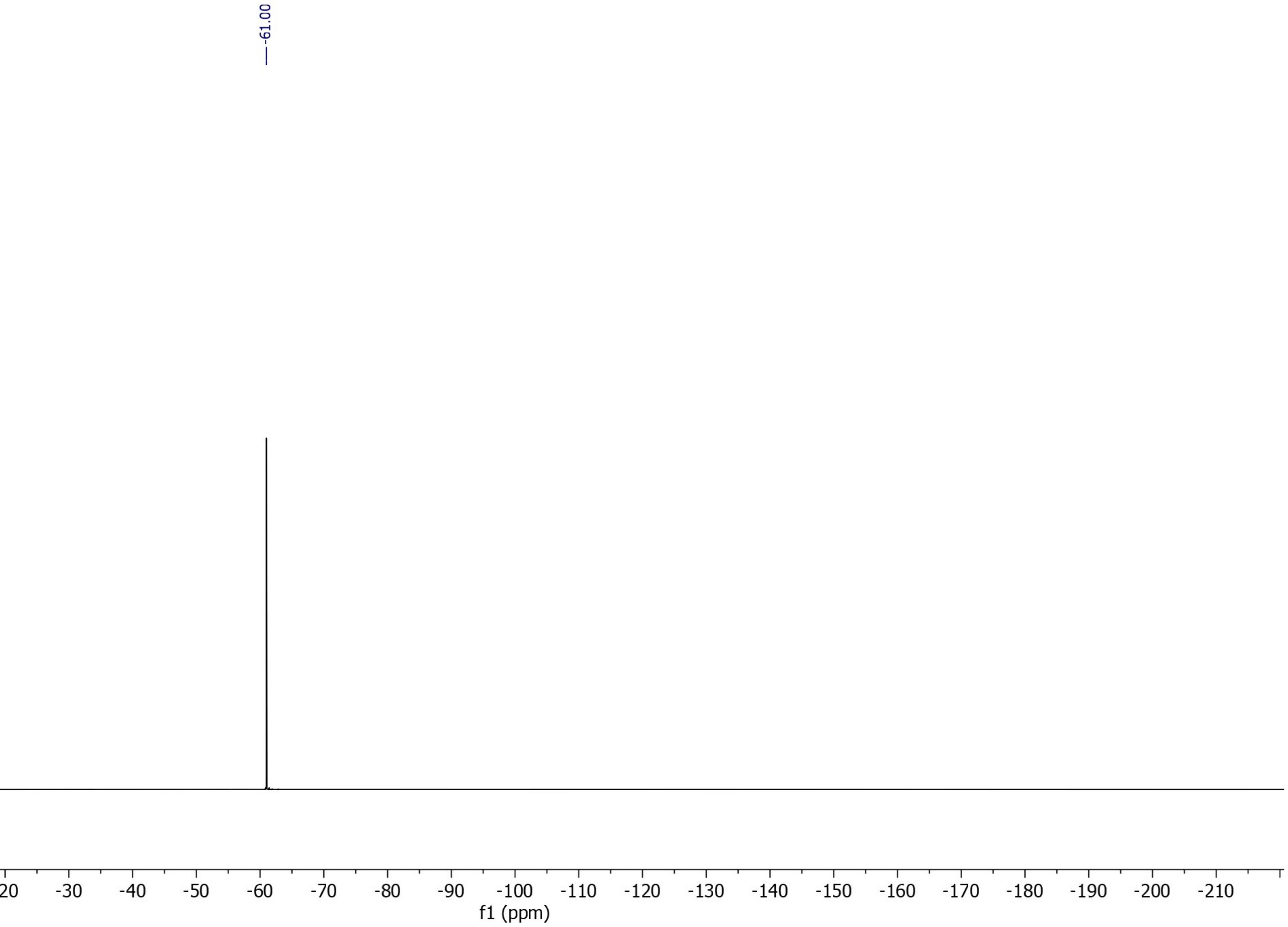

^{1}H NMR of 1,3-diene 11c

$CDCl_3$, 500 MHz, 23 °C

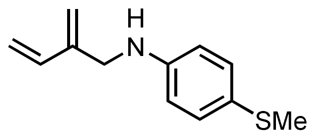

11c

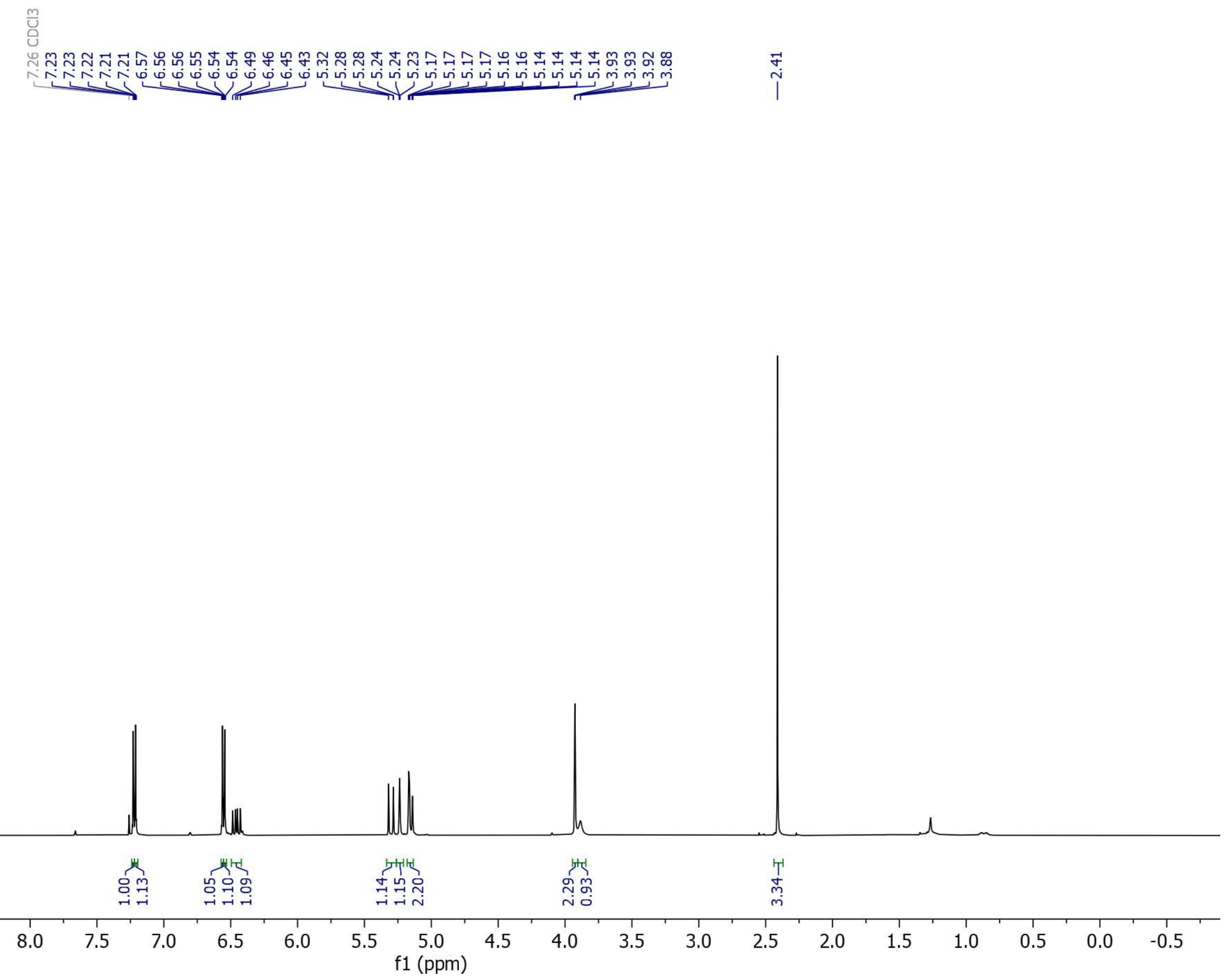

^{13}C NMR of 1,3-diene 11c

CDCl$_3$, 126 MHz, 23 °C

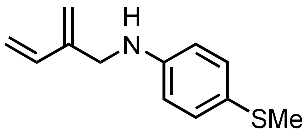

11c

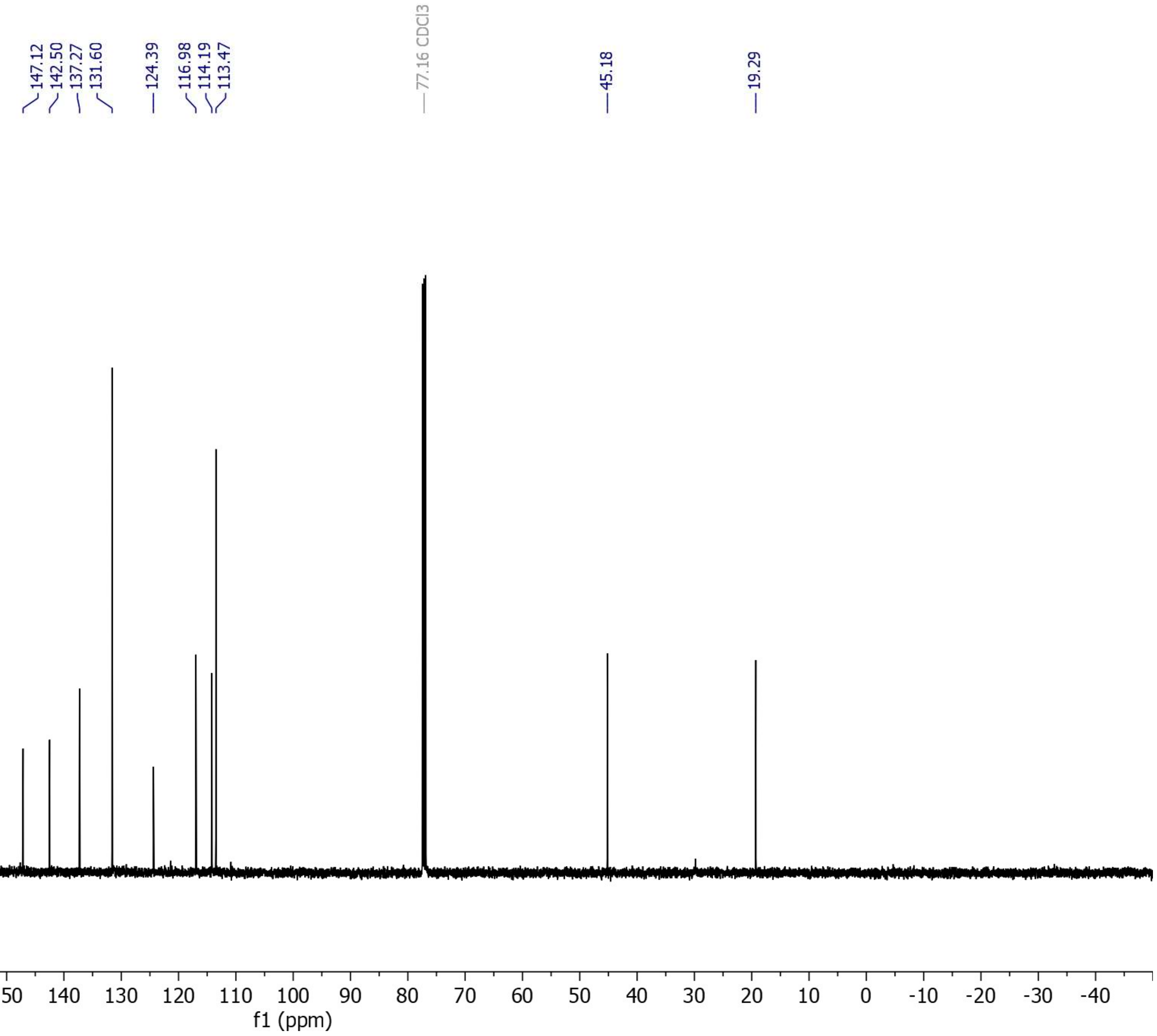

^{1}H NMR of 1,3-diene (±)-12

CDCl$_3$, 600 MHz, 23 °C

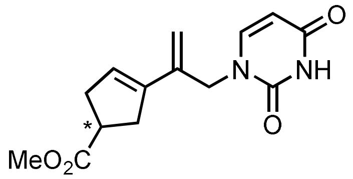

(±)-**12**

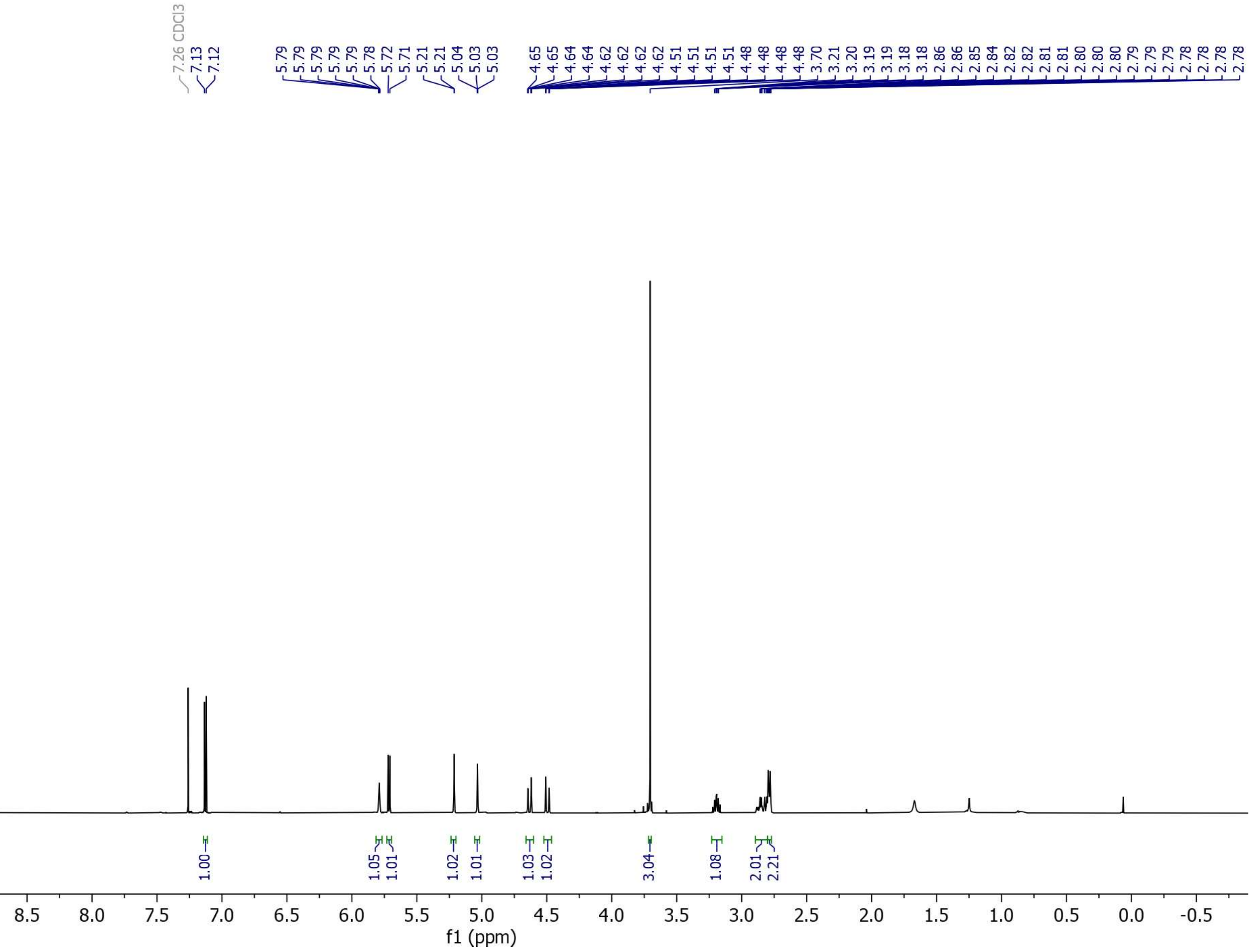

^{13}C NMR of 1,3-diene (±)-12

CDCl$_3$, 151 MHz, 23 °C

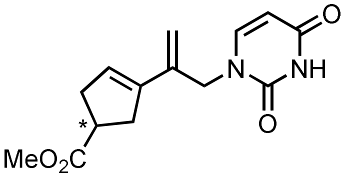

(±)-**12**

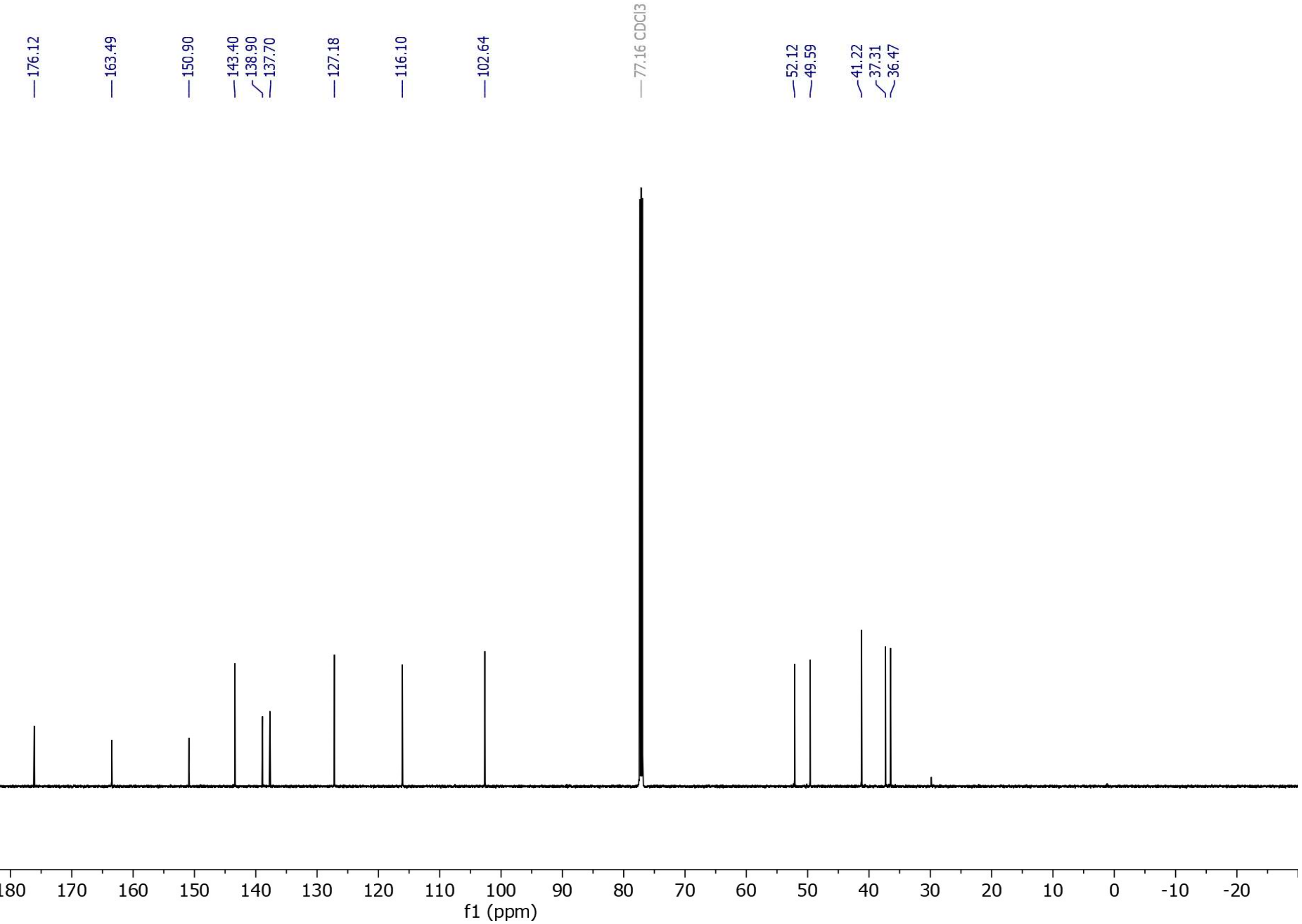

^{1}H NMR of 1,3-diene (±)-13

CDCl3, 500 MHz, 23 °C

(±)-**13**

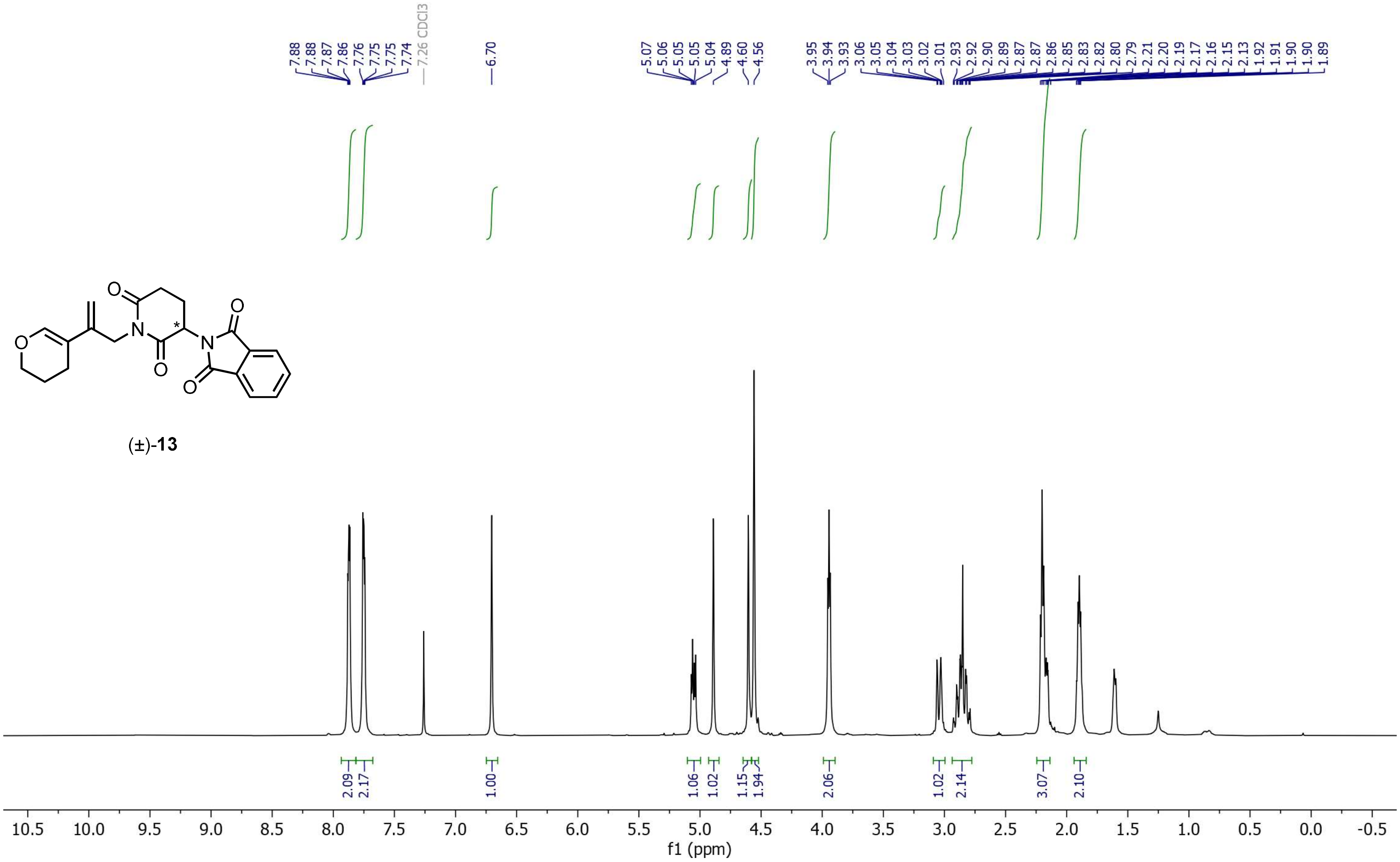

^{13}C NMR of 1,3-diene (±)-13

CDCl$_3$, 126 MHz, 23 °C

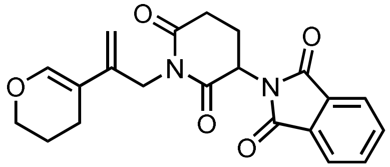

(±)-**13**

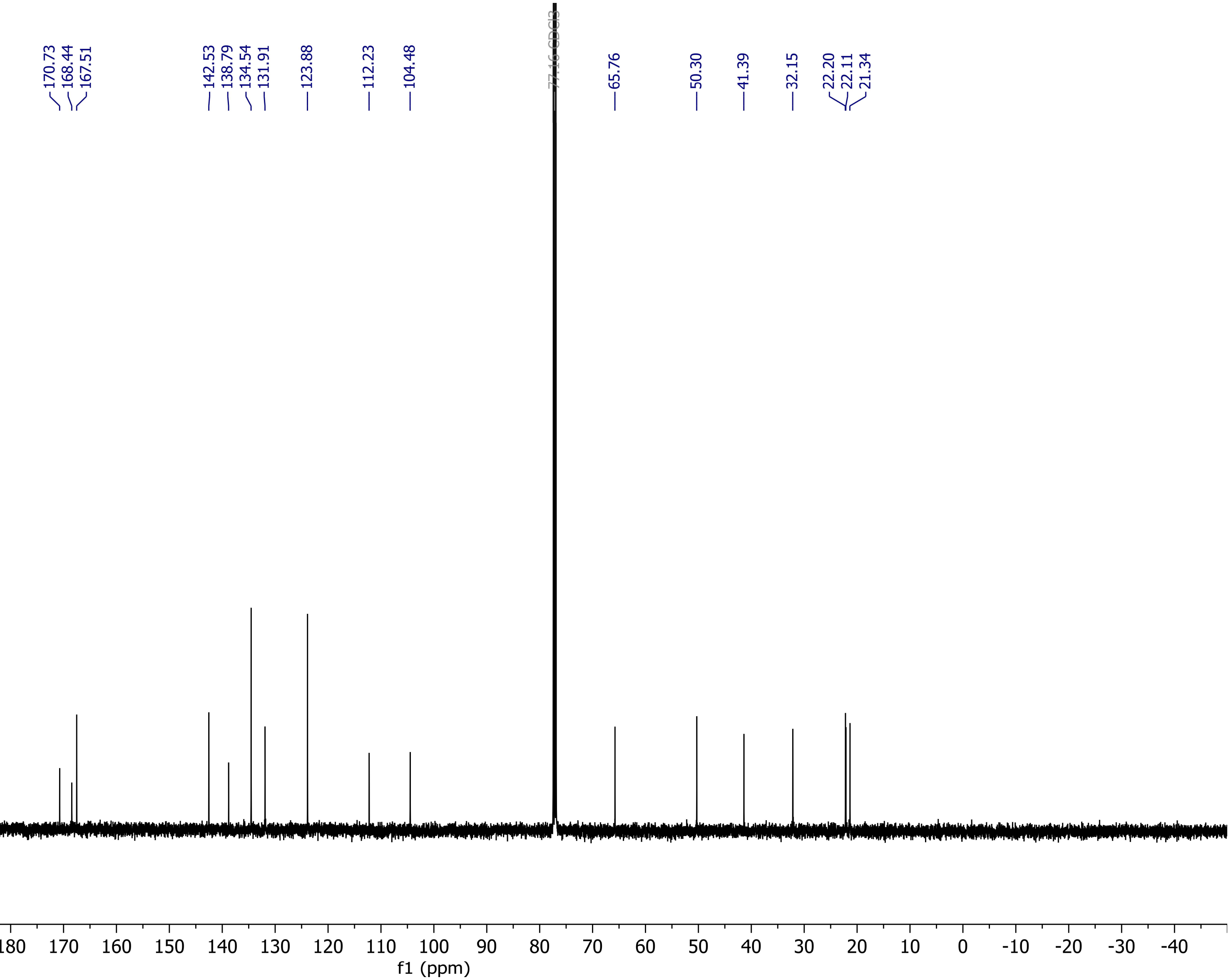

^{1}H NMR of 1,3-diene 14

$CDCl_3$, 500 MHz, 23 °C

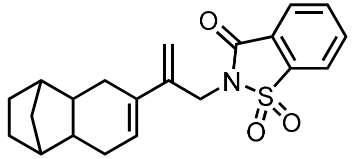

14

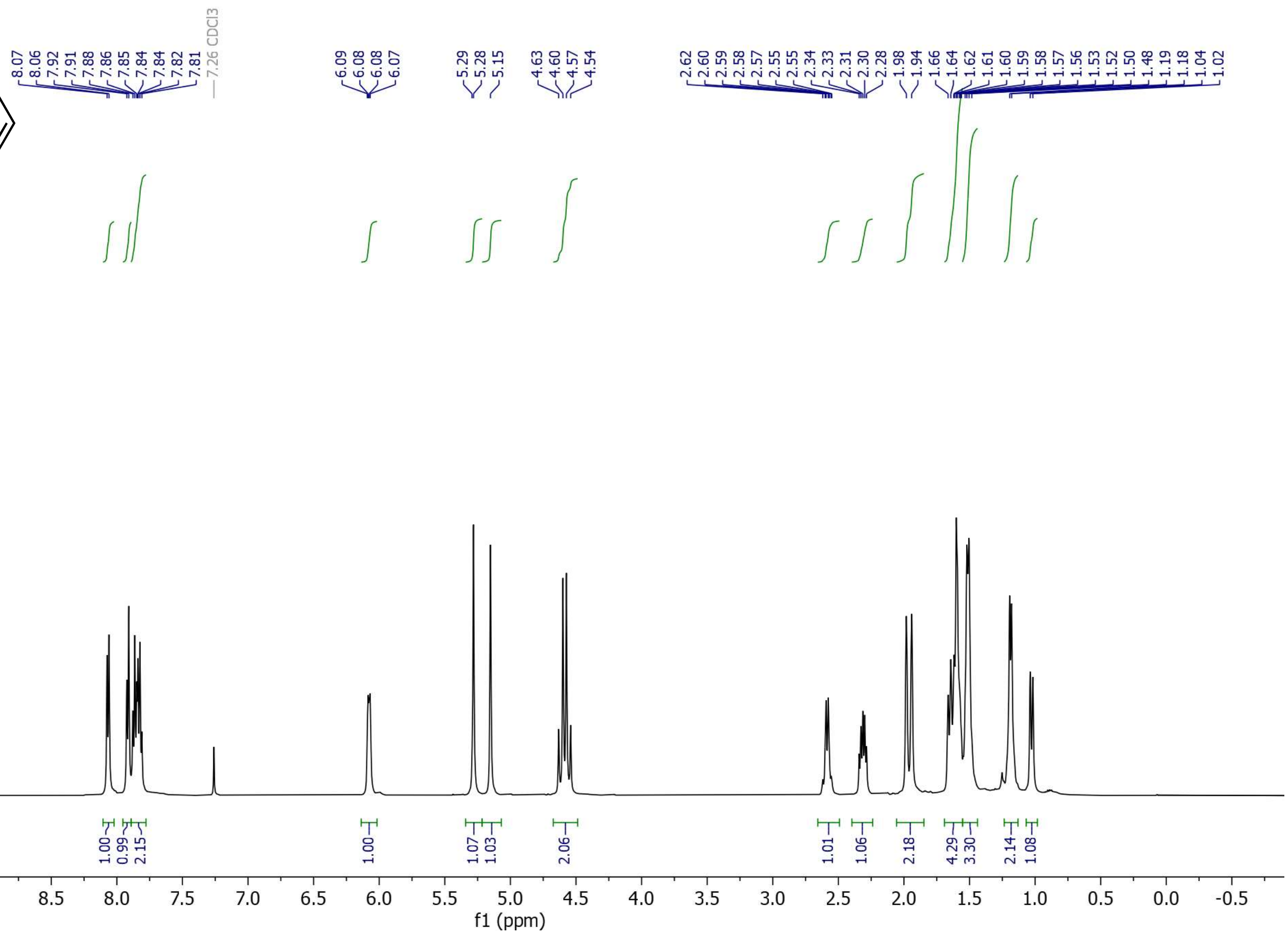

^{13}C NMR of 1,3-diene 14

$CDCl_3$, 126 MHz, 23 °C

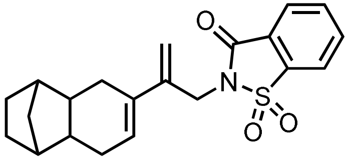

14

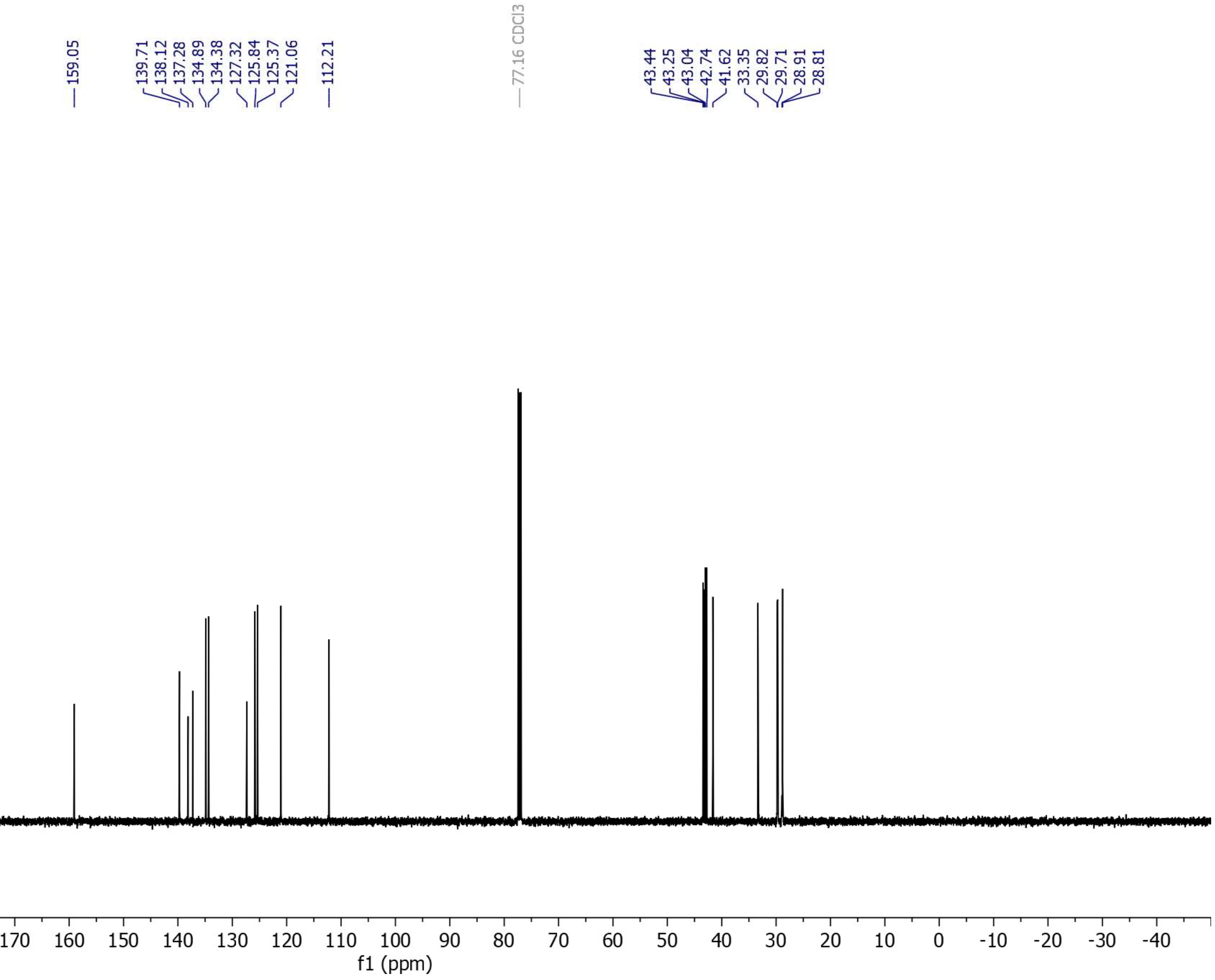

^{1}H NMR of 1,3-diene 15

$CDCl_3$, 500 MHz, 23 °C

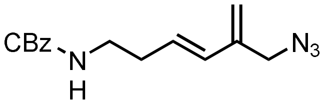

15

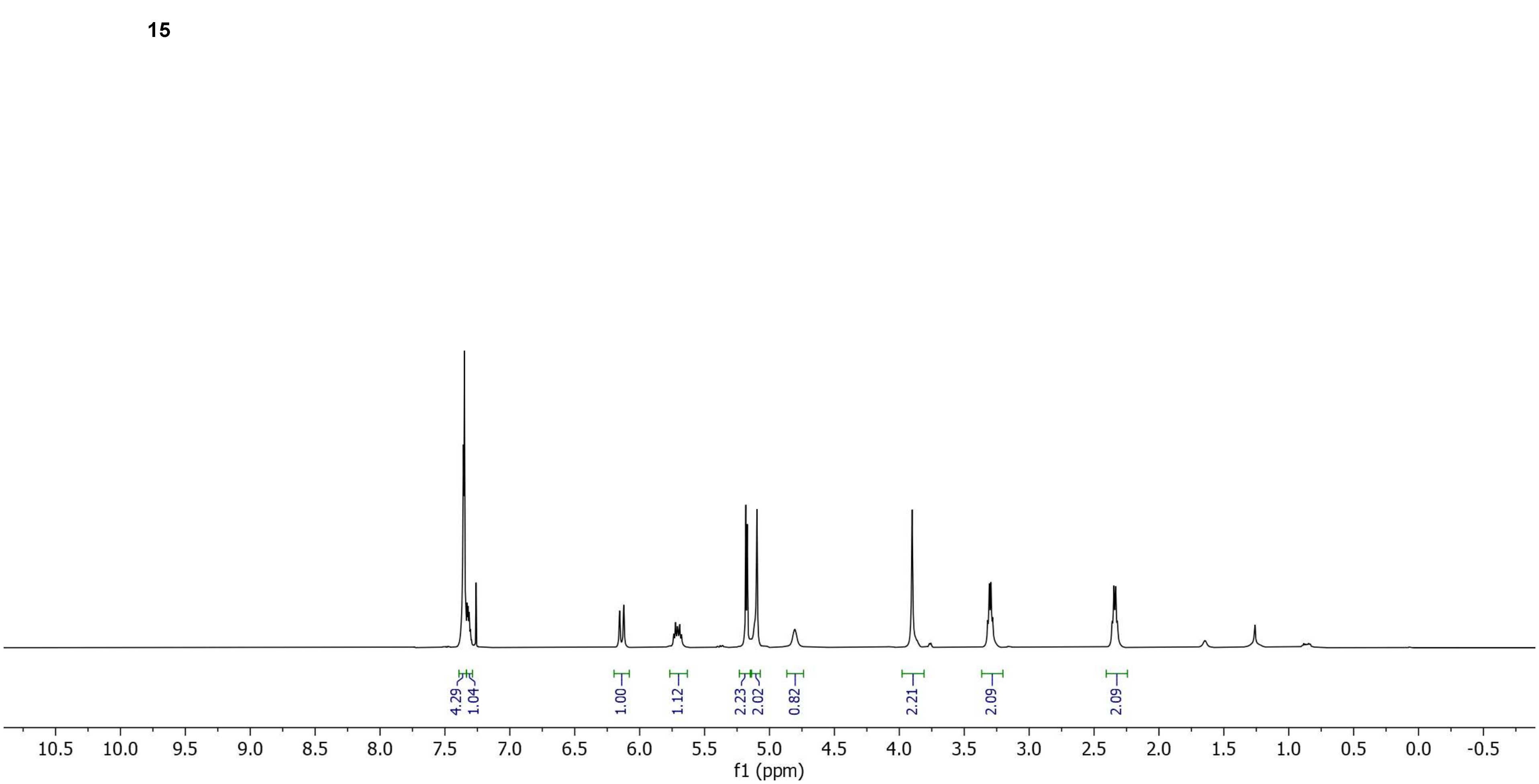

^{13}C NMR of 1,3-diene 15

$CDCl_3$, 126 MHz, 23 °C

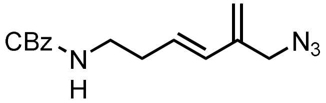

15

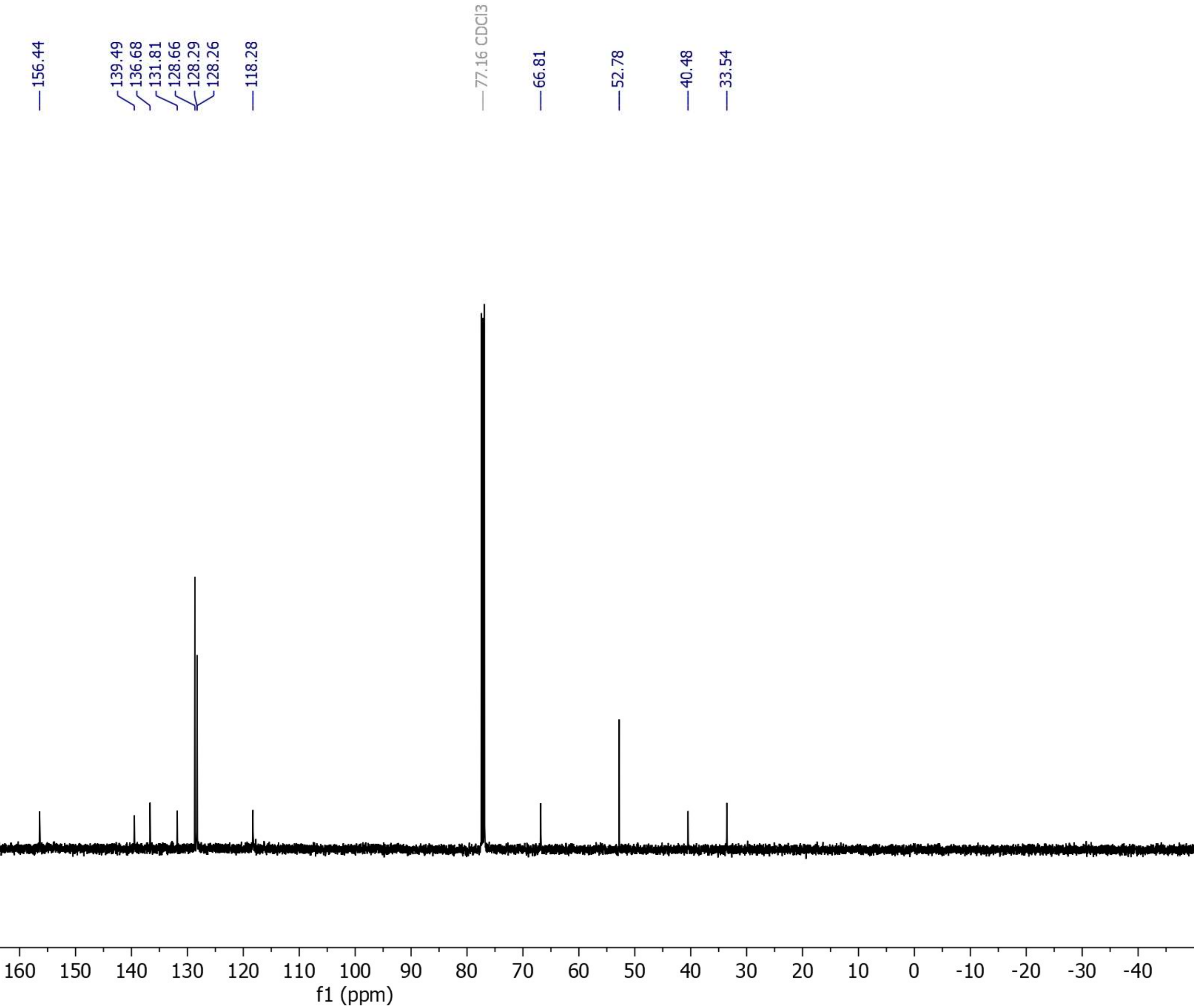

^{1}H NMR of 1,3-diene 16

$CDCl_3$, 500 MHz, 23 °C

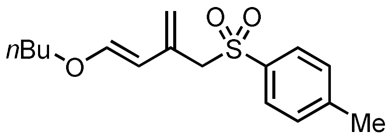

16

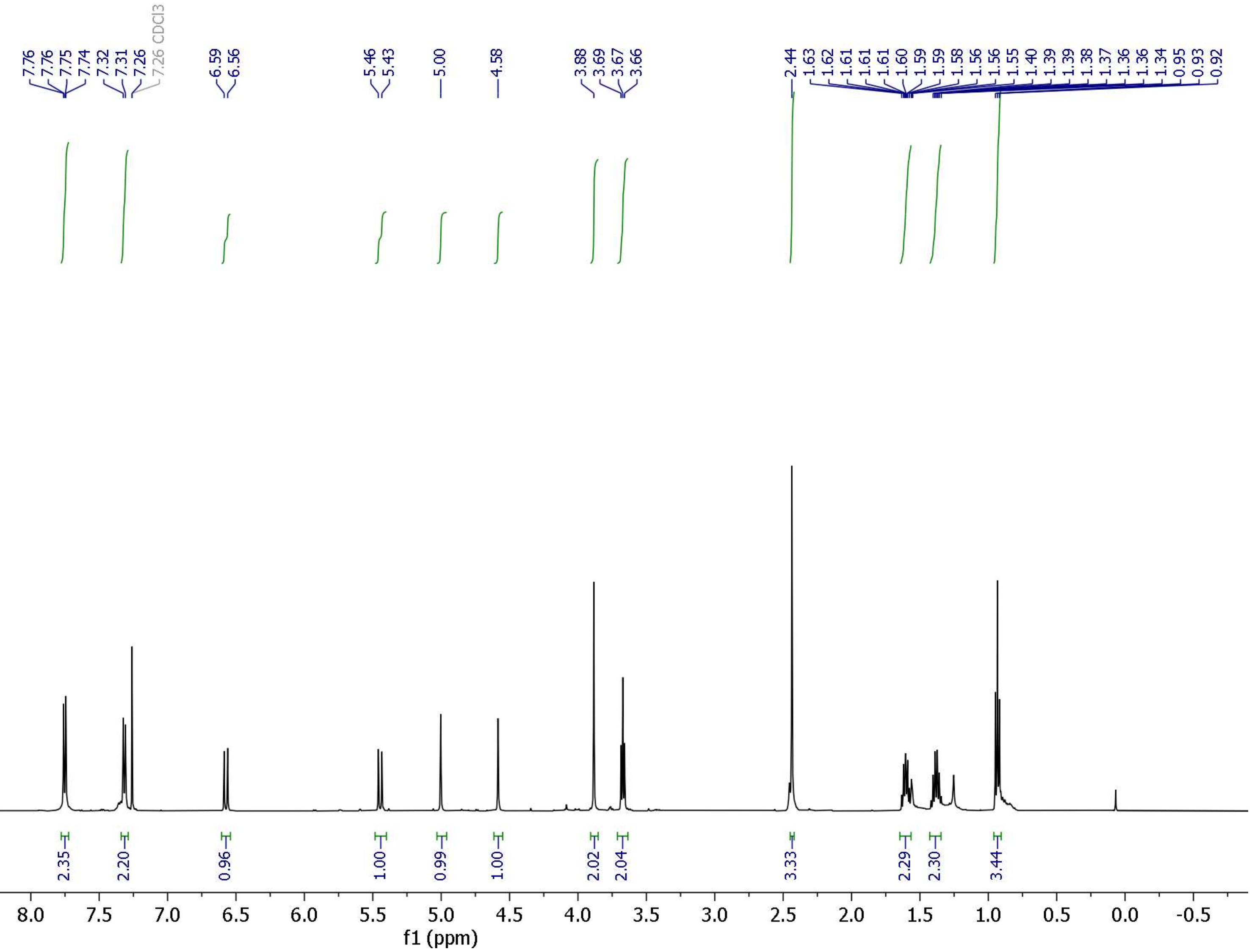

^{13}C NMR of 1,3-diene 16

$CDCl_3$, 126 MHz, 23 °C

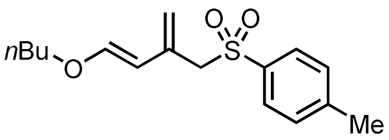

16

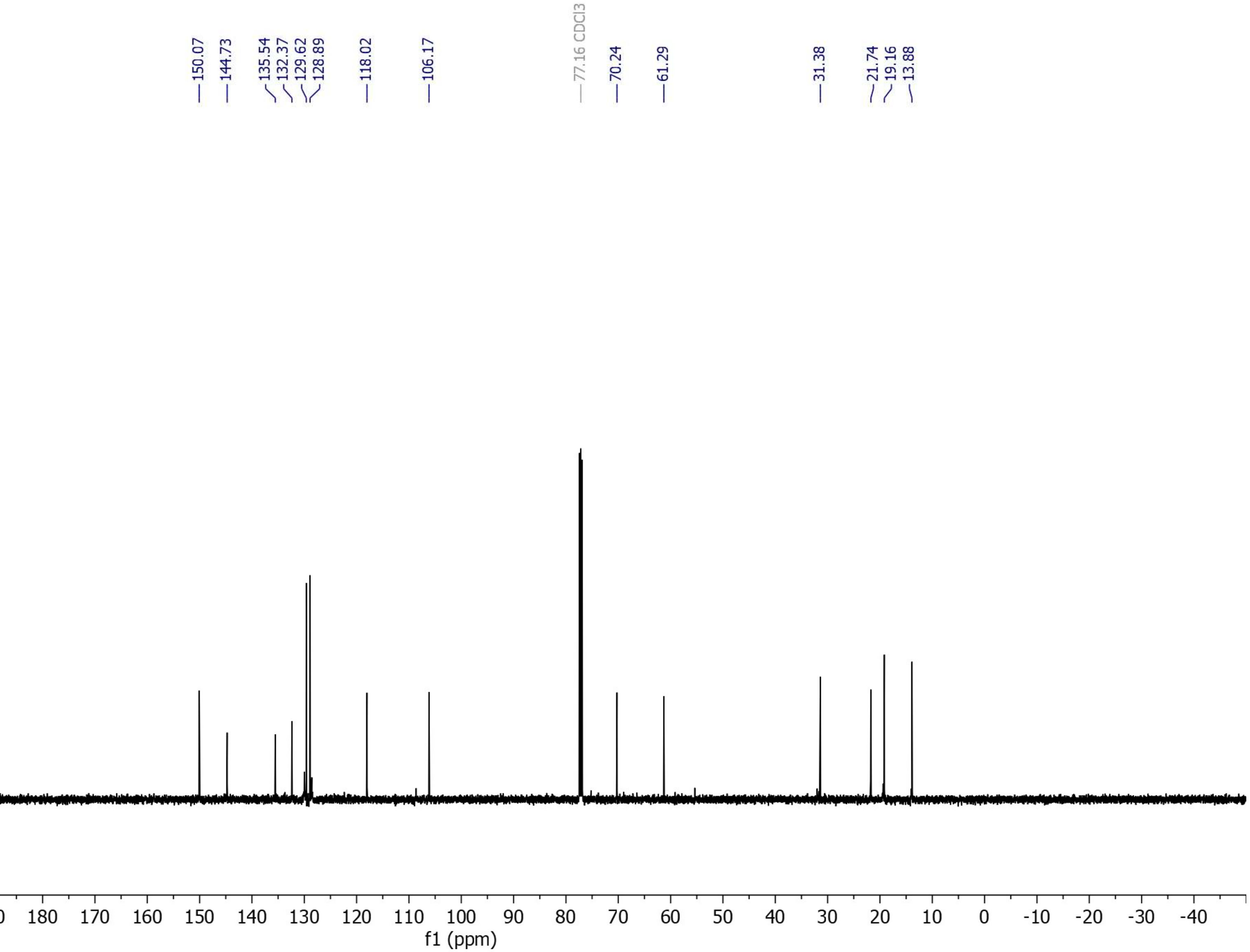

^{1}H NMR of 1,3-diene 17

CDCl$_3$, 500 MHz, 23 °C

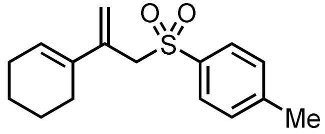

17

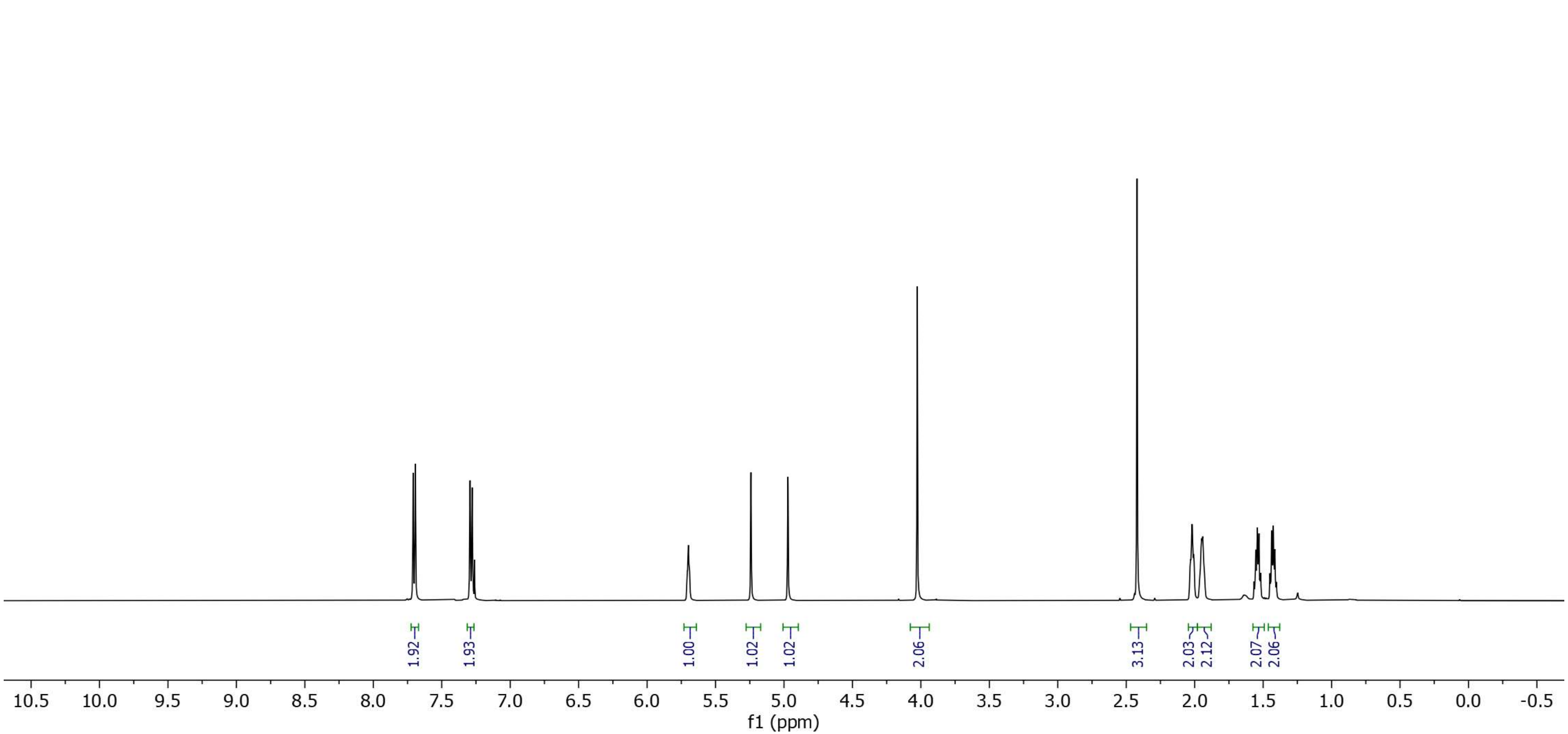

^{13}C NMR of 1,3-diene 17

$CDCl_3$, 126 MHz, 23 °C

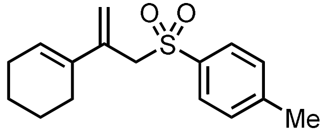

17

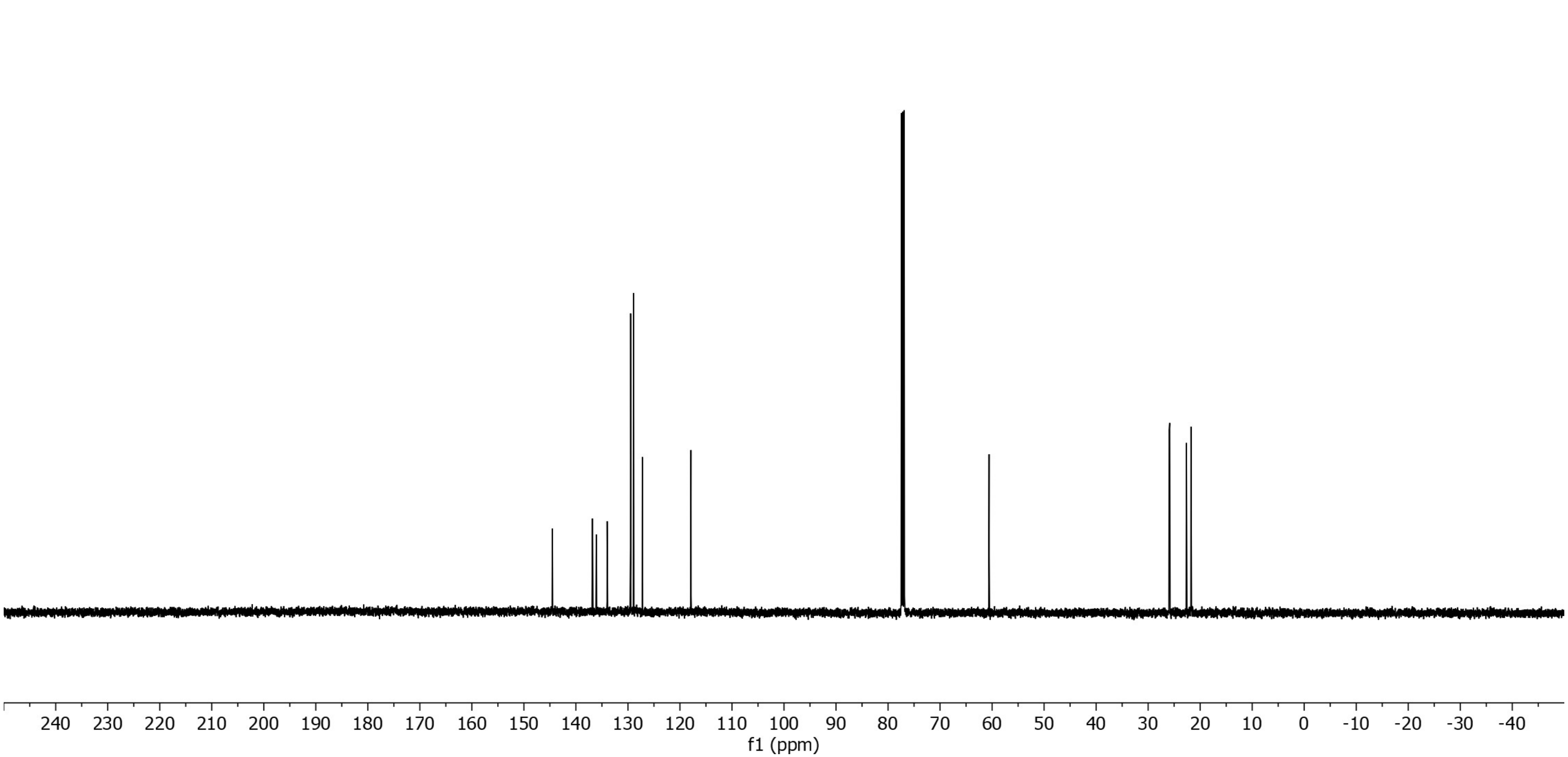

^{1}H NMR of 1,3-diene 18a

$CDCl_3$, 500 MHz, 23 °C

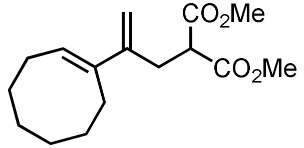

18a

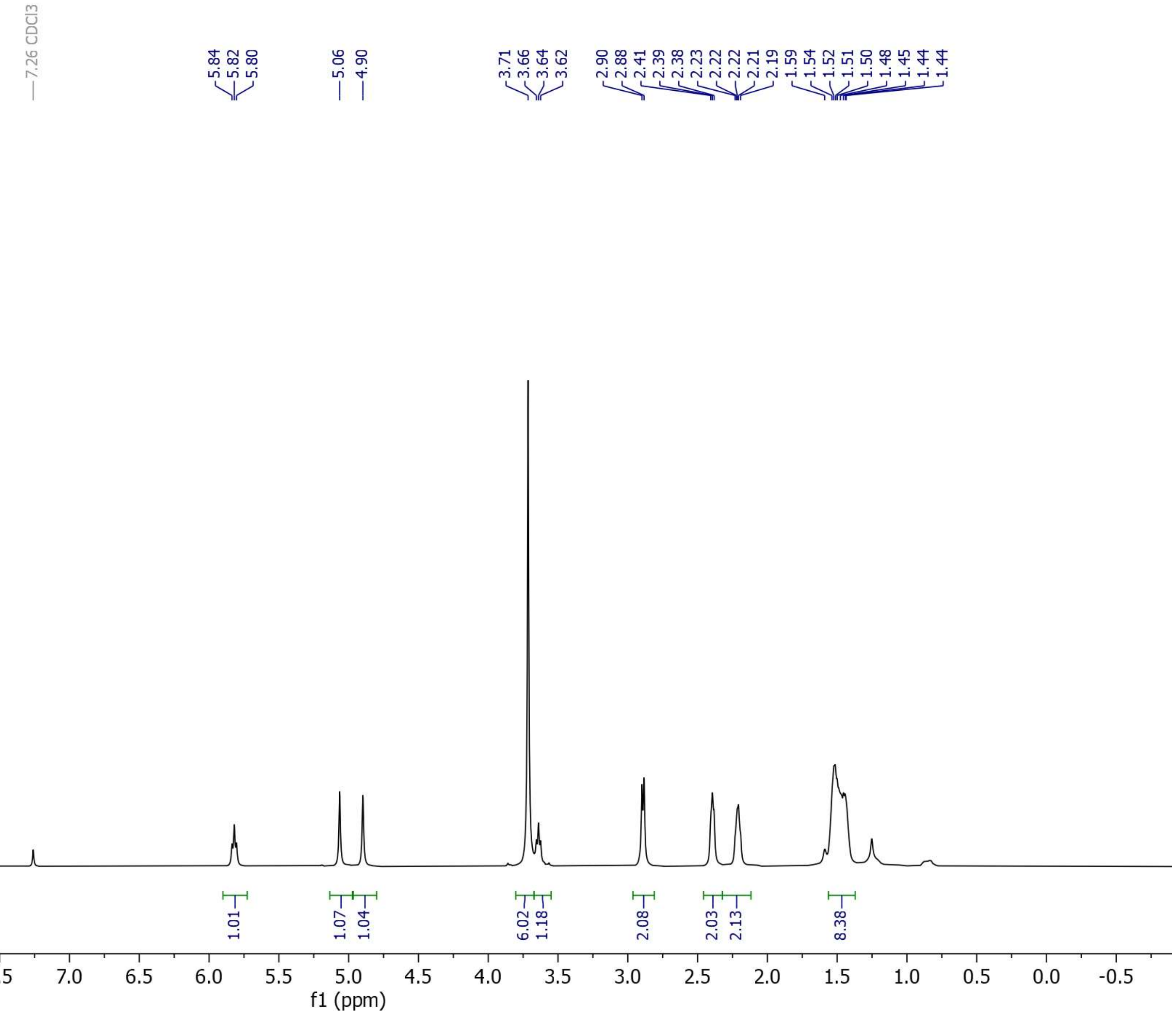

^{13}C NMR of 1,3-diene 18a

$CDCl_3$, 126 MHz, 23 °C

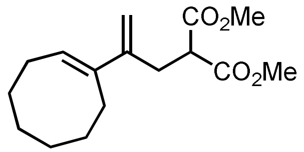

18a

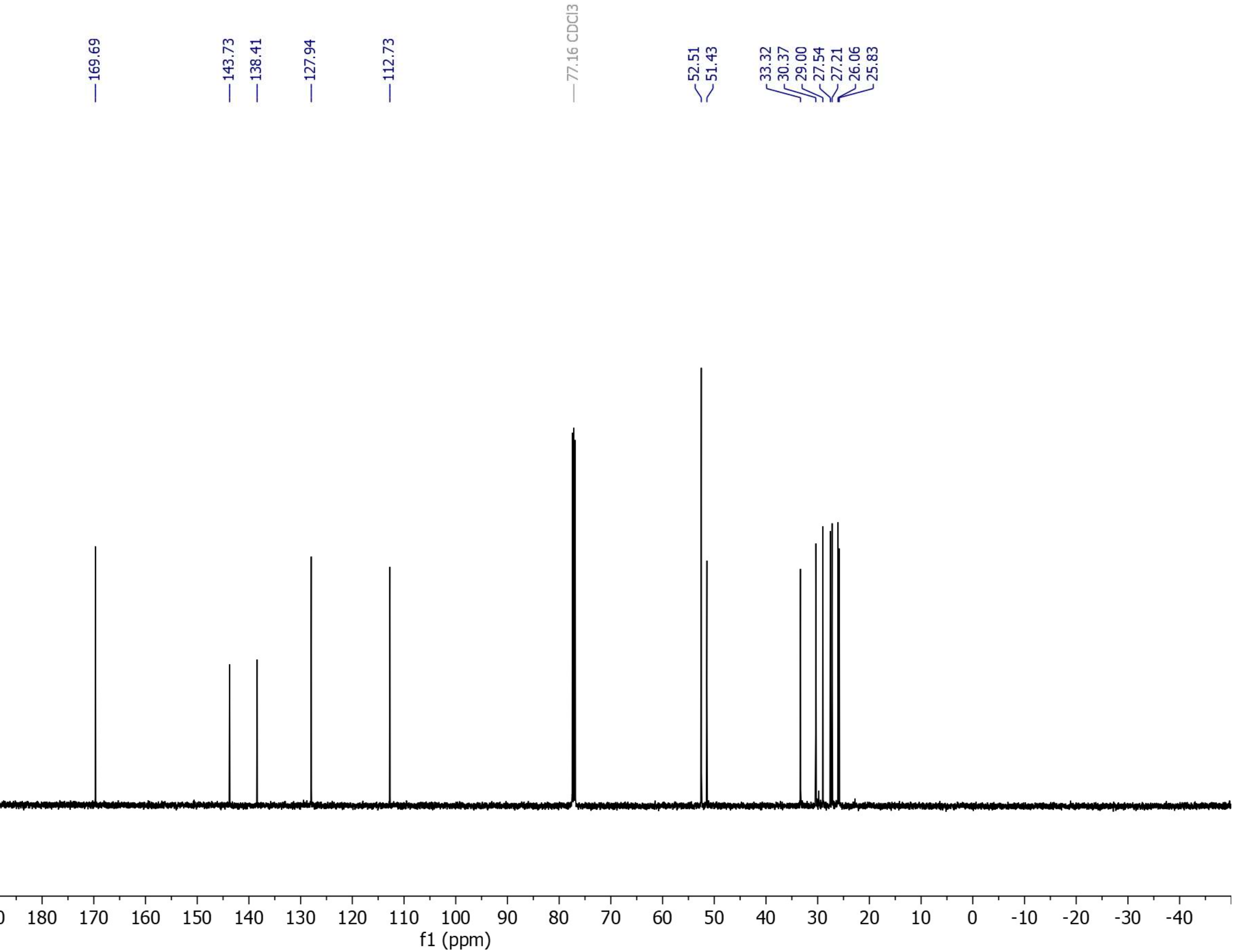

^{1}H NMR of 1,3-diene 18b

$CDCl_3$, 500 MHz, 23 °C

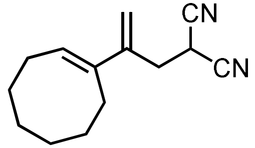

18b

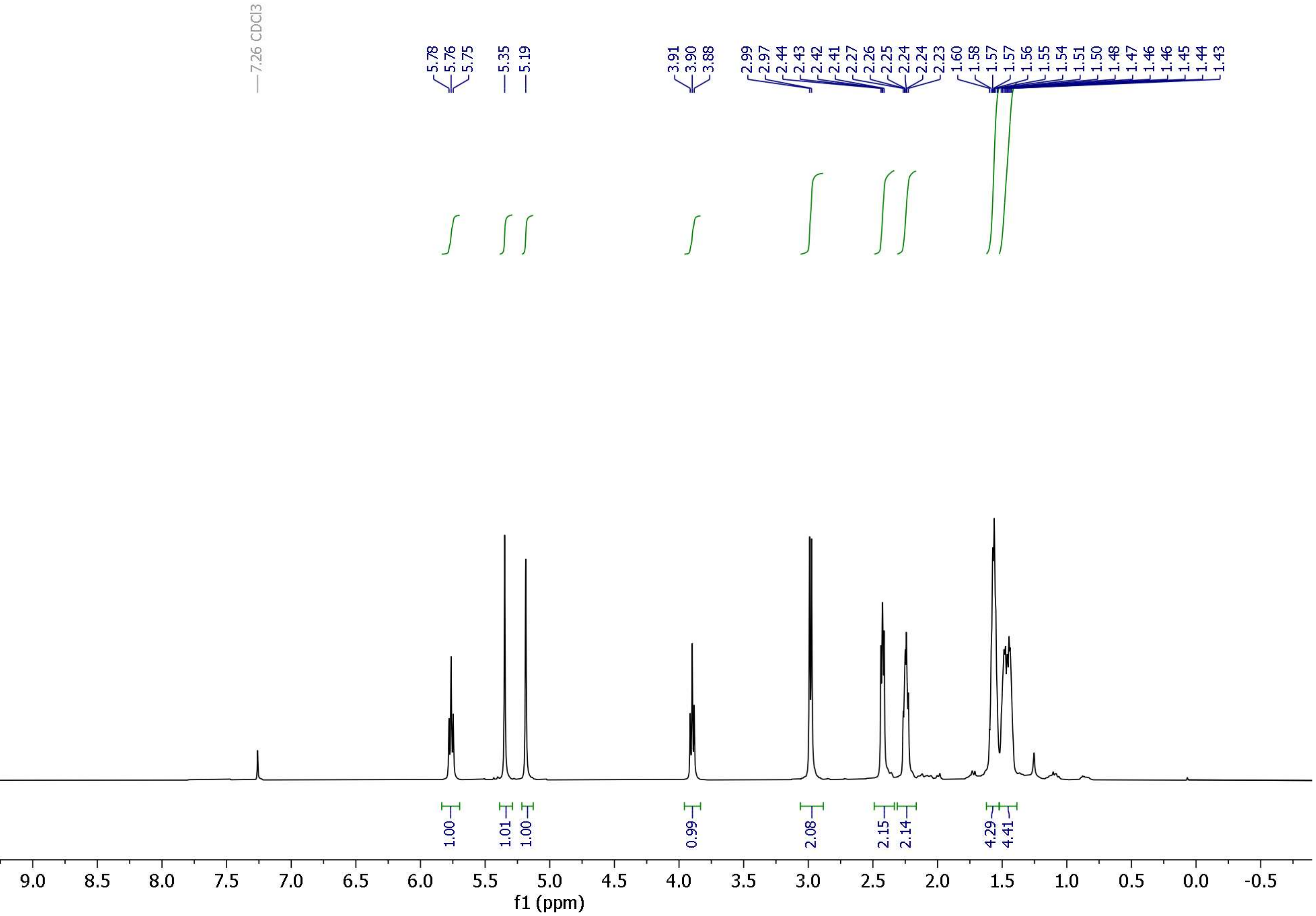

^{13}C NMR of 1,3-diene 18b

CDCl$_3$, 126 MHz, 23 °C

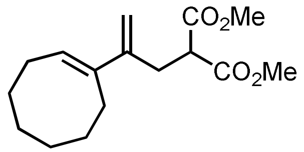

18a

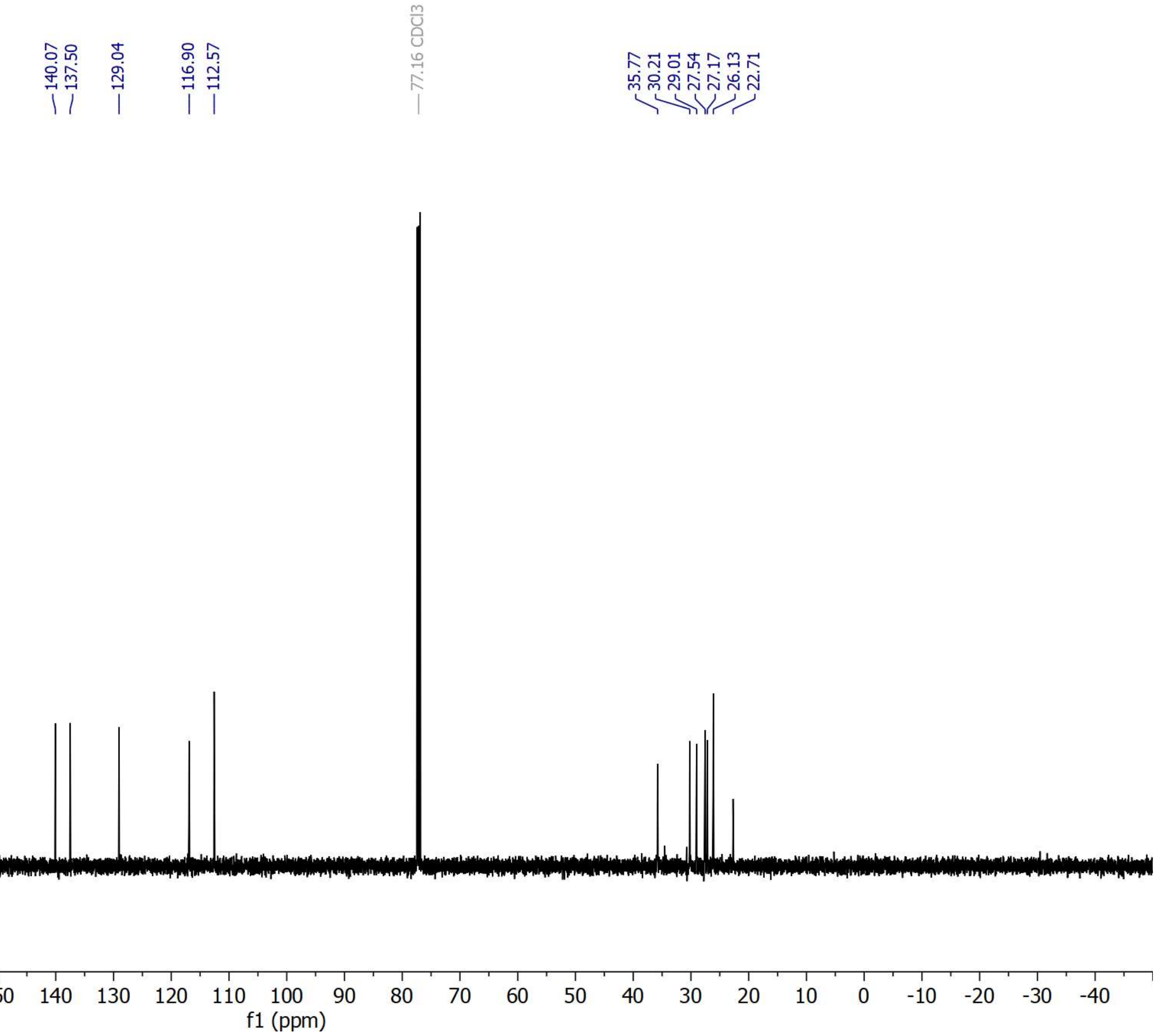

^{1}H NMR of 1,3-diene (±)-18c

CDCl3, 500 MHz, 23 °C

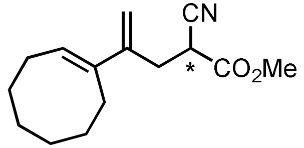

(±)-**18c**

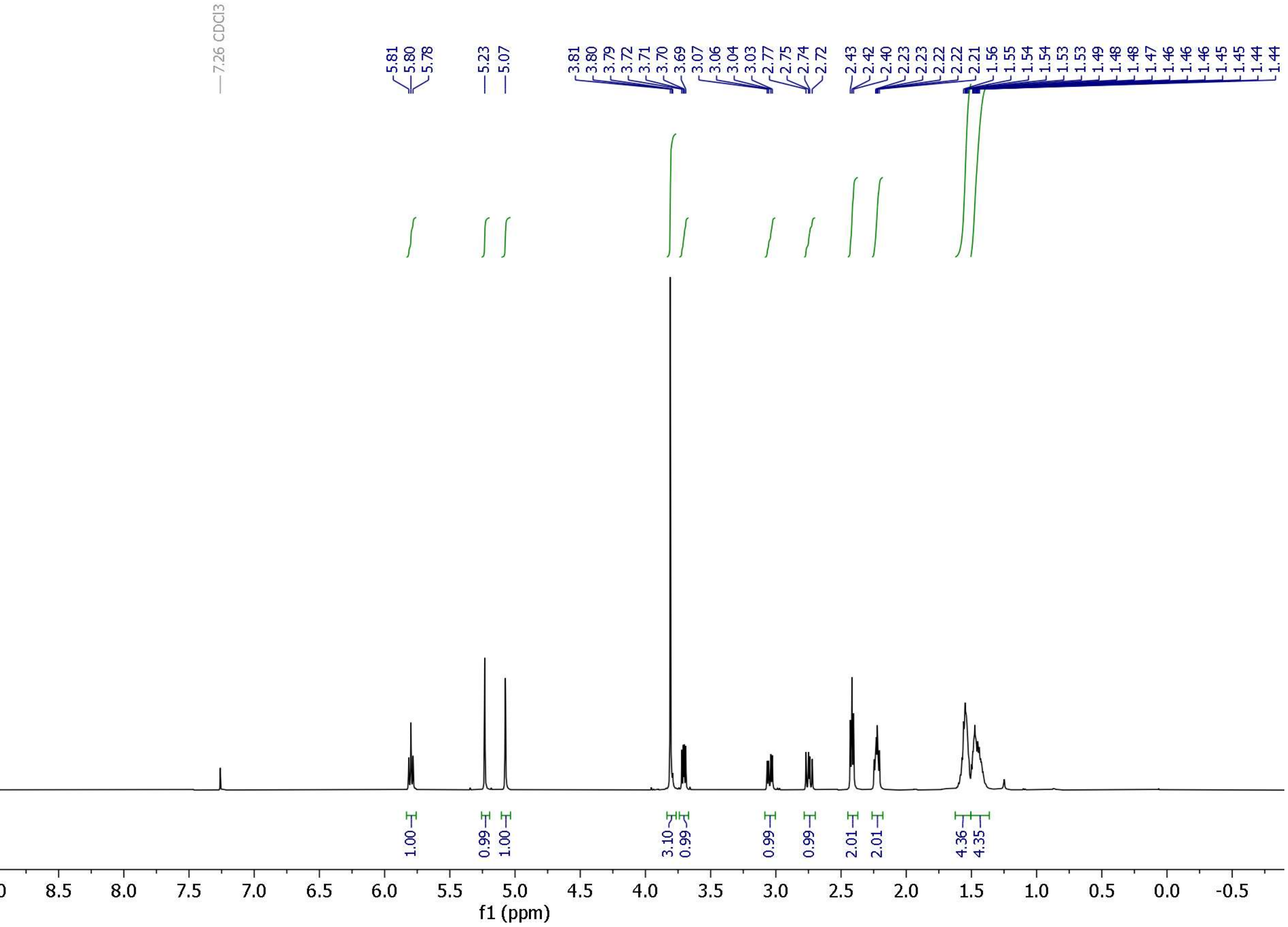

^{13}C NMR of 1,3-diene (±)-18c

$CDCl_3$, 151 MHz, 23 °C

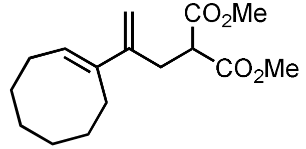

18a

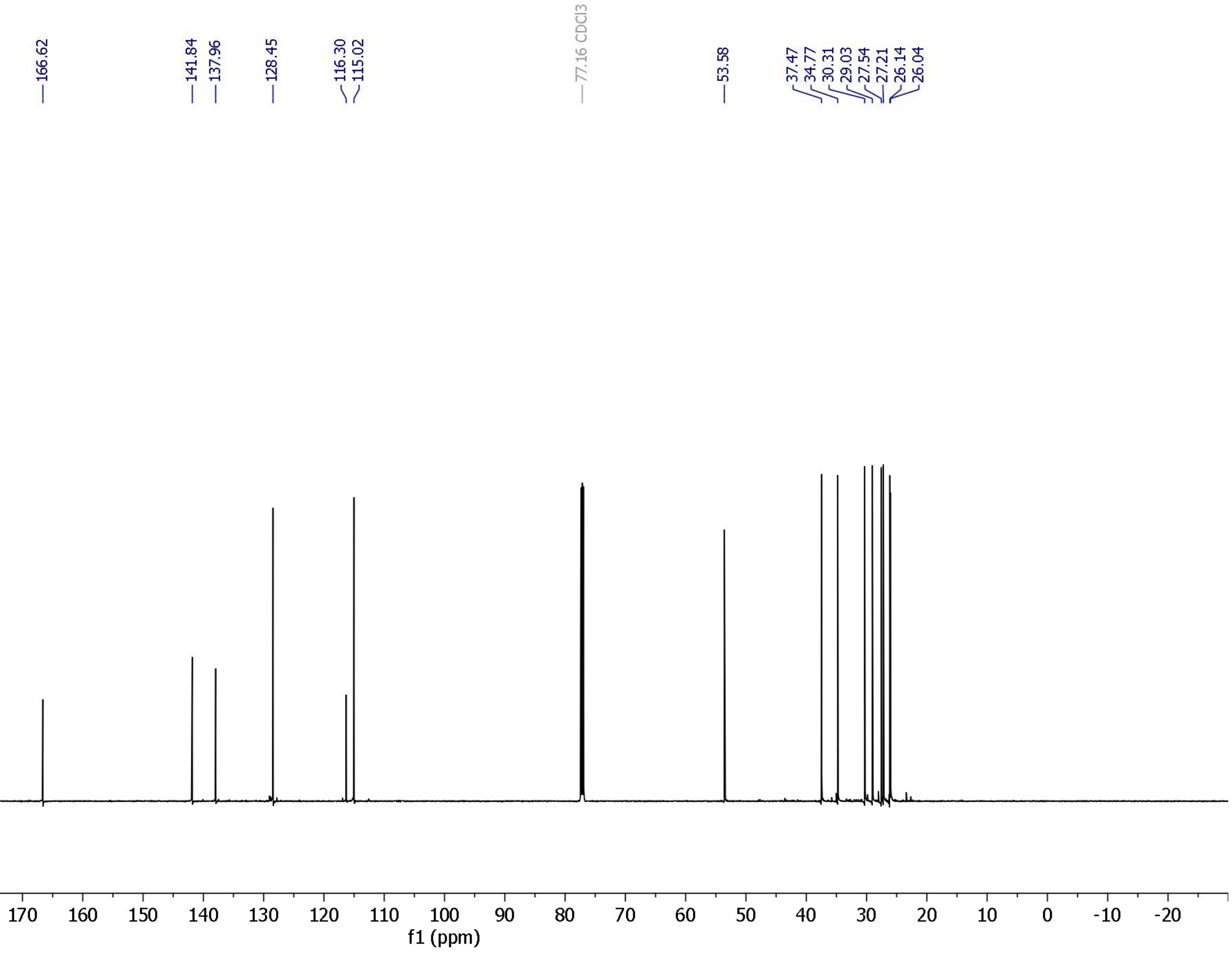

^{1}H NMR of 1,3-diene 18d

$CDCl_3$, 500 MHz, 23 °C

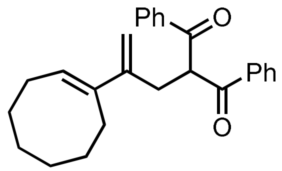

18d

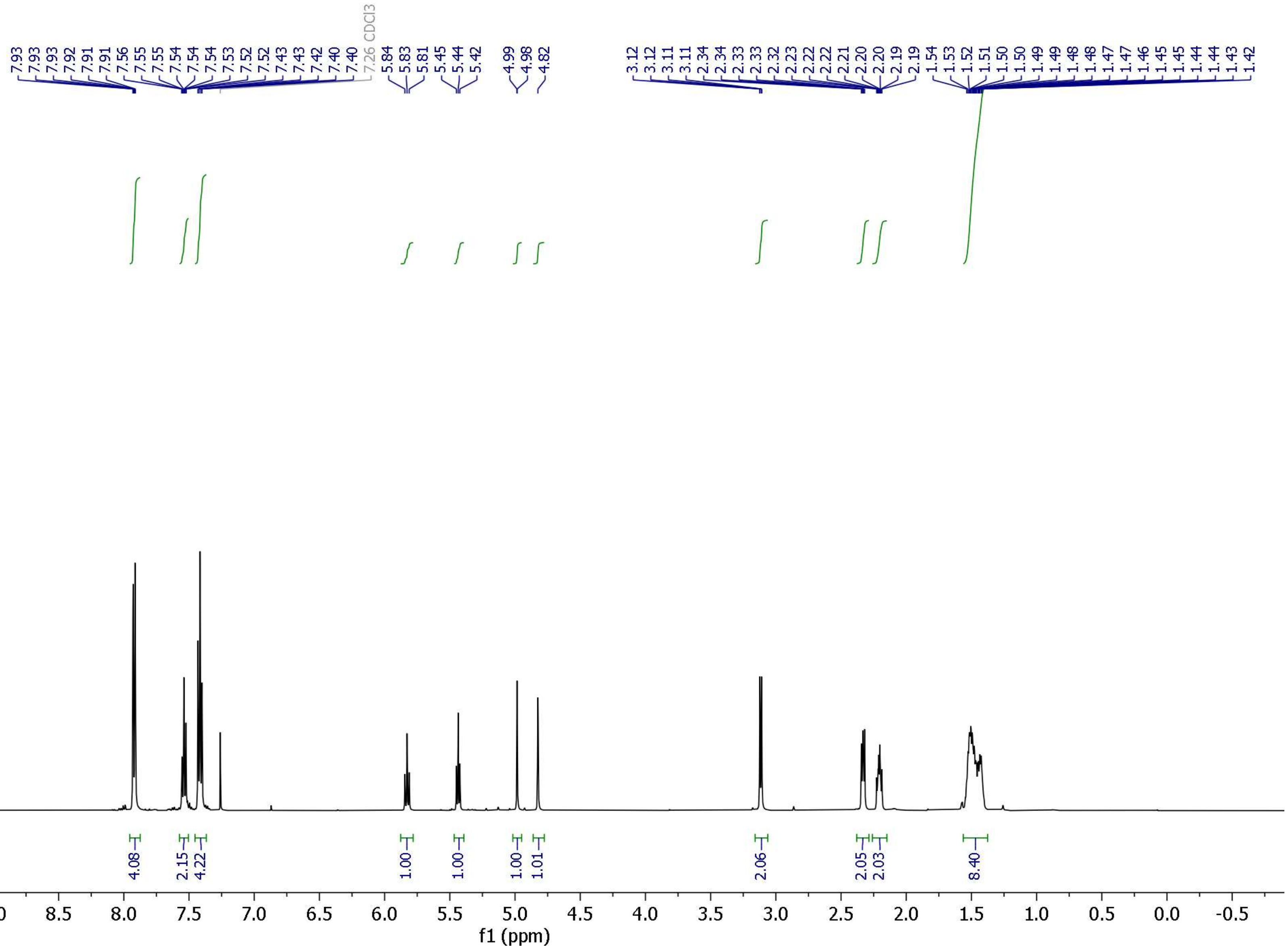

^{13}C NMR of 1,3-diene 18d

$CDCl_3$, 126 MHz, 23 °C

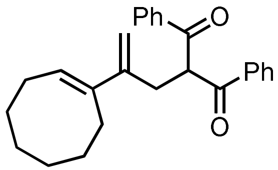

18d

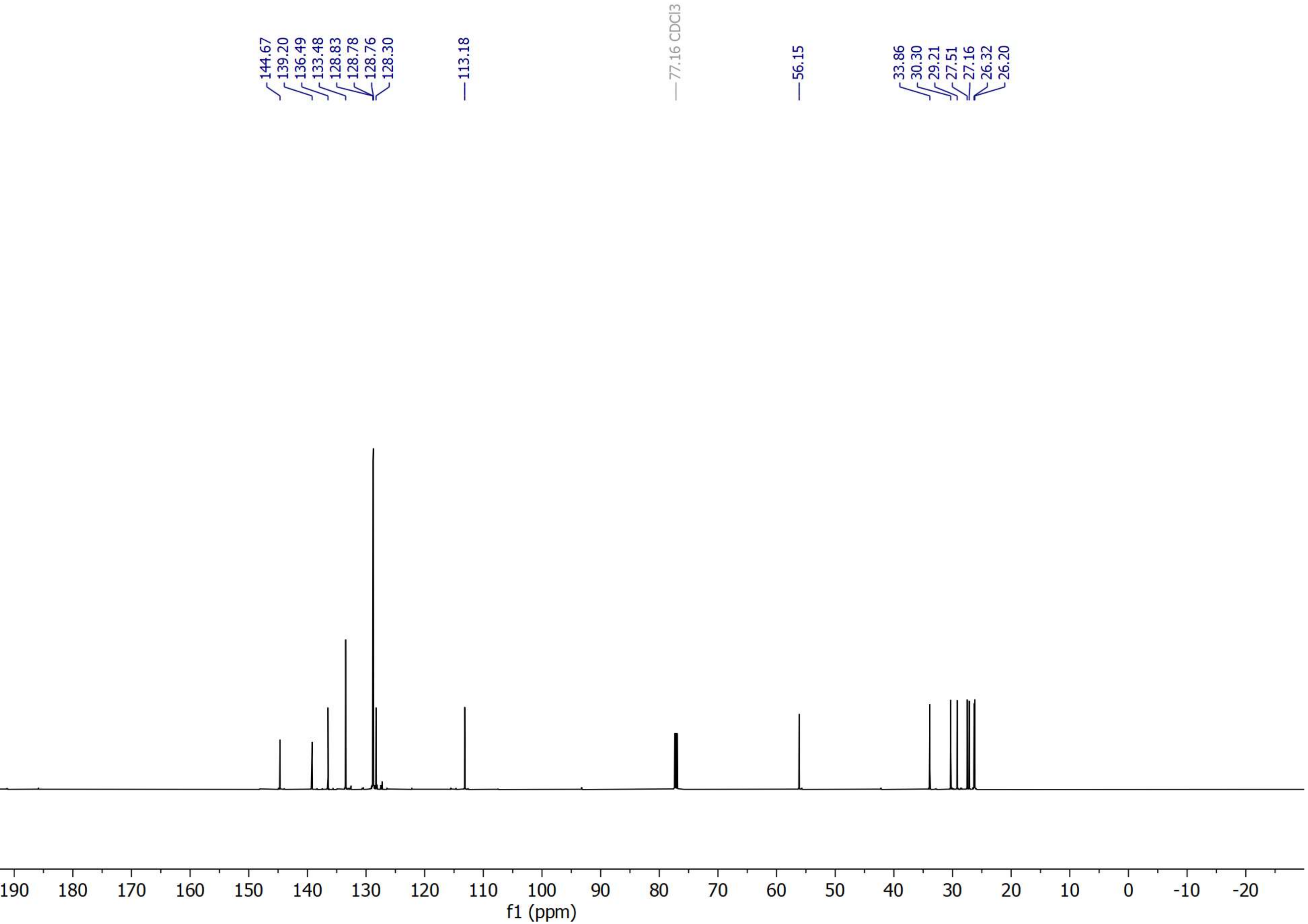

^{1}H NMR of 1,3-diene 21a

CDCl$_3$, 500 MHz, 23 °C

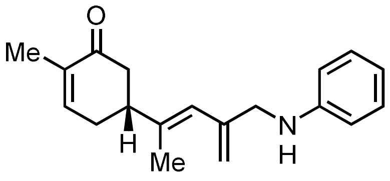

21a

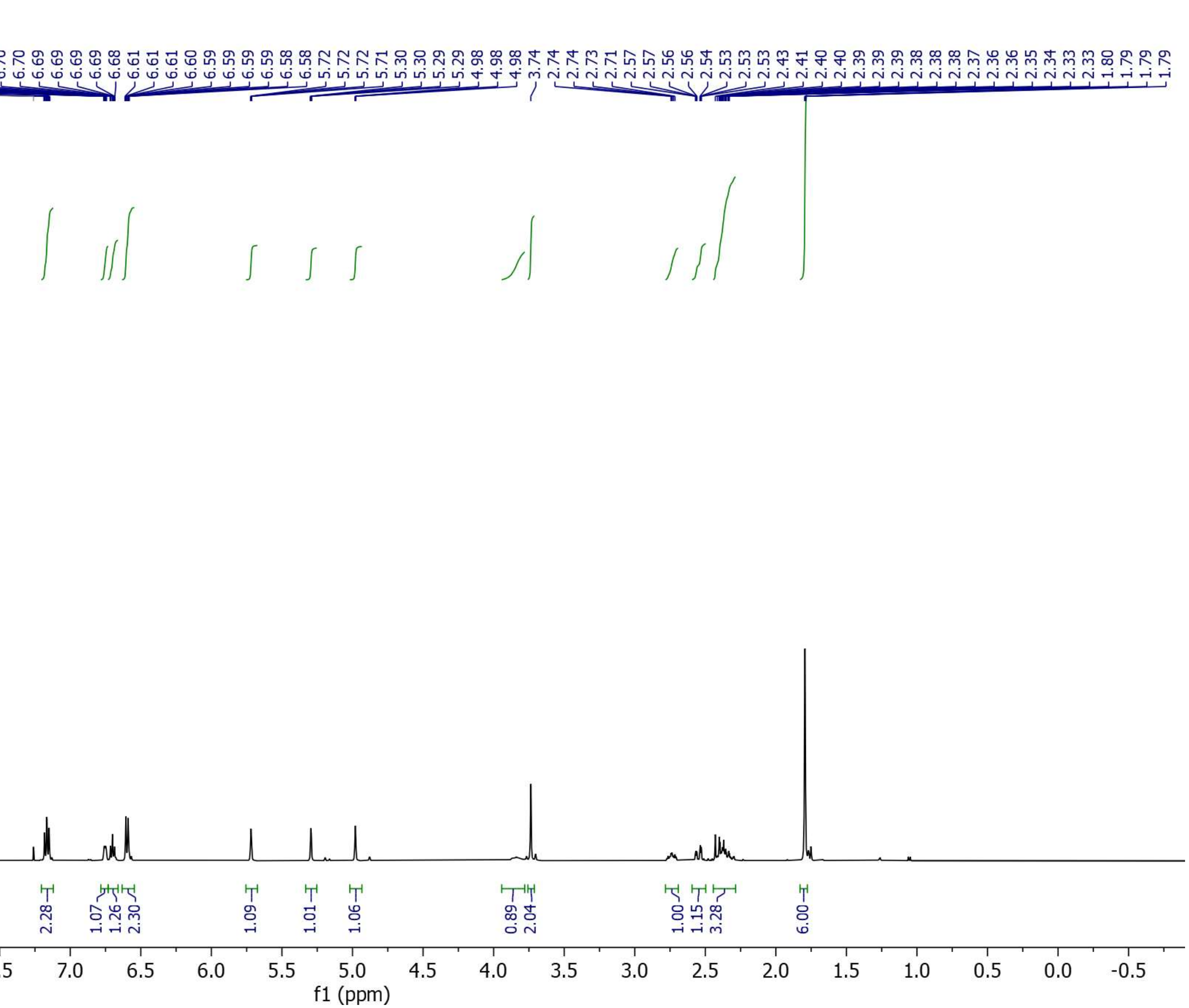

^{13}C NMR of 1,3-diene 21a

CDCl$_3$, 126 MHz, 23 °C

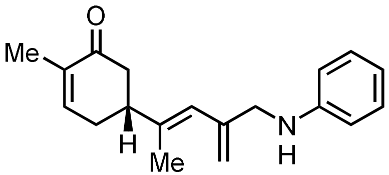

21a

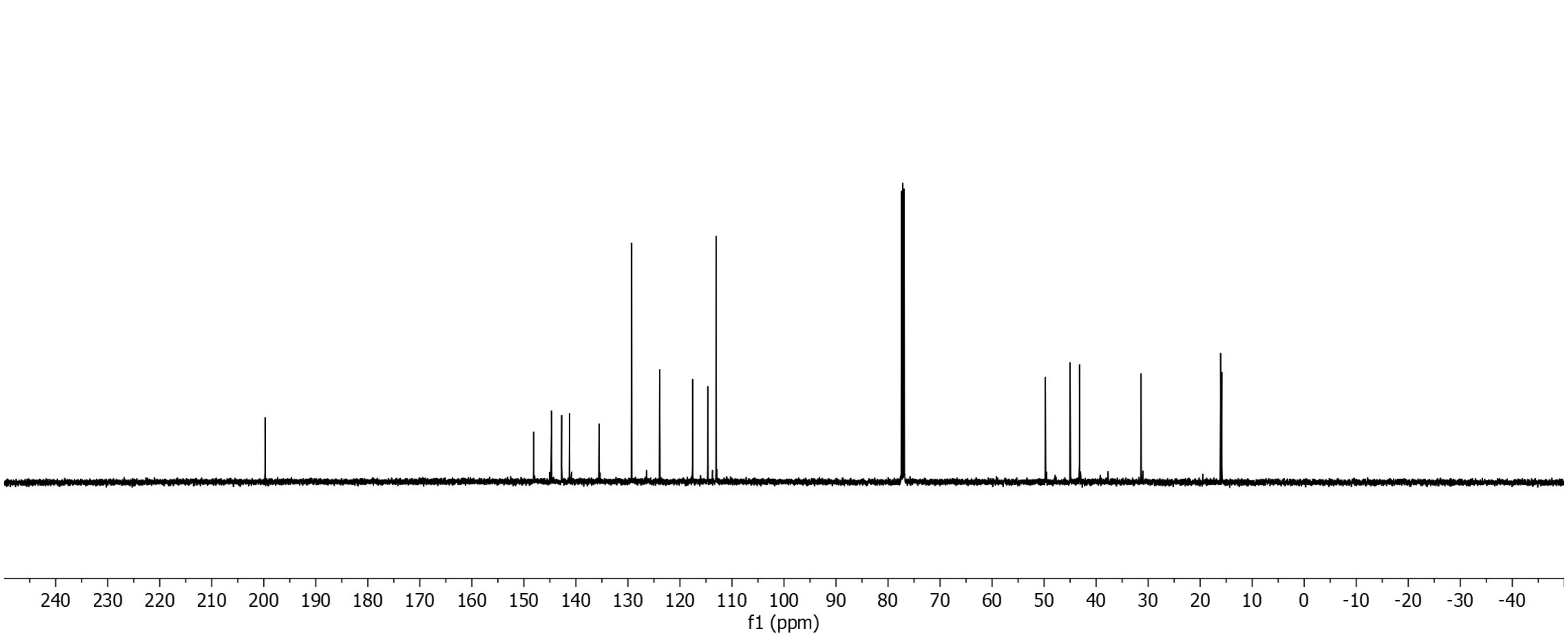

^{1}H NMR of allylic amine 21b

CDCl$_3$, 500 MHz, 23 °C

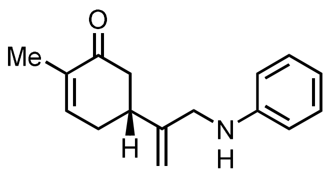

21b

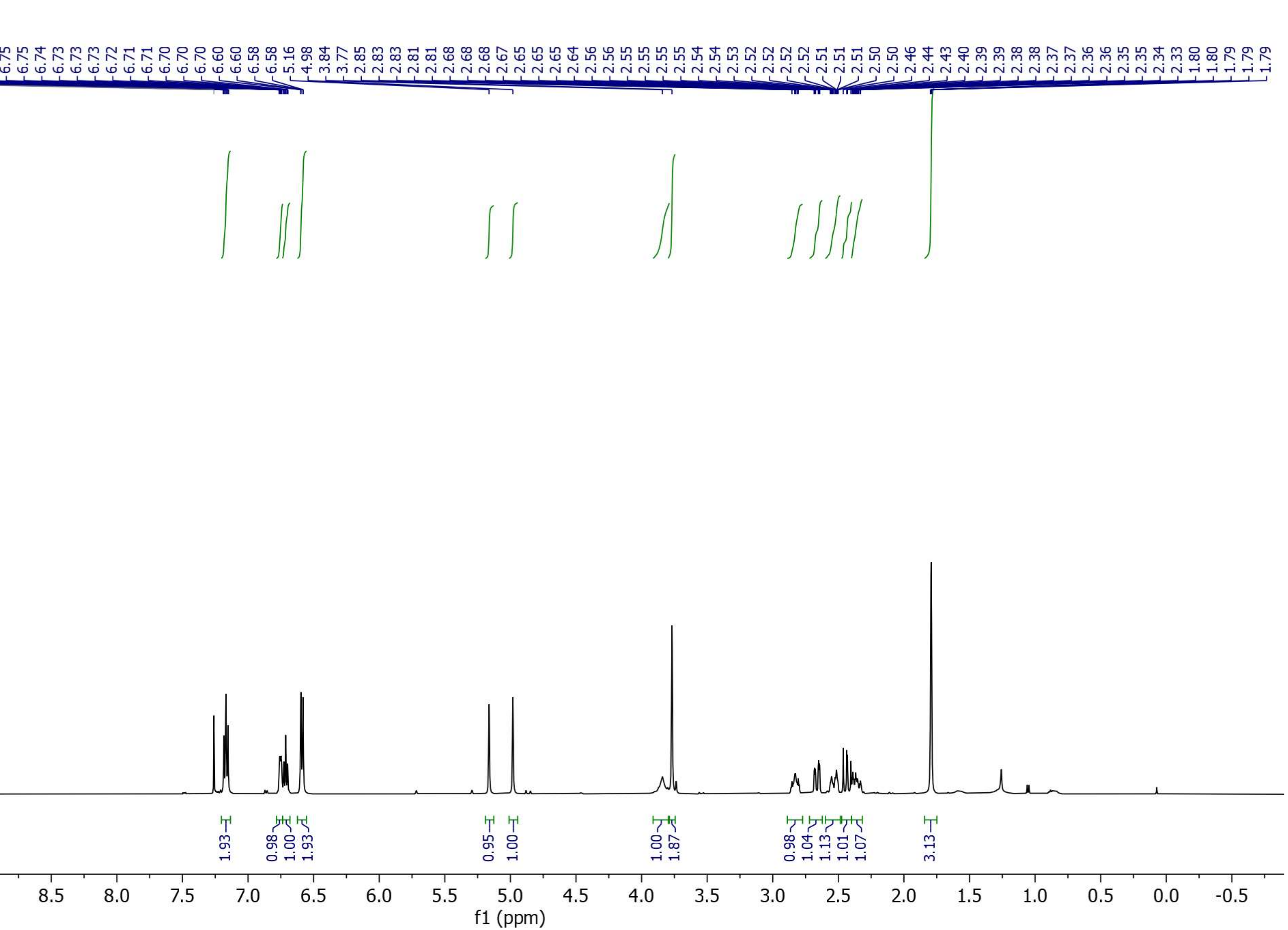

^{13}C NMR of allylic amine 21b

$CDCl_3$, 151 MHz, 23 °C

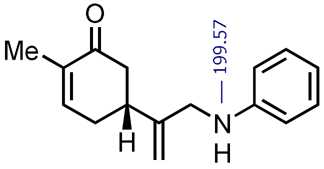

21b

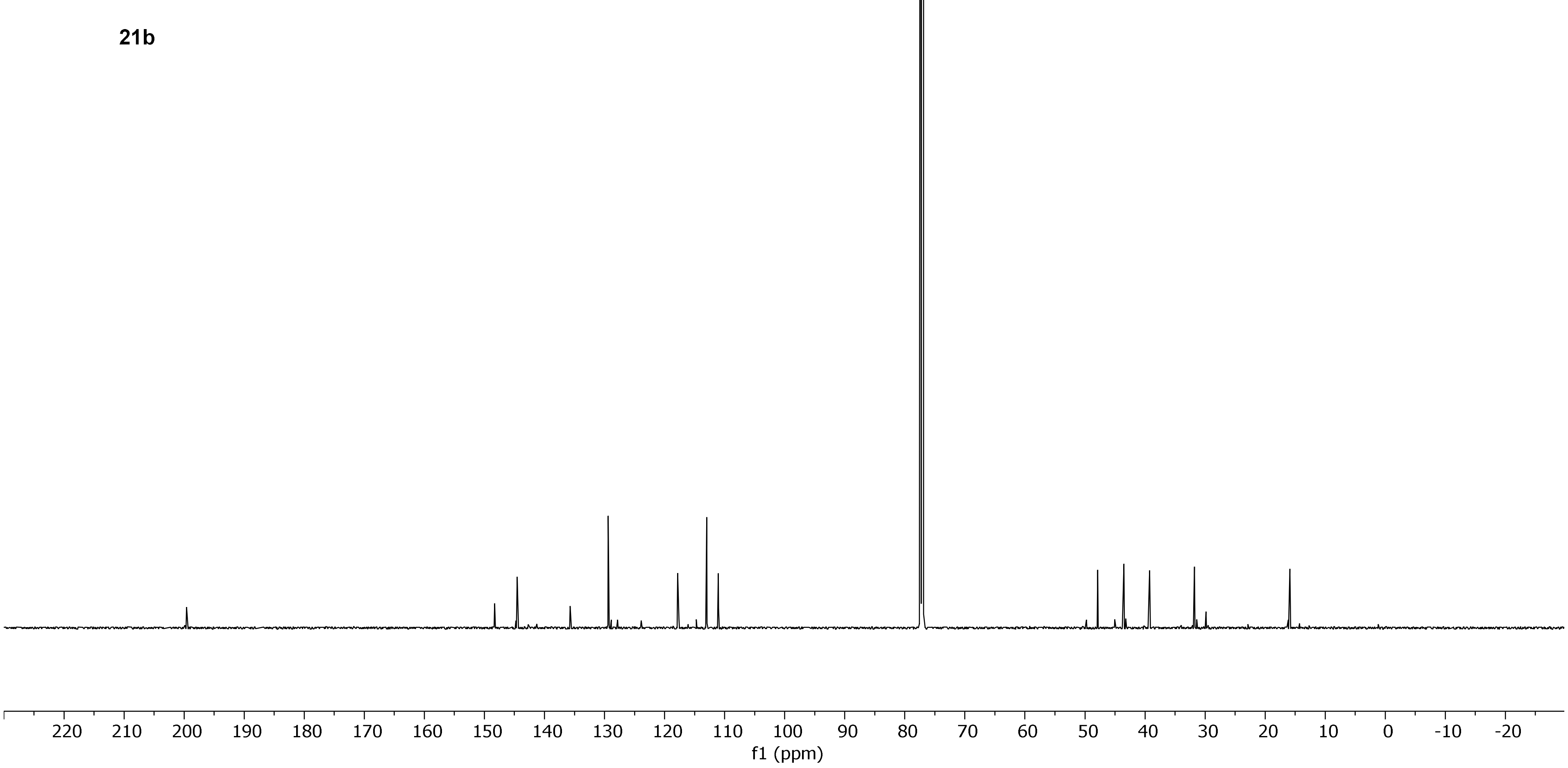

^{1}H NMR of allylic amine 21c

CDCl3, 500 MHz, 23 °C

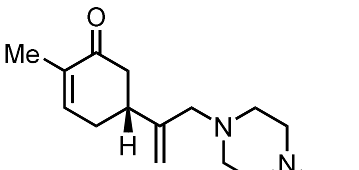

21c

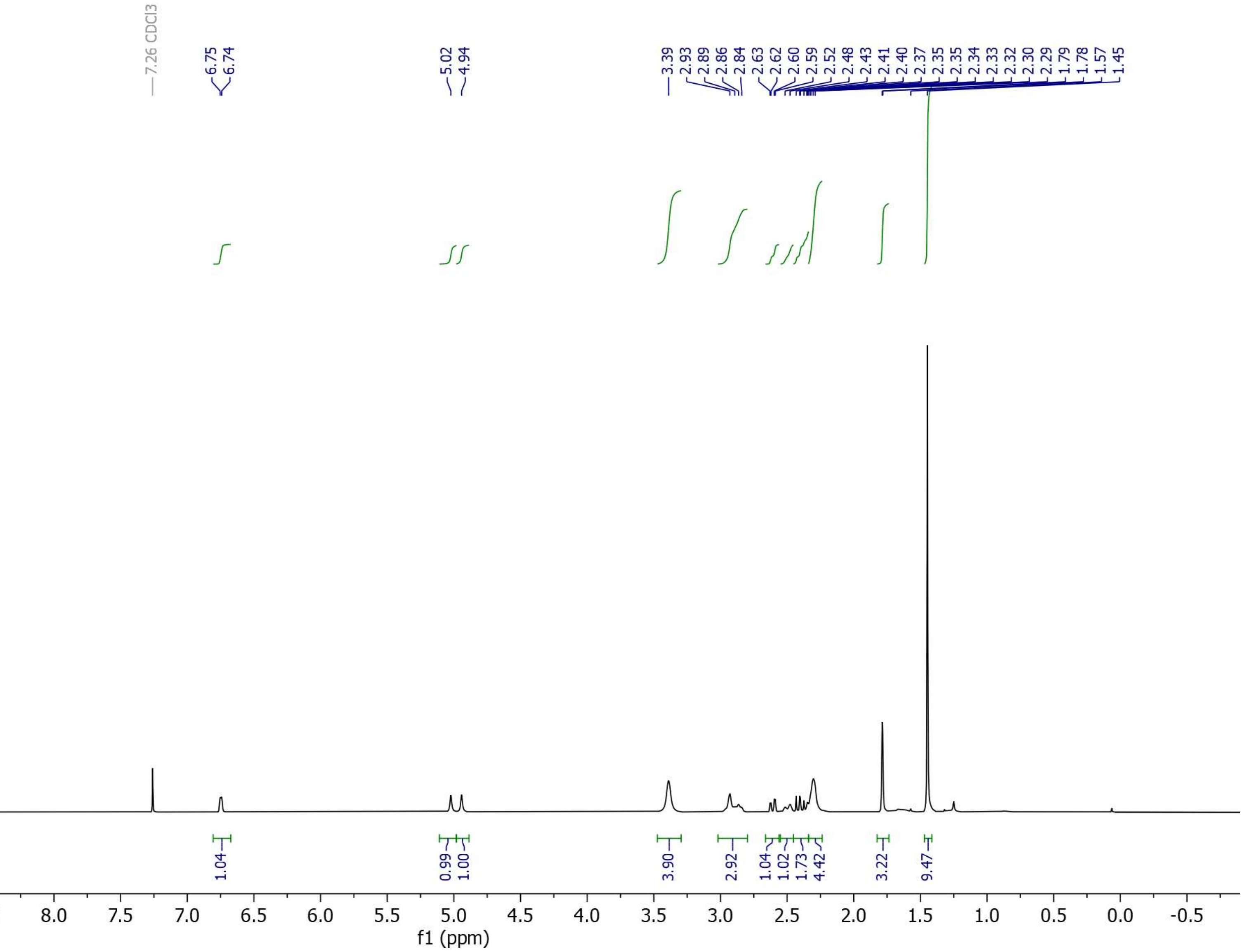

^{13}C NMR of allylic amine 21c

$CDCl_3$, 151 MHz, 23 °C

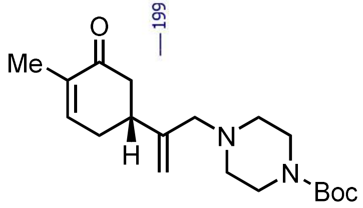

21c

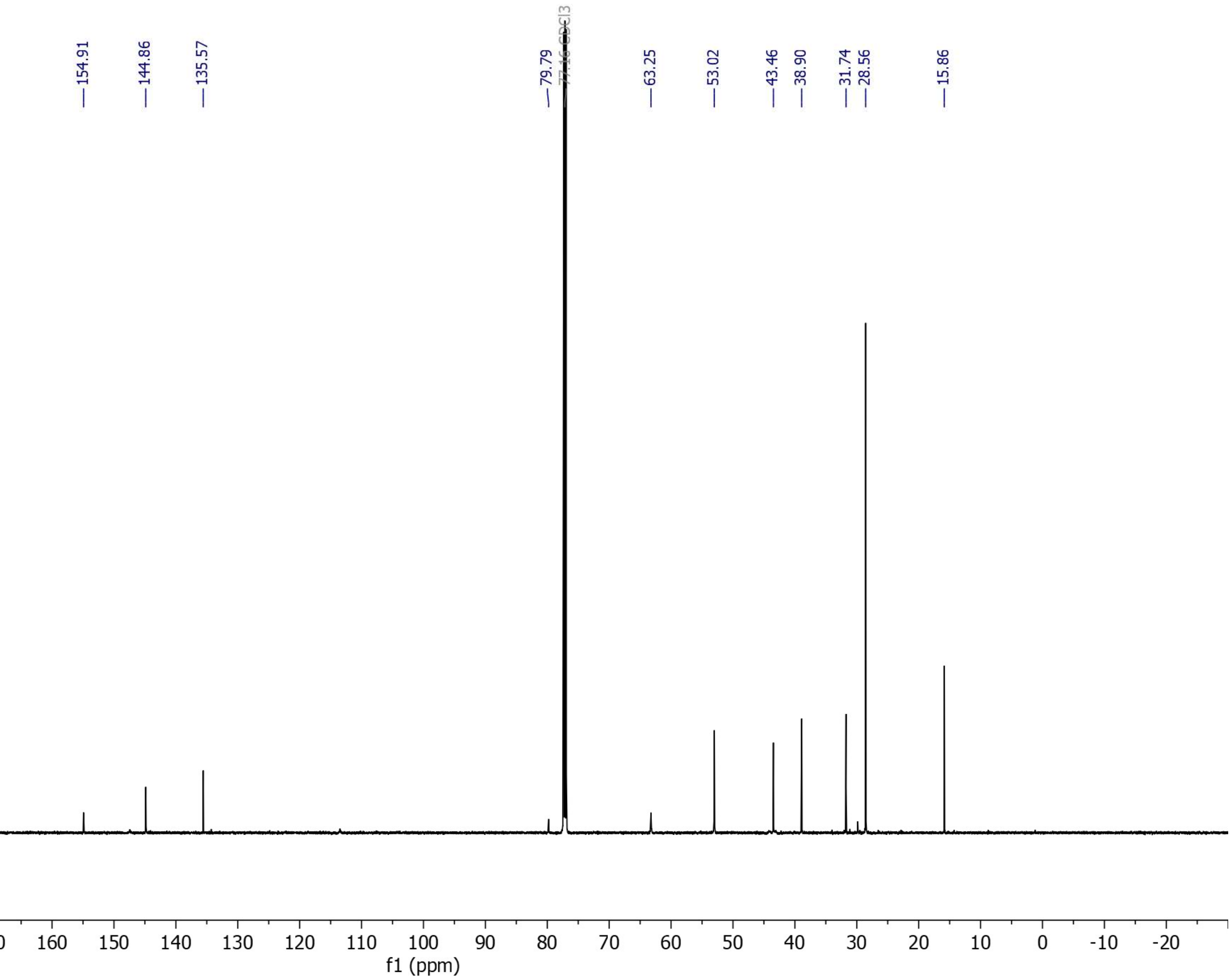

REFERENCES

1. Fulmer, G. R.; Miller, A. J. M.; Sherden, N. H.; Gottlieb, H. E.; Nudelman, A.; Stoltz, B. M.; Bercaw, J. E.; Goldberg, K. I., NMR Chemical Shifts of Trace Impurities: Common Laboratory Solvents, Organics, and Gases in Deuterated Solvents Relevant to the Organometallic Chemist. *Organometallics* **2010**, 29, 2176-2179.
2. *Prudent practices in the laboratory: handling and management of chemical hazards, updated version*. National Academies Press: Washington, DC, 2011.
3. Quan, L. G.; Lee, H. G.; Cha, J. K., Acid- and Pd(0)-Catalyzed Ring Opening of 1-(1-Cycloalkenyl)cyclopropyl Sulfonates. *Org. Lett.* **2007**, 9, 4439-4442.
4. Löber, H.; Kawatsura, M.; Hartwig, J. F., Palladium-Catalyzed Hydroamination of 1,3-Dienes: A Colorimetric Assay and Enantioselective Additions. *J. Am. Chem. Soc.* **2001**, 123.
5. Yang, Q.; Sheng, M.; Henkelis, J. J.; Tu, S.; Wiensch, E.; Zhang, H.; Zhang, Y.; Tucker, C.; Ejeh, D. E., Explosion Hazards of Sodium Hydride in Dimethyl Sulfoxide, N,N-Dimethylformamide, and N,N-Dimethylacetamide. *Org. Process Res. Dev.* **2019**, 23, 2210-2217.
6. Xu, Z.; Jiang, J.; Lu, Q.; Chen, J.; Chen, S.; Shi, L.; Yimei, Z., Photoredox Catalyzed Sulfonylation of Multisubstituted Allenes with Ru(bpy)3Cl2 or Rhodamine B. *J. Organomet. Chem.* **2022**, 957.
7. Baader, S.; Ohlmann, D. M.; Goossen, L. J., Isomerizing ethenolysis as an efficient strategy for styrene synthesis. *Chem. Eur. J.* **2013**, 19, 9807-10.
8. You, C.; Wei, B.; Li, X.; Yang, Y.; Liu, Y.; Lv, H.; Zhang, X., Rhodium-Catalyzed Desymmetrization by Hydroformylation of Cyclopentenes: Synthesis of Chiral Carbocyclic Nucleosides. *Angew. Chem. Int. Ed.* **2016**, 55, 6511-4.
9. Lang, S. B.; O'Nele, K. M.; Douglas, J. T.; Tunge, J. A., Dual Catalytic Decarboxylative Allylations of alpha-Amino Acids and Their Divergent Mechanisms. *Chem. Eur. J.* **2015**, 21, 18589-93.
10. Ahmadli, D.; Müller, S.; Xie, Y.; Smejkal, T.; Jaeckh, S.; Iosub, A. V.; Williams, S. R.; Ritter, T., Standardized Approach for Diversification of Complex Small Molecules via Aryl Thianthrenium Salts. *J. Am. Chem. Soc.* **2025**, 147, 4268-4283.
11. Chen, J.; Li, J.; Plutschack, M. B.; Berger, F.; Ritter, T., Regio- and Stereoselective Thianthrenation of Olefins To Access Versatile Alkenyl Electrophiles. *Angew. Chem. Int. Ed.* **2020**, 59, 5616-5620.
12. Ye, Y.; Zhu, J.; Xie, H.; Huang, Y., Rhodium-Catalyzed Divergent Arylation of Alkenylsulfonium Salts with Arylboroxines. *Angew. Chem. Int. Ed.* **2022**, 61, e202212522.
13. Zhu, J.; Ye, Y.; Huang, Y., Palladacycle-Catalyzed Olefinic C–P Cross-Coupling of Alkenylsulfonium Salts with Diarylphosphines to Access Alkenylphosphines. *Organometallics* **2022**, 41, 2342-2348.
14. Liu, M. S.; Du, H. W.; Cui, J. F.; Shu, W., Intermolecular Metal-Free Cyclopropanation and Aziridination of Alkenes with XH(2) (X=N, C) by Thianthrenation. *Angew. Chem. Int. Ed.* **2022**, 61, e202209929.
15. Sovari, B.; Angyal, P.; Babcsanyi, I. J.; Kotschy, A. M.; Dudas, A.; Turczel, G.; Varga, S.; Soos, T., Indirect Anti-Markovnikov Hydrofunctionalization of Terminal Alkenes via an Alkenyl Thianthrenium Intermediate. *Angew. Chem. Int. Ed.* **2025**, 64, e202424422.
